# Metagenomic Sequencing Identifies Highly Diverse Assemblages of Dinoflagellate Cysts in Sediments from Ships’ Ballast Tanks

**DOI:** 10.3390/microorganisms7080250

**Published:** 2019-08-09

**Authors:** Lixia Shang, Zhangxi Hu, Yunyan Deng, Yuyang Liu, Xinyu Zhai, Zhaoyang Chai, Xiaohan Liu, Zifeng Zhan, Fred C. Dobbs, Ying Zhong Tang

**Affiliations:** 1 CAS Key Laboratory of Marine Ecology and Environmental Sciences, Institute of Oceanology, Chinese Academy of Sciences, Qingdao 266071, China; 2 Laboratory for Marine Ecology and Environmental Science, Qingdao National Laboratory for Marine Science and Technology, Qingdao 266071, China; 3 Center for Ocean Mega-Science, Chinese Academy of Sciences, Qingdao 266071, China; 4 University of Chinese Academy of Sciences, Beijing 100049, China; 5 Department of Marine Organism Taxonomy and Phylogeny, Institute of Oceanology, Chinese Academy of Sciences, Qingdao 266071, China; 6 Department of Ocean, Earth and Atmospheric Sciences, Old Dominion University, Norfolk, VA 23529, USA

**Keywords:** harmful algal blooms, sediment, metabarcoding, invasive species, dinoflagellate resting cysts, Great Lakes, Chesapeake Bay, *Margalefidinium polykrikoides*, single-cell PCR, ballast water

## Abstract

Ships’ ballast tanks have long been known as vectors for the introduction of organisms. We applied next-generation sequencing to detect dinoflagellates (mainly as cysts) in 32 ballast tank sediments collected during 2001–2003 from ships entering the Great Lakes or Chesapeake Bay and subsequently archived. Seventy-three dinoflagellates were fully identified to species level by this metagenomic approach and single-cell polymerase chain reaction (PCR)-based sequencing, including 19 toxic species, 36 harmful algal bloom (HAB) forming species, 22 previously unreported as producing cysts, and 55 reported from ballast tank sediments for the first time (including 13 freshwater species), plus 545 operational taxonomic units (OTUs) not fully identified due to a lack of reference sequences, indicating tank sediments are repositories of many previously undocumented taxa. Analyses indicated great heterogeneity of species composition among samples from different sources. Light and scanning electron microscopy and single-cell PCR sequencing supported and confirmed results of the metagenomic approach. This study increases the number of fully identified dinoflagellate species from ballast tank sediments to 142 (>50% increase). From the perspective of ballast water management, the high diversity and spatiotemporal heterogeneity of dinoflagellates in ballast tanks argues for continuing research and stringent adherence to procedures intended to prevent unintended introduction of non-indigenous toxic and HAB-forming species.

## 1. Introduction

Ships’ ballast water and sediment in ballast tanks have been demonstrated as important vectors for the transportation of exotic phytoplankton among different regions of the world [1,2,3,4,5,6,7]. Although Ostenfeld [8] suggested the diatom *Biddulphia* (= *Odontella*) *sinensis* was introduced by ships in the North Sea in 1903, direct examination of phytoplankton in ballast water was not reported until Carlton’s [9] seminal study. Subsequently, a variety of viable cysts produced by toxic dinoflagellates and diatom resting stages were identified in ballast tanks of vessels entering Australian ports [3,10]. These studies strongly implicated ships’ ballast water and ballast tank sediments as responsible for the dispersal of toxic microalgae.

Being small, ubiquitous, and in some cases, capable of surviving in the dark, phytoplankton are prime candidates for successful transport via ballast tanks [11]. The introduction of non-indigenous species, harmful/toxic dinoflagellates in particular, by ballast tanks is of particular concern due to the potential risk to local fisheries, devastating impacts on ecosystems, and human health considerations [1,5,6,12,13]. Although definitive proof of a particular organism’s introduction via ballasting operations is lacking, many studies have provided strong evidence for the role of ballast tanks in the global dispersal of dinoflagellates, including potentially harmful/toxic forms. For example, Macdonald [14] found dinoflagellate cysts in 90% of sediment samples from ballast tanks of oil and gas tankers in Scottish ports. Hamer et al. [15] identified 48 dinoflagellate species from sediments in ships arriving to English and Welsh ports, including toxic, bloom-forming, and non-indigenous species. Burkholder et al. [16] detected 33 dinoflagellate species, including potentially harmful taxa (e.g., *Dinophysis acuminata, Karlodinium veneficum, Prorocentrum minimum*), from ballast water in U.S. military ships. Casas-Monroy et al. [17] identified cysts of 60 dinoflagellate taxa from sediments in 147 ships arriving to the east and west coasts of Canadian ports and the Great Lakes. One species, *Margalefidinium polykrikoides* (= *Cochlodinium polykrikoides* Margalef, 1961 [18]), can cause massive fish mortality [19], but was not problematic at the ports sampled.

Most biota in ballast tanks decline in abundance during long voyages, indicating death of these species during transit because of stressors such as predation, darkness, low oxygen, and temperature fluctuations within the tank [16,20,21]. But dinoflagellates, many of which form resting cysts under adverse conditions, can sporulate, sink to the bottom of ballast tanks, and if not removed by tank washdowns, can remain viable in the sediments for years [22,23]. Therefore, sediments in tanks likely contain more dinoflagellate species, surviving as resting stages, than the overlying water [10,24,25].

Identification of dinoflagellates from ballast tanks has been based mainly on germination experiments and morphological observation with the assistance of light or electron microscopy [3,15,26]. In addition to being time-consuming and laborious, these methods also require professional taxonomic expertise to identify species from an assemblage of organisms that may be of similar or highly simple morphologies, small in size, or have diagnostic features difficult to recognize. Misidentification is therefore common and almost unavoidable due to these difficulties, as noted by some scientists [18,27]. Advancements in molecular methods (e.g., fluorescence in situ hybridization (FISH), fluorescent quantitative polymerase chain reaction (qPCR), and single-cell PCR), however, have allowed us to identify species accurately from field samples and sometimes to track the origin of a species found at a particular location [28,29,30,31,32]. Notably, Bolch and de Salas [1] used molecular markers, together with a number of other approaches, to convincingly demonstrate that the dinoflagellates *Alexandrium tamarense* and *Gymnodinium catenatum* found in Australasia, were very likely introduced there via ships from Japan and/or other Southeast Asian countries during the past 100 years. Based on comparison of large ribosomal subunit (LSU) and its DNA sequences, Garrett et al. [33] posited that the harmful dinoflagellate, *Vulcanodinium rugosum*, recovered from a ballast tank in Port Tampa Bay (Florida, USA), likely originated from Japan. These studies exemplify molecular detection as a powerful tool in tracking the origins of non-indigenous species.

In particular, next-generation-sequencing (NGS), with its advantages of high-throughput, sensitivity, and specificity, as well as its amenability to automation and miniaturization, can quickly detect and identify species from bulk samples of water or sediment, and has great potential to detect organisms from ships’ ballast tanks. Zaiko et al. [34] applied a metabarcoding approach (targeting the cytochrome oxydase sub-unit I gene and a fragment of the RuBisCO gene) to detect potentially invasive species in ballast water and proposed the potential of NGS in this context. Recently, Shaw et al. [35] also applied the NGS approach to screen algae in historical and modern port and ballast tank sediments, but their primers targeted a 200 base-long fragment of 18S rDNA (including part of the V9 highly variable region). Even with the limited identification power of their primers (i.e., not specific enough), they detected 147 operational taxonomic units (OTUs) of microalgae from 63 samples, but could not identify them to species level.

In this study, we used PCR primers designed to target the large subunit ribosomal (LSU, =28S) rRNA gene (including the most variable D2 domain) of dinoflagellates in concert with high-throughput DNA sequencing to detect dinoflagellate cysts in archived samples of ballast tank sediments from ships that entered ports in the North American Great Lakes or Chesapeake Bay from 2001–2003. Direct light (LM) and scanning electron microscopic (SEM) observations and single-cell PCR and cloning sequencing were also conducted to validate, at least partly, the results of metagenomic sequencing. Although the most common and most abundant dinoflagellate species we found were previously reported from natural marine sediments or ballast tanks, we also found many dinoflagellate taxa that have never been described before or were unreported from ships’ ballast sediments, or both, with some species of particular significance.

## 2. Materials and Methods

### 2.1. Sediment Samples

In late 2015, samples (each 10 to 15 g wet weight) were taken from archived sediments and processed to detect dinoflagellates via metagenomic analyses (see Section 2.2). Archived sediments originally were collected in 2001 to 2003 from empty ballast tanks of ships entering the North American Great Lakes or the Chesapeake Bay, USA. Great Lakes samples (*n* = 27) represented sediment from 27 unique tanks distributed across 16 ships arriving between November 2001 and December 2002 (Table 1). One sample, UNK9074, was collected on 13 August 2002, for which other identifying information was not recovered. These ships were among those surveyed and sampled by Johengen et al. [36], i.e., a total of 103 vessels representing the ocean trade into the Great Lakes. Bulk carriers, chemical tankers, and general/project cargo carriers form the nucleus of the fleet, with dry bulk carriers constituting almost 90% of the entry tonnage. These 103 ships were operated by 55 individual owners or managers and registered in 26 different Flag States. The oldest ship surveyed was built in 1977, and the newest delivered in 2002. Ballast capacities ranged between 1485 m^3^ and 25,533 m^3^, with over 50% of the ballast capacities greater than 10,000 m^3^ [36]. Johengen et al. [36] haphazardly collected and pooled sediment samples from ships’ hopper side tanks, usually from the tank bottom adjacent to or near bilge longitudinal, and sometimes adjacent to the inner bilge girders of double-bottom, forepeak, and side tanks (Table 1). Sediments were collected using sterile trowels and deposited into sterilized buckets, which were covered for transport to the laboratory. Temperature and salinity of residual water were measured directly in each ballast tank using a YSI-85 m (Yellow Springs Instrument, Yellow Springs, OH, USA). In the case of salinity, only a hand-held refractometer was used in the laboratory. Sediments were placed into sterile, screw-cap jars for archival purposes and stored in darkness at 4 °C at Old Dominion University.

Fewer sediment samples (*n* = 4) were collected from ballast tanks of ships in Chesapeake Bay and represented individual tanks from 4 vessels arriving to the Port of Hampton Roads, Virginia between May and November 2003. Sediments were collected and archived as described above [36].

### 2.2. High-Throughput Metagenomic Approach

#### 2.2.1. Primers Design

The forward and reverse primers were designed to amplify the partial 28S rRNA gene (about 540 bases), including the highly variable D2 domain intended mainly for dinoflagellates, with some other closely related taxa such as *Perkinsus*, *Chromera*, and diatoms also being amplified. We chose to amplify this gene fragment because the D1–D5 region of the 28S rRNA gene has been increasingly used for phylogenetic analyses and species identification of dinoflagellates, particularly the highly variable D2 domain [37,38]. Reference sequences of 28S rDNA for some microalgae and ciliates were selected and aligned with those of dinoflagellates to determine areas highly conserved among dinoflagellates. We verified the suitability of the selected oligonucleotide sequences as primers using Primer 3 [39]. The specificity of the generated primer candidates was checked against the GenBank sequence collection by a standard nucleotide–nucleotide BLAST search for the sake of amplifying dinoflagellates only, resulting in the primers as follows: Forward primer LSU347 (5′–CAAGTACCATGAGGGAAA–3′) and reverse primer LSU929 (5′–ACGAACGATTTGCACGTCAGTA–3′), corresponding to the bases 347 and 929, respectively, of the NCBI reference sequence *Alexandrium tamarense* AY831406 and an amplicon of 562 bp encompassing the highly variable D2 domain of 28S rDNA.

#### 2.2.2. DNA Extraction, PCR Amplification, Pyrosequencing, and Data Processing

The total genomic DNA of the sediment samples was extracted from about 0.38–0.45 g wet weight sediment using the Fast DNA SPIN Kit for Soil (MP Biomedicals, Santa Ana, CA, USA) with a Fast PrepTM FP120 cell disrupter (Thermo Electron Corporation, Waltham, Massachusetts, USA). The quantity and quality of total DNA was analyzed with agarose gel electrophoresis and a NanoDropTM 1000 spectrophotometer (Thermo Fisher Scientific, Somerset, NJ, USA).

Samples were 454 pyrosequenced and analyzed by the Shanghai Majorbio Bio-pharm Biotechnology (Shanghai, China). The PCR products were purified using an AxyPrepTM DNA Gel Extraction Kit (Axygen Biosciences, Union City, CA, USA). Pyrosequencing was carried out using a Roche 454 Genome Sequencer FLX Titanium pyrosequencing instrument (454 Life Sciences, Branford, CT, USA) according to the manufacturer’s protocol. The sequencing depth was determined to be 10,000 reads per sample.

After pyrosequencing, the raw reads (567,075) were processed by trimming the adapters and primers using Quantitative Insights Into Microbial Ecology (QIIME, v.1.17, http://qiime.org/) [40]. To obtain high-quality reads, sequencing noises such as nonspecific amplification, chimeric sequences, ambiguous bases and homologous regions were also removed. Some of the criteria used to trim the sequences were as follows: Reads with fewer than 200 bp, reads with blurry bases, primers with more than two mismatched bases, and single base repeated more than 6 times. Ambiguous reads were deleted before the “trim” procedure. Finally, a total of 511,665 clean reads with an average length of 445 bp were obtained. The raw sequencing data set was submitted to the NCBI Short Read Archive (SRA) database under accession number SRP133860. All the clean reads were clustered and aligned to 489,653 effective reads, and then blasted against the NCBI database using Qiime (http://qiime.org/scripts/assign_taxonomy.html) and RDP Classifier [41] (http://sourceforge.net/projects/rdp-classifier/). Many, particularly those with high importance (e.g., species previously not reported to produce cysts) were manually blasted against NCBI database. The software program Usearch (version 7.1, http://drive5.com/uparse/) was used to define OTUs at the 97% similarity level [42].

#### 2.2.3. Data Analyses

OTUs with more than 96% identity and 100% coverage (unless the reference sequence was shorter than our OTUs) to a reference sequence in the NCBI GenBank database were considered as fully identified species (no matter whether or not a full species name was provided to the reference entity). The criterion of 96% identity was mainly based on our experience in identifying dinoflagellates using the LSU rDNA sequences rather than a golden standard from the literature. Other OTUs with lower coverage and identity were manually checked and corrected by blasting the individual sequences in the NCBI BLASTn; some of them were also considered to be fully identified species after considering the lengths of both the reference and blasting sequences. Principal coordinates analysis (PCoA) were conducted at the OTU level using the community ecology package (http://www.mothur.org/), while Venn diagrams were generated using the custom Perl scripts [43].

### 2.3. LM and SEM Observations of Dinoflagellate Cysts

Four ballast tank sediment samples (9055, 9059, 9067, and 9071), identified by molecular metrics as having high species diversity and abundance of cysts were selected for microscopic observations. For SEM observation, the cysts in 2 g wet sediment samples were concentrated with a density gradient centrifugation using sodium polytungstate (SPT) [44]. Cysts were fixed with osmium tetroxide (OsO_4_, 2% final concentration) for 40–50 min, gently filtered onto a 5 μm Millipore nylon membrane, dehydrated in an acetone series (10%, 30%, 50%, 70%, 90%, and 3 times in 100%), critical point-dried (automated critical point dryer, EM CPD 300, LEICA, Austria), sputter-coated with gold (Sputter/Carbon Thread, EM ACE200, LEICA, Austria), and observed with a S-3400N SEM (HiTACHI, Japan). For LM observation, 0.23 g of ballast sediment subsamples were suspended and well dispersed in filtered seawater in a 6-well culturing plate. The cysts were photographed using a digital camera (DP80, Olympus, Japan) coupled to an inverted microscope (IX73, Olympus, Japan).

### 2.4. Single-Cell PCR, Cloning and Sequencing

Some OTUs identified by molecular metrics represented taxa previously unreported in ballast tanks or as cyst producers. We sought to confirm, using single-cell PCR and subsequent cloning and sequencing, that these OTUs were in fact correct identifications. A total of 125 cysts were individually isolated from four ballast tank sediment samples (9045, 9047, 9068, and 9069) after being concentrated with the SPT method [44], washed three times with sterilized double-distilled water, and separately crushed between two sterilized coverslips in a PCR tube. To increase the probability of PCR success, three pairs of universal primers were set [45]: Pair I targets ~1400 bp of LSU rDNA fragment, with the forward primer COM28SF being 5′-ACCCGCTGAATTTAAGCATA, and the reverse primer COM28SR being 5′-GCTACTACCACCAAGATCTGC; Pair II targets ~500 bp of LSU rDNA fragment, with the forward primer SHORT28S1F being 5′-CAAGTACCATGAGGGAAA, and the reverse primer SHORT28S1R being 5′-ACGAACGATTTGCACGTCAGTA; Pair III targets ~500 bp of LSU rDNA fragment, with the forward primer SHORT28S2F being 5′- GCAAGTACCATGAGGG, and the reverse primer SHORT28S1R being 5′-ACGAACGATTTGCACGTCAGTA. A 50 µL PCR reaction system containing 25 µL Hifi Mix I (Trans, Beijing, China), 2 µL of each primer (10 µM), 2 µL BSA (10 mg/mL), and 21 µL nucleotide-free water was added into the PCR tube containing the coverslips. PCR reactions were conducted under the following conditions: For Pair I primers, 94 °C for 5 min, 35 cycles of 94 °C for 40 s, 56 °C for 40 s, 72 °C for 105 s, and a final 10 min extension at 72 °C; for Pair II and Pair III primers, 94 °C for 5 min, 35 cycles of 94 °C for 40 s, 53 °C for 40 s, 72 °C for 35 s, and a final 10 min extension at 72 °C. All PCR products were separated by electrophoresis in 1% agarose gels, then purified using a DNA gel extraction kit (Generay, Shanghai, China). The purified gene fragments were ligated into the PMD18-T vector (Takara, Dalian, China) and transformed to Trans1-T *Escherichia coli* (Trans, Beijing, China). Transformants with correct inserts detected by PCR were sent for sequencing at the Tsingke company (Tsingke, Shanghai, China). All sequences were blasted in GenBank using BLASTn (http://blast.ncbi.nlm.nih.gov/Blast.cgi) for their identities and annotations.

## 3. Results

### 3.1. Salinity and Temperature of the Samples

When sediments were collected from ships’ ballast tanks, salinity of the residual water for the 26 samples in which it was recorded ranged between 1.0 and 36.0 (Table 1), with 54% of the samples having salinity lower than 10.0 (i.e., fresh or brackish water). Water temperatures in ballast tanks were highly variable, from −0.7 °C to 24.5 °C, consistent with sampling over seasons and years in multiple locations.

### 3.2. Results of High-Throughput Metagenomic Sequencing

#### 3.2.1. General Descriptions

At the sequencing depth of 10,000 reads for each sample, a total of 567,075 raw rDNA sequence reads were generated from the 32 samples. All rarefaction curves, except that of sample 9067, approached or were at saturation levels, indicating that overall, sufficient sequences were sampled to detect the majority of taxa in the dinoflagellate assemblages (Figure S1). The rarefaction curves of two other samples (9064 and 9037), while distinct from one another, had plateaus greater than the cluster of curves exhibited by the remaining 29 samples.

A total of 1470 unique operational taxonomic units (OTUs) were obtained (NCBI accession No. SRP133860), 801 of which were dinoflagellate taxa representing 83.0% of the 489,653 effective reads. Although primers amplified the targeted sequences of all dinoflagellates, they also amplified some other eukaryotes, including species of other alveolates and stramenopiles (such as *Perkinsus*, *Chromera*, and diatoms). We determined that 669 OTUs were non-dinoflagellate taxa, presumably amplified by the non-specific reverse primer or mismatches in the forward primer. Among them, 30 OTUs were diatoms, with 12 fully identified to be *Chaetoceros costatus*, *C. socialis*, *C.* sp., *Cyclotella choctawhatcheeana*, *C. meneghiniana*, *Dactyliosolen* sp., *Discostella* sp., *Ditylum brightwellii*, *Navicula erifuga*, *Skeletonema grevillea*, and *Thalassiosira tenera*. These taxa were not included in the following data analyses.

#### 3.2.2. Diversity of Dinoflagellate Taxa

The 801 OTUs identified as dinoflagellates included 10 orders (84 OTUs annotated as not belonging to any known orders), 30 families (98 OTUs annotated as not belonging to any known family), 63 genera (111 OTUs annotated as not belonging to any known genus), and 101 species (298 OTUs not annotated as any known species) (Table S1). OTUs from 34 to 288 (mean = 79) were detected in the 32 samples, while the number of reads varied from 5000 to 23,518 (mean = 12,695) (Figure S2).

Among the 801 dinoflagellate OTUs, 494 could not be convincingly annotated to any known species having sequences deposited in GenBank (e.g., with gaps >50 bps, full-length coverage <85% or similarity <80%). Among the remaining 307 OTUs considered to be fully identified (high identity and coverage to a reference sequence in GenBank), 51 were taxonomically identical to an entity in GenBank but no species name was provided (e.g., *Warnowia* sp. for OTU1577, *Scrippsiell* sp. for OTU1150 and OTU1151, *Stoeckeria* sp. for OTU1636 and OTU1639, etc., Table S2). Together, these 545 OTUs (494 plus 51) might be novel taxa. Since many OTUs were annotated as the same species (e.g., 61 OTUs for *Scrippsiella acuminata*, 37 for *Biecheleria cincta*, 18 for *Biecheleria brevisulcata*, and 11 for *Heterocapsa rotundata*), the remaining 256 OTUs were fully identified and annotated to 71 dinoflagellate species, among which 55 were previously unreported from ships’ ballast tank sediments (Table 2).

#### 3.2.3. Most Abundant and Rarest Taxa

We sorted the 801 OTUs annotated as dinoflagellates according to their total reads and detection rates across all samples. Thirty of the OTUs each had reads >0.9% of total reads across all samples and were considered as the most abundant taxa; together they accounted for 70% of total reads (Table S3). Among them, 14 OTUs were fully identified as 9 species: *Apocalathium baicalense* (freshwater species), *Biecheleria brevisulcata*, *B. cincta*, *Borghiella tenuissima* (freshwater species), *Gyrodinium rubrum*, *Levanderina fissa* (brackish and marine species), *Pelagodinium béii*, *Palatinus apiculatus* (freshwater species) and *Scrippsiella acuminata*. The remaining dominant OTUs (*n* = 16) were likely novel taxa; 14 were not fully identified (definition in Section 2.2.3.) and 2 were “fully identified”, but no species name was provided in GenBank’s reference sequences.

No OTU was detected in all 32 samples, but 16 OTUs were detected in more than 50% of samples. Of these most common OTUs, 11 were fully identified as *Biecheleria brevisulcata*, *B. cincta*, and *Pelagodinium béii* (Table S4).

There were 82 and 50 OTUs having only 1 or 2 read(s), respectively, and were considered as rarest taxa (Table S5). Among these 132 OTUs, 74 were not fully identified, 22 were fully identified but had no species name provided in GenBank, and the remaining 36 were fully identified as belonging to 18 species. Other OTUs of these 18 species were detected at greater frequencies.

There were 366 and 146 OTUs detected only in one or two samples, respectively (Table S6). Among these 512 OTUs, 75% were considered as novel taxa, since they were either not fully identified (320 OTUs) or fully identified but without species names provided in the reference sequences in GenBank (63 OTUs). The remaining 129 OTUs were fully identified as belonging to 56 species.

#### 3.2.4. Habitat Assignments

Fifty-three of the 71 dinoflagellates fully identified and annotated to species level were marine species (Table 2). Among the remaining 18 species, 13 have been described or reported from freshwater only: *Apocalathium aciculiferum*, *A. baicalense*, *A. euryceps*, *Baldinia anauniensis*, *Borghiella dodgei*, *B. tenuissima*, *Chimonodinium lomnickii*, *Naiadinium polonicum*, *Peridiniopsis borgei*, *Palatinus apiculatus*, *Tovellia sanguinea*, *Tyrannodinium edax*, and *Woloszynskia pascheri*. Of the remaining 5 species, *Gymnodinium spirale* [104] and *Biecheleria baltica* [71,72] have been reported from freshwater, brackish, and marine habitats, *Cryptoperidiniopsis brodyi* [78] from brackish water, and *Levanderina fissa* [118] and *Oblea rotunda* [124] from both brackish and marine habitats. These 18 species, except 9043, were present in at least one of the 32 samples.

Six cold water species, i.e., reported with a narrow temperature window for growth (0 °C–6 °C), were identified: *Apocalathium aciculiferum* [58], *Biecheleria baltica* [71,72], *Borghiella dodgei*, *B. tenuissima* [76], *Chimonodinium lomnickii* [77], and *Woloszynskia pascheri* [152].

#### 3.2.5. Harmful and Parasitic Forms

Many of the 71 dinoflagellates fully identified and annotated to species level were potentially harmful/toxic species; 34 bloom-forming and 18 toxic dinoflagellate species were detected (Table 2). Six species of *Alexandrium* were detected from 6 samples, with 5 being paralytic shellfish poisoning (PSP) toxin producers: *A. affine*, *A. fundyense*, *A. ostenfeldii*, *A. pacificum*, *A. peruvianum*, and 1 being a goniodomin producer, *A. pseudogonyaulax* [56]. The number of reads for these 6 species ranged from low (*A. ostenfeldii*) to high (660 reads for *A. pseudogonyaulax*). Seven other well-known toxic dinoflagellates were detected, including the PSP toxin producer *Gymnodinium catenatum* [91], the azaspiracids producers *Azadinium poporum* and *A. polongum*, the yessotoxin producers *Gonyaulax polygramma* [86] and *G. spinifera* [88,89], the aerosol toxin producer *Karenia cristata* [115], and the polyether brevetoxin-2 (PbTx-2) producer *Karenia papilionacea* [2]. Some ichthyotoxic species with toxins, as yet unidentified, were also detected, including *Apocalathium aciculiferum* [58], *Margalefidinium fulvescens* (= *Cochlodinium fulvescens* [18]) [120], *Karlodinium antarcticum* [117,154], and *Takayama helix* [147]. The reactive oxygen species producer *Prorocentrum micans* was detected from one sample but with low abundance [121].

We detected 6 parasitic dinoflagellates: 1 parasitic in ctenophores (*Pentapharsodinium tyrrhenicum* [130], 2 parasitic to copepods (*Blastodinium contortum* [75] and *Dissodinium pseudolunula* [80]), and 3 parasitic to tintinnids (*Duboscquodinium collinii* [83], *Euduboscquella cachoni* [84], and *Euduboscquella crenulata* [85]). Of these, *B. contortum* and the three species associated with tintinnids have not previously been detected in ballast tanks.

#### 3.2.6. Species Previously Unreported to Produce Cysts

The following 22 fully identified species have not been reported to produce resting cysts, but their presence in sediments collected 16 to 18 years previously strongly argues that their cysts were detected here, not their vegetative forms: *Blastodinium contortum*, *Dinophysis lativelata*, *Duboscquodinium collinii*, *Euduboscquella cachoni*, *E. crenulata*, *Gonyaulax polygramma*, *Gymnodinium simplex*, *Gyrodiniellum shiwhaense*, *Gyrodinium dominans*, *G. heterogrammum*, *G. rubrum*, *Heterocapsa minima*, *Karenia cristata*, *K. papilionacea*, *Karlodinium antarcticum*, *Margalefidinium fulvescens*, *Pelagodinium beii*, *Pellucidodinium psammophilum*, *Prorocentrum micans*, *Protoperidinium monovelum*, *Takayama acrotrocha*, and *Takayama helix*. These species were all detected in low abundance (low reads) from 6 or fewer samples each, except for *Pelagodinium beii*, which was detected with a total of 5793 reads across 22 samples, and *Gyrodinium rubrum*, with 4108 reads among 5 samples.

### 3.3. Similarity and Difference in Species Composition Among Samples

**Figure 1:**
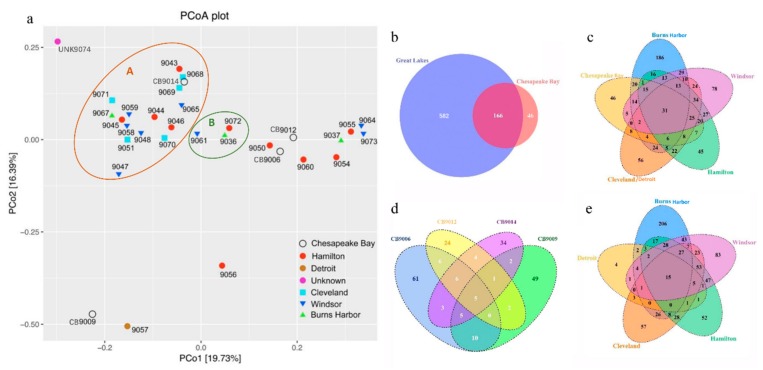
Principal coordinates analysis plot. (**a**) Venn diagrams (b–e) of dinoflagellate taxa at the OTU level in sediments from ships’ ballast tanks. (a) All 32 samples; (**b**) Chesapeake Bay (4 samples) and Great Lakes (27 samples); (**c**) Chesapeake Bay (4 samples), Burns Harbor (3 samples), Cleveland/Detroit (6 samples), Hamilton (10 samples), and Windsor (8 samples); (**d**) 4 samples from Chesapeake Bay; (**e**) five ports of the Great Lakes including Burns Harbor (3 samples), Cleveland (5 samples), Detroit (1 sample), Hamilton (10 samples), and Windsor (8 samples).

PCoA and Venn diagrams were prepared to visualize the similarity and difference of dinoflagellate taxa among different samples at the OTU level. PCoA showed that 16 samples with salinities ranging from 1.0 to 26.0 and three other samples (9061, 9036 and 9072) with salinities ranging from 28.0 to 36.0 formed cluster A and cluster B, respectively (Figure 1a). The remaining 13 samples with a salinity range of 2.0 to 35.0, exhibited little affiliation with one another (Figure 1a). Salinity was in some instances a determinant of species similarity. A related result emerged in considering the port at which sediment was collected. Although dinoflagellate assemblages from samples collected in Cleveland (*n* = 5) all plotted within cluster A, such fidelity was not seen in samples collected in Windsor (*n* = 8) or Hamilton (*n* = 10). Even when samples from two or three tanks were sampled from the same ship on the same day, similarities of species compositions were sometimes high (Ship 1024, Samples 9047 and 9048; Ship 1029, Samples 9058 and 9059; Ship 1014, Samples 9068 and 9069; Ship 1013, Samples 9070 and 9071; Ship 1023, Samples 9044, 9045, and 9046) and sometimes not (Ship 1033, Samples 9064 and 9065; Ship 1027, Samples 9054, 9055, and 9056) (Figure 1a). These PCoA results showed heterogeneity of species composition among samples collected at different times from different ships at different locations, and at times, even different tanks from the same ship.

The Venn diagram comparing the Chesapeake Bay and the Great Lakes showed that samples taken from these two areas shared 166 of the 794 OTUs detected (Figure 1b). In the 166 OTUs, 19 dinoflagellates were fully identified to species level, with 11 known cyst-producers (*Biecheleria baltica*, *B. brevisulcata*, *B. cincta*, *Borghiella tenuissima*, *Gonyaulax spinifera*, *Levanderina fissa*, *Pentapharsodinium dalei*, *Polykrikos kofoidii*, *Scrippsiella acuminate*, *Tyrannodinium edax*, and *Woloszynskia pascheri*), and 8 species previously unreported to produce cysts (*Blastodinium contortum*, *Dinophysis lativelata, Euduboscquella crenulate, Gyrodiniellum shiwhaense*, *Gyrodinium heterogrammum*, *G. rubrum*, *Pelagodinium béii*, and *Polykrikos geminatum*). Ports were assigned to five major categories (Chesapeake Bay, Burns Harbor, Cleveland/Detroit, Hamilton, and Windsor) and compared; they shared only 31 OTUs, with all of the fully identified species being common cyst producers (*Biecheleria brevisulcata*, *B. cincta*, and *Scrippsiella acuminate*), except for *Pelagodinium béii* and *Dinophysis lativelata* (Figure 1c). The four samples from the Chesapeake Bay shared only 5 of 212 OTUs (Figure 1d), while the five ports of the Great Lakes shared only 15 of 748 OTUs (Figure 1e). These Venn diagrams clearly demonstrated high dissimilarity in species composition among samples (sampling ports, ships, and even different tanks of the same ship).

### 3.4. LM and SEM Micrographs of Cysts

**Figure 2:**
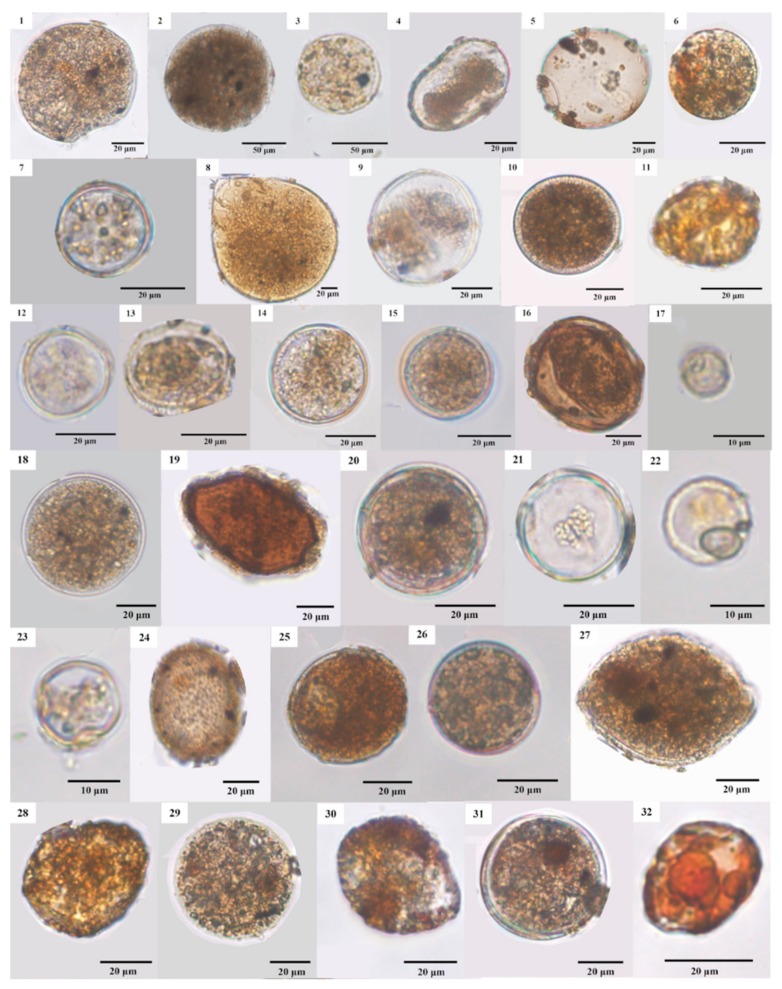
Light microscopic micrographs of cysts from Sample 9055. Except for *Protoperidinium* sp. (5), *Alexandrium* sp. (7, 15 and 22), and *Scrippsiella* sp. (24), all other cysts were not convincingly identified due to the absence of both identification references and diagnostic features.

**Figure 3:**
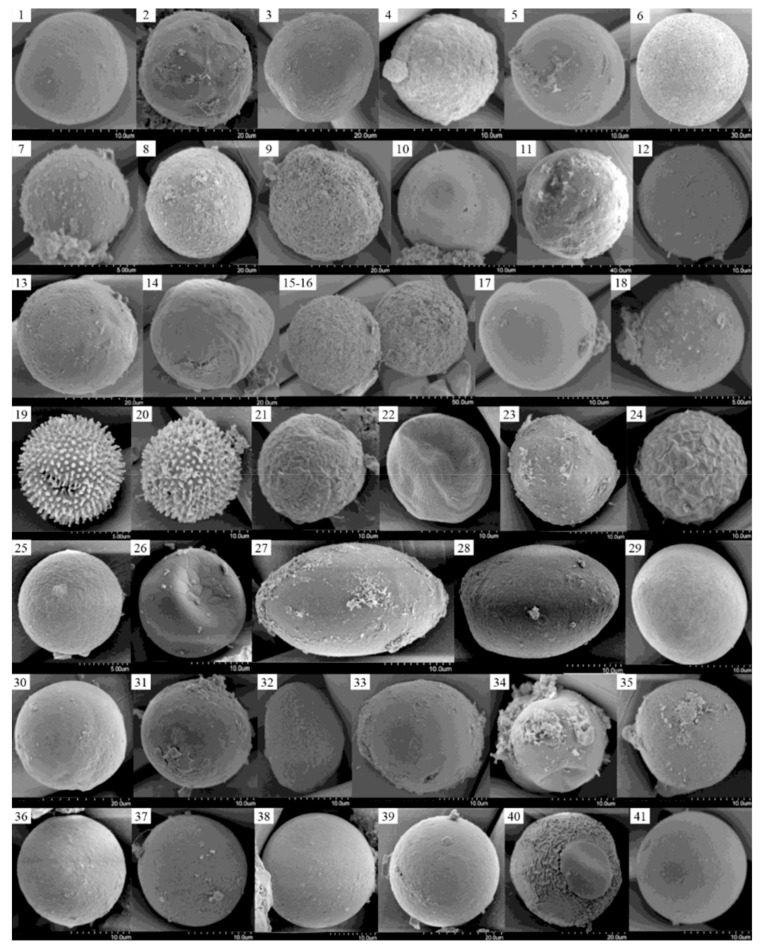
Scanning electron microscopic micrographs of cysts from Samples 9055 (1–18), 9059 (19–26), 9067 (27–32) and 9071 (33–41). Except for *Scrippsiella* sp. (19 and 20), all other cysts were not convincingly identified due to the absence of both identification references and diagnostic features.

To compare identifications determined using metagenomic sequencing to those made using microscopic techniques, we began by choosing four representative samples (Samples 9055, 9059, 9067 and 9071) having high diversity and abundance of cyst species based on their numbers of total reads and OTUs (Figure S2). We did observe many different cyst types in all four samples, with 32 recorded in LM micrographs from Sample 9055 (Figure 2), and 18, 8, 6, and 9 cyst types in SEM micrographs from Samples 9055, 9059, 9067, and 9071, respectively (Figure 3). Not surprisingly, almost all these cysts could not be unambiguously identified due to their smooth surface, simple morphology, or small size. For instance, 23 of the 73 cysts were smaller than 20 μm, with 5 cysts even smaller than 10 μm. With reference to Matsuoka and Fukuyo [155], we tentatively identified the cysts in Figure 2_5 as *Protoperidinium* sp., in Figure 2_7,_15,_22 as *Alexandrium* spp., and in Figure 2_24, Figure 3_19,_20 as *Scrippsiella* spp.

### 3.5. Dinoflagellate Cysts Identified Via Single-Cell PCR Sequencing

**Figure 4:**
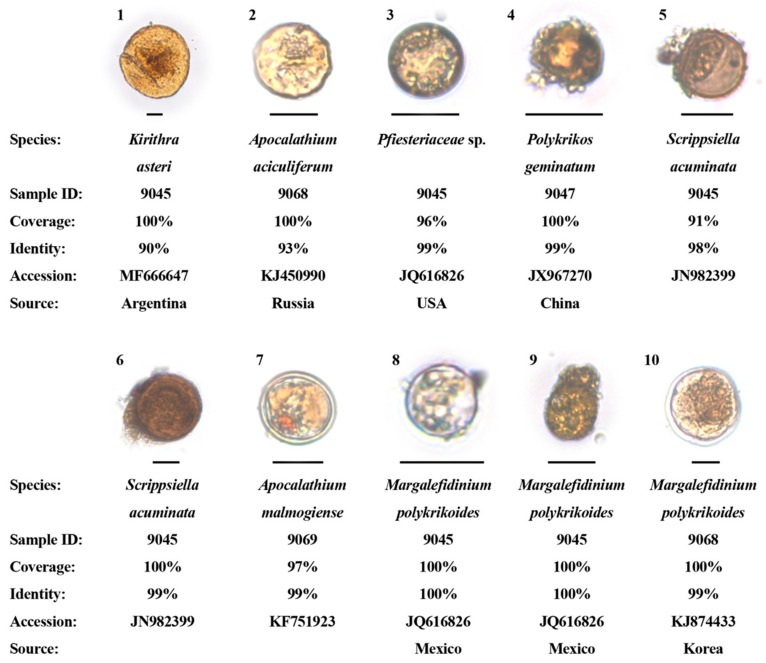
Dinoflagellate cysts identified by single-cell PCR and cloning sequencing from ballast tank sediments collected from ships entering the Great Lakes. Scale bars (lines at the bottom of micrographs of cysts): 20 μm. The term “source” refers to the geographic origin of the reference entities (shown in accession numbers).

Since most dinoflagellate cysts could not be convincingly identified based on microscopic observation alone, we isolated 125 cysts individually from Samples 9045, 9047, 9068, and 9069 and tried to identify them using single-cell PCR and sequencing. Ten of these cysts were successfully sequenced, with 2 not fully identified (identities 90% (Figure 4_1) and 93% (Figure 4_2)), 1 fully identified (*Pfiesteriaceae* sp., identity = 99%) but annotated to genus level (Figure 4_3) and 7 fully identified (with identities > 98%) and annotated to 4 species (*Apocalathium malmogiense*, *Margalefidinium polykrikoides*, *Polykrikos geminatum* and *Scrippsiella acuminata* (also listed in Table 2)). Of these four species, *Polykrikos geminatum*, a species frequently forming HABs in the South China Sea (Figure 4_4) and *Scrippsiella acuminata*, one of the abovementioned 9 most abundant species detected via metagenomic sequencing (Figure 4_5,_6) were also fully identified and annotated to species level by metagenomic sequencing, while *Apocalathium malmogiense* (cold water and HAB-forming species (Figure 4_7)) and *Margalefidinium polykrikoides* (with 3 cysts (Figure 4_8,9,10)) were fully identified and annotated to species level by the single-cell PCR method only (i.e., not identified by high-throughput sequencing). *Margalefidinium polykrikoides* is a notorious toxic and HAB-forming species and we detected its two Ribo-types (i.e., American/Malaysian type and East Asian type). It is noteworthy that the cyst of *Margalefidinium polykrikoides* shown in Figure 4_10 appeared to be germinating, which was quite possible, because the cyst was stored in sterile seawater at 4 °C in a refrigerator for 4 days before single-cell PCR processing after it was isolated from the ballast tank sediment sample.

## 4. Discussion

### 4.1. Dinoflagellates in Ships’ Ballast Tanks—Comparison with Previous Studies

To our knowledge, the present study is the first to use high-throughput NGS and 28S rRNA gene sequencing to identify dinoflagellates from ballast tank sediments. In samples collected and archived 16 to 18 years previously, highly diverse assemblages of dinoflagellate taxa were detected in ships entering the Great Lakes or Chesapeake Bay. PCoA analysis suggested the salinity of water overlying sediments sometimes influenced the composition of the dinoflagellate assemblages they contained. Venn diagrams showed that although cysts of some species commonly found in marine sediments, e.g., *Biecheleria brevisulcata*, *B. cincta*, and *Scrippsiella acuminata*, were present in many samples and at most ports, overall there was a limited number of OTUs shared among ports, ships, and sometimes even between samples collected on the same day from different tanks of the same ship. This high level of heterogeneity in dinoflagellate composition among different sources highlights the considerable possibility of transporting non-indigenous species from one port to another and is consistent with the paradigm that the species composition of microorganisms in a ship’s ballast tanks should be considered principally in the context of an individual tank’s ballasting history [24,29].

Metagenomic sequencing revealed the existence of many dinoflagellate species previously unreported from ballast tank sediments, and in some cases, belonging to higher-level taxa not yet described. We assume that the dinoflagellate assemblages we detected existed only as resting cysts after 16 to 18 years in cold (4 °C) and dark storage. Over that length of time, we assume the DNA of vegetative cells would have degraded substantially; therefore, only DNA of resting cysts would amplify. Our assumption is supported in that: (1) The most abundant and frequently detected species were cyst formers (except *Pelagodinium béii*), abundant and common in natural marine sediment samples; (2) more than 70% of the fully identified species in this study are proven cyst producers (Table 2); (3) light and scanning electron microscopy detected many dinoflagellate cysts, although, unsurprisingly, most could not be identified with certainty for reasons considered in the Introduction; and (4) single-cell PCR sequencing of individual cysts confirmed high-throughput NGS identification of *Polykrikos geminatum* and *Scrippsiella acuminata* cysts, and in addition, detected the cyst of *Apocalathium malmogiense* and cysts of two Ribo-types of *Margalefidinium polykrikoides*, the cyst production of which has been recently confirmed [156,157]. Admittedly, the possibility that some sequences, particularly those with extremely low reads, represented fragments of cells or even DNA relics, cannot be excluded.

We conducted a comprehensive literature review of dinoflagellates previously detected in ballast tank sediments, ballast water, or both (Table S7). Across 48 publications, 14 and 37 dinoflagellates have been reported in tank sediments or ballast water, respectively. Upper values for the number of dinoflagellate species (total taxa) reported in tank sediments are 33 (53) [10]; 39 (60) [17]; and 42 (54) [64]. In comparison, surveys of dinoflagellate species (taxa) in ballast water yielded greater maximum values, e.g., 114 (159) [57] and 142 (155) [26].

We combined results from the literature with those of the present study and listed all fully identified species detected from ballast tank sediments and ballast water in Table S8 and Table S9, respectively. The present study identified 73 species of dinoflagellates with convincing identity from sediments, 71 species fully identified and annotated to species level by high-throughput NGS and two by single-cell PCR sequencing (Table 2). Of these 73 species, 55 are reported here for the first time (Table S8), a much greater number than in any of the 13 previous studies of tank sediments that employed traditional microscopic identification and germination techniques. Further, these 73 species constitute 84% of the total number of dinoflagellate species identified from all previous surveys combined (87 species). The species count in the present study is very likely an underestimate, as it excludes 545 OTUs not fully annotated due to limitations in reference sequences currently available in databases. Combining our results with those of all previous surveys, a total of 142 and 238 dinoflagellate species have been detected from ballast tank sediments (Table S8) and ballast water samples (Table S9), respectively. Taking all OTUs into consideration, however, our metagenomic sequencing results support the idea that tank sediments likely contain many more dinoflagellate species than in the overlying water [10,24,25].

There are a number of reasons for the presumed underestimation of dinoflagellate species number in previous reports. Firstly, small dinoflagellates are hard to identify using traditional, light microscopic visualization, as was the case with our microscopic observations. In contrast, using molecular markers, we detected nine small-sized species (i.e., < 15 µm in length and width): *Biecheleria cincta* [27], *Biecheleriopsis adriatica* [74], *Cryptoperidiniopsis brodyi* [78], *Gyrodiniellum shiwhaense* [99], *Heterocapsa minima* [107], *Heterocapsa rotundata* [108], *Pelagodinium beii* [126], *Polarella glacialis* [133], and *Gymnodinium simplex* [96]. Of these, only *Heterocapsa rotundata* has been previously reported from ballast tank sediment [110] (Table 2). Secondly, it is difficult to distinguish species with similar or highly simple morphological characteristics with light microscopy, especially for species with smooth and thin cyst walls. Thirdly, with reference to probability of encounter, species with extremely low abundance can be easily overlooked, if encountered at all (e.g., the number of reads for *Gyrodinium undulans*, *Heterocapsa minima* and *Pellucidodinium psammophilum* was only one each). Finally, many dinoflagellates have been described as new species only very recently, and thus reference micrographs or sequences were not available for earlier investigations. Indeed, 28 of the 73 fully identified dinoflagellates in this study were described or re-described between 2009 and 2019 (Table 2). Among them, several *Alexandrium* species we found were not detected or reported in earlier publications, save one: *A. pacificum* was reported as *A. catenella* in ballast sediment [10,36] and in ballast water [21,51].

Twenty-two fully identified dinoflagellate species having no prior evidence of being cyst-formers were identified in this study. Among these, only *Prorocentrum micans* has been reported previously in tank sediments, but only as vegetative cells [51,82,87,90,139,140]. All other 21 species are reported from tank sediments for the first time here.

Our LM and SEM micrographs exemplify that most cysts in ballast tank sediments cannot be identified unambiguously, due to their very simple morphology (lacking well-defined diagnostic features), very small sizes, or very similar morphology to other cysts. While dinoflagellate cysts can be identified using single-cell PCR, applying the method to a large-scale investigation is ill-advised, because it is time-consuming, laborious, costly, and its success is not certain. That stated, there remain limitations to a metagenomic approach, e.g., it relies on the accuracy and species number of the GenBank sequences, it cannot, at present, quantify the absolute abundance of dinoflagellates and/or their cysts, and it must balance detection of many groups of organisms with as much taxonomic specificity as possible [158]). Nonetheless, a metagenomic approach has greater detection powers in terms of its high-throughput, identification accuracy, and capability to identify intra-specific and even intra-individual genetic diversity, and therefore provides a method almost indispensable and complementary to the traditional, morphology-based approach.

### 4.2. Both Marine and Freshwater Dinoflagellate Species Found

The structure of dinoflagellate assemblages in ballast tank sediments is influenced by the species composition of waters where ships take on ballast and the tolerance of those species to environmental conditions in the tanks. In turn, those sediments reflect particulate material remaining in the ship, integrated across multiple ballasting operations and for some ships, from fresh, brackish, and marine waters. In this study, marine dinoflagellate species were predictably ubiquitous in the tank sediments. Species having freshwater or brackish habitats, however, were also found in all samples (salinities between 1.0 and 36.0, except sample 9043 (salinity = 8.3)), in which no freshwater species were detected.

Although most marine dinoflagellate species cannot live in freshwater habitats, and vice versa, some estuarine and oceanic species can tolerate a wide range of salinities (0–45) and even grow in both fresh and saline waters [159]. For instance, *Biecheleria baltica* [71,72] and *Gymnodinium spirale* [104] have been reported in freshwater, brackish and marine habitats, while *Cryptoperidiniopsis brodyi* [78] commonly occurs in estuaries from Florida to Maryland. Furthermore, cysts of some marine dinoflagellates such as *Scrippsiella acuminata* can germinate in freshwater media and some strains of *Pfiesteria* can grow at salinities as low as 1–5 [48,160]. This euryhaline nature increases the likelihood that some dinoflagellates could establish populations in freshwater environments. Indeed, marine dinoflagellate cysts are common representatives in ballast tank sediments of transoceanic ships entering the freshwater Great Lakes [17,48].

Thirteen freshwater dinoflagellate species distributed among 10 genera (*Baldinia*, *Borghiella*, *Borghiella*, *Chimonodinium*, *Palatinus*, *Peridiniopsis*, *Peridinium*, *Tovellia*, *Tyrannodinium*, and *Woloszynskia*) were detected in more than 81% of the samples. Compared to freshwater invertebrates such as rotifers and bivalves [161], and phytoplankton such as diatoms and cyanobacteria [139,162], only a few freshwater dinoflagellate species have been previously reported in ballast tanks, e.g., *Apocalathium aciculiferum* [16] and *Ceratium hirundinela* [51]. In the present study, all dinoflagellate species identified as freshwater forms, except *Apocalathium aciculiferum,* were found in ballast tanks for the first time. The freshwater species *Apocalathium baicalense*, *Borghiella tenuissima* (bloom-forming species), and *Palatinus apiculatus* were among the most abundant species in the samples.

### 4.3. Potentially Harmful and/or Toxic Species

HABs may cause numerous negative impacts on marine ecosystems, wild fish stocks, aquaculture, and even human health [163,164,165,166,167]. In particular, some microalgae produce phycotoxins to obtain a competitive advantage, and they may accumulate in the marine food chain and eventually cause poisoning in humans [167,168,169,170]. In the context of HABs, some dinoflagellates are of concern due to their adverse impacts. In this study, bloom-forming dinoflagellate species were present in all samples and with respect to habitat type, were distributed across coastal to oceanic waters and cold to tropical regions. Although the presence of bloom-forming species in ballast tanks does not equate with their ability to establish populations outside their natural range, their tolerance and adaptability to a variety of environmental conditions conceivably threaten non-native environments.

Potentially toxic algal species have long been found in ships’ ballast water and tank sediments and the present study reinforces that observation: 19 toxic dinoflagellate species were identified (Table 2). Members of the genus *Alexandrium,* well-known for production of PSP toxins, are especially well documented in this context. Of the six species we found, *A. tamarense* is thought to have been introduced to Australia from Japan and Korea via ballast water transport [1], and has been observed in ballast tanks of ships arriving at Argentine ports [51], Canadian coasts [17,24,26], English and Welsh ports [15,64] and the Great Lakes [17,48]. We also detected *A. fundyense, A. pacificum*, *A. affine* (reported previously as *A. affinis* [36,48]), *A. ostenfeldii, A. pseudogonyaulax*, and *A. peruvianum*, with the latter reported in ballast sediments here for the first time. A little-known species in this genus, isolates of *A. peruvianum* from Malaysian waters and the Mediterranean Sea have been associated with production of PSP toxins [53] and spirolides [54].

We detected another well-known PSP producer, *Gymnodinium catenatum*, previously found in ships entering the Great Lakes [17,48] and demonstrated to cause shellfish poisoning worldwide [95,171,172,173,174]. We also identified *Gonyaulax spinifera*, isolates of which were documented to produce yessotoxin in New Zealand waters [88], and which were detected in tank sediments of ships entering the Great Lakes [48]. We found a third common species, *Gonyaulax polygramma*, known to cause mass mortality of finfish and shellfish [175,176] and previously discovered in tanks of ships entering Canadian coastal ports [26,57] and Tampa Bay, Florida, USA [87]. The following toxin-producing species are reported from tank sediments here for the first time: *Apocalathium aciculiferum, Azadinium poporum*, *A. polongum*, *Karenia cristata*, *K. papilionacea*, *Karlodinium antarcticum*, *Margalefidinium fulvescens* and *Takayama helix*.

It is noteworthy that we detected the cysts of two Ribo-types of *Margalefidinium polykrikoides* using single-cell PCR sequencing. This dinoflagellate has caused substantial mortality in caged fish with its ichthyotoxic mechanism unknown [177,178,179]. The reported occurrences of HABs caused by *M. polykrikoides* have greatly increased and geographically expanded in the past two decades [177] and its cysts have been found in ballast tanks of ships arriving to multiple ports in Canada and the United States [17,24] and in this study. This study’s detection of its East Asian Ribo-type suggests its expanded distribution to North American waters.

## 5. Conclusions

These results indicate the role of ships in transporting, releasing, and potentially introducing non-indigenous dinoflagellates, and very likely phytoplankton species in general, has been significantly underestimated using traditional methods of identification. The implications highlight the need for those involved with ballast management or interested in biogeography of HAB-forming dinoflagellates (e.g., tracking the source of newly appeared HAB-forming species) to be aware of the high diversity of dinoflagellates transported via ships’ ballast tank sediments and the profound heterogeneity among samples from different sources. In particular, the large number of freshwater species we found argues for practices to minimize their dissemination by ships. Further studies are necessary to determine the viability of dinoflagellates after experiencing ballast tank conditions and to investigate in more detail, the life cycle of dinoflagellates known to have expanded their geographical ranges during recent decades, but which have not been shown to produce resting cysts.

## Figures and Tables

**Table 1:** Sediment samples collected from ballast tanks of ships entering the North American Great Lakes or Chesapeake Bay, USA, in 2001–2003. Identifying information includes collection dates and ports, ship IDs, tank types, and temperature and salinity of residual water in the tanks. Even when the same ship was sampled on a single date, each sample represents a unique tank. Modified from Appendix 2 of Johengen et al. [36].

Sample ID	Sampling Date	Sampling Port	Ship Code	Tank Type	Temperature °C	Salinity
9036	19-Nov-01	Burns Harbor, Indiana, US	1017	DBT	11.7	35.0
9037	19-Nov-01	Burns Harbor, Indiana, US	1017	DBT	11.7	35.0
9043	29-Nov-01	Hamilton, Ontario, Canada	1020	FPK	8.3	7.0
9044	13-Jun-02	Hamilton, Ontario, Canada	1023	FPK	18.5	8.0
9045	13-Jun-02	Hamilton, Ontario, Canada	1023	DBT	18.1	2.0
9046	13-Jun-02	Hamilton, Ontario, Canada	1023	DBT	18.2	6.0
9047	24-Jun-02	Windsor, Ontario, Canada	1024	DBT	21.0	16.0
9048	24-Jun-02	Windsor, Ontario, Canada	1024	DBT	21.0	26.0
9050	19-Jul-02	Hamilton, Ontario, Canada	1025	DBT	21.1	26.0
9051	25-Jul-02	Cleveland, Ohio, US	1026	FPK	22.0	4.0
9054	6-Aug-02	Hamilton, Ontario, Canada	1027	DBT	22.2	5.0
9055	6-Aug-02	Hamilton, Ontario, Canada	1027	DBT	21.9	8.0
9056	6-Aug-02	Hamilton, Ontario, Canada	1027	FPK	23.3	2.0
9057	6-Aug-02	Detroit, Michigan, US	1028	FPK	N/A	N/A
9058	13-Aug-02	Windsor, Ontario, Canada	1029	DBT	24.5	8.0
9059	13-Aug-02	Windsor, Ontario, Canada	1029	DBT	24.5	3.0
9060	15-Aug-02	Hamilton, Ontario, Canada	1030	DBT	N/A	31.0
9061	5-Oct-02	Windsor, Ontario, Canada	1027	FPK	20.2	36.0
9064	20-Oct-02	Windsor, Ontario, Canada	1033	FPK	11.5	2.0
9065	20-Oct-02	Windsor, Ontario, Canada	1033	ST	10.9	1.0
9067	23-Oct-02	Burns Harbor, Indiana, US	1007	DBT	13.6	1.1
9068	12-Nov-02	Cleveland, Ohio, US	1014	DBT	12.3	26.0
9069	12-Nov-02	Cleveland, Ohio, US	1014	DBT	12.3	2.0
9070	19-Nov-02	Cleveland, Ohio, US	1013	DBT	9.8	21.0
9071	19-Nov-02	Cleveland, Ohio, US	1013	DBT	9.2	20.6
9072	26-Nov-02	Hamilton, Ontario, Canada	1034	DBT	7.4	28.0
9073	6-Dec-02	Windsor, Ontario, Canada	1035	FPK	-0.7	34.0
CB9006	16-May-03	Chesapeake, Virginia, US	N/A	FPK	N/A	N/A
CB9009	30-Jul-03	Chesapeake, Virginia, US	N/A	FPK	N/A	N/A
CB9012	3-Oct-03	Norfolk, Virginia, US	N/A	FPK	N/A	N/A
CB9014	21-Nov-03	Chesapeake, Virginia, US	N/A	FPK	N/A	N/A
UNK9074	13-Aug-02	Great Lakes	N/A	N/A	N/A	N/A

FPK = Forepeak tank; DBT = Double-bottom tank; ST = Side tanks; N/A = Data not available. CB = Chesapeake Bay; UNK = unknown.

**Table 2:** Dinoflagellate species fully identified in ballast tank sediments collected from ships entering the Great Lakes or Chesapeake Bay, USA.

Species	Synonyms	Habitat	Cyst	Harmful Effects	Reported in BS References	Reported in BW References
*Alexandrium affine* (1995)	*Protogonyaulax affinis* (1985)	M [46]	Y [46]	B/T [47]	Reported as *Alexandrium affinis* [36,48]	N
*Alexandrium fundyense* (1985)	*Alexandrium tamarense* species complex group I, *Alexandrium tamarense* (1995)	M [49]	Y [49]	B/T [49]	N	N
*Alexandrium ostenfeldii* (1985)	*Protogonyaulax ostenfeldii* (1985), *Triadinium ostenfeldii* (1981), *Gessnerium ostenfeldii* (1979), *Heteraulacus ostenfeldii* (1970), *Gonyaulax ostenfeldii* (1949), *Goniodoma ostenfeldii* (1904)	M [50]	Y [50]	B/T [50]	N	[26]
*Alexandrium pacificum* (2014)	*Alexandrium tamarense* species complex group IV, *Alexandrium catenella*	M [49]	Y [49]	B/T [49]	Reported as *A. catenella* [10,36]	Reported as *A. catenella* [21,51]
*Alexandrium peruvianum* (1985)		M [52]	Y [52]	B/T [53,54]	N	N
*Alexandrium pseudogonyaulax* (1992)	*Goniodoma pseudogonyaulax* (1952), *Triadinium pseudogonyaulax* (1981)	M [55]	Y [55]	B/T [56]	[15]	[26,57]
*Apocalathium aciculiferum* (2016)	*Peridinium aciculiferum* (1900)	F/C [58,59]	Y [58,60]	T [58]	N	Reported as *Peridinium aciculiferum* [16]
*Apocalathium baicalense* (2016)	*Peridinium baicalense* (1935)	F [59,61]	Y [61]		N	N
*Apocalathium euryceps* (2016)	*Peridinium euryceps* (1998)	F [59,62]	Y [62]		N	N
*Apocalathium malmogiense* (2016) *	***Scrippsiella hangoei* (1995)**	**M/C** [59,63]	**Y** [63]	**B** [63]	**Reported as *Scrippsiella hangoei*** [15,64]	**N**
*Archaeperidinium saanichi* (2012)		M [65]	Y [65]		N	N
*Azadinium polongum* (2012)		M [66]	Y [66]	T [66]	N	N
*Azadinium poporum* (2011)		M [67]	Y [68]	T [67,68]	N	N
*Baldinia anauniensis* (2007)		F [69]	Y [69]	B [69]	N	N
*Barrufeta bravensis* (2011)		M [70]	Y [70]	B [70]	N	N
*Biecheleria baltica* (2009)	*Woloszynskia halophila* (2005), *Gymnodinium halophilum* (1952)	F/Br/M/C [71,72]	Y [71]	B [71]	N	N
*Biecheleria brevisulcata* (2014)		M [73]	Y [73]		N	N
*Biecheleria cincta* (2012)	*Woloszynskia cincta* (2009)	M [27]	Y [27]		N	N
*Biecheleriopsis adriatica* (2009)		M [74]	Y [74]		N	N
*Blastodinium contortum* (1908)		M [75]	N	P [75]	N	N
*Borghiella dodgei* (2008)		F/C [76]	Y [76]	B [76]	N	N
*Borghiella tenuissima* (2008)	*Woloszynskia tenuissima* (1950), *Gymnodinium tenuissimum* (1894)	F/C [76]	Y [76]	B [76]	N	N
*Chimonodinium lomnickii* (2011)	*Glenodinium lomnickii* (1928), *Peridinium lomnickii* (1916)	F/C [77]	Y [77]	B [77]	N	N
*Cryptoperidiniopsis brodyi* (2006)		Br [78]	Y [78]		N	N
*Dinophysis lativelata* (1967)		M [79]	N		N	N
*Dissodinium pseudolunula* (1978)		M [80]	Y [80]	P [80]	[64]	[26,81,82]
*Duboscquodinium collinii* (1952)		M [83]	N	P [83]	N	N
*Euduboscquella cachoni* (2012)	*Duboscquella cachoni* (1988)	M [84]	N	P [84]	N	N
*Euduboscquella crenulata* (2012)		M [85]	N	P [85]	N	N
*Gonyaulax polygramma* (1883)		M [86]	N	B/T [86]	N	[26,57,87]
*Gonyaulax spinifera* (1866)	*Peridinium spiniferum* (1859)	M [88,89]	Y [88,89]	B/T [88,89]	Reported as *Gonyaulax spinifera* complex [10,15,17,24,36,48,87] and *Spiniferities bentori* [14]	[16,26,57,87,90]
*Gymnodinium catenatum* (1943)		M [91]	Y [92]	B [92]/T [91]	[14,17,24,36,48]	N
*Gymnodinium impudicum* (2000)	*Gyrodinium impudicum* (1995)	M [93]	Y [94]	B [93]	[17]	N
*Gymnodinium microreticulatum* (1999)		M [28,95]	Y [28,95]		N	N
*Gymnodinium simplex* (1921)	*Protodinium simplex* (1908)	M [96]	N	B [96]	N	[97,98]
*Gyrodiniellum shiwhaense* (2011)		M [99]	N		N	N
*Gyrodinium dominans* (1957)		M [100]	N		N	[101]
*Gyrodinium heterogrammum* (1996)		M [102]	N		N	N
*Gyrodinium rubrum* (2004)		M [103]	N		N	N
*Gyrodinium spirale* (1921)	*Gymnodinium spirale* (1881)	F/Br/M [90,104]	Y [105]		[87]	[26,51,57]
*Gyrodinium undulans* (1957)		M [106]	Y [106]		N	N
*Heterocapsa minima* (1989)		M [107]	N	B [107]	N	N
*Heterocapsa rotundata* (1995)	*Katodinium rotundatum* (1965), *Massartia rotundata* (1933), *Amphidinium rotundatum* (1908)	M [108]	Y [108]	B [108,109]	[110]	[6,16,101]
*Heterocapsa triquetra* (1883)	*Glenodinium triquetrum* (1840), *Peridinium triquetra* (1925)	M [108]	Y [108]	B [108]	[15,81]	[16,26,51,57,90,111,112]
*Islandinium tricingulatum* (2013)	*Protoperidinium tricingulatum* (2009)	M [113,114]	Y [113]		N	N
*Karenia cristata* (2003)		M [115]	N	B/T [115]	N	N
*Karenia papilionacea* (2004)		M [116]	N	B [116]/T [2]	N	[112]
*Karlodinium antarcticum* (2008)		M [117]	N	T [117]	N	N
*Levanderina fissa* (2014)	*Gymnodinium instriatum* (2002), *Gyrodinium instriatum* (1963), *Gyrodinium fissum* (1921), *Gymnodinium fissum* (1894)	M/Br [118]	Y [118]		N	N
*Margalefidinium fulvescens* (2017)	*Cochlodinium fulvescens* (2007)	M [18,119]	N	B/T [120]	N	N
*Margalefidinium polykrikoides* (2017)*	***Cochlodinium polykrikoides* (1961)**	**M** [18,120]	**Y** [103]	**B/T** [121]	**Reported as *Cochlodinium polykrikoides*** [17,24]	**N**
*Naiadinium polonicum* (2015)	*Peridiniopsis polonicum* (1968)	F [122,123]	Y [122]	B [122]	N	N
*Oblea rotunda* (1973)	*Peridiniopsis rotunda* (1922)	M/Br [124]	Y [124]		[15,110]	[26,57,111] Reported as *Oblea rotundata* [97,98]
*Palatinus apiculatus* (2009)	*Peridinium palatinum* (1896), *Peridinium apiculatum* (1859), *Glenodinium apiculatum* (1838)	F [125]	Y [125]		N	N
*Pelagodinium beii* (2010)	*Gymnodinium beii* (1987)	M [126,127]	N		N	N
*Pellucidodinium psammophilum* (2015)		M [128]	N		N	N
*Pentapharsodinium dalei* (1986)		M [129]	Y [129]		[14,15,17,24,64]	N
*Pentapharsodinium tyrrhenicum* (1993)	*Peridinium tyrrhenicum* (1990)	M [130]	Y [131]	B/P [130]	[15]	N
*Peridiniopsis borgei* (1904)	*Glenodinium borgei* (1937), *Peridinium borgei* (1910)	F [132]	Y [132]		N	N
*Polarella glacialis* (1999)		M [133]	Y [133]	B [133]	N	N
***Polykrikos geminatum* (2013)**	***Cochlodinium geminatum* (1896), *Gymnodinium geminatum* (1895)**	**M** [134]	**N**	**B** [134]	**N**	**N**
*Polykrikos kofoidii* (1914)		M [135]	Y [135]		[10,17]	[26,57,82,87]
*Prorocentrum micans* (1834)		M [136]	N [137]	B/T [136]	[81]	[16,21,26,51,57,82,87,90,112,138,139,140]
*Protoperidinium monovelum* (1974)	*Peridinium monovelum* (1936)	M [141]	N		N	N
*Protoperidinium steidingerae* (1979)		M [142]	Y [142]		N	N
***Scrippsiella acuminata* (2015)**	***Glenodinium acuminatum* (1899), *Goniodoma acuminatum* (1883), *Heteraulacus acuminatus* (1850), *Peridinium acuminatum* (1836), *Scrippsiella trochoidea* (1976)**	**M** [143,144]	**Y** [143,144]	**B** [144]	**Reported as *Scrippsiella trochoidea*** [10,14,15,17,24,36,48,64,81]	**Reported as *Scrippsiella trochoidea*** [16,21,26,51,57,82,90,139]
*Scrippsiella donghaienis* (2008)		M [145]	Y [145]		N	N
*Scrippsiella sweeneyae* (1965)		M [146]	Y [146]		N	N
*Takayama acrotrocha* (2003)	*Gyrodinium acrotrochum* (1996)	M [147]	N	B [148]	N	N
*Takayama helix* (2003)		M [147]	N	T [147]	N	N
*Tovellia sanguinea* (2006)		F [149]	Y [149]	B [149]	N	N
*Tyrannodinium edax* (2011)	*Tyrannodinium berolinense* (2009)	F [150,151]	Y [150]	B [150]	N	N
*Woloszynskia pascheri* (1973)	*Gymnodinium pascheri* (1954), *Gyrodinium pascheri* (1933), *Glenodinium pascheri* (1916)	F/C [152]	Y [152]	B [152]	N	N

The bold font indicates species fully identified and annotated to species level by both high-throughput metagenomic approach and single-cell PCR sequencing method; the asterisk indicates species fully identified and annotated to species level via single-cell PCR method only; all other species were fully identified and annotated to species level by high-throughput metagenomic approach only. Taxonomic synonyms and if known, habitat, cyst formation, and harmful effects are shown. The last two columns indicate whether the species has been previously reported in ballast tank sediments (BS) or ballast water (BW), respectively. F = Fresh water species; Br = Brackish species; M = Marine species; C = Cold water species; B = Bloom-forming species; T = Toxic species; P = Parasitic species; N = Not reported in references before, or absent; Y = Present. The synonyms are according to information provided by AlgaeBase (http://www.algaebase.org) except *Alexandrium fundyense* [49], *Alexandrium peruvianum* [153] and *Biecheleria baltica* [72], for which synonyms are indicated according to the corresponding references.

## Supplementary Materials

The following are available online at https://www.mdpi.com/2076-2607/7/8/250/s1, Figure S1: Rarefaction curves for all 32 samples showing the sequencing depths, Figure S2: The total number of operational taxonomic units (OTUs) and reads annotated as dinoflagellates in the 32 samples, Table S1: The 801 operational taxonomic units (OTUs) annotated as dinoflagellates with identity, coverage, and annotation to reference sequences in the NCBI GenBank, Table S2: The 51 operational taxonomic units (OTUs) taxonomically identical to an entity in GenBank but having no species name provided, Table S3: The most abundant taxa (each with reads > 0.9% of the total reads), Table S4: The most frequently detected operational taxonomic units (OTUs) (detected in > 50% of the samples) among the 801 OTUs annotated as dinoflagellates, Table S5: The most rare taxa among the 801 operational taxonomic units (OTUs) annotated as dinoflagellates (detected with 1 or 2 reads), Table S6: The least frequently detected operational taxonomic units (OTUs) (detected in 1 or 2 samples), Table S7: Numbers of dinoflagellate taxa reported in ships’ ballast tanks, including the present study, and the studies’ method(s) of identification, Table S8: Dinoflagellate species reported in ballast tank sediments. Species denoted with paleontological names were not included, Table S9: Dinoflagellate species reported in ships’ ballast water.
