# Supplementary material for: Metagenomic Sequencing Identifies Highly Diverse Assemblages of Dinoflagellate Cysts in Sediments from Ships’ Ballast Tanks
Source: Microorganisms. 2019 Aug 9;7(8):250. doi: 10.3390/microorganisms7080250 (PMC6724030; doi:10.3390/microorganisms7080250)
Supplement: Supplementary file 1 [file microorganisms-07-00250-s001.pdf]

microorganisms

MDPI

*Article*

# Metagenomic sequencing identifies highly diverse assemblages of dinoflagellate cysts in sediments from ships' ballast tanks

**Lixia Shang [1,2,3], Zhangxi Hu [1,2,3], Yunyan Deng [1,2,3], Yuyang Liu [1,4], Xinyu Zhai [1,4], Zhaoyang Chai [1,2,3], Xiaohan Liu [1,4], Zifeng Zhan [5], Fred C. Dobbs [6] and Ying Zhong Tang [1,2,3,*]**

[1] CAS Key Laboratory of Marine Ecology and Environmental Sciences, Institute of Oceanology, Chinese Academy of Sciences, 266071 Qingdao, China; lxshang@qdio.ac.cn (L.S.); zhu@qdio.ac.cn (Z.H.); yunyandeng@qdio.ac.cn (Y.D.); lyy9303130@163.com (Y. L.); vesuviusmo@hotmail.com (X.Z.); zhaoyangchai@qdio.ac.cn (Z.C.); 15264269301@163.com (X.L.)
[2] Laboratory for Marine Ecology and Environmental Science, Qingdao National Laboratory for Marine Science and Technology, 266071 Qingdao, China
[3] Center for Ocean Mega-Science, Chinese Academy of Sciences, 266071 Qingdao, China; e-mail@e-mail.com
[4] University of Chinese Academy of Sciences, 100049 Beijing, China
[5] Department of Marine Organism Taxonomy and Phylogeny, Institute of Oceanology, Chinese Academy of Sciences, 266071 Qingdao, China; zzhan@qdio.ac.cn (Z.Z.)
[6] Department of Ocean, Earth and Atmospheric Sciences, Old Dominion University, Norfolk, 23529 Virginia, USA; fdobbs@odu.edu (F.C.D.)
* Correspondence: yingzhong.tang@qdio.ac.cn; Tel.: +86-532-8289-6098 (Y.Z.T.)

## Legends for table and figures

**Table S1.** The 801 operational taxonomic units (OTUs) annotated as dinoflagellates with identity, coverage, and annotation to reference sequences in the NCBI GenBank.

**Table S2.** The 51 operational taxonomic units (OTUs) taxonomically identical to an entity in GenBank but having no species name provided.

**Table S3.** The most abundant taxa (each with reads >0.9% of the total reads).

**Table S4.** The most frequently detected operational taxonomic units (OTUs) (detected in >50% of the samples) among the 801 OTUs annotated as dinoflagellates.

**Table S5.** The most rare taxa among the 801 operational taxonomic units (OTUs) annotated as dinoflagellates (detected with 1 or 2 reads).

**Table S6.** The least frequently detected operational taxonomic units (OTUs) (detected in 1 or 2 samples).

**Table S7.** Numbers of dinoflagellate taxa reported in ships' ballast tanks, including the present study, and the studies' method(s) of identification.

**Table S8.** Dinoflagellate species reported in ballast tank sediments. Species denoted with paleontological names were not included.

**Table S9.** Dinoflagellate species reported in ships' ballast water.

**Figure S1.** Rarefaction curves for all 32 samples showing the sequencing depths.

**Figure S2.** The total number of operational taxonomic units (OTUs) and reads annotated as dinoflagellates in the 32 samples.

**Table S1.** The 801 operational taxonomic units (OTUs) annotated as dinoflagellates with identity, coverage, and annotation to reference sequences in the NCBI GenBank.

| OTU ID | OUT length | Gi number | Identity | Align length | coverage | Taxon |
|---|---|---|---|---|---|---|
| OTU100 | 485 | AB858353 | 97.32 | 485 | 100 | d__Eukaryota;k__Alveolata;p__norank;c__Dinophyceae;o__Suessiales;f__Biecheleriaceae;g__Biecheleria;s__Biecheleria_brevisulcata |
| OTU1007 | 331 | EF616462 | 86.41 | 309 | 92 | d__Eukaryota;k__Alveolata;p__norank;c__Dinophyceae;o__Gymnodiniales;f__Gymnodiniaceae;g__Cochlodinium;s__Cochlodinium_cf._geminatum |
| OTU1010 | 472 | EF616463 | 95.35 | 86 | 18 | d__Eukaryota;k__Alveolata;p__norank;c__Dinophyceae;o__norank;f__norank;g__norank;s__Unclassified DINO |
| OTU1012 | 478 | AB858353 | 92.48 | 479 | 100 | d__Eukaryota;k__Alveolata;p__norank;c__Dinophyceae;o__Suessiales;f__Biecheleriaceae;g__Biecheleria;s__Biecheleria_brevisulcata |
| OTU1013 | 489 | AB858353 | 92.86 | 490 | 100 | d__Eukaryota;k__Alveolata;p__norank;c__Dinophyceae;o__Suessiales;f__Biecheleriaceae;g__Biecheleria;s__Biecheleria_brevisulcata |
| OTU1014 | 435 | EF616464 | 98.68 | 76 | 17 | d__Eukaryota;k__Alveolata;p__norank;c__Dinophyceae;o__norank;f__norank;g__norank;s__Unclassified DINO |
| OTU1017 | 346 | EF616464 | 98.68 | 76 | 22 | d__Eukaryota;k__Alveolata;p__norank;c__Dinophyceae;o__norank;f__norank;g__norank;s__Unclassified DINO |
| OTU1019 | 499 | FJ947042 | 83.53 | 510 | 100 | d__Eukaryota;k__Alveolata;p__norank;c__Dinophyceae;o__Gymnodiniales;f__Warnowiaceae;g__Warnowia;s__Warnowia_sp._BSL-2009a |
| OTU102 | 496 | KJ508360 | 95.77 | 497 | 100 | d__Eukaryota;k__Alveolata;p__norank;c__Dinophyceae;o__Gymnodiniales;f__Kareniaceae;g__Karenia;s__Karenia_cristata |
| OTU1034 | 524 | EU048553 | 98.46 | 520 | 99 | d__Eukaryota;k__Alveolata;p__norank;c__Dinophyceae;o__Peridiniales;f__Pfiesteriaceae;g__unclassified_Pfiesteriaceae;s__Pfiesteriaceae_sp._masanensis |
| OTU1037 | 549 | HQ176320 | 90 | 90 | 35 | d__Eukaryota;k__Alveolata;p__norank;c__Dinophyceae;o__norank;f__norank;g__norank;s__Unclassified DINO |
| OTU1038 | 549 | AY950447 | 89.33 | 75 | 36 | d__Eukaryota;k__Alveolata;p__norank;c__Dinophyceae;o__norank;f__norank;g__norank;s__Unclassified DINO |
| OTU1039 | 546 | AY950447 | 89.33 | 75 | 36 | d__Eukaryota;k__Alveolata;p__norank;c__Dinophyceae;o__norank;f__norank;g__norank;s__Unclassified DINO |
| OTU104 | 248 | KC895475 | 97.14 | 245 | 99 | d__Eukaryota;k__Alveolata;p__norank;c__Dinophyceae;o__Suessiales;f__Biecheleriaceae;g__Biecheleria;s__Biecheleria_cincta |
| OTU1043 | 432 | EU048553 | 97.92 | 433 | 100 | d__Eukaryota;k__Alveolata;p__norank;c__Dinophyceae;o__Peridiniales;f__Pfiesteriaceae;g__unclassified_Pfiesteriaceae;s__Pfiesteriaceae_sp._masanensis |
| OTU105 | 251 | AF318248 | 96.41 | 251 | 99 | d__Eukaryota;k__Alveolata;p__norank;c__Dinophyceae;o__Gymnodiniales;f__Gymnodiniaceae;g__Gymnodinium;s__unclassified_Gymnodinium |
| OTU1051 | 374 | EU048553 | 99.43 | 352 | 94 | d__Eukaryota;k__Alveolata;p__norank;c__Dinophyceae;o__Peridiniales;f__Pfiesteriaceae;g__unclassified_Pfiesteriaceae;s__Pfiesteriaceae_sp._masanensis |
| OTU1058 | 500 | EU126801 | 77.84 | 528 | 100 | d__Eukaryota;k__Alveolata;p__norank;c__Dinophyceae;o__Suessiales;f__Borghiellaceae;g__Borghiella;s__Borghiella_dodgei |
| OTU1059 | 488 | AY571374 | 84.05 | 489 | 99 | d__Eukaryota;k__Alveolata;p__norank;c__Dinophyceae;o__Lophodiniales;f__Lophodiniaceae;g__Woloszynskia;s__Woloszynskia_tenuissima |

| | | | | | | |
|---|---|---|---|---|---|---|
| OTU1061 | 507 | AY571374 | 82.75 | 510 | 100 | d__Eukaryota;k__Alveolata;p__norank;c__Dinophyceae;o__Lophodiniales;f__Lophodiniaceae;g__Woloszynskia;s__Woloszynskia_tenuissima |
| OTU1063 | 501 | AY571374 | 81.32 | 514 | 100 | d__Eukaryota;k__Alveolata;p__norank;c__Dinophyceae;o__Lophodiniales;f__Lophodiniaceae;g__Woloszynskia;s__Woloszynskia_tenuissima |
| OTU1064 | 512 | JN119844 | 77.15 | 547 | 100 | d__Eukaryota;k__Alveolata;p__norank;c__Dinophyceae;o__Peridiniales;f__Heterocapsaceae;g__Heterocapsa;s__Heterocapsa_sp._HCBC88 |
| OTU1066 | 507 | JN119844 | 76.14 | 549 | 100 | d__Eukaryota;k__Alveolata;p__norank;c__Dinophyceae;o__Peridiniales;f__Heterocapsaceae;g__Heterocapsa;s__Heterocapsa_sp._HCBC88 |
| OTU1068 | 506 | AY571374.1 | 81 | 506 | 100 | d__Eukaryota;k__Alveolata;p__norank;c__Dinophyceae;o__Lophodiniales;f__Lophodiniaceae;g__Woloszynskia;s__Woloszynskia_sp. |
| OTU1069 | 504 | AY571374 | 79.35 | 523 | 100 | d__Eukaryota;k__Alveolata;p__norank;c__Dinophyceae;o__Lophodiniales;f__Lophodiniaceae;g__Woloszynskia;s__Woloszynskia_tenuissima |
| OTU1070 | 487 | AY571374 | 76.2 | 500 | 100 | d__Eukaryota;k__Alveolata;p__norank;c__Dinophyceae;o__Lophodiniales;f__Lophodiniaceae;g__Woloszynskia;s__Woloszynskia_tenuissima |
| OTU1071 | 513 | JN119844 | 76.35 | 554 | 100 | d__Eukaryota;k__Alveolata;p__norank;c__Dinophyceae;o__Peridiniales;f__Heterocapsaceae;g__Heterocapsa;s__Heterocapsa_sp._HCBC88 |
| OTU1072 | 487 | AY571374 | 84.6 | 487 | 99 | d__Eukaryota;k__Alveolata;p__norank;c__Dinophyceae;o__Lophodiniales;f__Lophodiniaceae;g__Woloszynskia;s__Woloszynskia_tenuissima |
| OTU1073 | 501 | AY571374 | 83.83 | 507 | 100 | d__Eukaryota;k__Alveolata;p__norank;c__Dinophyceae;o__Lophodiniales;f__Lophodiniaceae;g__Woloszynskia;s__Woloszynskia_tenuissima |
| OTU1074 | 512 | JN119844 | 76.17 | 554 | 100 | d__Eukaryota;k__Alveolata;p__norank;c__Dinophyceae;o__Peridiniales;f__Heterocapsaceae;g__Heterocapsa;s__Heterocapsa_sp._HCBC88 |
| OTU1075 | 477 | EU126801 | 98.74 | 478 | 100 | d__Eukaryota;k__Alveolata;p__norank;c__Dinophyceae;o__Suessiales;f__Borghiellaceae;g__Borghiella;s__Borghiella_dodgei |
| OTU1076 | 481 | AY571374 | 81.24 | 517 | 100 | d__Eukaryota;k__Alveolata;p__norank;c__Dinophyceae;o__Lophodiniales;f__Lophodiniaceae;g__Woloszynskia;s__Woloszynskia_tenuissima |
| OTU1078 | 490 | AY571374 | 81.14 | 493 | 99 | d__Eukaryota;k__Alveolata;p__norank;c__Dinophyceae;o__Lophodiniales;f__Lophodiniaceae;g__Woloszynskia;s__Woloszynskia_tenuissima |
| OTU1079 | 486 | AY571374 | 84.39 | 487 | 98 | d__Eukaryota;k__Alveolata;p__norank;c__Dinophyceae;o__Lophodiniales;f__Lophodiniaceae;g__Woloszynskia;s__Woloszynskia_tenuissima |
| OTU1081 | 503 | EU126801 | 75.73 | 515 | 100 | d__Eukaryota;k__Alveolata;p__norank;c__Dinophyceae;o__Suessiales;f__Borghiellaceae;g__Borghiella;s__Borghiella_dodgei |
| OTU1082 | 509 | EU126801 | 79.62 | 520 | 100 | d__Eukaryota;k__Alveolata;p__norank;c__Dinophyceae;o__Suessiales;f__Borghiellaceae;g__Borghiella;s__Borghiella_dodgei |
| OTU1083 | 488 | AY571374 | 83.5 | 491 | 99 | d__Eukaryota;k__Alveolata;p__norank;c__Dinophyceae;o__Lophodiniales;f__Lophodiniaceae;g__Woloszynskia;s__Woloszynskia_tenuissima |
| OTU1085 | 507 | EU126801.1 | 80 | 540 | 100 | d__Eukaryota;k__Alveolata;p__norank;c__Dinophyceae;o__Suessiales;f__Borghiellaceae;g__Borghiella;s__Borghiella_sp. |
| OTU1088 | 512 | JN119844 | 75.27 | 558 | 100 | d__Eukaryota;k__Alveolata;p__norank;c__Dinophyceae;o__Peridiniales;f__Heterocapsaceae;g__Heterocapsa;s__Heterocapsa_sp._HCBC88 |

| | | | | | | |
|---|---|---|---|---|---|---|
| OTU1090 | 500 | AY571374 | 82.02 | 534 | 100 | d__Eukaryota;k__Alveolata;p__norank;c__Dinophyceae;o__Lophodiniales;f__Lophodiniaceae;g__Woloszynskia;s__Woloszynskia_tenuissima |
| OTU1091 | 514 | HQ902267 | 76.44 | 556 | 100 | d__Eukaryota;k__Alveolata;p__norank;c__Dinophyceae;o__Peridiniales;f__Heterocapsaceae;g__Heterocapsa;s__Heterocapsa_triquetra |
| OTU1092 | 510 | JN119844 | 76.97 | 547 | 100 | d__Eukaryota;k__Alveolata;p__norank;c__Dinophyceae;o__Peridiniales;f__Heterocapsaceae;g__Heterocapsa;s__Heterocapsa_sp._HCBC88 |
| OTU1097 | 487 | EU126801 | 76.76 | 525 | 100 | d__Eukaryota;k__Alveolata;p__norank;c__Dinophyceae;o__Suessiales;f__Borghiellaceae;g__Borghiella;s__Borghiella_dodgei |
| OTU1098 | 476 | AY571374 | 79.18 | 490 | 99 | d__Eukaryota;k__Alveolata;p__norank;c__Dinophyceae;o__Lophodiniales;f__Lophodiniaceae;g__Woloszynskia;s__Woloszynskia_tenuissima |
| OTU1099 | 496 | AY571374 | 77.92 | 530 | 100 | d__Eukaryota;k__Alveolata;p__norank;c__Dinophyceae;o__Lophodiniales;f__Lophodiniaceae;g__Woloszynskia;s__Woloszynskia_tenuissima |
| OTU110 | 413 | KF031312 | 76.43 | 420 | 99 | d__Eukaryota;k__Alveolata;p__norank;c__Dinophyceae;o__Peridiniales;f__Heterocapsaceae;g__Heterocapsa;s__Heterocapsa_minima |
| OTU1101 | 489 | AY571374 | 79.11 | 517 | 100 | d__Eukaryota;k__Alveolata;p__norank;c__Dinophyceae;o__Lophodiniales;f__Lophodiniaceae;g__Woloszynskia;s__Woloszynskia_tenuissima |
| OTU1103 | 485 | AY571374 | 80.67 | 507 | 100 | d__Eukaryota;k__Alveolata;p__norank;c__Dinophyceae;o__Lophodiniales;f__Lophodiniaceae;g__Woloszynskia;s__Woloszynskia_tenuissima |
| OTU1104 | 478 | AY571374 | 81.93 | 487 | 97 | d__Eukaryota;k__Alveolata;p__norank;c__Dinophyceae;o__Lophodiniales;f__Lophodiniaceae;g__Woloszynskia;s__Woloszynskia_tenuissima |
| OTU1105 | 505 | AY571374 | 81.84 | 523 | 100 | d__Eukaryota;k__Alveolata;p__norank;c__Dinophyceae;o__Lophodiniales;f__Lophodiniaceae;g__Woloszynskia;s__Woloszynskia_tenuissima |
| OTU1110 | 501 | AY571374 | 80.9 | 513 | 100 | d__Eukaryota;k__Alveolata;p__norank;c__Dinophyceae;o__Lophodiniales;f__Lophodiniaceae;g__Woloszynskia;s__Woloszynskia_tenuissima |
| OTU1112 | 494 | AY571374 | 82.34 | 504 | 100 | d__Eukaryota;k__Alveolata;p__norank;c__Dinophyceae;o__Lophodiniales;f__Lophodiniaceae;g__Woloszynskia;s__Woloszynskia_tenuissima |
| OTU1113 | 528 | EU126801.1 | 94 | 91 | 49 | d__Eukaryota;k__Alveolata;p__norank;c__Dinophyceae;o__norank;f__norank;g__norank;s__Unclassified DINO |
| OTU1115 | 281 | EU126801 | 96.47 | 283 | 100 | d__Eukaryota;k__Alveolata;p__norank;c__Dinophyceae;o__Suessiales;f__Borghiellaceae;g__Borghiella;s__Borghiella_dodgei |
| OTU1117 | 235 | EF058276 | 83.33 | 234 | 99 | d__Eukaryota;k__Alveolata;p__norank;c__Dinophyceae;o__Lophodiniales;f__Lophodiniaceae;g__Woloszynskia;s__Woloszynskia_pascheri |
| OTU1119 | 500 | AY571374 | 82.82 | 489 | 96 | d__Eukaryota;k__Alveolata;p__norank;c__Dinophyceae;o__Lophodiniales;f__Lophodiniaceae;g__Woloszynskia;s__Woloszynskia_tenuissima |
| OTU1138 | 506 | JN119844 | 82.84 | 507 | 100 | d__Eukaryota;k__Alveolata;p__norank;c__Dinophyceae;o__Peridiniales;f__Heterocapsaceae;g__Heterocapsa;s__Heterocapsa_sp._HCBC88 |
| OTU1146 | 522 | HM483396 | 88.7 | 522 | 100 | d__Eukaryota;k__Alveolata;p__norank;c__Dinophyceae;o__Peridiniales;f__Thoracosphaeraceae;g__Scrippsiella;s__Scrippsiella_trochoidea |
| OTU1150 | 317 | EU165289 | 97.16 | 317 | 100 | d__Eukaryota;k__Alveolata;p__norank;c__Dinophyceae;o__Peridiniales;f__Thoracosphaeraceae;g__Scrippsiella;s__Scrippsiella_sp._CCMP2775 |

| | | | | | | |
|---|---|---|---|---|---|---|
| OTU1151 | 261 | EU165289 | 98.43 | 255 | 98 | d__Eukaryota;k__Alveolata;p__norank;c__Dinophyceae;o__Peridiniales;f__Thoracosphaeraceae;g__Scrippsiella;s__Scrippsiella_sp._CCMP2775 |
| OTU1158 | 236 | HQ845331 | 94.92 | 236 | 100 | d__Eukaryota;k__Alveolata;p__norank;c__Dinophyceae;o__Peridiniales;f__Thoracosphaeraceae;g__Scrippsiella;s__Scrippsiella_sweeneyae |
| OTU1161 | 313 | HM483396 | 93.29 | 313 | 100 | d__Eukaryota;k__Alveolata;p__norank;c__Dinophyceae;o__Peridiniales;f__Thoracosphaeraceae;g__Scrippsiella;s__Scrippsiella_trochoidea |
| OTU1162 | 501 | KF751924 | 92.48 | 532 | 100 | d__Eukaryota;k__Alveolata;p__norank;c__Dinophyceae;o__Peridiniales;f__Thoracosphaeraceae;g__Scrippsiella;s__Scrippsiella_aff._acuminata |
| OTU1178 | 487 | HQ670228.1 | 98 | 487 | 100 | d__Eukaryota;k__Alveolata;p__norank;c__Dinophyceae;o__Peridiniales;f__Thoracosphaeraceae;g__Scrippsiella;s__Scrippsiella_trochoidea |
| OTU1180 | 295 | EU370959 | 96.96 | 296 | 100 | d__Eukaryota;k__Alveolata;p__norank;c__Dinophyceae;o__Peridiniales;f__Thoracosphaeraceae;g__Scrippsiella;s__Scrippsiella_trochoidea |
| OTU1182 | 549 | KT804914.1 | 90 | 95 | 18 | d__Eukaryota;k__Alveolata;p__norank;c__Dinophyceae;o__norank;f__norank;g__norank;s__Unclassified DINO |
| OTU1183 | 487 | AY154965 | 74.64 | 414 | 81 | d__Eukaryota;k__Alveolata;p__norank;c__Dinophyceae;o__Gonyaulacales;f__Gonyaulacaceae;g__Gonyaulax;s__Gonyaulax-like_sp. |
| OTU1184 | 544 | KT390028.1 | 95.22 | 544 | 100 | d__Eukaryota;k__Alveolata;p__norank;c__Dinophyceae;o__ Blastodiniales;f__norank;g__Blastodinium;s__Blastodinium_sp. |
| OTU1188 | 477 | KT390028.1 | 89.1 | 477 | 100 | d__Eukaryota;k__Alveolata;p__norank;c_Dinophyceae;o_ Blastodiniales;f_norank;g_Blastodinium;s_Blastodinium_sp. |
| OTU1189 | 532 | KT390028.1 | 95.86 | 532 | 100 | d__Eukaryota;k__Alveolata;p__norank;c_Dinophyceae;o_ Blastodiniales;f_norank;g_Blastodinium;s_Blastodinium_contortum |
| OTU1198 | 526 | KT390028.1 | 95.27 | 486 | 92 | d__Eukaryota;k__Alveolata;p__norank;c_Dinophyceae;o_ Blastodiniales;f_norank;g_Blastodinium;s_Blastodinium_sp. |
| OTU12 | 523 | EU126801 | 74.54 | 538 | 100 | d__Eukaryota;k__Alveolata;p__norank;c__Dinophyceae;o__Suessiales;f__Borghiellaceae;g__Borghiella;s__Borghiella_sp. |
| OTU1201 | 497 | FJ947042 | 86.08 | 510 | 100 | d__Eukaryota;k__Alveolata;p__norank;c__Dinophyceae;o__Gymnodiniales;f__Warnowiaceae;g__Warnowia;s__Warnowia_sp._BSL-2009a |
| OTU1202 | 487 | AF200669 | 79.84 | 501 | 99 | d__Eukaryota;k__Alveolata;p__norank;c__Dinophyceae;o__Gymnodiniales;f__Gymnodiniaceae;g__Lepidodinium;s__Lepidodinium_chlorophorum |
| OTU1308 | 494 | EF469233.1 | 75 | 519 | 99 | d_Eukaryota;k_Alveolata;p_norank;c_Dinophyceae;o_Gymnodiniales;f_Kareniaceae;g_Karlodinium-like;s_Karlodinium-like_sp. |
| OTU1314 | 497 | FR720082 | 81.71 | 514 | 100 | d__Eukaryota;k__Alveolata;p__norank;c__Dinophyceae;o__Gymnodiniales;f__Gymnodiniaceae;g__Gyrodiniellum;s__Gyrodiniellum_shiwhaense |
| OTU1316 | 500 | KP790188 | 91.71 | 422 | 84 | d__Eukaryota;k__Alveolata;p__norank;c__Dinophyceae;o__Gymnodiniales;f__Gymnodiniaceae;g__Gymnodinium;s__Gymnodinium_sp. |
| OTU1318 | 533 | KP790233 | 93.58 | 436 | 82 | d__Eukaryota;k__Alveolata;p__norank;c__Dinophyceae;o__Gymnodiniales;f__Brachidiniaceae;g__Torodinium;s__Torodinium_teredo |
| OTU1319 | 509 | FJ024703 | 98.43 | 509 | 100 | d__Eukaryota;k__Alveolata;p__norank;c__Dinophyceae;o__Gymnodiniales;f__Kareniaceae;g__Takayama;s__Takayama_acrotrocha |

| | | | | | | |
|---|---|---|---|---|---|---|
| OTU1320 | 495 | FJ024703 | 96.38 | 497 | 100 | d__Eukaryota;k__Alveolata;p__norank;c__Dinophyceae;o__Gymnodiniales;f__Kareniaceae;g__Takayama;s__Takayama_acrotrocha |
| OTU1321 | 489 | FJ024705 | 100 | 489 | 100 | d__Eukaryota;k__Alveolata;p__norank;c__Dinophyceae;o__Suessiales;f__Biecheleriaceae;g__Biecheleria;s__Biecheleria_cincta |
| OTU1322 | 497 | FJ024705 | 98.39 | 497 | 100 | d__Eukaryota;k__Alveolata;p__norank;c__Dinophyceae;o__Suessiales;f__Biecheleriaceae;g__Biecheleria;s__Biecheleria_cincta |
| OTU1324 | 501 | FJ024705 | 96.01 | 501 | 100 | d__Eukaryota;k__Alveolata;p__norank;c__Dinophyceae;o__Suessiales;f__Biecheleriaceae;g__Biecheleria;s__Biecheleria_cincta |
| OTU1326 | 497 | AB858353 | 96.39 | 499 | 100 | d__Eukaryota;k__Alveolata;p__norank;c__Dinophyceae;o__Suessiales;f__Biecheleriaceae;g__Biecheleria;s__Biecheleria_brevisulcata |
| OTU1327 | 499 | FJ024705 | 97.6 | 499 | 100 | d__Eukaryota;k__Alveolata;p__norank;c__Dinophyceae;o__Suessiales;f__Biecheleriaceae;g__Biecheleria;s__Biecheleria_cincta |
| OTU1328 | 510 | FJ024705 | 91.73 | 508 | 99 | d__Eukaryota;k__Alveolata;p__norank;c__Dinophyceae;o__Suessiales;f__Biecheleriaceae;g__Biecheleria;s__Biecheleria_cincta |
| OTU1329 | 508 | FJ024705 | 95.44 | 504 | 99 | d__Eukaryota;k__Alveolata;p__norank;c__Dinophyceae;o__Suessiales;f__Biecheleriaceae;g__Biecheleria;s__Biecheleria_cincta |
| OTU1330 | 499 | FJ024705 | 93.99 | 499 | 100 | d__Eukaryota;k__Alveolata;p__norank;c__Dinophyceae;o__Suessiales;f__Biecheleriaceae;g__Biecheleria;s__Biecheleria_cincta |
| OTU1331 | 510 | FJ024705 | 94.07 | 506 | 99 | d__Eukaryota;k__Alveolata;p__norank;c__Dinophyceae;o__Suessiales;f__Biecheleriaceae;g__Biecheleria;s__Biecheleria_cincta |
| OTU1332 | 494 | FJ024705 | 97.57 | 494 | 100 | d__Eukaryota;k__Alveolata;p__norank;c__Dinophyceae;o__Suessiales;f__Biecheleriaceae;g__Biecheleria;s__Biecheleria_cincta |
| OTU1333 | 500 | FJ024705 | 92.22 | 501 | 100 | d__Eukaryota;k__Alveolata;p__norank;c__Dinophyceae;o__Suessiales;f__Biecheleriaceae;g__Biecheleria;s__Biecheleria_cincta |
| OTU1335 | 494 | FJ024705 | 92.12 | 495 | 100 | d__Eukaryota;k__Alveolata;p__norank;c__Dinophyceae;o__Suessiales;f__Biecheleriaceae;g__Biecheleria;s__Biecheleria_cincta |
| OTU1336 | 487 | FJ024705 | 96.71 | 487 | 100 | d__Eukaryota;k__Alveolata;p__norank;c__Dinophyceae;o__Suessiales;f__Biecheleriaceae;g__Biecheleria;s__Biecheleria_cincta |
| OTU1337 | 499 | FJ024705 | 94.19 | 499 | 100 | d__Eukaryota;k__Alveolata;p__norank;c__Dinophyceae;o__Suessiales;f__Biecheleriaceae;g__Biecheleria;s__Biecheleria_cincta |
| OTU1339 | 485 | FJ024705 | 94.02 | 485 | 100 | d__Eukaryota;k__Alveolata;p__norank;c__Dinophyceae;o__Suessiales;f__Biecheleriaceae;g__Biecheleria;s__Biecheleria_cincta |
| OTU1342 | 485 | FJ024705 | 93.81 | 485 | 100 | d__Eukaryota;k__Alveolata;p__norank;c__Dinophyceae;o__Suessiales;f__Biecheleriaceae;g__Biecheleria;s__Biecheleria_cincta |
| OTU1343 | 490 | FJ024705 | 97.56 | 491 | 100 | d__Eukaryota;k__Alveolata;p__norank;c__Dinophyceae;o__Suessiales;f__Biecheleriaceae;g__Biecheleria;s__Biecheleria_cincta |
| OTU1345 | 477 | FJ024705 | 92.75 | 483 | 100 | d__Eukaryota;k__Alveolata;p__norank;c__Dinophyceae;o__Suessiales;f__Biecheleriaceae;g__Biecheleria;s__Biecheleria_cincta |
| OTU1346 | 489 | FJ024705 | 91.85 | 491 | 100 | d__Eukaryota;k__Alveolata;p__norank;c__Dinophyceae;o__Suessiales;f__Biecheleriaceae;g__Biecheleria;s__Biecheleria_cincta |

| | | | | | | |
|---|---|---|---|---|---|---|
| OTU1347 | 499 | FJ024705 | 92.22 | 501 | 100 | d__Eukaryota;k__Alveolata;p__norank;c__Dinophyceae;o__Suessiales;f__Biecheleriaceae;g__Biecheleria;s__Biecheleria_cincta |
| OTU1349 | 497 | FJ024705 | 92.6 | 500 | 100 | d__Eukaryota;k__Alveolata;p__norank;c__Dinophyceae;o__Suessiales;f__Biecheleriaceae;g__Biecheleria;s__Biecheleria_cincta |
| OTU1351 | 485 | FJ024705 | 95.82 | 502 | 99 | d__Eukaryota;k__Alveolata;p__norank;c__Dinophyceae;o__Suessiales;f__Biecheleriaceae;g__Biecheleria;s__Biecheleria_cincta |
| OTU1353 | 479 | FJ024705 | 93.95 | 479 | 100 | d__Eukaryota;k__Alveolata;p__norank;c__Dinophyceae;o__Suessiales;f__Biecheleriaceae;g__Biecheleria;s__Biecheleria_cincta |
| OTU1354 | 479 | FJ024705 | 92.9 | 479 | 100 | d__Eukaryota;k__Alveolata;p__norank;c__Dinophyceae;o__Suessiales;f__Biecheleriaceae;g__Biecheleria;s__Biecheleria_cincta |
| OTU1356 | 493 | FJ024705 | 93.1 | 493 | 100 | d__Eukaryota;k__Alveolata;p__norank;c__Dinophyceae;o__Suessiales;f__Biecheleriaceae;g__Biecheleria;s__Biecheleria_cincta |
| OTU1357 | 494 | FJ024705 | 93.72 | 494 | 100 | d__Eukaryota;k__Alveolata;p__norank;c__Dinophyceae;o__Suessiales;f__Biecheleriaceae;g__Biecheleria;s__Biecheleria_cincta |
| OTU1359 | 492 | FJ024705 | 95.53 | 492 | 100 | d__Eukaryota;k__Alveolata;p__norank;c__Dinophyceae;o__Suessiales;f__Biecheleriaceae;g__Biecheleria;s__Biecheleria_cincta |
| OTU1360 | 491 | FJ024705 | 92.68 | 492 | 100 | d__Eukaryota;k__Alveolata;p__norank;c__Dinophyceae;o__Suessiales;f__Biecheleriaceae;g__Biecheleria;s__Biecheleria_cincta |
| OTU1361 | 491 | FJ024705 | 90.84 | 491 | 100 | d__Eukaryota;k__Alveolata;p__norank;c__Dinophyceae;o__Suessiales;f__Biecheleriaceae;g__Biecheleria;s__Biecheleria_cincta |
| OTU1363 | 488 | FJ024705 | 93.24 | 488 | 100 | d__Eukaryota;k__Alveolata;p__norank;c__Dinophyceae;o__Suessiales;f__Biecheleriaceae;g__Biecheleria;s__Biecheleria_cincta |
| OTU1364 | 486 | FJ024705 | 92.59 | 486 | 100 | d__Eukaryota;k__Alveolata;p__norank;c__Dinophyceae;o__Suessiales;f__Biecheleriaceae;g__Biecheleria;s__Biecheleria_cincta |
| OTU1365 | 497 | FJ024705 | 91.98 | 499 | 100 | d__Eukaryota;k__Alveolata;p__norank;c__Dinophyceae;o__Suessiales;f__Biecheleriaceae;g__Biecheleria;s__Biecheleria_cincta |
| OTU1366 | 508 | FJ024705 | 94.44 | 504 | 99 | d__Eukaryota;k__Alveolata;p__norank;c__Dinophyceae;o__Suessiales;f__Biecheleriaceae;g__Biecheleria;s__Biecheleria_cincta |
| OTU1367 | 488 | FJ024705 | 90.8 | 489 | 100 | d__Eukaryota;k__Alveolata;p__norank;c__Dinophyceae;o__Suessiales;f__Biecheleriaceae;g__Biecheleria;s__Biecheleria_cincta |
| OTU1369 | 513 | FJ024705 | 84.27 | 515 | 100 | d__Eukaryota;k__Alveolata;p__norank;c__Dinophyceae;o__Suessiales;f__Biecheleriaceae;g__Biecheleria;s__Biecheleria_cincta |
| OTU1370 | 209 | FJ024706 | 98.06 | 206 | 98 | d__Eukaryota;k__Alveolata;p__norank;c__Dinophyceae;o__Lophodiniales;f__Lophodiniaceae;g__Woloszynskia;s__Woloszynskia_sp._MB-1 |
| OTU1372 | 229 | FJ024706 | 98.67 | 225 | 98 | d__Eukaryota;k__Alveolata;p__norank;c__Dinophyceae;o__Lophodiniales;f__Lophodiniaceae;g__Woloszynskia;s__Woloszynskia_sp._MB-1 |
| OTU1400 | 525 | KT390028 | 79 | 525 | 100 | d__Eukaryota;k__Alveolata;p__norank;c__Dinophyceae;o__Blastodiniales; f__Blastodiniales;g__Blastodinium-like;s__Blastodinium-like_sp. |
| OTU1401 | 514 | KT390028 | 91.31 | 518 | 100 | d__Eukaryota;k__Alveolata;p__norank;c__Dinophyceae;o__Blastodiniales; f__Blastodiniales;g__Blastodinium-like;s__Blastodinium-like_sp. |

| | | | | | | |
|---|---|---|---|---|---|---|
| OTU1402 | 517 | KT390028 | 91.68 | 517 | 100 | d__Eukaryota;k__Alveolata;p__norank;c__Dinophyceae;o__Blastodiniales; f__Blastodiniales;g__Blastodinium-like;s__Blastodinium-like_sp. |
| OTU1404 | 528 | KT390028 | 90.53 | 528 | 100 | d__Eukaryota;k__Alveolata;p__norank;c__Dinophyceae;o__Blastodiniales; f__Blastodiniales;g__Blastodinium-like;s__Blastodinium-like_sp. |
| OTU1407 | 528 | KT390028 | 89.58 | 528 | 100 | d__Eukaryota;k__Alveolata;p__norank;c__Dinophyceae;o__Blastodiniales; f__Blastodiniales;g__Blastodinium-like;s__Blastodinium-like_sp. |
| OTU1409 | 501 | KT390028 | 91.82 | 501 | 100 | d__Eukaryota;k__Alveolata;p__norank;c__Dinophyceae;o__Blastodiniales; f__Blastodiniales;g__Blastodinium-like;s__Blastodinium-like_sp. |
| OTU1410 | 531 | KT390028 | 86.96 | 537 | 100 | d__Eukaryota;k__Alveolata;p__norank;c__Dinophyceae;o__Blastodiniales; f__Blastodiniales;g__Blastodinium-like;s__Blastodinium-like_sp. |
| OTU1412 | 518 | KT390028 | 84.45 | 521 | 100 | d__Eukaryota;k__Alveolata;p__norank;c__Dinophyceae;o__Blastodiniales; f__Blastodiniales;g__Blastodinium-like;s__Blastodinium-like_sp. |
| OTU1413 | 529 | KT390028 | 88.72 | 532 | 100 | d__Eukaryota;k__Alveolata;p__norank;c__Dinophyceae;o__Blastodiniales; f__Blastodiniales;g__Blastodinium-like;s__Blastodinium-like_sp. |
| OTU1414 | 521 | KT390028 | 94.63 | 521 | 100 | d__Eukaryota;k__Alveolata;p__norank;c__Dinophyceae;o__Blastodiniales; f__Blastodiniales;g__Blastodinium-like;s__Blastodinium-like_sp. |
| OTU1415 | 531 | KT390028 | 90.4 | 531 | 100 | d__Eukaryota;k__Alveolata;p__norank;c__Dinophyceae;o__Blastodiniales; f__Blastodiniales;g__Blastodinium-like;s__Blastodinium-like_sp. |
| OTU1417 | 526 | KT390028 | 86.09 | 532 | 100 | d__Eukaryota;k__Alveolata;p__norank;c__Dinophyceae;o__Blastodiniales; f__Blastodiniales;g__Blastodinium-like;s__Blastodinium-like_sp. |
| OTU1436 | 478 | EF616465.1 | 87 | 478 | 100 | d__Eukaryota;k__Alveolata;p__norank;c__Dinophyceae;o__Gymnodiniales;f_Gy mnodiniaceae;g__Gymnodinium;s__Gymnodinium_impudicum-like |
| OTU1437 | 486 | FR720082.1 | 95 | 488 | 100 | d__Eukaryota;k__Alveolata;p__norank;c__Dinophyceae;o__Gymnodiniales;f_Gy mnodiniaceae;g__Gyrodiniellum;s__Gyrodiniellum_shiwhaense |
| OTU1438 | 483 | FR720082.1 | 94 | 483 | 100 | d_Eukaryota;k_Alveolata;p_norank;c_Dinophyceae;o_Gymnodiniales;f_Gymnodi niaceae;g_Gyrodiniellum;s_Gyrodiniellum_shiwhaense |
| OTU1439 | 489 | FR720082.1 | 95 | 483 | 100 | d_Eukaryota;k_Alveolata;p_norank;c_Dinophyceae;o_Gymnodiniales;f_Gymnodi niaceae;g_Gyrodiniellum;s_Gyrodiniellum_shiwhaense |
| OTU1440 | 488 | FR720082.1 | 92.21 | 488 | 100 | d_Eukaryota;k_Alveolata;p_norank;c_Dinophyceae;o_Gymnodiniales;f_Gymnodi niaceae;g_Gyrodiniellum;s_Gyrodiniellum_shiwhaense |
| OTU1448 | 530 | FJ160592 | 90.12 | 81 | 33 | d__Eukaryota;k__Alveolata;p__norank;c__Dinophyceae;o__norank;f__norank;g_ _norank;s__Unclassified DINO |
| OTU1449 | 536 | FJ160592 | 90.12 | 81 | 33 | d__Eukaryota;k__Alveolata;p__norank;c__Dinophyceae;o__norank;f__norank;g_ _norank;s__Unclassified DINO |
| OTU1450 | 535 | FJ160592 | 90.12 | 81 | 33 | d__Eukaryota;k__Alveolata;p__norank;c__Dinophyceae;o__norank;f__norank;g_ _norank;s__Unclassified DINO |
| OTU1451 | 552 | FJ160592 | 90.12 | 81 | 32 | d__Eukaryota;k__Alveolata;p__norank;c__Dinophyceae;o__norank;f__norank;g_ _norank;s__Unclassified DINO |
| OTU1453 | 548 | FJ160592 | 90.12 | 81 | 32 | d__Eukaryota;k__Alveolata;p__norank;c__Dinophyceae;o__norank;f__norank;g_ _norank;s__Unclassified DINO |

| | | | | | | |
|---|---|---|---|---|---|---|
| OTU1454 | 562 | FJ160592 | 90.12 | 81 | 31 | d__Eukaryota;k__Alveolata;p__norank;c__Dinophyceae;o__norank;f__norank;g__norank;s__Unclassified DINO |
| OTU1455 | 548 | FJ160592 | 90.12 | 81 | 32 | d__Eukaryota;k__Alveolata;p__norank;c__Dinophyceae;o__norank;f__norank;g__norank;s__Unclassified DINO |
| OTU1456 | 540 | FJ160592 | 90.12 | 81 | 33 | d__Eukaryota;k__Alveolata;p__norank;c__Dinophyceae;o__norank;f__norank;g__norank;s__Unclassified DINO |
| OTU1457 | 505 | FJ167681 | 97.43 | 505 | 100 | d__Eukaryota;k__Alveolata;p__norank;c__Dinophyceae;o__Peridiniales;f__Pfiesteriaceae;g__Tyrannodinium;s__Tyrannodinium_berolinense |
| OTU1459 | 505 | FJ167681 | 96.63 | 505 | 100 | d__Eukaryota;k__Alveolata;p__norank;c__Dinophyceae;o__Peridiniales;f__Pfiesteriaceae;g__Tyrannodinium;s__Tyrannodinium_berolinense |
| OTU1466 | 457 | JQ616825 | 72 | 250 | 56 | d__Eukaryota;k__Alveolata;p__norank;c__Dinophyceae;o__Gymnodiniales;f__Gymnodiniaceae;g__Gymnodinium-like;s__Gymnodinium-like_sp. |
| OTU1494 | 490 | FJ211386 | 98.98 | 490 | 100 | d__Eukaryota;k__Alveolata;p__norank;c__Dinophyceae;o__Gymnodiniales;f__Polykrikaceae;g__Polykrikos;s__Polykrikos_geminatum |
| OTU1495 | 503 | KT389895.1 | 99 | 503 | 100 | d__Eukaryota;k__Alveolata;p__norank;c__Dinophyceae;o__Pyrocystales;f__Pyrocystaceae;g__Dissodinium;s__Dissodinium_pseudolunula |
| OTU1498 | 507 | FJ236464 | 100 | 502 | 99 | d__Eukaryota;k__Alveolata;p__norank;c__Dinophyceae;o__Peridiniales;f__Glenodiniaceae;g__Peridiniopsis;s__Peridiniopsis_borgei |
| OTU1511 | 489 | EU707473 | 92.06 | 491 | 100 | d__Eukaryota;k__Alveolata;p__norank;c__Dinophyceae;o__Gonyaulacales;f__Gonyaulacaceae;g__Alexandrium;s__Alexandrium_minutum |
| OTU1520 | 484 | FJ939579 | 72.27 | 494 | 99 | d__Eukaryota;k__Alveolata;p__norank;c__Dinophyceae;o__Gonyaulacales;f__Gonyaulacaceae;g__Gonyaulax;s__Gonyaulax_sp. |
| OTU1550 | 332 | KJ481814 | 85 | 200 | 60 | d__Eukaryota;k__Alveolata;p__norank;c__Dinophyceae;s__Unclaassified DINO |
| OTU1570 | 500 | KP790240 | 98.33 | 419 | 84 | d__Eukaryota;k__Alveolata;p__norank;c__Dinophyceae;o__Gymnodiniales;f__Warnowiaceae;g__Warnowia;s__Warnowia_sp._4_AR-2015 |
| OTU1571 | 497 | FJ947041 | 89.56 | 498 | 100 | d__Eukaryota;k__Alveolata;p__norank;c__Dinophyceae;o__Gymnodiniales;f__Warnowiaceae;g__Nematodinium;s__Nematodinium_sp._BSL-2009a |
| OTU1572 | 492 | FJ947041 | 93.71 | 493 | 100 | d__Eukaryota;k__Alveolata;p__norank;c__Dinophyceae;o__Gymnodiniales;f__Warnowiaceae;g__Nematodinium;s__Nematodinium_sp._BSL-2009a |
| OTU1573 | 407 | FJ947041 | 88.24 | 408 | 100 | d__Eukaryota;k__Alveolata;p__norank;c__Dinophyceae;o__Gymnodiniales;f__Warnowiaceae;g__Nematodinium;s__Nematodinium_sp._BSL-2009a |
| OTU1574 | 353 | FJ947041 | 90.67 | 343 | 97 | d__Eukaryota;k__Alveolata;p__norank;c__Dinophyceae;o__Gymnodiniales;f__Warnowiaceae;g__Nematodinium;s__Nematodinium_sp._BSL-2009a |
| OTU1576 | 498 | FJ947042 | 89.68 | 504 | 100 | d__Eukaryota;k__Alveolata;p__norank;c__Dinophyceae;o__Gymnodiniales;f__Warnowiaceae;g__Warnowia;s__Warnowia_sp._BSL-2009a |
| OTU1577 | 418 | KP790242 | 98.8 | 418 | 100 | d__Eukaryota;k__Alveolata;p__norank;c__Dinophyceae;o__Gymnodiniales;f__Warnowiaceae;g__Warnowia;s__Warnowia_sp._5_AR-2015 |
| OTU1588 | 297 | AB473665 | 97.31 | 297 | 100 | d__Eukaryota;k__Alveolata;p__norank;c__Dinophyceae;o__Dinophysiales;f__Dinophysiaceae;g__Dinophysis;s__Dinophysis_lativelata |
| OTU1593 | 490 | JQ247713 | 73.1 | 513 | 100 | d__Eukaryota;k__Alveolata;p__norank;c__Dinophyceae;o__Peridiniales;f__Heter |

| | | | | | | |
|---|---|---|---|---|---|---|
| | | | | | | ocapsaceae;g__Heterocapsa;s__Heterocapsa_sp. |
| OTU1594 | 463 | FN357291 | 72.73 | 484 | 99 | d__Eukaryota;k__Alveolata;p__norank;c__Dinophyceae;o__Gymnodiniales;f__Kareniaceae;g__Karlodinium;s__Karlodinium_sp._KAMS0708 |
| OTU1599 | 506 | FJ600087 | 93.48 | 506 | 100 | d__Eukaryota;k__Alveolata;p__norank;c__Dinophyceae;o__Peridiniales;f__Pfiesteriaceae;g__Pfiesteria;s__Pfiesteria_piscicida |
| OTU1607 | 503 | FJ600087 | 88.66 | 529 | 100 | d__Eukaryota;k__Alveolata;p__norank;c__Dinophyceae;o__Peridiniales;f__Pfiesteriaceae;g__Pfiesteria;s__Pfiesteria_piscicida |
| OTU1612 | 478 | KF751927.1 | 70 | 478 | 99 | d__Eukaryota;k__Alveolata;p__norank;c__Dinophyceae;o__Peridiniales;f__Thoracosphaeraceae;g__Scrippsiella;s__Scrippsiella_sp. |
| OTU1613 | 461 | EF205003 | 83.72 | 86 | 53 | d__Eukaryota;k__Alveolata;p__norank;c__Dinophyceae;o__norank;f__norank;g__norank;s__Unclassified DINO |
| OTU1620 | 334 | LC027066 | 82.08 | 106 | 32 | d__Eukaryota;k__Alveolata;p__norank;c__Dinophyceae;o__norank;f__norank;g__norank;s__Unclassified DINO |
| OTU1622 | 447 | KJ189480 | 83.5 | 103 | 23 | d__Eukaryota;k__Alveolata;p__norank;c__Dinophyceae;o__Peridiniales;f__Thoracosphaeraceae;g__Scrippsiella-like;s__Scrippsiella-like_sp. |
| OTU1625 | 466 | EU126801.1 | 70 | 466 | 100 | d__Eukaryota;k__Alveolata;p__norank;c__Dinophyceae;o__Suessiales; ;f__Borghiellaceae;g__Borghiella;s__Borghiella__sp. |
| OTU1626 | 506 | HQ902267 | 80 | 141 | 90 | d__Eukaryota;k__Alveolata;p__norank;c__Dinophyceae;o__Peridiniales;f__Heterocapsaceae;g__Heterocapsa;s__Heterocapsa_sp. |
| OTU1629 | 458 | KJ433986 | 72.79 | 463 | 99 | d__Eukaryota;k__Alveolata;p__norank;c__Dinophyceae;o__Peridiniales;f__Peridiniaceae;g__Pentapharsodinium;s__Pentapharsodinium_sp._ZL-2014 |
| OTU1630 | 496 | LC068842 | 81 | 549 | 100 | d__Eukaryota;k__Alveolata;p__norank;c__Dinophyceae;o__Lophodiniales;f__Lophodiniaceae;g__Biecheleria-like;s__Biecheleria-like_sp. |
| OTU1636 | 494 | FN557541 | 96.76 | 494 | 100 | d__Eukaryota;k__Alveolata;p__norank;c__Dinophyceae;o__unclassified_Dinophyceae;f__norank;g__Stoeckeria;s__Stoeckeria_sp._SSMS0806 |
| OTU1639 | 504 | FN557541 | 99.8 | 504 | 100 | d__Eukaryota;k__Alveolata;p__norank;c__Dinophyceae;o__unclassified_Dinophyceae;f__norank;g__Stoeckeria;s__Stoeckeria_sp._SSMS0806 |
| OTU1640 | 485 | LK934662 | 92.26 | 478 | 98 | d__Eukaryota;k__Alveolata;p__norank;c__Dinophyceae;o__Peridiniales;f__Pfiesteriaceae;g__Aduncodinium;s__Aduncodinium_glandula |
| OTU1643 | 498 | FN557541 | 96.79 | 498 | 100 | d__Eukaryota;k__Alveolata;p__norank;c__Dinophyceae;o__unclassified_Dinophyceae;f__norank;g__Stoeckeria;s__Stoeckeria_sp._SSMS0806 |
| OTU1645 | 489 | FN557541 | 93.05 | 489 | 100 | d__Eukaryota;k__Alveolata;p__norank;c__Dinophyceae;o__unclassified_Dinophyceae;f__norank;g__Stoeckeria;s__Stoeckeria_sp._SSMS0806 |
| OTU1676 | 525 | HE611580 | 94.32 | 88 | 55 | d__Eukaryota;k__Alveolata;p__norank;c__Dinophyceae;o__Gymnodiniales;f__Gymnodiniaceae;g__Gyrodinium;s__Gyrodinium_sp._HJ-2011 |
| OTU1695 | 507 | LC002848 | 82.09 | 402 | 79 | d__Eukaryota;k__Alveolata;p__norank;c__Dinophyceae;o__unclassified_Dinophyceae;f__norank;g__norank;s__Unclassified DINO |
| OTU1775 | 504 | HM483396 | 96.83 | 504 | 100 | d__Eukaryota;k__Alveolata;p__norank;c__Dinophyceae;o__Peridiniales;f__Thoracosphaeraceae;g__Scrippsiella;s__Scrippsiella_trochoidea |
| OTU1776 | 482 | HM483396 | 92.95 | 482 | 100 | d__Eukaryota;k__Alveolata;p__norank;c__Dinophyceae;o__Peridiniales;f__Thora |

| | | | | | | |
|---|---|---|---|---|---|---|
| | | | | | | cosphaeraceae;g__Scrippsiella;s__Scrippsiella_trochoidea |
| OTU1777 | 506 | HM483396 | 96.83 | 505 | 99 | d__Eukaryota;k__Alveolata;p__norank;c__Dinophyceae;o__Peridiniales;f__Thoracosphaeraceae;g__Scrippsiella;s__Scrippsiella_trochoidea |
| OTU1778 | 494 | HM483396 | 95.75 | 494 | 100 | d__Eukaryota;k__Alveolata;p__norank;c__Dinophyceae;o__Peridiniales;f__Thoracosphaeraceae;g__Scrippsiella;s__Scrippsiella_trochoidea |
| OTU1779 | 503 | HM483396 | 97.01 | 501 | 99 | d__Eukaryota;k__Alveolata;p__norank;c__Dinophyceae;o__Peridiniales;f__Thoracosphaeraceae;g__Scrippsiella;s__Scrippsiella_trochoidea |
| OTU1780 | 499 | HM483396 | 94.39 | 499 | 100 | d__Eukaryota;k__Alveolata;p__norank;c__Dinophyceae;o__Peridiniales;f__Thoracosphaeraceae;g__Scrippsiella;s__Scrippsiella_trochoidea |
| OTU1781 | 499 | HM483396 | 93.79 | 499 | 100 | d__Eukaryota;k__Alveolata;p__norank;c__Dinophyceae;o__Peridiniales;f__Thoracosphaeraceae;g__Scrippsiella;s__Scrippsiella_trochoidea |
| OTU1782 | 505 | HM483396 | 97.82 | 505 | 100 | d__Eukaryota;k__Alveolata;p__norank;c__Dinophyceae;o__Peridiniales;f__Thoracosphaeraceae;g__Scrippsiella;s__Scrippsiella_trochoidea |
| OTU1783 | 526 | KF751926 | 85.33 | 525 | 99 | d__Eukaryota;k__Alveolata;p__norank;c__Dinophyceae;o__Peridiniales;f__Thoracosphaeraceae;g__Scrippsiella;s__Scrippsiella_sp. |
| OTU1785 | 527 | HM483396 | 95.98 | 522 | 99 | d__Eukaryota;k__Alveolata;p__norank;c__Dinophyceae;o__Peridiniales;f__Thoracosphaeraceae;g__Scrippsiella;s__Scrippsiella_trochoidea |
| OTU1788 | 505 | HM483396 | 96.44 | 505 | 100 | d__Eukaryota;k__Alveolata;p__norank;c__Dinophyceae;o__Peridiniales;f__Thoracosphaeraceae;g__Scrippsiella;s__Scrippsiella_trochoidea |
| OTU1789 | 513 | HM483396 | 95.13 | 513 | 100 | d__Eukaryota;k__Alveolata;p__norank;c__Dinophyceae;o__Peridiniales;f__Thoracosphaeraceae;g__Scrippsiella;s__Scrippsiella_trochoidea |
| OTU1790 | 511 | HM483396 | 86.19 | 507 | 98 | d__Eukaryota;k__Alveolata;p__norank;c__Dinophyceae;o__Peridiniales;f__Thoracosphaeraceae;g__Scrippsiella;s__Scrippsiella_trochoidea |
| OTU1792 | 503 | HM483396 | 96.21 | 501 | 99 | d__Eukaryota;k__Alveolata;p__norank;c__Dinophyceae;o__Peridiniales;f__Thoracosphaeraceae;g__Scrippsiella;s__Scrippsiella_trochoidea |
| OTU1794 | 499 | HM483396 | 96.79 | 498 | 99 | d__Eukaryota;k__Alveolata;p__norank;c__Dinophyceae;o__Peridiniales;f__Thoracosphaeraceae;g__Scrippsiella;s__Scrippsiella_trochoidea |
| OTU1795 | 489 | HM483396 | 96.52 | 489 | 100 | d__Eukaryota;k__Alveolata;p__norank;c__Dinophyceae;o__Peridiniales;f__Thoracosphaeraceae;g__Scrippsiella;s__Scrippsiella_trochoidea |
| OTU1796 | 505 | HM483396 | 95.84 | 505 | 100 | d__Eukaryota;k__Alveolata;p__norank;c__Dinophyceae;o__Peridiniales;f__Thoracosphaeraceae;g__Scrippsiella;s__Scrippsiella_trochoidea |
| OTU1799 | 503 | HM483396 | 89.66 | 503 | 100 | d__Eukaryota;k__Alveolata;p__norank;c__Dinophyceae;o__Peridiniales;f__Thoracosphaeraceae;g__Scrippsiella;s__Scrippsiella_trochoidea |
| OTU1800 | 504 | HM483396 | 96.63 | 504 | 100 | d__Eukaryota;k__Alveolata;p__norank;c__Dinophyceae;o__Peridiniales;f__Thoracosphaeraceae;g__Scrippsiella;s__Scrippsiella_trochoidea |
| OTU1804 | 496 | HM483396 | 94.02 | 502 | 100 | d__Eukaryota;k__Alveolata;p__norank;c__Dinophyceae;o__Peridiniales;f__Thoracosphaeraceae;g__Scrippsiella;s__Scrippsiella_trochoidea |
| OTU1807 | 496 | HM483396 | 93.15 | 496 | 100 | d__Eukaryota;k__Alveolata;p__norank;c__Dinophyceae;o__Peridiniales;f__Thoracosphaeraceae;g__Scrippsiella;s__Scrippsiella_trochoidea |
| OTU1811 | 527 | HM483396 | 88.95 | 525 | 99 | d__Eukaryota;k__Alveolata;p__norank;c__Dinophyceae;o__Peridiniales;f__Thora |

| | | | | | | |
|---|---|---|---|---|---|---|
| | | | | | | cosphaeraceae;g__Scrippsiella;s__Scrippsiella_trochoidea |
| OTU1821 | 493 | HM483396 | 94.64 | 504 | 100 | d__Eukaryota;k__Alveolata;p__norank;c__Dinophyceae;o__Peridiniales;f__Thoracosphaeraceae;g__Scrippsiella;s__Scrippsiella_trochoidea |
| OTU1822 | 497 | HM483396 | 93.37 | 498 | 100 | d__Eukaryota;k__Alveolata;p__norank;c__Dinophyceae;o__Peridiniales;f__Thoracosphaeraceae;g__Scrippsiella;s__Scrippsiella_trochoidea |
| OTU1824 | 492 | HM483396 | 88.96 | 498 | 100 | d__Eukaryota;k__Alveolata;p__norank;c__Dinophyceae;o__Peridiniales;f__Thoracosphaeraceae;g__Scrippsiella;s__Scrippsiella_trochoidea |
| OTU1826 | 493 | HM483396 | 92.12 | 520 | 100 | d__Eukaryota;k__Alveolata;p__norank;c__Dinophyceae;o__Peridiniales;f__Thoracosphaeraceae;g__Scrippsiella;s__Scrippsiella_trochoidea |
| OTU1828 | 490 | HM483396 | 91.8 | 488 | 99 | d__Eukaryota;k__Alveolata;p__norank;c__Dinophyceae;o__Peridiniales;f__Thoracosphaeraceae;g__Scrippsiella;s__Scrippsiella_trochoidea |
| OTU1832 | 369 | HM483396 | 92.14 | 369 | 100 | d__Eukaryota;k__Alveolata;p__norank;c__Dinophyceae;o__Peridiniales;f__Thoracosphaeraceae;g__Scrippsiella;s__Scrippsiella_trochoidea |
| OTU1834 | 377 | HM483396 | 91.8 | 378 | 100 | d__Eukaryota;k__Alveolata;p__norank;c__Dinophyceae;o__Peridiniales;f__Thoracosphaeraceae;g__Scrippsiella;s__Scrippsiella_trochoidea |
| OTU1836 | 508 | HM483396 | 88.04 | 510 | 100 | d__Eukaryota;k__Alveolata;p__norank;c__Dinophyceae;o__Peridiniales;f__Thoracosphaeraceae;g__Scrippsiella;s__Scrippsiella_trochoidea |
| OTU1842 | 516 | HM483396 | 94.79 | 518 | 100 | d__Eukaryota;k__Alveolata;p__norank;c__Dinophyceae;o__Peridiniales;f__Thoracosphaeraceae;g__Scrippsiella;s__Scrippsiella_trochoidea |
| OTU1848 | 500 | HM483396 | 94.19 | 516 | 100 | d__Eukaryota;k__Alveolata;p__norank;c__Dinophyceae;o__Peridiniales;f__Thoracosphaeraceae;g__Scrippsiella;s__Scrippsiella_trochoidea |
| OTU185 | 518 | AY245689 | 97.68 | 517 | 99 | d__Eukaryota;k__Alveolata;p__norank;c__Dinophyceae;o__Peridiniales;f__Pfiesteriaceae;g__unclassified_Pfiesteriaceae;s__Pfiesteria-like_DINO_Lucy |
| OTU1850 | 486 | HM483396 | 91.56 | 486 | 100 | d__Eukaryota;k__Alveolata;p__norank;c__Dinophyceae;o__Peridiniales;f__Thoracosphaeraceae;g__Scrippsiella;s__Scrippsiella_trochoidea |
| OTU1853 | 494 | EF152961 | 91.5 | 494 | 100 | d__Eukaryota;k__Alveolata;p__norank;c__Dinophyceae;o__Peridiniales;f__Diplopsaliaceae;g__Preperidinium;s__Protoperidinium_cf._steidingerae |
| OTU1855 | 506 | HM483396 | 93.74 | 527 | 100 | d__Eukaryota;k__Alveolata;p__norank;c__Dinophyceae;o__Peridiniales;f__Thoracosphaeraceae;g__Scrippsiella;s__Scrippsiella_trochoidea |
| OTU186 | 519 | AY245689 | 94.8 | 519 | 100 | d__Eukaryota;k__Alveolata;p__norank;c__Dinophyceae;o__Peridiniales;f__Pfiesteriaceae;g__unclassified_Pfiesteriaceae;s__Pfiesteria-like_DINO_Lucy |
| OTU1864 | 476 | HM483396 | 94.76 | 477 | 100 | d__Eukaryota;k__Alveolata;p__norank;c__Dinophyceae;o__Peridiniales;f__Thoracosphaeraceae;g__Scrippsiella;s__Scrippsiella_trochoidea |
| OTU1866 | 358 | HM483396 | 92.46 | 358 | 100 | d__Eukaryota;k__Alveolata;p__norank;c__Dinophyceae;o__Peridiniales;f__Thoracosphaeraceae;g__Scrippsiella;s__Scrippsiella_trochoidea |
| OTU1867 | 489 | HM483396 | 95.71 | 489 | 100 | d__Eukaryota;k__Alveolata;p__norank;c__Dinophyceae;o__Peridiniales;f__Thoracosphaeraceae;g__Scrippsiella;s__Scrippsiella_trochoidea |
| OTU1870 | 502 | HM483396 | 95 | 500 | 99 | d__Eukaryota;k__Alveolata;p__norank;c__Dinophyceae;o__Peridiniales;f__Thoracosphaeraceae;g__Scrippsiella;s__Scrippsiella_trochoidea |
| OTU1871 | 362 | HM483396 | 96.97 | 363 | 100 | d__Eukaryota;k__Alveolata;p__norank;c__Dinophyceae;o__Peridiniales;f__Thora |

| | | | | | | |
|---|---|---|---|---|---|---|
| | | | | | | cosphaeraceae;g__Scrippsiella;s__Scrippsiella_trochoidea |
| OTU1876 | 459 | HM483396 | 95.21 | 459 | 100 | d__Eukaryota;k__Alveolata;p__norank;c__Dinophyceae;o__Peridiniales;f__Thoracosphaeraceae;g__Scrippsiella;s__Scrippsiella_trochoidea |
| OTU1879 | 436 | HM483396 | 89.33 | 431 | 99 | d__Eukaryota;k__Alveolata;p__norank;c__Dinophyceae;o__Peridiniales;f__Thoracosphaeraceae;g__Scrippsiella;s__Scrippsiella_trochoidea |
| OTU1884 | 489 | HM483396 | 92.02 | 489 | 100 | d__Eukaryota;k__Alveolata;p__norank;c__Dinophyceae;o__Peridiniales;f__Thoracosphaeraceae;g__Scrippsiella;s__Scrippsiella_trochoidea |
| OTU1886 | 490 | HM483396 | 94.67 | 488 | 99 | d__Eukaryota;k__Alveolata;p__norank;c__Dinophyceae;o__Peridiniales;f__Thoracosphaeraceae;g__Scrippsiella;s__Scrippsiella_trochoidea |
| OTU1887 | 475 | HM483396 | 97.05 | 475 | 100 | d__Eukaryota;k__Alveolata;p__norank;c__Dinophyceae;o__Peridiniales;f__Thoracosphaeraceae;g__Scrippsiella;s__Scrippsiella_trochoidea |
| OTU1892 | 511 | HM483396 | 96.69 | 513 | 100 | d__Eukaryota;k__Alveolata;p__norank;c__Dinophyceae;o__Peridiniales;f__Thoracosphaeraceae;g__Scrippsiella;s__Scrippsiella_trochoidea |
| OTU1897 | 498 | HM483396 | 90.56 | 498 | 100 | d__Eukaryota;k__Alveolata;p__norank;c__Dinophyceae;o__Peridiniales;f__Thoracosphaeraceae;g__Scrippsiella;s__Scrippsiella_trochoidea |
| OTU1898 | 480 | HM483396 | 92.97 | 498 | 100 | d__Eukaryota;k__Alveolata;p__norank;c__Dinophyceae;o__Peridiniales;f__Thoracosphaeraceae;g__Scrippsiella;s__Scrippsiella_trochoidea |
| OTU1906 | 395 | HM483396 | 96.23 | 398 | 100 | d__Eukaryota;k__Alveolata;p__norank;c__Dinophyceae;o__Peridiniales;f__Thoracosphaeraceae;g__Scrippsiella;s__Scrippsiella_trochoidea |
| OTU1907 | 500 | HM483396 | 92.83 | 516 | 100 | d__Eukaryota;k__Alveolata;p__norank;c__Dinophyceae;o__Peridiniales;f__Thoracosphaeraceae;g__Scrippsiella;s__Scrippsiella_trochoidea |
| OTU1910 | 505 | HM483396 | 89.5 | 505 | 100 | d__Eukaryota;k__Alveolata;p__norank;c__Dinophyceae;o__Peridiniales;f__Thoracosphaeraceae;g__Scrippsiella;s__Scrippsiella_trochoidea |
| OTU1911 | 507 | KP702719 | 92.31 | 507 | 100 | d__Eukaryota;k__Alveolata;p__norank;c__Dinophyceae;o__Peridiniales;f__Diplopsaliaceae;g__Oblea;s__Oblea_rotunda |
| OTU1912 | 507 | HM483396 | 95.87 | 508 | 100 | d__Eukaryota;k__Alveolata;p__norank;c__Dinophyceae;o__Peridiniales;f__Thoracosphaeraceae;g__Scrippsiella;s__Scrippsiella_trochoidea |
| OTU1913 | 495 | AF260393 | 94.55 | 495 | 100 | d__Eukaryota;k__Alveolata;p__norank;c__Dinophyceae;o__Peridiniales;f__Thoracosphaeraceae;g__Scrippsiella;s__Scrippsiella_trochoidea |
| OTU1915 | 280 | HM483396 | 95.68 | 278 | 99 | d__Eukaryota;k__Alveolata;p__norank;c__Dinophyceae;o__Peridiniales;f__Thoracosphaeraceae;g__Scrippsiella;s__Scrippsiella_trochoidea |
| OTU1917 | 496 | HM483396 | 89.31 | 496 | 100 | d__Eukaryota;k__Alveolata;p__norank;c__Dinophyceae;o__Peridiniales;f__Thoracosphaeraceae;g__Scrippsiella;s__Scrippsiella_trochoidea |
| OTU192 | 499 | AY245689 | 94.59 | 499 | 100 | d__Eukaryota;k__Alveolata;p__norank;c__Dinophyceae;o__Peridiniales;f__Pfiesteriaceae;g__unclassified_Pfiesteriaceae;s__Pfiesteria-like_DINO_Lucy |
| OTU1920 | 489 | HM483399 | 89.82 | 491 | 100 | d__Eukaryota;k__Alveolata;p__norank;c__Dinophyceae;o__unclassified_Dinophyceae;f__norank;g__Duboscquodinium;s__Duboscquodinium_collinii |
| OTU1921 | 509 | HM483399 | 95.86 | 507 | 99 | d__Eukaryota;k__Alveolata;p__norank;c__Dinophyceae;o__unclassified_Dinophyceae;f__norank;g__Duboscquodinium;s__Duboscquodinium_collinii |
| OTU1925 | 492 | HM483399 | 88.66 | 494 | 100 | d__Eukaryota;k__Alveolata;p__norank;c__Dinophyceae;o__unclassified_Dinoph |

| | | | | | | |
|---|---|---|---|---|---|---|
| | | | | | | yceae;f__norank;g__Duboscquodinium;s__Duboscquodinium_collinii |
| OTU1926 | 502 | HM483399 | 92.87 | 505 | 100 | d__Eukaryota;k__Alveolata;p__norank;c__Dinophyceae;o__unclassified_Dinophyceae;f__norank;g__Duboscquodinium;s__Duboscquodinium_collinii |
| OTU1928 | 502 | HM483399 | 92.54 | 496 | 99 | d__Eukaryota;k__Alveolata;p__norank;c__Dinophyceae;o__unclassified_Dinophyceae;f__norank;g__Duboscquodinium;s__Duboscquodinium_collinii |
| OTU1931 | 327 | HM483399 | 83.17 | 315 | 96 | d__Eukaryota;k__Alveolata;p__norank;c__Dinophyceae;o__unclassified_Dinophyceae;f__norank;g__Duboscquodinium;s__Duboscquodinium_collinii |
| OTU1932 | 297 | HM483399 | 86.38 | 279 | 94 | d__Eukaryota;k__Alveolata;p__norank;c__Dinophyceae;o__unclassified_Dinophyceae;f__norank;g__Duboscquodinium;s__Duboscquodinium_collinii |
| OTU199 | 525 | AY245689 | 96.88 | 32 | 81 | d__Eukaryota;k__Alveolata;p__norank;c__Dinophyceae;o__Peridiniales;f__Pfiesteriaceae;g__unclassified_Pfiesteriaceae;s__Pfiesteria-like_DINO_Lucy |
| OTU1996 | 482 | EF205002 | 85.9 | 78 | 38 | d__Eukaryota;k__Alveolata;p__norank;c__Dinophyceae;o__norank;f__norank;g__norank;s__Unclassified DINO |
| OTU1997 | 474 | EF205002 | 88.24 | 68 | 37 | d__Eukaryota;k__Alveolata;p__norank;c__Dinophyceae;o__norank;f__norank;g__norank;s__Unclassified DINO |
| OTU1998 | 477 | KR362886 | 89.47 | 57 | 36 | d__Eukaryota;k__Alveolata;p__norank;c__Dinophyceae;o__norank;f__norank;g__norank;s__Unclassified DINO |
| OTU2000 | 494 | FR720082 | 80.72 | 498 | 100 | d__Eukaryota;k__Alveolata;p__norank;c__Dinophyceae;o__Gymnodiniales;f__Gymnodiniaceae;g__Gyrodiniellum;s__Gyrodiniellum_sp. |
| OTU2002 | 493 | FR720082 | 82.29 | 497 | 100 | d__Eukaryota;k__Alveolata;p__norank;c__Dinophyceae;o__Gymnodiniales;f__Gymnodiniaceae;g__Gyrodiniellum;s__Gyrodiniellum_sp. |
| OTU2003 | 499 | AM408889 | 81.37 | 510 | 100 | d__Eukaryota;k__Alveolata;p__norank;c__Dinophyceae;o__Gymnodiniales;f__Gymnodiniaceae;g__Paragymnodinium;s__Paragymnodinium_sp. |
| OTU2008 | 333 | EF205002 | 92.59 | 108 | 32 | d__Eukaryota;k__Alveolata;p__norank;c__Dinophyceae;o__norank;f__norank;g__norank;s__Unclassified DINO |
| OTU201 | 227 | LK934662 | 91.15 | 226 | 99 | d__Eukaryota;k__Alveolata;p__norank;c__Dinophyceae;o__Peridiniales;f__Pfiesteriaceae;g__Aduncodinium;s__Aduncodinium_glandula |
| OTU2013 | 221 | EF205002 | 92.59 | 108 | 49 | d__Eukaryota;k__Alveolata;p__norank;c__Dinophyceae;o__Peridiniales;f__Glenodiniaceae;g__Glenodiniopsis;s__Glenodiniopsis_sp. |
| OTU2014 | 296 | EF205002 | 92.59 | 108 | 36 | d__Eukaryota;k__Alveolata;p__norank;c__Dinophyceae;o__Peridiniales;f__Glenodiniaceae;g__Glenodiniopsis;s__Glenodiniopsis_sp. |
| OTU2023 | 532 | JX262498 | 77.01 | 548 | 100 | d__Eukaryota;k__Alveolata;p__norank;c__Dinophyceae;o__Peridiniales;f__Peridiniaceae;g__Pentapharsodinium;s__Pentapharsodinium_sp. |
| OTU2024 | 518 | JN119844 | 76.28 | 548 | 99 | d__Eukaryota;k__Alveolata;p__norank;c__Dinophyceae;o__Peridiniales;f__Heterocapsaceae;g__Heterocapsa;s__Heterocapsa_sp._HCBC88 |
| OTU2025 | 504 | AF260399 | 80.35 | 514 | 100 | d__Eukaryota;k__Alveolata;p__norank;c__Dinophyceae;o__Peridiniales;f__Heterocapsaceae;g__Heterocapsa;s__Heterocapsa_sp. |
| OTU2027 | 484 | JX262498 | 91.74 | 484 | 100 | d__Eukaryota;k__Alveolata;p__norank;c__Dinophyceae;o__Peridiniales;f__Peridiniaceae;g__Pentapharsodinium;s__Pentapharsodinium_dalei |
| OTU2028 | 458 | JN119844 | 74 | 500 | 100 | d__Eukaryota;k__Alveolata;p__norank;c__Dinophyceae;o__Peridiniales;f__Heter |

| | | | | | | |
|---|---|---|---|---|---|---|
| | | | | | | ocapsaceae;g__Heterocapsa;s__Heterocapsa_sp._HCBC88 |
| OTU2031 | 282 | JN119844 | 82.17 | 258 | 91 | d__Eukaryota;k__Alveolata;p__norank;c__Dinophyceae;o__Peridiniales;f__Heterocapsaceae;g__Heterocapsa;s__Heterocapsa_sp._HCBC88 |
| OTU2032 | 235 | HQ845329 | 86.94 | 222 | 94 | d__Eukaryota;k__Alveolata;p__norank;c__Dinophyceae;o__Peridiniales;f__Peridiniaceae;g__Pentapharsodinium;s__Pentapharsodinium_tyrrhenicum |
| OTU2033 | 288 | HQ845329 | 96.89 | 289 | 100 | d__Eukaryota;k__Alveolata;p__norank;c__Dinophyceae;o__Peridiniales;f__Peridiniaceae;g__Pentapharsodinium;s__Pentapharsodinium_tyrrhenicum |
| OTU2034 | 364 | HQ845328 | 79.94 | 359 | 99 | d__Eukaryota;k__Alveolata;p__norank;c__Dinophyceae;o__Peridiniales;f__Thoracosphaeraceae;g__Ensiculifera;s__Ensiculifera_aff._loeblichii |
| OTU2036 | 495 | KF751926 | 89.05 | 539 | 100 | d__Eukaryota;k__Alveolata;p__norank;c__Dinophyceae;o__Peridiniales;f__Thoracosphaeraceae;g__Scrippsiella;s__Scrippsiella_sp. |
| OTU2037 | 379 | HM483396 | 93.8 | 371 | 98 | d__Eukaryota;k__Alveolata;p__norank;c__Dinophyceae;o__Peridiniales;f__Thoracosphaeraceae;g__Scrippsiella;s__Scrippsiella_trochoidea |
| OTU2038 | 505 | AF260399 | 91.18 | 510 | 100 | d__Eukaryota;k__Alveolata;p__norank;c__Dinophyceae;o__Peridiniales;f__Heterocapsaceae;g__Heterocapsa;s__Heterocapsa_sp. |
| OTU2039 | 496 | HQ902267 | 97.98 | 496 | 100 | d__Eukaryota;k__Alveolata;p__norank;c__Dinophyceae;o__Peridiniales;f__Heterocapsaceae;g__Heterocapsa;s__Heterocapsa_triquetra |
| OTU204 | 502 | KJ450986 | 93.21 | 501 | 99 | d__Eukaryota;k__Alveolata;p__norank;c__Dinophyceae;o__Peridiniales;f__Peridiniaceae;g__Peridinium;s__Peridinium_baicalense |
| OTU2042 | 512 | HQ902267 | 82.91 | 515 | 99 | d__Eukaryota;k__Alveolata;p__norank;c__Dinophyceae;o__Peridiniales;f__Heterocapsaceae;g__Heterocapsa;s__Heterocapsa_triquetra |
| OTU2044 | 502 | HQ902267 | 82.9 | 503 | 100 | d__Eukaryota;k__Alveolata;p__norank;c__Dinophyceae;o__Peridiniales;f__Heterocapsaceae;g__Heterocapsa;s__Heterocapsa_triquetra |
| OTU2046 | 423 | EU165312 | 88.86 | 431 | 100 | d__Eukaryota;k__Alveolata;p__norank;c__Dinophyceae;o__Peridiniales;f__Heterocapsaceae;g__Heterocapsa;s__Heterocapsa_rotundata |
| OTU2050 | 487 | KJ433986 | 75.51 | 490 | 100 | d__Eukaryota;k__Alveolata;p__norank;c__Dinophyceae;o__Peridiniales;f__Peridiniaceae;g__Pentapharsodinium;s__Pentapharsodinium_sp._ZL-2014 |
| OTU2059 | 416 | JF521637 | 98.56 | 417 | 100 | d__Eukaryota;k__Alveolata;p__norank;c__Dinophyceae;o__Gonyaulacales;f__Gonyaulacaceae;g__Alexandrium;s__Alexandrium_ostenfeldii |
| OTU2060 | 466 | JF521638 | 100 | 466 | 100 | d__Eukaryota;k__Alveolata;p__norank;c__Dinophyceae;o__Gonyaulacales;f__Gonyaulacaceae;g__Alexandrium;s__Alexandrium_pseudogoniaulax |
| OTU2061 | 481 | JF521638 | 100 | 398 | 83 | d__Eukaryota;k__Alveolata;p__norank;c__Dinophyceae;o__Gonyaulacales;f__Gonyaulacaceae;g__Alexandrium;s__Alexandrium_pseudogoniaulax |
| OTU2062 | 474 | JF521638 | 90.32 | 475 | 100 | d__Eukaryota;k__Alveolata;p__norank;c__Dinophyceae;o__Gonyaulacales;f__Gonyaulacaceae;g__Alexandrium;s__Alexandrium_pseudogoniaulax |
| OTU2068 | 502 | JF430394 | 92.63 | 502 | 100 | d__Eukaryota;k__Alveolata;p__norank;c__Dinophyceae;o__Peridiniales;f__Pfiesteriaceae;g__Chimonodinium;s__Chimonodinium_lomnickii |
| OTU207 | 488 | KJ670426.1 | 76 | 488 | 100 | d__Eukaryota;k__Alveolata;p__norank;c__Dinophyceae;o__Gymnodiniales;f__Kareniaceae;g__Karlodinium;s__Karlodinium_sp. |
| OTU2078 | 515 | JN119844 | 84.17 | 518 | 100 | d__Eukaryota;k__Alveolata;p__norank;c__Dinophyceae;o__Peridiniales;f__Heter |

| | | | | | | |
|---|---|---|---|---|---|---|
| | | | | | | ocapsaceae;g__Heterocapsa;s__Heterocapsa_sp._HCBC88 |
| OTU2079 | 284 | HM483399 | 85.77 | 281 | 99 | d__Eukaryota;k__Alveolata;p__norank;c__Dinophyceae;o__unclassified_Dinophyceae;f__norank;g__Duboscquodinium;s__Duboscquodinium_collinii |
| OTU2083 | 511 | HQ670228 | 96.08 | 510 | 99 | d__Eukaryota;k__Alveolata;p__norank;c__Dinophyceae;o__Peridiniales;f__Thoracosphaeraceae;g__Scrippsiella;s__Scrippsiella_trochoidea |
| OTU2085 | 498 | HQ670228 | 95.38 | 498 | 100 | d__Eukaryota;k__Alveolata;p__norank;c__Dinophyceae;o__Peridiniales;f__Thoracosphaeraceae;g__Scrippsiella;s__Scrippsiella_trochoidea |
| OTU2093 | 497 | HQ670228 | 94.37 | 497 | 100 | d__Eukaryota;k__Alveolata;p__norank;c__Dinophyceae;o__Peridiniales;f__Thoracosphaeraceae;g__Scrippsiella;s__Scrippsiella_trochoidea |
| OTU21 | 481 | AF260389 | 92.53 | 482 | 100 | d__Eukaryota;k__Alveolata;p__norank;c__Dinophyceae;o__Gonyaulacales;f__Ceratiaceae;g__Neoceratium;s__Neoceratium_tripos |
| OTU210 | 426 | EU165272 | 80.12 | 322 | 75 | d__Eukaryota;k__Alveolata;p__norank;c__Dinophyceae;o__Peridiniales;f__Heterocapsaceae;g__unclassified_Heterocapsaceae;s__Heterocapsaceae-like sp. |
| OTU2109 | 428 | HQ670228 | 96.9 | 420 | 98 | d__Eukaryota;k__Alveolata;p__norank;c__Dinophyceae;o__Peridiniales;f__Thoracosphaeraceae;g__Scrippsiella;s__Scrippsiella_trochoidea |
| OTU2110 | 436 | HQ670228 | 95.86 | 435 | 99 | d__Eukaryota;k__Alveolata;p__norank;c__Dinophyceae;o__Peridiniales;f__Thoracosphaeraceae;g__Scrippsiella;s__Scrippsiella_trochoidea |
| OTU2149 | 474 | AB871538 | 83.67 | 49 | 19 | d__Eukaryota;k__Alveolata;p__norank;c__Dinophyceae;o__norank;f__norank;g__norank;s__Unclassified DINO |
| OTU215 | 465 | AY284951 | 98.71 | 465 | 100 | d__Eukaryota;k__Alveolata;p__norank;c__Dinophyceae;o__Gymnodiniales;f__Kareniaceae;g__Takayama;s__Takayama_helix |
| OTU2173 | 499 | LC027067 | 74 | 82 | 50 | d__Eukaryota;k__Alveolata;p__norank;c__Dinophyceae;o__norank;f__norank;g__norank;s__Unclassified DINO |
| OTU2176 | 513 | KJ508390 | 92.42 | 66 | 35 | d__Eukaryota;k__Alveolata;p__norank;c__Dinophyceae;o__norank;f__norank;g__norank;s__Unclassified DINO |
| OTU2177 | 501 | HQ176320 | 99.6 | 502 | 100 | d__Eukaryota;k__Alveolata;p__norank;c__Dinophyceae;o__Peridiniales;f__Pfiesteriaceae;g__Chimonodinium;s__Chimonodinium_lomnickii |
| OTU2181 | 471 | HQ176320 | 86.86 | 472 | 100 | d__Eukaryota;k__Alveolata;p__norank;c__Dinophyceae;o__Peridiniales;f__Pfiesteriaceae;g__Chimonodinium;s__Chimonodinium_lomnickii |
| OTU2185 | 497 | FN647674 | 96.45 | 422 | 85 | d__Eukaryota;k__Alveolata;p__norank;c__Dinophyceae;o__Gymnodiniales;f__Gymnodiniaceae;g__Barrufeta;s__Barrufeta_bravensis |
| OTU2188 | 494 | FN647674 | 98.1 | 421 | 85 | d__Eukaryota;k__Alveolata;p__norank;c__Dinophyceae;o__Gymnodiniales;f__Gymnodiniaceae;g__Barrufeta;s__Barrufeta_bravensis |
| OTU2190 | 477 | FN649409 | 91.96 | 112 | 23 | d__Eukaryota;k__Alveolata;p__norank;c__Dinophyceae;o__Gymnodiniales;f__Gymnodiniaceae;g__norank;s__Unclassified Gymnodinium-like_species |
| OTU2191 | 478 | AF200673 | 91.96 | 112 | 42 | d__Eukaryota;k__Alveolata;p__norank;c__Dinophyceae;o__Gymnodiniales;f__Gymnodiniaceae;g__norank;s__Unclassified Gymnodinium-like_species |
| OTU2196 | 479 | DQ195375 | 75.21 | 480 | 99 | d__Eukaryota;k__Alveolata;p__norank;c__Dinophyceae;o__Suessiales;f__Suessiaceae;g__Pelagodinium;s__Pelagodinium_sp. |
| OTU2197 | 479 | JN558107 | 95.6 | 477 | 99 | d__Eukaryota;k__Alveolata;p__norank;c__Dinophyceae;o__Suessiales;f__Suessia |

| | | | | | | |
|---|---|---|---|---|---|---|
| | | | | | | ceae;g__Pelagodinium;s__Pelagodinium_béii |
| OTU2198 | 503 | JN558107 | 73.87 | 509 | 100 | d__Eukaryota;k__Alveolata;p__norank;c__Dinophyceae;o__Suessiales;f__Suessiaceae;g__Pelagodinium;s__Pelagodinium_sp. |
| OTU2202 | 478 | JN558107 | 96.44 | 478 | 100 | d__Eukaryota;k__Alveolata;p__norank;c__Dinophyceae;o__Suessiales;f__Suessiaceae;g__Pelagodinium;s__Pelagodinium_béii |
| OTU2206 | 443 | JN558107 | 94 | 147 | 46 | d__Eukaryota;k__Alveolata;p__norank;c__Dinophyceae;o__norank;f__norank;g__norank;s__Unclassified DINO |
| OTU2207 | 489 | JN558110 | 77.92 | 548 | 100 | d__Eukaryota;k__Alveolata;p__norank;c__Dinophyceae;o__Suessiales;f__Suessiaceae;g__Polarella;s__Polarella_glacialis |
| OTU2208 | 485 | JN558110 | 83 | 485 | 99 | d__Eukaryota;k__Alveolata;p__norank;c__Dinophyceae;o__Suessiales;f__Suessiaceae;g__Polarella;s__Polarella_sp. |
| OTU2211 | 481 | JN558110 | 97.51 | 481 | 100 | d__Eukaryota;k__Alveolata;p__norank;c__Dinophyceae;o__Suessiales;f__Suessiaceae;g__Polarella;s__Polarella_glacialis |
| OTU2212 | 489 | JN558110 | 79 | 544 | 97 | d__Eukaryota;k__Alveolata;p__norank;c__Dinophyceae;o__Suessiales;f__Suessiaceae;g__Polarella;s__Polarella_sp. |
| OTU2213 | 469 | JN558110 | 90.04 | 472 | 100 | d__Eukaryota;k__Alveolata;p__norank;c__Dinophyceae;o__Suessiales;f__Suessiaceae;g__Polarella;s__Polarella_glacialis |
| OTU2218 | 440 | KF031312 | 77.95 | 449 | 100 | d__Eukaryota;k__Alveolata;p__norank;c__Dinophyceae;o__Peridiniales;f__Heterocapsaceae;g__Heterocapsa;s__Heterocapsa_minima |
| OTU2224 | 495 | JN119844 | 80.24 | 506 | 100 | d__Eukaryota;k__Alveolata;p__norank;c__Dinophyceae;o__Peridiniales;f__Heterocapsaceae;g__Heterocapsa;s__Heterocapsa_sp._HCBC88 |
| OTU225 | 480 | AY154960 | 98.96 | 480 | 100 | d__Eukaryota;k__Alveolata;p__norank;c__Dinophyceae;o__Gonyaulacales;f__Gonyaulacaceae;g__Gonyaulax;s__Gonyaulax_cf._spinifera |
| OTU2258 | 498 | HQ902267 | 71 | 531 | 100 | d__Eukaryota;k__Alveolata;p__norank;c__Dinophyceae;o__Peridiniales;f__Heterocapsaceae;g__Heterocapsa;s__Heterocapsa_triquetra |
| OTU226 | 486 | AY154960 | 97.54 | 407 | 83 | d__Eukaryota;k__Alveolata;p__norank;c__Dinophyceae;o__Gonyaulacales;f__Gonyaulacaceae;g__Gonyaulax;s__Gonyaulax_cf._spinifera |
| OTU2261 | 492 | AF260400 | 81.25 | 496 | 100 | d__Eukaryota;k__Alveolata;p__norank;c__Dinophyceae;o__Peridiniales;f__Heterocapsaceae;g__Heterocapsa;s__Heterocapsa_rotundata |
| OTU2263 | 496 | JQ247713 | 81.6 | 500 | 100 | d__Eukaryota;k__Alveolata;p__norank;c__Dinophyceae;o__Peridiniales;f__Heterocapsaceae;g__Heterocapsa;s__Heterocapsa_niei |
| OTU2264 | 510 | HQ902267 | 72.46 | 541 | 99 | d__Eukaryota;k__Alveolata;p__norank;c__Dinophyceae;o__Peridiniales;f__Heterocapsaceae;g__Heterocapsa;s__Heterocapsa_triquetra |
| OTU2265 | 493 | JQ247714 | 88.38 | 499 | 100 | d__Eukaryota;k__Alveolata;p__norank;c__Dinophyceae;o__Gonyaulacales;f__Cladopyxidaceae;g__Peridiniella;s__Peridiniella_sp._NC-2011 |
| OTU2266 | 515 | HQ902267 | 81.98 | 516 | 100 | d__Eukaryota;k__Alveolata;p__norank;c__Dinophyceae;o__Peridiniales;f__Heterocapsaceae;g__Heterocapsa;s__Heterocapsa_triquetra |
| OTU2267 | 516 | JQ247714 | 93.86 | 521 | 100 | d__Eukaryota;k__Alveolata;p__norank;c__Dinophyceae;o__Gonyaulacales;f__Cladopyxidaceae;g__Peridiniella;s__Peridiniella_sp._NC-2011 |
| OTU2269 | 497 | JQ247713 | 83 | 500 | 100 | d__Eukaryota;k__Alveolata;p__norank;c__Dinophyceae;o__Peridiniales;f__Heter |

| | | | | | | |
|---|---|---|---|---|---|---|
| | | | | | | ocapsaceae;g__Heterocapsa;s__Heterocapsa_niei |
| OTU227 | 487 | AY154960 | 96.45 | 479 | 98 | d__Eukaryota;k__Alveolata;p__norank;c__Dinophyceae;o__Gonyaulacales;f__Gonyaulacaceae;g__Gonyaulax;s__Gonyaulax_cf._spinifera |
| OTU2271 | 513 | JQ247714 | 92.65 | 517 | 100 | d__Eukaryota;k__Alveolata;p__norank;c__Dinophyceae;o__Gonyaulacales;f__Cladopyxidaceae;g__Peridiniella;s__Peridiniella_sp._NC-2011 |
| OTU2277 | 446 | HQ890883 | 98 | 47 | 9 | d__Eukaryota;k__Alveolata;p__norank;c__Dinophyceae;o__norank;f__norank;g__norank;s__Unclassified DINO |
| OTU2279 | 444 | HQ890883 | 98 | 81 | 11 | d__Eukaryota;k__Alveolata;p__norank;c__Dinophyceae;o__norank;f__norank;g__norank;s__Unclassified DINO |
| OTU228 | 483 | AY154960 | 97.49 | 478 | 99 | d__Eukaryota;k__Alveolata;p__norank;c__Dinophyceae;o__Gonyaulacales;f__Gonyaulacaceae;g__Gonyaulax;s__Gonyaulax_cf._spinifera |
| OTU2280 | 398 | HQ890883 | 98 | 73 | 11 | d__Eukaryota;k__Alveolata;p__norank;c__Dinophyceae;o__norank;f__norank;g__norank;s__Unclassified DINO |
| OTU2281 | 319 | HQ176319 | 89.47 | 38 | 37 | d__Eukaryota;k__Alveolata;p__norank;c__Dinophyceae;o__norank;f__norank;g__norank;s__Unclassified DINO |
| OTU2305 | 544 | JN606065 | 97.8 | 545 | 100 | d__Eukaryota;k__Alveolata;p__norank;c__Dinophyceae;o__Syndiniales;f__Euduboscquellidae;g__Euduboscquella;s__Euduboscquella_crenulata |
| OTU2310 | 533 | JN606065 | 98.12 | 533 | 100 | d__Eukaryota;k__Alveolata;p__norank;c__Dinophyceae;o__Syndiniales;f__Euduboscquellidae;g__Euduboscquella;s__Euduboscquella_crenulata |
| OTU2311 | 425 | JN606065 | 95.67 | 416 | 98 | d__Eukaryota;k__Alveolata;p__norank;c__Dinophyceae;o__Syndiniales;f__Euduboscquellidae;g__Euduboscquella;s__Euduboscquella_crenulata |
| OTU2316 | 542 | JN934984 | 96.68 | 542 | 100 | d__Eukaryota;k__Alveolata;p__norank;c__Dinophyceae;o__Syndiniales;f__Euduboscquellidae;g__Euduboscquella;s__Euduboscquella_sp._ex_Tintinnopsis_sp._1 |
| OTU2321 | 359 | JN934984 | 94.74 | 361 | 100 | d__Eukaryota;k__Alveolata;p__norank;c__Dinophyceae;o__Syndiniales;f__Euduboscquellidae;g__Euduboscquella;s__Euduboscquella_sp._ex_Tintinnopsis_sp._1 |
| OTU2324 | 555 | JN934988 | 98.2 | 556 | 100 | d__Eukaryota;k__Alveolata;p__norank;c__Dinophyceae;o__Syndiniales;f__Euduboscquellidae;g__Euduboscquella;s__Euduboscquella_cachoni |
| OTU2325 | 561 | JN934988 | 96.8 | 563 | 100 | d__Eukaryota;k__Alveolata;p__norank;c__Dinophyceae;o__Syndiniales;f__Euduboscquellidae;g__Euduboscquella;s__Euduboscquella_cachoni |
| OTU2326 | 544 | JN934988 | 99.26 | 537 | 99 | d__Eukaryota;k__Alveolata;p__norank;c__Dinophyceae;o__Syndiniales;f__Euduboscquellidae;g__Euduboscquella;s__Euduboscquella_cachoni |
| OTU2330 | 511 | JN934989 | 73.9 | 521 | 100 | d__Eukaryota;k__Alveolata;p__norank;c__Dinophyceae;o__Syndiniales;f__Euduboscquellidae;g__Euduboscquella;s__Euduboscquella_sp._ex_Favella_arcuata |
| OTU2332 | 498 | JN934989 | 69.26 | 501 | 99 | d__Eukaryota;k__Alveolata;p__norank;c__Dinophyceae;o__Syndiniales;f__Euduboscquellidae;g__Euduboscquella;s__Euduboscquella_sp._ex_Favella_arcuata |
| OTU2340 | 542 | JN934989 | 76.78 | 547 | 100 | d__Eukaryota;k__Alveolata;p__norank;c__Dinophyceae;o__Syndiniales;f__Euduboscquellidae;g__Euduboscquella;s__Euduboscquella_sp._ex_Favella_arcuata |
| OTU2343 | 537 | JN934989 | 76.33 | 545 | 100 | d__Eukaryota;k__Alveolata;p__norank;c__Dinophyceae;o__Syndiniales;f__Euduboscquellidae;g__Euduboscquella;s__Euduboscquella_sp._ex_Favella_arcuata |
| OTU2344 | 540 | JN934994 | 99.63 | 540 | 100 | d__Eukaryota;k__Alveolata;p__norank;c__Dinophyceae;o__Syndiniales;f__Eudub |

| | | | | | | |
|---|---|---|---|---|---|---|
| | | | | | | osquellidae;g__Euduboscquella;s__Euduboscquella_sp._ex_Tintinnopsis_sp._2 |
| OTU2345 | 477 | JN934999 | 90.79 | 76 | 27 | d__Eukaryota;k__Alveolata;p__norank;c__Dinophyceae;o__Syndiniales;f__Eudub osquellidae;g__norank;s__Unclassified Eudubosquellidae species |
| OTU2348 | 503 | FR877580 | 93.47 | 505 | 100 | d__Eukaryota;k__Alveolata;p__norank;c__Dinophyceae;o__Gonyaulacales;f__Am phidomataceae;g__Azadinium;s__Azadinium_cf._poporum_HJ-2011 |
| OTU2350 | 499 | FR877580 | 96.99 | 499 | 100 | d__Eukaryota;k__Alveolata;p__norank;c__Dinophyceae;o__Gonyaulacales;f__Am phidomataceae;g__Azadinium;s__Azadinium_cf._poporum_HJ-2011 |
| OTU236 | 473 | AY154960 | 81.54 | 65 | 47 | d__Eukaryota;k__Alveolata;p__norank;c__Dinophyceae;o__Gonyaulacales;f__Go nyaulacaceae;g__norank;s__Unclassified DINO |
| OTU237 | 470 | AY154960 | 94.89 | 489 | 100 | d__Eukaryota;k__Alveolata;p__norank;c__Dinophyceae;o__Gonyaulacales;f__Go nyaulacaceae;g__Gonyaulax;s__Gonyaulax_cf._spinifera |
| OTU2393 | 477 | JQ616825 | 92 | 106 | 32 | d__Eukaryota;k__Alveolata;p__norank;c__Dinophyceae;o__Gymnodiniales;f__no rank;g__norank;s__Unclassified DINO |
| OTU2399 | 490 | JF921198 | 98.78 | 490 | 100 | d__Eukaryota;k__Alveolata;p__norank;c__Dinophyceae;o__Gonyaulacales;f__Go nyaulacaceae;g__Alexandrium;s__Alexandrium_peruvianum |
| OTU240 | 436 | AY154960 | 96.45 | 422 | 97 | d__Eukaryota;k__Alveolata;p__norank;c__Dinophyceae;o__Gonyaulacales;f__Go nyaulacaceae;g__Gonyaulax;s__Gonyaulax_cf._spinifera |
| OTU2407 | 494 | JQ616825 | 76.41 | 496 | 99 | d__Eukaryota;k__Alveolata;p__norank;c__Dinophyceae;o__Gymnodiniales;f__G ymnodiniaceae;g__Gymnodinium;s__Gymnodinium_catenatum |
| OTU2408 | 507 | AY331681 | 81.57 | 510 | 100 | d__Eukaryota;k__Alveolata;p__norank;c__Dinophyceae;o__Gymnodiniales;f__G ymnodiniaceae;g__Lepidodinium;s__Lepidodinium_chlorophorum |
| OTU2419 | 460 | KP702713 | 74.19 | 461 | 98 | d__Eukaryota;k__Alveolata;p__norank;c__Dinophyceae;o__Peridiniales;f__Diplo psaliaceae;g__Lebouraia;s__Lebouraia_pusilla |
| OTU2425 | 409 | HM596557 | 90 | 154 | 50 | d__Eukaryota;k__Alveolata;p__norank;c__Dinophyceae;o__Gymnodiniales;f__G ymnodiniaceae;g__norank;s__Unclassified gymnodiniaceae species |
| OTU2426 | 460 | JX262498 | 78.46 | 455 | 96 | d__Eukaryota;k__Alveolata;p__norank;c__Dinophyceae;o__Peridiniales;f__Peridi niaceae;g__Pentapharsodinium;s__Pentapharsodinium_sp. |
| OTU2428 | 235 | JQ639752 | 85.71 | 189 | 80 | d__Eukaryota;k__Alveolata;p__norank;c__Dinophyceae;o__Peridiniales;f__Gleno diniaceae;g__Peridiniopsis;s__Peridiniopsis_minima |
| OTU2433 | 519 | FN557541 | 94.38 | 89 | 54 | d__Eukaryota;k__Alveolata;p__norank;c__Dinophyceae;o__unclassified_Dinoph yceae;f__norank;g__norank;s__Unclassified DINO |
| OTU2442 | 496 | JQ413373 | 89.56 | 498 | 100 | d__Eukaryota;k__Alveolata;p__norank;c__Dinophyceae;o__Suessiales;f__Biechele riaceae;g__Biecheleria;s__Biecheleria_cincta |
| OTU2446 | 507 | FJ024705 | 89.11 | 505 | 99 | d__Eukaryota;k__Alveolata;p__norank;c__Dinophyceae;o__Suessiales;f__Biechele riaceae;g__Biecheleria;s__Biecheleria_cincta |
| OTU2447 | 502 | FJ024705 | 88.14 | 506 | 100 | d__Eukaryota;k__Alveolata;p__norank;c__Dinophyceae;o__Suessiales;f__Biechele riaceae;g__Biecheleria;s__Biecheleria_cincta |
| OTU2448 | 384 | JQ413374 | 96.35 | 384 | 100 | d__Eukaryota;k__Alveolata;p__norank;c__Dinophyceae;o__Suessiales;f__Biechele riaceae;g__Biecheleria;s__Biecheleria_cincta |
| OTU2450 | 492 | FJ024705 | 86.87 | 495 | 100 | d__Eukaryota;k__Alveolata;p__norank;c__Dinophyceae;o__Suessiales;f__Biechele |

| | | | | | | |
|---|---|---|---|---|---|---|
| | | | | | | riaceae;g__Biecheleria;s__Biecheleria_cincta |
| OTU2451 | 481 | FJ024705 | 91.12 | 484 | 100 | d__Eukaryota;k__Alveolata;p__norank;c__Dinophyceae;o__Suessiales;f__Biecheleriaceae;g__Biecheleria;s__Biecheleria_cincta |
| OTU2452 | 487 | FJ024705 | 95.89 | 487 | 100 | d__Eukaryota;k__Alveolata;p__norank;c__Dinophyceae;o__Suessiales;f__Biecheleriaceae;g__Biecheleria;s__Biecheleria_cincta |
| OTU2453 | 447 | JQ413374 | 97.32 | 447 | 100 | d__Eukaryota;k__Alveolata;p__norank;c__Dinophyceae;o__Suessiales;f__Biecheleriaceae;g__Biecheleria;s__Biecheleria_cincta |
| OTU2454 | 431 | JQ413374 | 97.65 | 426 | 99 | d__Eukaryota;k__Alveolata;p__norank;c__Dinophyceae;o__Suessiales;f__Biecheleriaceae;g__Biecheleria;s__Biecheleria_cincta |
| OTU2455 | 506 | FJ024705 | 88.39 | 508 | 100 | d__Eukaryota;k__Alveolata;p__norank;c__Dinophyceae;o__Suessiales;f__Biecheleriaceae;g__Biecheleria;s__Biecheleria_cincta |
| OTU2456 | 444 | JQ413374 | 96.26 | 454 | 100 | d__Eukaryota;k__Alveolata;p__norank;c__Dinophyceae;o__Suessiales;f__Biecheleriaceae;g__Biecheleria;s__Biecheleria_cincta |
| OTU2457 | 428 | JQ413374 | 97.44 | 430 | 100 | d__Eukaryota;k__Alveolata;p__norank;c__Dinophyceae;o__Suessiales;f__Biecheleriaceae;g__Biecheleria;s__Biecheleria_cincta |
| OTU2458 | 505 | AB858353 | 91.68 | 505 | 100 | d__Eukaryota;k__Alveolata;p__norank;c__Dinophyceae;o__Suessiales;f__Biecheleriaceae;g__Biecheleria;s__Biecheleria_brevisulcata |
| OTU2459 | 489 | FJ024705 | 90.1 | 495 | 100 | d__Eukaryota;k__Alveolata;p__norank;c__Dinophyceae;o__Suessiales;f__Biecheleriaceae;g__Biecheleria;s__Biecheleria_cincta |
| OTU2461 | 389 | JQ413374 | 95.85 | 386 | 99 | d__Eukaryota;k__Alveolata;p__norank;c__Dinophyceae;o__Suessiales;f__Biecheleriaceae;g__Biecheleria;s__Biecheleria_cincta |
| OTU2463 | 404 | JQ413374 | 97.28 | 404 | 100 | d__Eukaryota;k__Alveolata;p__norank;c__Dinophyceae;o__Suessiales;f__Biecheleriaceae;g__Biecheleria;s__Biecheleria_cincta |
| OTU2466 | 403 | JQ413374 | 96.78 | 404 | 100 | d__Eukaryota;k__Alveolata;p__norank;c__Dinophyceae;o__Suessiales;f__Biecheleriaceae;g__Biecheleria;s__Biecheleria_cincta |
| OTU2467 | 492 | FJ024705 | 86.49 | 496 | 100 | d__Eukaryota;k__Alveolata;p__norank;c__Dinophyceae;o__Suessiales;f__Biecheleriaceae;g__Biecheleria;s__Biecheleria_cincta |
| OTU2468 | 356 | JQ413374 | 94.38 | 356 | 100 | d__Eukaryota;k__Alveolata;p__norank;c__Dinophyceae;o__Suessiales;f__Biecheleriaceae;g__Biecheleria;s__Biecheleria_cincta |
| OTU2470 | 486 | JQ413374 | 84.54 | 485 | 99 | d__Eukaryota;k__Alveolata;p__norank;c__Dinophyceae;o__Suessiales;f__Biecheleriaceae;g__Biecheleria;s__Biecheleria_cincta |
| OTU2471 | 470 | JQ413374 | 98.67 | 300 | 63 | d__Eukaryota;k__Alveolata;p__norank;c__Dinophyceae;o__Suessiales;f__Biecheleriaceae;g__Biecheleria;s__Biecheleria-like_sp. |
| OTU2473 | 485 | JQ413374 | 98.68 | 303 | 62 | d__Eukaryota;k__Alveolata;p__norank;c__Dinophyceae;o__Suessiales;f__Biecheleriaceae;g__Biecheleria;s__Biecheleria-like_sp. |
| OTU2474 | 309 | JQ413374 | 96.44 | 309 | 100 | d__Eukaryota;k__Alveolata;p__norank;c__Dinophyceae;o__Suessiales;f__Biecheleriaceae;g__Biecheleria;s__Biecheleria_cincta |
| OTU2475 | 312 | JQ413374 | 93.2 | 309 | 99 | d__Eukaryota;k__Alveolata;p__norank;c__Dinophyceae;o__Suessiales;f__Biecheleriaceae;g__Biecheleria;s__Biecheleria_cincta |
| OTU2488 | 226 | HQ845329 | 84.58 | 227 | 99 | d__Eukaryota;k__Alveolata;p__norank;c__Dinophyceae;o__Peridiniales;f__Peridi |

| | | | | | | |
|---|---|---|---|---|---|---|
| | | | | | | niaceae;g__Pentapharsodinium;s__Pentapharsodinium_tyrrhenicum |
| OTU2492 | 512 | JQ179865 | 80 | 78 | 61 | d__Eukaryota;k__Alveolata;p__norank;c__Dinophyceae;o__Gymnodiniales;f__Unclassified Gymnodiniaceae;g__norank;s__Unclassified Gymnodiniaceae |
| OTU2495 | 501 | KP790208 | 89.47 | 76 | 15 | d__Eukaryota;k__Alveolata;p__norank;c__Dinophyceae;o__norank;f__norank;g__norank;s__Unclassifid DINO |
| OTU2496 | 517 | KP790208 | 89.47 | 76 | 15 | d__Eukaryota;k__Alveolata;p__norank;c__Dinophyceae;o__norank;f__norank;g__norank;s__Unclassifid DINO |
| OTU2497 | 515 | AB702991 | 99.42 | 515 | 100 | d__Eukaryota;k__Alveolata;p__norank;c__Dinophyceae;o__Peridiniales;f__Amphidiniopsidaceae;g__Archaeperidinium;s__Archaeperidinium_saanichi |
| OTU25 | 503 | AF260392 | 85.74 | 505 | 100 | d__Eukaryota;k__Alveolata;p__norank;c__Dinophyceae;o__Peridiniales;f__Thoracosphaeraceae;g__Scrippsiella;s__Scrippsiella_sp. |
| OTU250 | 462 | AY154962 | 90.52 | 464 | 100 | d__Eukaryota;k__Alveolata;p__norank;c__Dinophyceae;o__Gonyaulacales;f__Gonyaulacaceae;g__Gonyaulax;s__Gonyaulax_baltica |
| OTU2537 | 477 | HQ845327 | 88 | 84 | 23 | d__Eukaryota;k__Alveolata;p__norank;c__Dinophyceae;o__norank;f__norank;g__norank;s__Unclassified DINO |
| OTU2560 | 508 | HQ902267 | 73.01 | 515 | 98 | d__Eukaryota;k__Alveolata;p__norank;c__Dinophyceae;o__Peridiniales;f__Heterocapsaceae;g__Heterocapsa;s__Heterocapsa_triquetra |
| OTU2561 | 502 | EU165273 | 84.34 | 198 | 39 | d__Eukaryota;k__Alveolata;p__norank;c__Dinophyceae;o__Peridiniales;f__Heterocapsaceae;g__unclassified_Heterocapsaceae;s__Unclassified Heterocapsaceae_sp. |
| OTU2565 | 311 | JN982387 | 97.75 | 311 | 100 | d__Eukaryota;k__Alveolata;p__norank;c__Dinophyceae;o__Peridiniales;f__Thoracosphaeraceae;g__Scrippsiella;s__Scrippsiella_donghaienis |
| OTU2567 | 248 | JN982387 | 96.33 | 245 | 99 | d__Eukaryota;k__Alveolata;p__norank;c__Dinophyceae;o__Peridiniales;f__Thoracosphaeraceae;g__Scrippsiella;s__Scrippsiella_donghaienis |
| OTU2572 | 484 | HM483396 | 91.21 | 478 | 99 | d__Eukaryota;k__Alveolata;p__norank;c__Dinophyceae;o__Peridiniales;f__Thoracosphaeraceae;g__Scrippsiella;s__Scrippsiella_trochoidea |
| OTU2582 | 505 | HQ902267 | 73.15 | 514 | 99 | d__Eukaryota;k__Alveolata;p__norank;c__Dinophyceae;o__Peridiniales;f__Heterocapsaceae;g__Heterocapsa;s__Heterocapsa_triquetra |
| OTU2583 | 464 | AF260400 | 71.22 | 476 | 99 | d__Eukaryota;k__Alveolata;p__norank;c__Dinophyceae;o__Peridiniales;f__Heterocapsaceae;g__Heterocapsa;s__Heterocapsa_rotundata |
| OTU2586 | 437 | HM483396 | 87.53 | 465 | 100 | d__Eukaryota;k__Alveolata;p__norank;c__Dinophyceae;o__Peridiniales;f__Thoracosphaeraceae;g__Scrippsiella;s__Scrippsiella_trochoidea |
| OTU2588 | 290 | JN982392 | 94.96 | 278 | 96 | d__Eukaryota;k__Alveolata;p__norank;c__Dinophyceae;o__Peridiniales;f__Thoracosphaeraceae;g__Scrippsiella;s__Scrippsiella_sweeneyae |
| OTU2602 | 298 | KJ189491 | 97.99 | 299 | 100 | d__Eukaryota;k__Alveolata;p__norank;c__Dinophyceae;o__Peridiniales;f__Thoracosphaeraceae;g__Scrippsiella;s__Scrippsiella_cf._acuminata |
| OTU2609 | 260 | KJ189491 | 95.87 | 242 | 93 | d__Eukaryota;k__Alveolata;p__norank;c__Dinophyceae;o__Peridiniales;f__Thoracosphaeraceae;g__Scrippsiella;s__Scrippsiella_cf._acuminata |
| OTU2614 | 281 | JN982400 | 98.12 | 266 | 95 | d__Eukaryota;k__Alveolata;p__norank;c__Dinophyceae;o__Peridiniales;f__Thoracosphaeraceae;g__Scrippsiella;s__Scrippsiella_aff._acuminata |

| | | | | | | |
|---|---|---|---|---|---|---|
| OTU2616 | 260 | JN982400 | 92.25 | 258 | 99 | d__Eukaryota;k__Alveolata;p__norank;c__Dinophyceae;o__Peridiniales;f__Thoracosphaeraceae;g__Scrippsiella;s__Scrippsiella_aff._acuminata |
| OTU2619 | 499 | JN982400 | 100 | 499 | 65 | d__Eukaryota;k__Alveolata;p__norank;c__Dinophyceae;o__Peridiniales;f__Thoracosphaeraceae;g__Scrippsiella;s__Scrippsiella_aff._acuminata |
| OTU262 | 455 | AY154960 | 70.06 | 471 | 100 | d__Eukaryota;k__Alveolata;p__norank;c__Dinophyceae;o__Gonyaulacales;f__Gonyaulacaceae;g__Gonyaulax;s__Gonyaulax_sp. |
| OTU2620 | 320 | JN982400 | 97.5 | 320 | 100 | d__Eukaryota;k__Alveolata;p__norank;c__Dinophyceae;o__Peridiniales;f__Thoracosphaeraceae;g__Scrippsiella;s__Scrippsiella_aff._acuminata |
| OTU2632 | 222 | KJ189485 | 91.44 | 222 | 100 | d__Eukaryota;k__Alveolata;p__norank;c__Dinophyceae;o__Peridiniales;f__Thoracosphaeraceae;g__Scrippsiella;s__Scrippsiella_aff._acuminata |
| OTU2636 | 217 | JN982402 | 93 | 200 | 92 | d__Eukaryota;k__Alveolata;p__norank;c__Dinophyceae;o__Peridiniales;f__Thoracosphaeraceae;g__Scrippsiella;s__Scrippsiella_sp. |
| OTU2641 | 299 | JN982402 | 97.57 | 288 | 96 | d__Eukaryota;k__Alveolata;p__norank;c__Dinophyceae;o__Peridiniales;f__Thoracosphaeraceae;g__Scrippsiella;s__Scrippsiella_sp. |
| OTU2644 | 513 | JX559886 | 99.1 | 442 | 86 | d__Eukaryota;k__Alveolata;p__norank;c__Dinophyceae;o__Gonyaulacales;f__Amphidomataceae;g__Azadinium;s__Azadinium_polongum |
| OTU265 | 499 | JX912182.1 | 90 | 175 | 35 | d__Eukaryota;k__Alveolata;p__norank;c__Dinophyceae;o__unclassified;f__unclassified;g__unclassified;s__Unclassified DINO |
| OTU2652 | 497 | DQ195367 | 86.21 | 87 | 63 | d__Eukaryota;k__Alveolata;p__norank;c__Dinophyceae;o__Suessiales;f__Suessiaceae;g__Pelagodinium;s__Pelagodinium-like species |
| OTU2653 | 523 | AB716928 | 90.06 | 523 | 100 | d__Eukaryota;k__Alveolata;p__norank;c__Dinophyceae;o__Peridiniales;f__Protoperidiniaceae;g__Protoperidinium;s__Protoperidinium_monovelum |
| OTU2655 | 506 | AB716928 | 96.05 | 506 | 100 | d__Eukaryota;k__Alveolata;p__norank;c__Dinophyceae;o__Peridiniales;f__Protoperidiniaceae;g__Protoperidinium;s__Protoperidinium_monovelum |
| OTU2656 | 499 | AB716928 | 90.38 | 499 | 100 | d__Eukaryota;k__Alveolata;p__norank;c__Dinophyceae;o__Peridiniales;f__Protoperidiniaceae;g__Protoperidinium;s__Protoperidinium_monovelum |
| OTU2665 | 510 | AB716928 | 90.14 | 507 | 99 | d__Eukaryota;k__Alveolata;p__norank;c__Dinophyceae;o__Peridiniales;f__Protoperidiniaceae;g__Protoperidinium;s__Protoperidinium_monovelum |
| OTU2666 | 490 | AB716928 | 91.65 | 491 | 100 | d__Eukaryota;k__Alveolata;p__norank;c__Dinophyceae;o__Peridiniales;f__Protoperidiniaceae;g__Protoperidinium;s__Protoperidinium_monovelum |
| OTU2669 | 359 | AB716928 | 86.35 | 359 | 100 | d__Eukaryota;k__Alveolata;p__norank;c__Dinophyceae;o__Peridiniales;f__Protoperidiniaceae;g__Protoperidinium;s__Protoperidinium_monovelum |
| OTU2674 | 492 | AB716928 | 89.11 | 496 | 100 | d__Eukaryota;k__Alveolata;p__norank;c__Dinophyceae;o__Peridiniales;f__Protoperidiniaceae;g__Protoperidinium;s__Protoperidinium_monovelum |
| OTU2675 | 480 | AB716929 | 98.8 | 125 | 61 | d__Eukaryota;k__Alveolata;p__norank;c__Dinophyceae;o__Peridiniales;f__Protoperidiniaceae;g__Protoperidinium;s__Protoperidinium-like_sp. |
| OTU2679 | 440 | AB716929 | 97.6 | 125 | 64 | d__Eukaryota;k__Alveolata;p__norank;c__Dinophyceae;o__Peridiniales;f__Protoperidiniaceae;g__Protoperidinium;s__Protoperidinium-like_sp. |
| OTU2680 | 459 | AB716929 | 96.8 | 125 | 59 | d__Eukaryota;k__Alveolata;p__norank;c__Dinophyceae;o__Peridiniales;f__Protoperidiniaceae;g__Protoperidinium;s__Protoperidinium-like_sp. |

| | | | | | | |
|---|---|---|---|---|---|---|
| OTU2708 | 515 | JX262498 | 87.77 | 515 | 99 | d__Eukaryota;k__Alveolata;p__norank;c__Dinophyceae;o__Peridiniales;f__Peridiniaceae;g__Pentapharsodinium;s__Pentapharsodinium_dalei |
| OTU2709 | 492 | JX262498 | 100 | 492 | 100 | d__Eukaryota;k__Alveolata;p__norank;c__Dinophyceae;o__Peridiniales;f__Peridiniaceae;g__Pentapharsodinium;s__Pentapharsodinium_dalei |
| OTU2710 | 507 | JX262498 | 89.15 | 507 | 100 | d__Eukaryota;k__Alveolata;p__norank;c__Dinophyceae;o__Peridiniales;f__Peridiniaceae;g__Pentapharsodinium;s__Pentapharsodinium_dalei |
| OTU2711 | 473 | JX262498 | 73 | 473 | 100 | d__Eukaryota;k__Alveolata;p__norank;c__Dinophyceae;o__Peridiniales;f__Peridiniaceae;g__Pentapharsodinium;s__Pentapharsodinium_sp. |
| OTU2714 | 492 | JX262498 | 95.73 | 492 | 100 | d__Eukaryota;k__Alveolata;p__norank;c__Dinophyceae;o__Peridiniales;f__Peridiniaceae;g__Pentapharsodinium;s__Pentapharsodinium_dalei |
| OTU2717 | 498 | JX262498 | 98.6 | 357 | 72 | d__Eukaryota;k__Alveolata;p__norank;c__Dinophyceae;o__Peridiniales;f__Peridiniaceae;g__Pentapharsodinium;s__Pentapharsodinium_sp. |
| OTU2723 | 527 | JX262498 | 86.55 | 528 | 100 | d__Eukaryota;k__Alveolata;p__norank;c__Dinophyceae;o__Peridiniales;f__Peridiniaceae;g__Pentapharsodinium;s__Pentapharsodinium_dalei |
| OTU2724 | 457 | JX262498 | 86.68 | 458 | 100 | d__Eukaryota;k__Alveolata;p__norank;c__Dinophyceae;o__Peridiniales;f__Peridiniaceae;g__Pentapharsodinium;s__Pentapharsodinium_dalei |
| OTU2730 | 418 | JX262498 | 95.92 | 147 | 98 | d__Eukaryota;k__Alveolata;p__norank;c__Dinophyceae;o__Peridiniales;f__Peridiniaceae;g__Pentapharsodinium;s__Pentapharsodinium_dalei |
| OTU2738 | 386 | KP702719 | 98.19 | 387 | 100 | d__Eukaryota;k__Alveolata;p__norank;c__Dinophyceae;o__Peridiniales;f__Diplopsaliaceae;g__Oblea;s__Oblea_rotunda |
| OTU2740 | 484 | JX262498 | 95.66 | 484 | 100 | d__Eukaryota;k__Alveolata;p__norank;c__Dinophyceae;o__Peridiniales;f__Peridiniaceae;g__Pentapharsodinium;s__Pentapharsodinium_dalei |
| OTU2743 | 425 | JX262498 | 83.8 | 426 | 100 | d__Eukaryota;k__Alveolata;p__norank;c__Dinophyceae;o__Peridiniales;f__Peridiniaceae;g__Pentapharsodinium;s__Pentapharsodinium_dalei |
| OTU2748 | 490 | JX262498 | 92.67 | 505 | 100 | d__Eukaryota;k__Alveolata;p__norank;c__Dinophyceae;o__Peridiniales;f__Peridiniaceae;g__Pentapharsodinium;s__Pentapharsodinium_dalei |
| OTU2750 | 490 | JX262498 | 97.13 | 488 | 99 | d__Eukaryota;k__Alveolata;p__norank;c__Dinophyceae;o__Peridiniales;f__Peridiniaceae;g__Pentapharsodinium;s__Pentapharsodinium_dalei |
| OTU2753 | 487 | JX262498 | 94.06 | 488 | 100 | d__Eukaryota;k__Alveolata;p__norank;c__Dinophyceae;o__Peridiniales;f__Peridiniaceae;g__Pentapharsodinium;s__Pentapharsodinium_dalei |
| OTU2768 | 501 | JQ247713 | 73.05 | 512 | 100 | d__Eukaryota;k__Alveolata;p__norank;c__Dinophyceae;o__Peridiniales;f__Heterocapsaceae;g__Heterocapsa;s__Heterocapsa_niei |
| OTU277 | 467 | KF998563 | 88.89 | 90 | 26 | d__Eukaryota;k__Alveolata;p__norank;c__Dinophyceae;o__unclassified;f__unclassified;g__unclassified;s__Unclassified DINO |
| OTU2799 | 483 | JN558110 | 83 | 33 | 95 | d__Eukaryota;k__Alveolata;p__norank;c__Dinophyceae;o__Suessiales;f__Suessiaceae;g__Polarella;s__Polarella_sp. |
| OTU280 | 477 | AY154965 | 77.62 | 496 | 99 | d__Eukaryota;k__Alveolata;p__norank;c__Dinophyceae;o__Gonyaulacales;f__Gonyaulacaceae;g__Gonyaulax;s__Gonyaulax_sp. |
| OTU2801 | 215 | KC895475 | 99.49 | 197 | 92 | d__Eukaryota;k__Alveolata;p__norank;c__Dinophyceae;o__Suessiales;f__Biecheleriaceae;g__Biecheleria;s__Biecheleria_cincta |

| | | | | | | |
|---|---|---|---|---|---|---|
| OTU2804 | 235 | KC895475 | 97.42 | 233 | 99 | d__Eukaryota;k__Alveolata;p__norank;c__Dinophyceae;o__Suessiales;f__Biecheleriaceae;g__Biecheleria;s__Biecheleria_cincta |
| OTU2806 | 205 | KC895475 | 99.49 | 196 | 95 | d__Eukaryota;k__Alveolata;p__norank;c__Dinophyceae;o__Suessiales;f__Biecheleriaceae;g__Biecheleria;s__Biecheleria_cincta |
| OTU2807 | 262 | KC895475 | 97.69 | 260 | 99 | d__Eukaryota;k__Alveolata;p__norank;c__Dinophyceae;o__Suessiales;f__Biecheleriaceae;g__Biecheleria;s__Biecheleria_cincta |
| OTU2808 | 219 | KC895475 | 96.36 | 220 | 100 | d__Eukaryota;k__Alveolata;p__norank;c__Dinophyceae;o__Suessiales;f__Biecheleriaceae;g__Biecheleria;s__Biecheleria_cincta |
| OTU2809 | 221 | KC895475 | 96.38 | 221 | 100 | d__Eukaryota;k__Alveolata;p__norank;c__Dinophyceae;o__Suessiales;f__Biecheleriaceae;g__Biecheleria;s__Biecheleria_cincta |
| OTU2811 | 209 | KC895475 | 99.49 | 198 | 95 | d__Eukaryota;k__Alveolata;p__norank;c__Dinophyceae;o__Suessiales;f__Biecheleriaceae;g__Biecheleria;s__Biecheleria_cincta |
| OTU2812 | 228 | KC895475 | 98.66 | 224 | 98 | d__Eukaryota;k__Alveolata;p__norank;c__Dinophyceae;o__Suessiales;f__Biecheleriaceae;g__Biecheleria;s__Biecheleria_cincta |
| OTU2813 | 262 | KC895475 | 96.95 | 262 | 100 | d__Eukaryota;k__Alveolata;p__norank;c__Dinophyceae;o__Suessiales;f__Biecheleriaceae;g__Biecheleria;s__Biecheleria_cincta |
| OTU2856 | 478 | KP790181 | 89.42 | 104 | 22 | d__Eukaryota;k__Alveolata;p__norank;c__Dinophyceae;o__norank;f__norank;g__norank;s__Unclassified DINO |
| OTU2858 | 496 | HQ834210 | 78.67 | 497 | 100 | d__Eukaryota;k__Alveolata;p__norank;c__Dinophyceae;o__Gymnodiniales;f__Polykrikaceae;g__Pheopolykrikos;s__Pheopolykrikos_hartmannii-like |
| OTU2952 | 489 | KF245462 | 91.24 | 491 | 100 | d__Eukaryota;k__Alveolata;p__norank;c__Dinophyceae;o__Gymnodiniales;f__Gymnodiniaceae;g__Gymnodinium;s__Gymnodinium_sp. |
| OTU2953 | 491 | KF031312 | 99.32 | 443 | 90 | d__Eukaryota;k__Alveolata;p__norank;c__Dinophyceae;o__Peridiniales;f__Heterocapsaceae;g__Heterocapsa;s__Heterocapsa_minima |
| OTU2961 | 503 | JQ247713 | 83.4 | 512 | 99 | d__Eukaryota;k__Alveolata;p__norank;c__Dinophyceae;o__Peridiniales;f__Heterocapsaceae;g__Heterocapsa;s__Heterocapsa_niei |
| OTU2962 | 491 | AY571372 | 75.36 | 491 | 98 | d__Eukaryota;k__Alveolata;p__norank;c__Dinophyceae;o__Peridiniales;f__Heterocapsaceae;g__Heterocapsa;s__Heterocapsa_arctica |
| OTU2965 | 257 | KF240778 | 96.96 | 263 | 100 | d__Eukaryota;k__Alveolata;p__norank;c__Dinophyceae;o__Peridiniales;f__Heterocapsaceae;g__Heterocapsa;s__Heterocapsa_rotundata |
| OTU2966 | 337 | KF240778 | 96.15 | 338 | 100 | d__Eukaryota;k__Alveolata;p__norank;c__Dinophyceae;o__Peridiniales;f__Heterocapsaceae;g__Heterocapsa;s__Heterocapsa_rotundata |
| OTU2967 | 425 | KF240778 | 97.19 | 427 | 100 | d__Eukaryota;k__Alveolata;p__norank;c__Dinophyceae;o__Peridiniales;f__Heterocapsaceae;g__Heterocapsa;s__Heterocapsa_rotundata |
| OTU2969 | 313 | KF240778 | 98.06 | 310 | 99 | d__Eukaryota;k__Alveolata;p__norank;c__Dinophyceae;o__Peridiniales;f__Heterocapsaceae;g__Heterocapsa;s__Heterocapsa_rotundata |
| OTU2971 | 504 | JN119844 | 83.2 | 506 | 100 | d__Eukaryota;k__Alveolata;p__norank;c__Dinophyceae;o__Peridiniales;f__Heterocapsaceae;g__Heterocapsa;s__Heterocapsa_sp._HCBC88 |
| OTU2972 | 435 | KF240778 | 96.34 | 437 | 100 | d__Eukaryota;k__Alveolata;p__norank;c__Dinophyceae;o__Peridiniales;f__Heterocapsaceae;g__Heterocapsa;s__Heterocapsa_rotundata |

| | | | | | | |
|---|---|---|---|---|---|---|
| OTU2984 | 519 | KF543359 | 96.29 | 431 | 83 | d__Eukaryota;k__Alveolata;p__norank;c__Dinophyceae;o__Gonyaulacales;f__Amphidomataceae;g__Azadinium;s__Azadinium_dalianense |
| OTU2999 | 494 | FJ808705 | 90.91 | 66 | 48 | d__Eukaryota;k__Alveolata;p__norank;c__Dinophyceae;o__norank;f__norank;g__norank;s__Unclassified DINO |
| OTU3 | 385 | FN649409 | 88.79 | 116 | 30 | d__Eukaryota;k__Alveolata;p__norank;c__Dinophyceae;o__unclassified;f__unclassified;g__unclassified;s__unclassified DINO |
| OTU3016 | 523 | HQ176320 | 93.02 | 86 | 54 | d__Eukaryota;k__Alveolata;p__norank;c__Dinophyceae;o__Peridiniales;f__norank;g__norank;s__Unclassified DINO |
| OTU3021 | 527 | KJ508376 | 85.26 | 95 | 63 | d__Eukaryota;k__Alveolata;p__norank;c__Dinophyceae;o__Gymnodiniales;f__Kareniaceae;g__norank;s__Unclassified Kareniaceae |
| OTU3033 | 509 | KF651047 | 94.5 | 509 | 100 | d__Eukaryota;k__Alveolata;p__norank;c__Dinophyceae;o__Peridiniales;f__Protoperidiniaceae;g__Protoperidinium;s__Protoperidinium_tricingulatum |
| OTU3063 | 490 | LC002848 | 85 | 60 | 46 | d__Eukaryota;k__Alveolata;p__norank;c__Dinophyceae;o__unclassified_Dinophyceae;f__norank;g__Pseudadenoides;s__Pseudadenoides_kofoidii (non-DINO) |
| OTU3156 | 400 | KF383297 | 88.89 | 90 | 23 | d__Eukaryota;k__Alveolata;p__norank;c__Dinophyceae;o__Suessiales;f__Symbiodiniaceae;g__Symbiodinium;s__Symbiodinium-like_sp. |
| OTU317 | 487 | AY438015 | 100 | 487 | 100 | d__Eukaryota;k__Alveolata;p__norank;c__Dinophyceae;o__Gonyaulacales;f__Gonyaulacaceae;g__Alexandrium;s__Alexandrium_affine |
| OTU323 | 510 | AY571369 | 98.82 | 510 | 100 | d__Eukaryota;k__Alveolata;p__norank;c__Dinophyceae;o__Gymnodiniales;f__Gymnodiniaceae;g__Gyrodinium;s__Gyrodinium_rubrum |
| OTU324 | 509 | KP790197 | 100 | 422 | 83 | d__Eukaryota;k__Alveolata;p__norank;c__Dinophyceae;o__Gymnodiniales;f__Gymnodiniaceae;g__Gyrodinium;s__Gyrodinium_heterogrammum |
| OTU325 | 499 | AY571369 | 88.6 | 500 | 100 | d__Eukaryota;k__Alveolata;p__norank;c__Dinophyceae;o__Gymnodiniales;f__Gymnodiniaceae;g__Gyrodinium;s__Gyrodinium_rubrum |
| OTU3256 | 524 | AB778763 | 87 | 84 | 26 | d__Eukaryota;k__Alveolata; c__Dinophyceae;o__norank;f__norank;g__norank;s__Unclassified DINO |
| OTU326 | 514 | AY571369 | 93.35 | 511 | 99 | d__Eukaryota;k__Alveolata;p__norank;c__Dinophyceae;o__Gymnodiniales;f__Gymnodiniaceae;g__Gyrodinium;s__Gyrodinium_rubrum |
| OTU327 | 512 | AY571369 | 91.23 | 513 | 100 | d__Eukaryota;k__Alveolata;p__norank;c__Dinophyceae;o__Gymnodiniales;f__Gymnodiniaceae;g__Gyrodinium;s__Gyrodinium_rubrum |
| OTU328 | 499 | KP790196 | 97.4 | 423 | 84 | d__Eukaryota;k__Alveolata;p__norank;c__Dinophyceae;o__Gymnodiniales;f__Gymnodiniaceae;g__Gyrodinium;s__Gyrodinium_heterogrammum |
| OTU3287 | 499 | EF616462 | 86.07 | 524 | 100 | d__Eukaryota;k__Alveolata;p__norank;c__Dinophyceae;o__Gymnodiniales;f__Gymnodiniaceae;g__Cochlodinium;s__Cochlodinium_cf._geminatum |
| OTU3288 | 500 | HQ834210 | 84.79 | 539 | 100 | d__Eukaryota;k__Alveolata;p__norank;c__Dinophyceae;o__Gymnodiniales;f__Polykrikaceae;g__Pheopolykrikos;s__Pheopolykrikos_hartmannii |
| OTU329 | 394 | AY571369 | 97.22 | 395 | 100 | d__Eukaryota;k__Alveolata;p__norank;c__Dinophyceae;o__Gymnodiniales;f__Gymnodiniaceae;g__Gyrodinium;s__Gyrodinium_rubrum |
| OTU3297 | 499 | KF878935 | 100 | 499 | 100 | d__Eukaryota;k__Alveolata;p__norank;c__Dinophyceae;o__Gymnodiniales;f__Gymnodiniaceae;g__Gymnodinium;s__Gymnodinium_instriatum |

| | | | | | | |
|---|---|---|---|---|---|---|
| OTU3298 | 512 | KF878935 | 90.66 | 514 | 100 | d__Eukaryota;k__Alveolata;p__norank;c__Dinophyceae;o__Gymnodiniales;f__Gymnodiniaceae;g__Gymnodinium;s__Gymnodinium_instriatum |
| OTU3300 | 496 | KF878935 | 96.57 | 496 | 100 | d__Eukaryota;k__Alveolata;p__norank;c__Dinophyceae;o__Gymnodiniales;f__Gymnodiniaceae;g__Gymnodinium;s__Gymnodinium_instriatum |
| OTU3301 | 499 | KF878935 | 97.98 | 496 | 99 | d__Eukaryota;k__Alveolata;p__norank;c__Dinophyceae;o__Gymnodiniales;f__Gymnodiniaceae;g__Gymnodinium;s__Gymnodinium_instriatum |
| OTU3303 | 491 | KF878935 | 96.14 | 492 | 100 | d__Eukaryota;k__Alveolata;p__norank;c__Dinophyceae;o__Gymnodiniales;f__Gymnodiniaceae;g__Gymnodinium;s__Gymnodinium_instriatum |
| OTU3305 | 489 | KF878935 | 94.31 | 492 | 100 | d__Eukaryota;k__Alveolata;p__norank;c__Dinophyceae;o__Gymnodiniales;f__Gymnodiniaceae;g__Gymnodinium;s__Gymnodinium_instriatum |
| OTU3306 | 493 | KF878935 | 96.75 | 493 | 100 | d__Eukaryota;k__Alveolata;p__norank;c__Dinophyceae;o__Gymnodiniales;f__Gymnodiniaceae;g__Gymnodinium;s__Gymnodinium_instriatum |
| OTU3307 | 491 | KF878935 | 96.96 | 493 | 100 | d__Eukaryota;k__Alveolata;p__norank;c__Dinophyceae;o__Gymnodiniales;f__Gymnodiniaceae;g__Gymnodinium;s__Gymnodinium_instriatum |
| OTU3309 | 460 | KF878935 | 96.52 | 460 | 100 | d__Eukaryota;k__Alveolata;p__norank;c__Dinophyceae;o__Gymnodiniales;f__Gymnodiniaceae;g__Gymnodinium;s__Gymnodinium_instriatum |
| OTU331 | 510 | AY571370 | 99.8 | 510 | 100 | d__Eukaryota;k__Alveolata;p__norank;c__Dinophyceae;o__Gymnodiniales;f__Gymnodiniaceae;g__Gyrodinium;s__Gyrodinium_dominans |
| OTU3316 | 509 | KF568378 | 86.6 | 97 | 19 | d__Eukaryota;k__Alveolata;p__norank;c__Dinophyceae;s__Unclaassified DINO |
| OTU332 | 505 | AY571371 | 99.21 | 505 | 100 | d__Eukaryota;k__Alveolata;p__norank;c__Dinophyceae;o__Gymnodiniales;f__Gymnodiniaceae;g__Gyrodinium;s__Gyrodinium_spirale |
| OTU3320 | 488 | KP702714 | 84.62 | 65 | 53 | d__Eukaryota;k__Alveolata;p__norank;c__Dinophyceae;o__Peridiniales;f__Diplopsaliaceae;g__Lebouraia;s__Unclassified DINO |
| OTU3328 | 499 | HM483396 | 92.99 | 499 | 100 | d__Eukaryota;k__Alveolata;p__norank;c__Dinophyceae;o__Peridiniales;f__Thoracosphaeraceae;g__Scrippsiella;s__Scrippsiella_trochoidea |
| OTU3330 | 499 | HM483396 | 92.35 | 497 | 99 | d__Eukaryota;k__Alveolata;p__norank;c__Dinophyceae;o__Peridiniales;f__Thoracosphaeraceae;g__Scrippsiella;s__Scrippsiella_trochoidea |
| OTU3331 | 505 | KF751924 | 99.4 | 502 | 99 | d__Eukaryota;k__Alveolata;p__norank;c__Dinophyceae;o__Peridiniales;f__Thoracosphaeraceae;g__Scrippsiella;s__Scrippsiella_aff._acuminata |
| OTU3332 | 503 | KF751924 | 96.63 | 504 | 100 | d__Eukaryota;k__Alveolata;p__norank;c__Dinophyceae;o__Peridiniales;f__Thoracosphaeraceae;g__Scrippsiella;s__Scrippsiella_aff._acuminata |
| OTU3333 | 515 | KF751924 | 90.72 | 517 | 100 | d__Eukaryota;k__Alveolata;p__norank;c__Dinophyceae;o__Peridiniales;f__Thoracosphaeraceae;g__Scrippsiella;s__Scrippsiella_aff._acuminata |
| OTU3335 | 519 | KF751924 | 89.21 | 519 | 99 | d__Eukaryota;k__Alveolata;p__norank;c__Dinophyceae;o__Peridiniales;f__Thoracosphaeraceae;g__Scrippsiella;s__Scrippsiella_aff._acuminata |
| OTU3336 | 537 | KF751924 | 85.92 | 547 | 100 | d__Eukaryota;k__Alveolata;p__norank;c__Dinophyceae;o__Peridiniales;f__Thoracosphaeraceae;g__Scrippsiella;s__Scrippsiella_aff._acuminata |
| OTU3338 | 496 | KF751924 | 96.57 | 496 | 100 | d__Eukaryota;k__Alveolata;p__norank;c__Dinophyceae;o__Peridiniales;f__Thoracosphaeraceae;g__Scrippsiella;s__Scrippsiella_aff._acuminata |
| OTU334 | 497 | AF260392 | 86.97 | 499 | 100 | d__Eukaryota;k__Alveolata;p__norank;c__Dinophyceae;o__Peridiniales;f__Thora |

| | | | | | | |
|---|---|---|---|---|---|---|
| | | | | | | cosphaeraceae;g__Scrippsiella;s__Scrippsiella_sp. |
| OTU3340 | 498 | AF260393 | 93.99 | 499 | 100 | d__Eukaryota;k__Alveolata;p__norank;c__Dinophyceae;o__Peridiniales;f__Thoracosphaeraceae;g__Scrippsiella;s__Scrippsiella_trochoidea |
| OTU3341 | 492 | KF751924 | 95.75 | 494 | 100 | d__Eukaryota;k__Alveolata;p__norank;c__Dinophyceae;o__Peridiniales;f__Thoracosphaeraceae;g__Scrippsiella;s__Scrippsiella_aff._acuminata |
| OTU3344 | 323 | KF751924 | 96.6 | 324 | 100 | d__Eukaryota;k__Alveolata;p__norank;c__Dinophyceae;o__Peridiniales;f__Thoracosphaeraceae;g__Scrippsiella;s__Scrippsiella_aff._acuminata |
| OTU3345 | 474 | KF751924 | 93.26 | 475 | 100 | d__Eukaryota;k__Alveolata;p__norank;c__Dinophyceae;o__Peridiniales;f__Thoracosphaeraceae;g__Scrippsiella;s__Scrippsiella_aff._acuminata |
| OTU3346 | 405 | KF751924 | 96.77 | 402 | 99 | d__Eukaryota;k__Alveolata;p__norank;c__Dinophyceae;o__Peridiniales;f__Thoracosphaeraceae;g__Scrippsiella;s__Scrippsiella_aff._acuminata |
| OTU3349 | 451 | KF751924 | 94.81 | 462 | 100 | d__Eukaryota;k__Alveolata;p__norank;c__Dinophyceae;o__Peridiniales;f__Thoracosphaeraceae;g__Scrippsiella;s__Scrippsiella_aff._acuminata |
| OTU3350 | 486 | KF751924 | 96.09 | 486 | 100 | d__Eukaryota;k__Alveolata;p__norank;c__Dinophyceae;o__Peridiniales;f__Thoracosphaeraceae;g__Scrippsiella;s__Scrippsiella_aff._acuminata |
| OTU3354 | 504 | KF751924 | 96.43 | 504 | 100 | d__Eukaryota;k__Alveolata;p__norank;c__Dinophyceae;o__Peridiniales;f__Thoracosphaeraceae;g__Scrippsiella;s__Scrippsiella_aff._acuminata |
| OTU3356 | 501 | KF751924 | 93.28 | 491 | 98 | d__Eukaryota;k__Alveolata;p__norank;c__Dinophyceae;o__Peridiniales;f__Thoracosphaeraceae;g__Scrippsiella;s__Scrippsiella_aff._acuminata |
| OTU3357 | 493 | KF751924 | 93.74 | 495 | 100 | d__Eukaryota;k__Alveolata;p__norank;c__Dinophyceae;o__Peridiniales;f__Thoracosphaeraceae;g__Scrippsiella;s__Scrippsiella_aff._acuminata |
| OTU3358 | 452 | KF751924 | 94.91 | 452 | 100 | d__Eukaryota;k__Alveolata;p__norank;c__Dinophyceae;o__Peridiniales;f__Thoracosphaeraceae;g__Scrippsiella;s__Scrippsiella_aff._acuminata |
| OTU3364 | 279 | KF751924 | 97.84 | 278 | 99 | d__Eukaryota;k__Alveolata;p__norank;c__Dinophyceae;o__Peridiniales;f__Thoracosphaeraceae;g__Scrippsiella;s__Scrippsiella_aff._acuminata |
| OTU3365 | 410 | KF751924 | 90.64 | 406 | 99 | d__Eukaryota;k__Alveolata;p__norank;c__Dinophyceae;o__Peridiniales;f__Thoracosphaeraceae;g__Scrippsiella;s__Scrippsiella_aff._acuminata |
| OTU3366 | 409 | KF751924 | 94.38 | 409 | 99 | d__Eukaryota;k__Alveolata;p__norank;c__Dinophyceae;o__Peridiniales;f__Thoracosphaeraceae;g__Scrippsiella;s__Scrippsiella_aff._acuminata |
| OTU3368 | 513 | KF751926 | 97.83 | 508 | 99 | d__Eukaryota;k__Alveolata;p__norank;c__Dinophyceae;o__Peridiniales;f__Thoracosphaeraceae;g__Scrippsiella;s__Scrippsiella_sp. |
| OTU337 | 497 | JX262498 | 89.16 | 498 | 100 | d__Eukaryota;k__Alveolata;p__norank;c__Dinophyceae;o__Peridiniales;f__Peridiniaceae;g__Pentapharsodinium;s__Pentapharsodinium_dalei |
| OTU3370 | 510 | KF751926 | 97.98 | 496 | 97 | d__Eukaryota;k__Alveolata;p__norank;c__Dinophyceae;o__Peridiniales;f__Thoracosphaeraceae;g__Scrippsiella;s__Scrippsiella_sp. |
| OTU3376 | 487 | KF751926 | 94.26 | 488 | 100 | d__Eukaryota;k__Alveolata;p__norank;c__Dinophyceae;o__Peridiniales;f__Thoracosphaeraceae;g__Scrippsiella;s__Scrippsiella_sp. |
| OTU3377 | 425 | KF751926 | 96.47 | 425 | 100 | d__Eukaryota;k__Alveolata;p__norank;c__Dinophyceae;o__Peridiniales;f__Thoracosphaeraceae;g__Scrippsiella;s__Scrippsiella_sp. |
| OTU3379 | 502 | KF751926 | 95.59 | 521 | 100 | d__Eukaryota;k__Alveolata;p__norank;c__Dinophyceae;o__Peridiniales;f__Thora |

| | | | | | | |
|---|---|---|---|---|---|---|
| | | | | | | cosphaeraceae;g__Scrippsiella;s__Scrippsiella_sp. |
| OTU338 | 492 | AY571374 | 85.25 | 488 | 97 | d__Eukaryota;k__Alveolata;p__norank;c__Dinophyceae;o__Lophodiniales;f__Lophodiniaceae;g__Woloszynskia;s__Woloszynskia_tenuissima |
| OTU3385 | 495 | HM483396 | 94.34 | 495 | 100 | d__Eukaryota;k__Alveolata;p__norank;c__Dinophyceae;o__Peridiniales;f__Thoracosphaeraceae;g__Scrippsiella;s__Scrippsiella_trochoidea |
| OTU3387 | 493 | KF751926 | 91.06 | 492 | 99 | d__Eukaryota;k__Alveolata;p__norank;c__Dinophyceae;o__Peridiniales;f__Thoracosphaeraceae;g__Scrippsiella;s__Scrippsiella_sp. |
| OTU3388 | 485 | KF751926 | 93.75 | 496 | 100 | d__Eukaryota;k__Alveolata;p__norank;c__Dinophyceae;o__Peridiniales;f__Thoracosphaeraceae;g__Scrippsiella;s__Scrippsiella_sp. |
| OTU339 | 470 | AY571374 | 100 | 470 | 100 | d__Eukaryota;k__Alveolata;p__norank;c__Dinophyceae;o__Lophodiniales;f__Lophodiniaceae;g__Woloszynskia;s__Woloszynskia_tenuissima |
| OTU3390 | 459 | KF751926 | 93.08 | 491 | 100 | d__Eukaryota;k__Alveolata;p__norank;c__Dinophyceae;o__Peridiniales;f__Thoracosphaeraceae;g__Scrippsiella;s__Scrippsiella_sp. |
| OTU3391 | 423 | KF751926 | 95.72 | 421 | 99 | d__Eukaryota;k__Alveolata;p__norank;c__Dinophyceae;o__Peridiniales;f__Thoracosphaeraceae;g__Scrippsiella;s__Scrippsiella_sp. |
| OTU3394 | 510 | AB871535 | 96.86 | 509 | 99 | d__Eukaryota;k__Alveolata;p__norank;c__Dinophyceae;o__Gonyaulacales;f__Crypthecodiniaceae;g__Crypthecodinium;s__Crypthecodinium_sp._ShSu-2013 |
| OTU3399 | 495 | AB858353 | 99.53 | 424 | 85 | d__Eukaryota;k__Alveolata;p__norank;c__Dinophyceae;o__Suessiales;f__Biecheleriaceae;g__Biecheleria;s__Biecheleria_brevisulcata |
| OTU340 | 490 | AY571374 | 84.71 | 484 | 98 | d__Eukaryota;k__Alveolata;p__norank;c__Dinophyceae;o__Lophodiniales;f__Lophodiniaceae;g__Woloszynskia;s__Woloszynskia_tenuissima |
| OTU3400 | 498 | AB858353 | 95.38 | 498 | 100 | d__Eukaryota;k__Alveolata;p__norank;c__Dinophyceae;o__Suessiales;f__Biecheleriaceae;g__Biecheleria;s__Biecheleria_brevisulcata |
| OTU3401 | 498 | AB858353 | 95.21 | 501 | 100 | d__Eukaryota;k__Alveolata;p__norank;c__Dinophyceae;o__Suessiales;f__Biecheleriaceae;g__Biecheleria;s__Biecheleria_brevisulcata |
| OTU3402 | 496 | AB858353 | 92.96 | 497 | 100 | d__Eukaryota;k__Alveolata;p__norank;c__Dinophyceae;o__Suessiales;f__Biecheleriaceae;g__Biecheleria;s__Biecheleria_brevisulcata |
| OTU3404 | 494 | AB858353 | 98.38 | 494 | 100 | d__Eukaryota;k__Alveolata;p__norank;c__Dinophyceae;o__Suessiales;f__Biecheleriaceae;g__Biecheleria;s__Biecheleria_brevisulcata |
| OTU3405 | 497 | AB858353 | 95.38 | 498 | 100 | d__Eukaryota;k__Alveolata;p__norank;c__Dinophyceae;o__Suessiales;f__Biecheleriaceae;g__Biecheleria;s__Biecheleria_brevisulcata |
| OTU3406 | 515 | AB858353 | 94.69 | 508 | 99 | d__Eukaryota;k__Alveolata;p__norank;c__Dinophyceae;o__Suessiales;f__Biecheleriaceae;g__Biecheleria;s__Biecheleria_brevisulcata |
| OTU3407 | 501 | AB858353 | 88.42 | 501 | 100 | d__Eukaryota;k__Alveolata;p__norank;c__Dinophyceae;o__Suessiales;f__Biecheleriaceae;g__Biecheleria;s__Biecheleria_brevisulcata |
| OTU3408 | 225 | AB858353 | 99.06 | 213 | 94 | d__Eukaryota;k__Alveolata;p__norank;c__Dinophyceae;o__Suessiales;f__Biecheleriaceae;g__Biecheleria;s__Biecheleria_brevisulcata |
| OTU3409 | 497 | AB858353 | 94.57 | 497 | 100 | d__Eukaryota;k__Alveolata;p__norank;c__Dinophyceae;o__Suessiales;f__Biecheleriaceae;g__Biecheleria;s__Biecheleria_brevisulcata |
| OTU341 | 488 | AY571374 | 83.88 | 490 | 99 | d__Eukaryota;k__Alveolata;p__norank;c__Dinophyceae;o__Lophodiniales;f__Lop |

| | | | | | | |
|---|---|---|---|---|---|---|
| | | | | | | hodiniaceae;g__Woloszynskia;s__Woloszynskia_tenuissima |
| OTU3410 | 264 | AB858353 | 97.73 | 264 | 100 | d__Eukaryota;k__Alveolata;p__norank;c__Dinophyceae;o__Suessiales;f__Biecheleriaceae;g__Biecheleria;s__Biecheleria_brevisulcata |
| OTU3412 | 222 | AB858353 | 99.52 | 210 | 94 | d__Eukaryota;k__Alveolata;p__norank;c__Dinophyceae;o__Suessiales;f__Biecheleriaceae;g__Biecheleria;s__Biecheleria_brevisulcata |
| OTU3413 | 495 | AB858353 | 89.54 | 497 | 100 | d__Eukaryota;k__Alveolata;p__norank;c__Dinophyceae;o__Suessiales;f__Biecheleriaceae;g__Biecheleria;s__Biecheleria_brevisulcata |
| OTU3414 | 505 | AB858353 | 98.35 | 303 | 66 | d__Eukaryota;k__Alveolata;p__norank;c__Dinophyceae;o__Suessiales;f__Biecheleriaceae;g__Biecheleria;s__Biecheleria-like_sp. |
| OTU3415 | 488 | AB858353 | 96.93 | 489 | 100 | d__Eukaryota;k__Alveolata;p__norank;c__Dinophyceae;o__Suessiales;f__Biecheleriaceae;g__Biecheleria;s__Biecheleria_brevisulcata |
| OTU3418 | 454 | AB858353 | 92.97 | 455 | 100 | d__Eukaryota;k__Alveolata;p__norank;c__Dinophyceae;o__Suessiales;f__Biecheleriaceae;g__Biecheleria;s__Biecheleria_brevisulcata |
| OTU342 | 481 | AY571374 | 99.5 | 398 | 83 | d__Eukaryota;k__Alveolata;p__norank;c__Dinophyceae;o__Lophodiniales;f__Lophodiniaceae;g__Woloszynskia;s__Woloszynskia_tenuissima-like |
| OTU3420 | 351 | AB858353 | 96.85 | 349 | 99 | d__Eukaryota;k__Alveolata;p__norank;c__Dinophyceae;o__Suessiales;f__Biecheleriaceae;g__Biecheleria;s__Biecheleria_brevisulcata |
| OTU3423 | 331 | AB858353 | 96.07 | 331 | 100 | d__Eukaryota;k__Alveolata;p__norank;c__Dinophyceae;o__Suessiales;f__Biecheleriaceae;g__Biecheleria;s__Biecheleria_brevisulcata |
| OTU3424 | 488 | AB858353 | 95.09 | 489 | 100 | d__Eukaryota;k__Alveolata;p__norank;c__Dinophyceae;o__Suessiales;f__Biecheleriaceae;g__Biecheleria;s__Biecheleria_brevisulcata |
| OTU3426 | 496 | AB858353 | 93.75 | 496 | 100 | d__Eukaryota;k__Alveolata;p__norank;c__Dinophyceae;o__Suessiales;f__Biecheleriaceae;g__Biecheleria;s__Biecheleria_brevisulcata |
| OTU3427 | 502 | AB858353 | 94.62 | 502 | 100 | d__Eukaryota;k__Alveolata;p__norank;c__Dinophyceae;o__Suessiales;f__Biecheleriaceae;g__Biecheleria;s__Biecheleria_brevisulcata |
| OTU3428 | 463 | AB858353 | 91.81 | 464 | 100 | d__Eukaryota;k__Alveolata;p__norank;c__Dinophyceae;o__Suessiales;f__Biecheleriaceae;g__Biecheleria;s__Biecheleria_brevisulcata |
| OTU3429 | 478 | LM992906 | 99.79 | 478 | 100 | d__Eukaryota;k__Alveolata;p__norank;c__Dinophyceae;o__Lophodiniales;f__Lophodiniaceae;g__Biecheleriopsis;s__Biecheleriopsis_adriatica |
| OTU343 | 476 | AY571374 | 99.52 | 416 | 87 | d__Eukaryota;k__Alveolata;p__norank;c__Dinophyceae;o__Lophodiniales;f__Lophodiniaceae;g__Woloszynskia;s__Woloszynskia_tenuissima-like |
| OTU3430 | 494 | LM992906 | 94.13 | 494 | 100 | d__Eukaryota;k__Alveolata;p__norank;c__Dinophyceae;o__Lophodiniales;f__Lophodiniaceae;g__Biecheleriopsis;s__Biecheleriopsis_adriatica |
| OTU3431 | 498 | LM992906 | 93.96 | 497 | 99 | d__Eukaryota;k__Alveolata;p__norank;c__Dinophyceae;o__Lophodiniales;f__Lophodiniaceae;g__Biecheleriopsis;s__Biecheleriopsis_adriatica |
| OTU3434 | 484 | LM992906 | 92.52 | 481 | 99 | d__Eukaryota;k__Alveolata;p__norank;c__Dinophyceae;o__Lophodiniales;f__Lophodiniaceae;g__Biecheleriopsis;s__Biecheleriopsis_adriatica |
| OTU344 | 477 | AY571374 | 96.44 | 477 | 100 | d__Eukaryota;k__Alveolata;p__norank;c__Dinophyceae;o__Lophodiniales;f__Lophodiniaceae;g__Woloszynskia;s__Woloszynskia_tenuissima |
| OTU3443 | 489 | LM992906 | 91.46 | 492 | 100 | d__Eukaryota;k__Alveolata;p__norank;c__Dinophyceae;o__Lophodiniales;f__Lop |

| | | | | | | |
|---|---|---|---|---|---|---|
| | | | | | | hodiniaceae;g__Biecheleriopsis;s__Biecheleriopsis_adriatica |
| OTU3445 | 511 | LC068838.1 | 88 | 506 | 98 | d__Eukaryota;k__Alveolata;p__norank;c__Dinophyceae;o__Suessiales; f__Suessiaceae;g__norank;s__Unclassified Suessiaceae |
| OTU3446 | 485 | LM992905 | 100 | 339 | 70 | d__Eukaryota;k__Alveolata;p__norank;c__Dinophyceae;o__Lophodiniales;f__Lophodiniaceae;g__Biecheleriopsis;s__Biecheleriopsis_adriatica |
| OTU3447 | 275 | LM992906 | 81.58 | 152 | 99 | d__Eukaryota;k__Alveolata;p__norank;c__Dinophyceae;o__Lophodiniales;f__Lophodiniaceae;g__Biecheleriopsis;s__Biecheleriopsis_sp. |
| OTU3448 | 441 | KF646451 | 98.19 | 441 | 100 | d__Eukaryota;k__Alveolata;p__norank;c__Dinophyceae;o__Gonyaulacales;f__Gonyaulacaceae;g__Alexandrium;s__Alexandrium_tamarense_species_complex_group_IV |
| OTU3449 | 499 | KJ879232 | 100 | 499 | 100 | d__Eukaryota;k__Alveolata;p__norank;c__Dinophyceae;o__Gonyaulacales;f__Gonyaulacaceae;g__Alexandrium;s__Alexandrium_catenella |
| OTU345 | 489 | AY571374 | 96.93 | 489 | 100 | d__Eukaryota;k__Alveolata;p__norank;c__Dinophyceae;o__Lophodiniales;f__Lophodiniaceae;g__Woloszynskia;s__Woloszynskia_tenuissima |
| OTU3451 | 511 | KF998563 | 91.67 | 48 | 27 | d__Eukaryota;k__Alveolata;p__norank;c__Dinophyceae;o__Gymnodiniales;f__Kareniaceae;g__Karenia;s__Unclassified DINO |
| OTU3452 | 541 | LC002848 | 87.36 | 87 | 43 | d__Eukaryota;k__Alveolata;p__norank;c__Dinophyceae;o__unclassified_Dinophyceae;f__norank;g__Pseudadenoides;s__Unclassified DINO |
| OTU346 | 482 | AY571374 | 96.69 | 483 | 100 | d__Eukaryota;k__Alveolata;p__norank;c__Dinophyceae;o__Lophodiniales;f__Lophodiniaceae;g__Woloszynskia;s__Woloszynskia_tenuissima |
| OTU3463 | 489 | AB860180 | 87.7 | 496 | 100 | d__Eukaryota;k__Alveolata;p__norank;c__Dinophyceae;o__Gymnodiniales;f__Gymnodiniaceae;g__Gymnodinium;s__unclassified_Gymnodinium |
| OTU3467 | 490 | HG005135 | 94.29 | 490 | 100 | d__Eukaryota;k__Alveolata;p__norank;c__Dinophyceae;o__Gymnodiniales;f__Gymnodiniaceae;g__Gymnodinium;s__unclassified_Gymnodinium |
| OTU347 | 487 | AY571374 | 82.92 | 486 | 98 | d__Eukaryota;k__Alveolata;p__norank;c__Dinophyceae;o__Lophodiniales;f__Lophodiniaceae;g__Woloszynskia;s__Woloszynskia_like |
| OTU348 | 471 | AY571374 | 85.2 | 473 | 100 | d__Eukaryota;k__Alveolata;p__norank;c__Dinophyceae;o__Lophodiniales;f__Lophodiniaceae;g__Woloszynskia;s__Woloszynskia_tenuissima |
| OTU349 | 485 | AY571374 | 95.71 | 489 | 100 | d__Eukaryota;k__Alveolata;p__norank;c__Dinophyceae;o__Lophodiniales;f__Lophodiniaceae;g__Woloszynskia;s__Woloszynskia_tenuissima |
| OTU3490 | 501 | HG005135 | 92.38 | 499 | 99 | d__Eukaryota;k__Alveolata;p__norank;c__Dinophyceae;o__Gymnodiniales;f__Gymnodiniaceae;g__Gymnodinium;s__unclassified_Gymnodinium |
| OTU35 | 496 | AF260392 | 89.11 | 496 | 100 | d__Eukaryota;k__Alveolata;p__norank;c__Dinophyceae;o__Peridiniales;f__Thoracosphaeraceae;g__Scrippsiella;s__Scrippsiella_sp. |
| OTU3508 | 470 | AB860180 | 84.07 | 477 | 100 | d__Eukaryota;k__Alveolata;p__norank;c__Dinophyceae;o__Gymnodiniales;f__Gymnodiniaceae;g__Gymnodinium;s__unclassified_Gymnodinium |
| OTU3509 | 483 | HG005135 | 99.17 | 483 | 100 | d__Eukaryota;k__Alveolata;p__norank;c__Dinophyceae;o__Gymnodiniales;f__Gymnodiniaceae;g__Gymnodinium;s__Gymnodinium_sp. GSSW10 |
| OTU351 | 488 | AY571374 | 96.29 | 485 | 99 | d__Eukaryota;k__Alveolata;p__norank;c__Dinophyceae;o__Lophodiniales;f__Lophodiniaceae;g__Woloszynskia;s__Woloszynskia_tenuissima |

| | | | | | | |
|---|---|---|---|---|---|---|
| OTU3512 | 484 | AB860180 | 84.88 | 496 | 100 | d__Eukaryota;k__Alveolata;p__norank;c__Dinophyceae;o__Gymnodiniales;f__Gymnodiniaceae;g__Gymnodinium;s__unclassified_Gymnodinium |
| OTU352 | 447 | AY571374 | 96.02 | 452 | 100 | d__Eukaryota;k__Alveolata;p__norank;c__Dinophyceae;o__Lophodiniales;f__Lophodiniaceae;g__Woloszynskia;s__Woloszynskia_tenuissima |
| OTU3527 | 483 | KF646481 | 99.79 | 484 | 100 | d__Eukaryota;k__Alveolata;p__norank;c__Dinophyceae;o__Gonyaulacales;f__Gonyaulacaceae;g__Alexandrium;s__Alexandrium_tamarense_species_complex_group_I |
| OTU353 | 391 | AY571374 | 94.64 | 392 | 99 | d__Eukaryota;k__Alveolata;p__norank;c__Dinophyceae;o__Lophodiniales;f__Lophodiniaceae;g__Woloszynskia;s__Woloszynskia_tenuissima |
| OTU3530 | 497 | KJ433986 | 87.62 | 501 | 99 | d__Eukaryota;k__Alveolata;p__norank;c__Dinophyceae;o__Peridiniales;f__Peridiniaceae;g__Pentapharsodinium;s__Pentapharsodinium_sp._ZL-2014 |
| OTU3535 | 437 | KJ433986 | 90.13 | 476 | 100 | d__Eukaryota;k__Alveolata;p__norank;c__Dinophyceae;o__Peridiniales;f__Peridiniaceae;g__Pentapharsodinium;s__Pentapharsodinium_sp._ZL-2014 |
| OTU3538 | 466 | KJ433986 | 90.79 | 467 | 100 | d__Eukaryota;k__Alveolata;p__norank;c__Dinophyceae;o__Peridiniales;f__Peridiniaceae;g__Pentapharsodinium;s__Pentapharsodinium_sp._ZL-2014 |
| OTU355 | 505 | DQ991376 | 95.86 | 507 | 100 | d__Eukaryota;k__Alveolata;p__norank;c__Dinophyceae;o__Peridiniales;f__Pfiesteriaceae;g__Cryptoperidiniopsis;s__Cryptoperidiniopsis_brodyi |
| OTU356 | 507 | AY590476 | 99.6 | 501 | 99 | d__Eukaryota;k__Alveolata;p__norank;c__Dinophyceae;o__Peridiniales;f__Pfiesteriaceae;g__Cryptoperidiniopsis;s__Cryptoperidiniopsis_brodyi |
| OTU357 | 328 | AY590477 | 98.79 | 330 | 100 | d__Eukaryota;k__Alveolata;p__norank;c__Dinophyceae;o__Peridiniales;f__Pfiesteriaceae;g__unclassified_Pfiesteriaceae;s__Pfiesteriaceae_sp._CCMP1835 |
| OTU3587 | 228 | HG005132 | 88.89 | 198 | 87 | d__Eukaryota;k__Alveolata;p__norank;c__Dinophyceae;o__unclassified_Dinophyceae;f__norank;g__Stoeckeria;s__Stoeckeria_sp._SSSC09 |
| OTU3588 | 222 | HG005132 | 89.39 | 198 | 89 | d__Eukaryota;k__Alveolata;p__norank;c__Dinophyceae;o__unclassified_Dinophyceae;f__norank;g__Stoeckeria;s__Stoeckeria_sp._SSSC09 |
| OTU3589 | 416 | KJ450988 | 91.15 | 418 | 100 | d__Eukaryota;k__Alveolata;p__norank;c__Dinophyceae;o__Peridiniales;f__Peridiniaceae;g__Peridinium;s__Peridinium_euryceps |
| OTU3593 | 516 | AB716928 | 89.36 | 517 | 100 | d__Eukaryota;k__Alveolata;p__norank;c__Dinophyceae;o__Peridiniales;f__Protoperidiniaceae;g__Protoperidinium;s__Protoperidinium_monovelum |
| OTU3602 | 486 | KP790206 | 97.24 | 434 | 89 | d__Eukaryota;k__Alveolata;p__norank;c__Dinophyceae;o__Gymnodiniales;f__Gymnodiniaceae;g__Gyrodinium;s__Gyrodinium_undulans |
| OTU361 | 413 | LC027052 | 76.64 | 244 | 58 | d__Eukaryota;k__Alveolata;p__norank;c__Dinophyceae;o__Gymnodiniales;f__Gymnodiniaceae;g__Unclassified;s__Unclassified_Gymnodiniaceae |
| OTU3612 | 499 | KJ481814 | 90.2 | 500 | 100 | d__Eukaryota;k__Alveolata;p__norank;c__Dinophyceae;o__Gonyaulacales;f__Amphidomataceae;g__Azadinium;s__Azadinium_trinitatum |
| OTU3616 | 477 | JX262498 | 77.78 | 477 | 100 | d__Eukaryota;k__Alveolata;p__norank;c__Dinophyceae;o__Peridiniales;f__Peridiniaceae;g__Pentapharsodinium;s__Pentapharsodinium_dalei |
| OTU3617 | 476 | KJ433986 | 77.87 | 479 | 100 | d__Eukaryota;k__Alveolata;p__norank;c__Dinophyceae;o__Peridiniales;f__Peridiniaceae;g__Pentapharsodinium;s__Pentapharsodinium_sp._ZL-2014 |
| OTU3624 | 506 | AY284949 | 73 | 506 | 100 | d__Eukaryota;k__Alveolata;p__norank;c__Dinophyceae;o__Gymnodiniales;f__Ka |

| | | | | | | |
|---|---|---|---|---|---|---|
| | | | | | | reniaceae;g__Takayama; s__Takayama_sp. |
| OTU3639 | 510 | JX262498 | 85 | 510 | 100 | d__Eukaryota;k__Alveolata;p__norank;c__Dinophyceae;o__Peridiniales; f__Peridiniaceae; g__Pentapharsodinium; s__Pentapharsodinium_sp. |
| OTU365 | 504 | AY863008 | 99.4 | 504 | 100 | d__Eukaryota;k__Alveolata;p__norank;c__Dinophyceae;o__Prorocentrales;f__Prorocentraceae;g__Prorocentrum;s__Prorocentrum_micans |
| OTU367 | 484 | AY916538 | 97.49 | 479 | 99 | d__Eukaryota;k__Alveolata;p__norank;c__Dinophyceae;o__Gymnodiniales;f__Gymnodiniaceae;g__Gymnodinium;s__Gymnodinium_microreticulatum |
| OTU3686 | 495 | JX262498 | 75 | 356 | 72 | d__Eukaryota;k__Alveolata;p__norank;c__Dinophyceae;o__Peridiniales; f__Peridiniaceae;g__Pentapharsodinium;s__Pentapharsodinium_sp. |
| OTU3689 | 262 | FJ808690 | 80 | 200 | 75 | d__Eukaryota;k__Alveolata;p__norank;c__Dinophyceae;o__Dinophysiales;f__Dinophysiaceae;g__Dinophysis;s__Dinophysis_sp. |
| OTU369 | 482 | AY916538 | 98.74 | 476 | 99 | d__Eukaryota;k__Alveolata;p__norank;c__Dinophyceae;o__Gymnodiniales;f__Gymnodiniaceae;g__Gymnodinium;s__Gymnodinium_microreticulatum |
| OTU3690 | 384 | KJ433986 | 74 | 384 | 100 | d__Eukaryota;k__Alveolata;p__norank;c__Dinophyceae;o__Peridiniales; f__Peridiniaceae; g__Pentapharsodinium; s__Pentapharsodinium_sp. |
| OTU3691 | 334 | FJ808690 | 78.05 | 205 | 60 | d__Eukaryota;k__Alveolata;p__norank;c__Dinophyceae;o__Dinophysiales;f__Dinophysiaceae;g__Dinophysis;s__Dinophysis_schuettii |
| OTU3692 | 317 | KP790210 | 92.86 | 112 | 35 | d__Eukaryota;k__Alveolata;p__norank;c__Dinophyceae;o__Gymnodiniales;f__Gymnodiniaceae;g__Gyrodinium;s__Gyrodinium_sp. |
| OTU3693 | 403 | KJ433986 | 74 | 113 | 99 | d__Eukaryota;k__Alveolata;p__norank;c__Dinophyceae;o__Peridiniales; f__Peridiniaceae; g__Pentapharsodinium; s__Pentapharsodinium_sp. |
| OTU3694 | 459 | JX262498 | 75 | 66 | 85 | d__Eukaryota;k__Alveolata;p__norank;c__Dinophyceae;o__Peridiniales; f__Peridiniaceae; g__Pentapharsodinium; s__Pentapharsodinium_sp. |
| OTU3695 | 360 | KT389956.1 | 81 | 356 | 97 | d__Eukaryota;k__Alveolata;p__norank;c__Dinophyceae;o__Peridiniales;f__Thoracosphaeraceae;g__Scrippsiella;s__Scrippsiella_sp. |
| OTU3696 | 507 | EU490149 | 73.41 | 504 | 96 | d__Eukaryota;k__Fungi;p__environmental_samples;c__Dinophyceae;o__norank;f__norank;g__norank;s__unclassified DINO |
| OTU3697 | 494 | EU490149 | 73.02 | 504 | 99 | d__Eukaryota;k__Fungi;p__environmental_samples;c__Dinophyceae;o__norank;f__norank;g__norank;s__unclassified DINO |
| OTU3698 | 511 | KF751922 | 95.35 | 86 | 53 | d__Eukaryota;k__Alveolata;p__norank;c__Dinophyceae;o__Peridiniales;f__Thoracosphaeraceae;g__Calciodinellum;s__Calciodinellum_operosum |
| OTU3699 | 506 | EU490149 | 71.49 | 505 | 97 | d__Eukaryota;k__Fungi;p__environmental_samples;c__Dinophyceae;o__norank;f__norank;g__norank;s__unclassified DINO |
| OTU3701 | 511 | HQ176320 | 87.74 | 106 | 57 | d__Eukaryota;k__Alveolata;p__norank;c__Dinophyceae;o__Peridiniales;f__Pfiesteriaceae;g__Chimonodinium;s__Chimonodinium_like |
| OTU3702 | 495 | HQ176320 | 84 | 507 | 55 | d__Eukaryota;k__Fungi;p__environmental_samples;c__Dinophyceae;o__norank;f__norank;g__norank;s__unclassified DINO |
| OTU3704 | 522 | HM483397 | 85.32 | 88 | 54 | d__Eukaryota;k__Alveolata;p__norank;c__Dinophyceae;o__unclassified_Dinophyceae;f__norank;g__Tintinnophagus;s__unclassified DINO |
| OTU3705 | 516 | FN557541 | 85.38 | 89 | 54 | d__Eukaryota;k__Alveolata;p__norank;c__Dinophyceae;o__unclassified_Dinoph |

| | | | | | | |
|---|---|---|---|---|---|---|
| | | | | | | yceae;f__norank;g__Stoeckeria;s__Stoeckeria_sp._SSMS0806 |
| OTU3706 | 519 | HE611580 | 96.51 | 86 | 54 | d__Eukaryota;k__Alveolata;p__norank;c__Dinophyceae;o__Gymnodiniales;f__Gymnodiniaceae;g__Gyrodinium;s__Gyrodinium_sp._HJ-2011 |
| OTU3708 | 523 | HM483395 | 73.16 | 529 | 99 | d__Eukaryota;k__Alveolata;p__norank;c__Dinophyceae;o__Syndiniales;f__Amoebophryaceae;g__Amoebophrya;s__Amoebophrya_sp._ex_Akashiwo_sanguineaum |
| OTU371 | 493 | AY916539 | 90.2 | 490 | 99 | d__Eukaryota;k__Alveolata;p__norank;c__Dinophyceae;o__Gymnodiniales;f__Gymnodiniaceae;g__Gymnodinium;s__Gymnodinium_microreticulatum |
| OTU3710 | 507 | EU490149 | 72.34 | 499 | 96 | d__Eukaryota;k__Alveolata;p__norank;;c__Dinophyceae;o__norank;f__norank;g__norank;s__unclassified DINO |
| OTU3711 | 514 | EU490149 | 72.58 | 507 | 96 | d__Eukaryota;k__Alveolata;p__norank;c__Dinophyceae;o__norank;f__norank;g__norank;s__unclassified DINO |
| OTU3712 | 494 | EU490149 | 72.55 | 499 | 98 | d__Eukaryota;k__Alveolata;p__norank; c__Dinophyceae;o__norank;f__norank;g__norank;s__unclassified DINO |
| OTU3717 | 543 | KF835600 | 90.18 | 112 | 21 | d__Eukaryota;k__Alveolata;p__norank;c__Dinophyceae;o__Prorocentrales;f__Prorocentraceae;g__Prorocentrum;s__unclassified DINO |
| OTU3720 | 479 | HG792066 | 100 | 479 | 100 | d__Eukaryota;k__Alveolata;p__norank;c__Dinophyceae;o__unclassified_Dinophyceae;f__norank;g__norank;s__Dinophyceae_sp._1_HJH-2013 |
| OTU3721 | 505 | LM992906 | 91 | 89 | 72 | d__Eukaryota;k__Alveolata;p__norank;c__Dinophyceae;o__Suessiales;f__Biecheleriaceae;g__norank;s__Biecheleriopsis-like species |
| OTU3722 | 493 | AF260402 | 81 | 467 | 100 | d__Eukaryota;k__Alveolata;p__norank;c__Dinophyceae;o__Lophodiniales;f__Lophodiniaceae;g__Woloszynskia;s__Woloszynskia_sp. |
| OTU3724 | 492 | AF260402 | 81.51 | 465 | 100 | d__Eukaryota;k__Alveolata;p__norank;c__Dinophyceae;o__Lophodiniales;f__Lophodiniaceae;g__Woloszynskia;s__Woloszynskia-like_sp. |
| OTU3726 | 475 | HG792066 | 96.84 | 475 | 100 | d__Eukaryota;k__Alveolata;p__norank;c__Dinophyceae;o__unclassified_Dinophyceae;f__norank;g__norank;s__Dinophyceae_sp._1_HJH-2013 |
| OTU373 | 485 | AY916539 | 89 | 482 | 99 | d__Eukaryota;k__Alveolata;p__norank;c__Dinophyceae;o__Gymnodiniales;f__Gymnodiniaceae;g__Gymnodinium;s__Gymnodinium_microreticulatum |
| OTU3730 | 475 | HG792066 | 91.01 | 89 | 71 | d__Eukaryota;k__Alveolata;p__norank;c__Dinophyceae;o__unclassified_Dinophyceae;f__norank;g__norank;s__Dinophyceae_sp._1_HJH-2013 |
| OTU3731 | 469 | HG792066 | 94.38 | 89 | 74 | d__Eukaryota;k__Alveolata;p__norank;c__Dinophyceae;o__unclassified_Dinophyceae;f__norank;g__norank;s__Dinophyceae_sp._1_HJH-2013 |
| OTU3733 | 265 | AB858353 | 86.36 | 132 | 50 | d__Eukaryota;k__Alveolata;p__norank;c__Dinophyceae;o__Suessiales;f__Biecheleriaceae;g__Biecheleria;s__unclassified DINO |
| OTU3735 | 406 | LC068838.1 | 85 | 406 | 100 | d__Eukaryota;k__Alveolata;p__norank;c__Dinophyceae;o__Suessiales;f__Biecheleriaceae;g__norank;s__Unclassified Suessiales |
| OTU3736 | 287 | LC068838.1 | 83.19 | 282 | 98 | d__Eukaryota;k__Alveolata;p__norank;c__Dinophyceae;o__Suessiales;f__Biecheleriaceae;g__norank;s__Unclassified Suessiales |
| OTU3737 | 241 | AB858353 | 85.61 | 132 | 55 | d__Eukaryota;k__Alveolata;p__norank;c__Dinophyceae;o__Suessiales;f__Biecheleriaceae;g__norank;s__Unclassified Suessiales |

| | | | | | | |
|---|---|---|---|---|---|---|
| OTU3738 | 402 | JQ413374 | 77.34 | 406 | 100 | d__Eukaryota;k__Alveolata;p__norank;c__Dinophyceae;o__Suessiales;f__Biecheleriaceae;g__Biecheleria;s__Biecheleria_cincta |
| OTU3741 | 389 | AY916553 | 77.06 | 388 | 98 | d__Eukaryota;k__Alveolata;p__norank;c__Dinophyceae;o__Lophodiniales;f__Lophodiniaceae;g__Woloszynskia;s__Woloszynskia_sp._CS-341 |
| OTU3744 | 531 | LM049720 | 85.71 | 63 | 27 | d__Eukaryota;k__Alveolata;p__norank;c__Dinophyceae;o__norank;f__norank;g__norank;s__unclassified DINO |
| OTU3757 | 480 | LC027049 | 98.34 | 481 | 100 | d__Eukaryota;k__Alveolata;p__norank;c__Dinophyceae;o__unclassified_Dinophyceae;f__norank;g__Pellucidodinium;s__Pellucidodinium_psammophilum |
| OTU3759 | 478 | LC027049 | 89.77 | 479 | 99 | d__Eukaryota;k__Alveolata;p__norank;c__Dinophyceae;o__unclassified_Dinophyceae;f__norank;g__Pellucidodinium;s__Pellucidodinium_psammophilum |
| OTU376 | 505 | AY916546 | 99.8 | 499 | 99 | d__Eukaryota;k__Alveolata;p__norank;c__Dinophyceae;o__Peridiniales;f__Thoracosphaeraceae;g__Scrippsiella;s__Scrippsiella_sp._CS297 |
| OTU377 | 514 | AY916546 | 87.15 | 506 | 98 | d__Eukaryota;k__Alveolata;p__norank;c__Dinophyceae;o__Peridiniales;f__Thoracosphaeraceae;g__Scrippsiella;s__Scrippsiella_sp._CS297 |
| OTU3779 | 492 | JX262498 | 75 | 190 | 79 | d__Eukaryota;k__Alveolata;p__norank;c__Dinophyceae;o__Peridiniales; f__Peridiniaceae; g__Pentapharsodinium; s__Pentapharsodinium_sp. |
| OTU3787 | 538 | AB473665 | 97 | 538 | 73 | d__Eukaryota;k__Alveolata;p__norank;c__Dinophyceae; o__Dinophysiales; f__Dinophysiaceae; g__Dinophysis;s__Dinophysis_lativelata |
| OTU3788 | 490 | KJ508397 | 95.74 | 493 | 100 | d__Eukaryota;k__Alveolata;p__norank;c__Dinophyceae;o__Gymnodiniales;f__Warnowiaceae;g__Warnowia;s__Warnowia_sp._IFR1101 |
| OTU379 | 492 | AY916546 | 95.9 | 488 | 99 | d__Eukaryota;k__Alveolata;p__norank;c__Dinophyceae;o__Peridiniales;f__Thoracosphaeraceae;g__Scrippsiella;s__Scrippsiella_sp._CS297 |
| OTU3790 | 506 | FJ032669 | 88 | 460 | 100 | d__Eukaryota;k__Alveolata;p__norank;c__Dinophyceae;o__Dinophysiales;f__ Dinophysiaceae;g__Dinophysis;s__Dinophysis_sp. |
| OTU3795 | 510 | KJ508377 | 79 | 520 | 99 | d__Eukaryota;k__Alveolata;p__norank;c__Dinophyceae;o__Gymnodiniales;f__Kareniaceae;g__Karlodinium;s__Karlodinium_sp. |
| OTU3797 | 528 | JX262498 | 76 | 109 | 100 | d__Eukaryota;k__Alveolata;p__norank;c__Dinophyceae;o__Peridiniales;f__Peridiniaceae;g__Pentapharsodinium;s__Pentapharsodinium_sp. |
| OTU3798 | 506 | KJ450990 | 94.43 | 503 | 99 | d__Eukaryota;k__Alveolata;p__norank;c__Dinophyceae;o__Peridiniales;f__Peridiniaceae;g__Peridinium;s__Peridinium_aciculiferum |
| OTU3804 | 497 | AF260400 | 71.6 | 507 | 100 | d__Eukaryota;k__Alveolata;p__norank;c__Dinophyceae;o__Peridiniales;f__Heterocapsaceae;g__Heterocapsa;s__Heterocapsa_rotundata |
| OTU3810 | 487 | KJ450987 | 95.58 | 498 | 100 | d__Eukaryota;k__Alveolata;p__norank;c__Dinophyceae;o__Peridiniales;f__Peridiniaceae;g__Peridinium;s__Peridinium_baicalense |
| OTU3812 | 497 | EF469234 | 78.52 | 512 | 100 | d__Eukaryota;k__Alveolata;p__norank;c__Dinophyceae;o__Gymnodiniales;f__Kareniaceae;g__Karenia;s__Unclassified Kareniaceae__sp. |
| OTU3814 | 499 | KJ508393 | 99 | 499 | 100 | d__Eukaryota;k__Alveolata;p__norank;c__Dinophyceae;o__Gymnodiniales;f__Gymnodiniaceae;g__Gymnodinium;s__Gymnodinium_impudicum |
| OTU3815 | 446 | KP099821 | 85.71 | 56 | 33 | d__Eukaryota;k__Alveolata;p__norank;c__Dinophyceae;o__environmental_samples;f__norank;g__norank;s__unclassified_DINO |

| | | | | | | |
|---|---|---|---|---|---|---|
| OTU3816 | 485 | HQ176320.1 | 85 | 240 | 50 | d__Eukaryota;k__Alveolata;p__norank;c__Dinophyceae;o__no rank;f__no rank;g__no rank;s__Unclassified DINO |
| OTU3819 | 490 | KJ450986 | 98.36 | 487 | 99 | d__Eukaryota;k__Alveolata;p__norank;c__Dinophyceae;o__Peridiniales;f__Peridiniaceae;g__Peridinium;s__Peridinium_baicalense |
| OTU382 | 498 | AY916546 | 96.75 | 492 | 99 | d__Eukaryota;k__Alveolata;p__norank;c__Dinophyceae;o__Peridiniales;f__Thoracosphaeraceae;g__Scrippsiella;s__Scrippsiella_sp._CS297 |
| OTU3820 | 468 | KP790228 | 88.76 | 89 | 19 | d__Eukaryota;k__Alveolata;p__norank;c__Dinophyceae;o__Gymnodiniales;f__Polykrikaceae;g__Polykrikos;s__Polykrikos-like_sp. |
| OTU3822 | 501 | AF260392 | 87.08 | 503 | 99 | d__Eukaryota;k__Alveolata;p__norank;c__Dinophyceae;o__Peridiniales;f__Thoracosphaeraceae;g__Scrippsiella;s__Scrippsiella_sp. |
| OTU3823 | 454 | KJ508367 | 98.68 | 455 | 100 | d__Eukaryota;k__Alveolata;p__norank;c__Dinophyceae;o__Gymnodiniales;f__Kareniaceae;g__Karenia;s__Karenia_papilionacea |
| OTU3826 | 488 | KJ450988 | 95.71 | 489 | 100 | d__Eukaryota;k__Alveolata;p__norank;c__Dinophyceae;o__Peridiniales;f__Peridiniaceae;g__Peridinium;s__Peridinium_euryceps |
| OTU3829 | 491 | KJ508395 | 90.02 | 491 | 100 | d__Eukaryota;k__Alveolata;p__norank;c__Dinophyceae;o__unclassified_Dinophyceae;f__norank;g__Levanderina;s__Levanderina_sp. |
| OTU3831 | 498 | JX262498 | 83.03 | 501 | 100 | d__Eukaryota;k__Alveolata;p__norank;c__Dinophyceae;o__Peridiniales;f__Peridiniaceae;g__Pentapharsodinium;s__Pentapharsodinium_dalei |
| OTU3835 | 480 | JX262498 | 80.93 | 493 | 100 | d__Eukaryota;k__Alveolata;p__norank;c__Dinophyceae;o__Peridiniales;f__Peridiniaceae;g__Pentapharsodinium;s__Pentapharsodinium_dalei |
| OTU385 | 499 | AY916546 | 94.93 | 493 | 99 | d__Eukaryota;k__Alveolata;p__norank;c__Dinophyceae;o__Peridiniales;f__Thoracosphaeraceae;g__Scrippsiella;s__Scrippsiella_sp._CS297 |
| OTU388 | 499 | AY916546 | 96.15 | 493 | 99 | d__Eukaryota;k__Alveolata;p__norank;c__Dinophyceae;o__Peridiniales;f__Thoracosphaeraceae;g__Scrippsiella;s__Scrippsiella_sp._CS297 |
| OTU389 | 508 | AY916546 | 91.2 | 500 | 98 | d__Eukaryota;k__Alveolata;p__norank;c__Dinophyceae;o__Peridiniales;f__Thoracosphaeraceae;g__Scrippsiella;s__Scrippsiella_sp._CS297 |
| OTU392 | 475 | AY916553 | 96.57 | 466 | 98 | d__Eukaryota;k__Alveolata;p__norank;c__Dinophyceae;o__Lophodiniales;f__Lophodiniaceae;g__Woloszynskia;s__Woloszynskia_sp._CS-341 |
| OTU393 | 490 | FJ024705 | 93.27 | 490 | 100 | d__Eukaryota;k__Alveolata;p__norank;c__Dinophyceae;o__Suessiales;f__Biecheleriaceae;g__Biecheleria;s__Biecheleria_cincta |
| OTU394 | 483 | FJ024705 | 95.45 | 484 | 100 | d__Eukaryota;k__Alveolata;p__norank;c__Dinophyceae;o__Suessiales;f__Biecheleriaceae;g__Biecheleria;s__Biecheleria_cincta |
| OTU395 | 448 | AY916553 | 96.88 | 449 | 99 | d__Eukaryota;k__Alveolata;p__norank;c__Dinophyceae;o__Lophodiniales;f__Lophodiniaceae;g__Woloszynskia;s__Woloszynskia_sp._CS-341 |
| OTU396 | 441 | AY916553 | 96.55 | 435 | 99 | d__Eukaryota;k__Alveolata;p__norank;c__Dinophyceae;o__Lophodiniales;f__Lophodiniaceae;g__Woloszynskia;s__Woloszynskia_sp._CS-341 |
| OTU398 | 472 | AY916553 | 96.33 | 463 | 98 | d__Eukaryota;k__Alveolata;p__norank;c__Dinophyceae;o__Lophodiniales;f__Lophodiniaceae;g__Woloszynskia;s__Woloszynskia_sp._CS-341 |
| OTU4 | 259 | KF245459 | 79.28 | 111 | 43 | d__Eukaryota;k__Alveolata;p__norank;c__Dinophyceae;o__unclassified;f__unclassified;g__unclassified;s__unclassified DINO |

| | | | | | | |
|---|---|---|---|---|---|---|
| OTU40 | 515 | AF260393 | 96.7 | 515 | 100 | d__Eukaryota;k__Alveolata;p__norank;c__Dinophyceae;o__Peridiniales;f__Thoracosphaeraceae;g__Scrippsiella;s__Scrippsiella_trochoidea |
| OTU42 | 513 | AF260393 | 97.86 | 513 | 100 | d__Eukaryota;k__Alveolata;p__norank;c__Dinophyceae;o__Peridiniales;f__Thoracosphaeraceae;g__Scrippsiella;s__Scrippsiella_trochoidea |
| OTU45 | 516 | AF260393 | 97.09 | 516 | 100 | d__Eukaryota;k__Alveolata;p__norank;c__Dinophyceae;o__Peridiniales;f__Thoracosphaeraceae;g__Scrippsiella;s__Scrippsiella_trochoidea |
| OTU459 | 485 | AY154965 | 76.63 | 475 | 95 | d__Eukaryota;k__Alveolata;p__norank;c__Dinophyceae;o__Gonyaulacales;f__Gonyaulacaceae;g__Gonyaulax;s__Gonyaulax_sp. |
| OTU461 | 478 | AY154965 | 74.14 | 406 | 81 | d__Eukaryota;k__Alveolata;p__norank;c__Dinophyceae;o__Gonyaulacales;f__Gonyaulacaceae;g__Gonyaulax;s__Gonyaulax_sp. |
| OTU464 | 451 | DQ162802 | 95.37 | 454 | 100 | d__Eukaryota;k__Alveolata;p__norank;c__Dinophyceae;o__Gonyaulacales;f__Gonyaulacaceae;g__Gonyaulax;s__Gonyaulax_polygramma |
| OTU470 | 378 | AY154965 | 73.74 | 396 | 99 | d__Eukaryota;k__Alveolata;p__norank;c__Dinophyceae;o__Gonyaulacales;f__Gonyaulacaceae;g__Gonyaulax;s__Gonyaulax_membranacea |
| OTU475 | 411 | AY154965 | 73.26 | 430 | 99 | d__Eukaryota;k__Alveolata;p__norank;c__Dinophyceae;o__Gonyaulacales;f__Gonyaulacaceae;g__Gonyaulax;s__Gonyaulax_membranacea |
| OTU481 | 472 | JN558110 | 82.77 | 499 | 100 | d__Eukaryota;k__Alveolata;p__norank;c__Dinophyceae;o__Suessiales;f__Suessiaceae;g__Polarella;s__Polarella_glacialis |
| OTU482 | 475 | DQ195346 | 93.47 | 475 | 100 | d__Eukaryota;k__Alveolata;p__norank;c__Dinophyceae;o__Suessiales;f__Suessiaceae;g__Pelagodinium;s__Pelagodinium_béii |
| OTU483 | 487 | DQ195346 | 99.59 | 487 | 100 | d__Eukaryota;k__Alveolata;p__norank;c__Dinophyceae;o__Suessiales;f__Suessiaceae;g__Pelagodinium;s__Pelagodinium_béii |
| OTU485 | 480 | JN558107 | 93.1 | 478 | 99 | d__Eukaryota;k__Alveolata;p__norank;c__Dinophyceae;o__Suessiales;f__Suessiaceae;g__Pelagodinium;s__Pelagodinium_béii |
| OTU486 | 517 | AY571374.1 | 78 | 517 | 100 | d__Eukaryota;k__Alveolata;p__norank;c__Dinophyceae;o__Lophodiniales; f__Lophodiniaceae;g__Woloszynskia;s__Woloszynskia_sp. |
| OTU487 | 264 | DQ195364 | 97.33 | 262 | 98 | d__Eukaryota;k__Alveolata;p__norank;c__Dinophyceae;o__Suessiales;f__Suessiaceae;g__Pelagodinium;s__Pelagodinium_béii |
| OTU488 | 492 | DQ195367 | 98.14 | 484 | 98 | d__Eukaryota;k__Alveolata;p__norank;c__Dinophyceae;o__Suessiales;f__Suessiaceae;g__Pelagodinium;s__Pelagodinium_béii |
| OTU489 | 481 | DQ195368 | 100 | 481 | 100 | d__Eukaryota;k__Alveolata;p__norank;c__Dinophyceae;o__Suessiales;f__Suessiaceae;g__Pelagodinium;s__Pelagodinium_béii |
| OTU490 | 477 | DQ195368 | 96.86 | 478 | 100 | d__Eukaryota;k__Alveolata;p__norank;c__Dinophyceae;o__Suessiales;f__Suessiaceae;g__Pelagodinium;s__Pelagodinium_béii |
| OTU493 | 495 | FJ024705 | 82.02 | 495 | 99 | d__Eukaryota;k__Alveolata;p__norank;c__Dinophyceae;o__Suessiales;f__Biecheleriaceae;g__Biecheleria;s__Biecheleria_cincta |
| OTU494 | 492 | LC068838.1 | 88 | 492 | 100 | d__Eukaryota;k__Alveolata;p__norank;c__Dinophyceae;o__Suessiales;f__Suessiaceae;g__Unclassified;s__Unclassified Suessiaceae |
| OTU495 | 492 | LC068838.1 | 87 | 492 | 100 | d__Eukaryota;k__Alveolata;p__norank;c__Dinophyceae;o__Suessiales;f__Suessiaceae;g__Unclassified;s__Unclassified Suessiaceae |

| | | | | | | |
|---|---|---|---|---|---|---|
| OTU496 | 485 | FJ024705 | 81.39 | 489 | 100 | d__Eukaryota;k__Alveolata;p__norank;c__Dinophyceae;o__Suessiales;f__Biecheleriaceae;g__Biecheleria;s__Biecheleria_cincta |
| OTU497 | 467 | JN558110 | 76.54 | 486 | 100 | d__Eukaryota;k__Alveolata;p__norank;c__Dinophyceae;o__Suessiales;f__Suessiaceae;g__Polarella;s__Polarella_glacialis |
| OTU499 | 447 | DQ195376 | 96.65 | 448 | 100 | d__Eukaryota;k__Alveolata;p__norank;c__Dinophyceae;o__Suessiales;f__Suessiaceae;g__Pelagodinium;s__Pelagodinium_béii |
| OTU505 | 437 | JQ439944 | 84.48 | 58 | 37 | d__Eukaryota;k__Alveolata;p__norank;c__Dinophyceae;o__Gymnodiniales;f__Unclassified;g__Unclassified;s__Unclassified_Gymnodiniales |
| OTU506 | 463 | JQ439944 | 79.28 | 111 | 46 | d__Eukaryota;k__Alveolata;p__norank;c__Dinophyceae;o__Gymnodiniales;f__Unclassified;g__Unclassified;s__Unclassified_Gymnodiniales |
| OTU507 | 474 | DQ289020 | 90.62 | 64 | 36 | d__Eukaryota;k__Alveolata;p__norank;c__Dinophyceae;o__Gymnodiniales;f__Unclassified;g__Unclassified;s__Unclassified_Gymnodiniales |
| OTU508 | 444 | DQ286734 | 100 | 444 | 100 | d__Eukaryota;k__Alveolata;p__norank;c__Dinophyceae;o__Gymnodiniales;f__Gymnodiniaceae;g__Gymnodinium;s__Gymnodinium_catenatum |
| OTU519 | 448 | DQ320627 | 98.21 | 448 | 100 | d__Eukaryota;k__Alveolata;p__norank;c__Dinophyceae;o__Gymnodiniales;f__Tovelliaceae;g__Tovellia;s__Tovellia_sanguinea |
| OTU522 | 475 | DQ444233 | 84 | 475 | 100 | d__Eukaryota;k__Alveolata;p__norank;c__Dinophyceae;o__Peridiniales;f__Protoperidiniaceae;g__Protoperidinium;s__Protoperidinium_pellucidum |
| OTU529 | 475 | DQ480430 | 94.12 | 34 | 21 | d__Eukaryota;k__Alveolata;p__norank;c__Dinophyceae;o__Suessiales;f__Symbiodiniaceae;g__Symbiodinium;s__unclassified_Symbiodinium |
| OTU530 | 450 | DQ480430 | 94.29 | 35 | 22 | d__Eukaryota;k__Alveolata;p__norank;c__Dinophyceae;o__Suessiales;f__Symbiodiniaceae;g__Symbiodinium;s__unclassified_Symbiodinium |
| OTU532 | 480 | AM408889 | 82.27 | 485 | 100 | d__Eukaryota;k__Alveolata;p__norank;c__Dinophyceae;o__Gymnodiniales;f__Gymnodiniaceae;g__Paragymnodinium;s__Paragymnodinium_shiwhaense |
| OTU533 | 503 | FJ032677 | 80.71 | 508 | 99 | d__Eukaryota;k__Alveolata;p__norank;c__Dinophyceae;o__environmental_samples;f__norank;g__norank;s__uncultured_marine_DINO |
| OTU535 | 435 | AM408889 | 79.27 | 439 | 100 | d__Eukaryota;k__Alveolata;p__norank;c__Dinophyceae;o__Gymnodiniales;f__Gymnodiniaceae;g__Paragymnodinium;s__Paragymnodinium_shiwhaense |
| OTU548 | 373 | AB265968 | 83.91 | 379 | 100 | d__Eukaryota;k__Alveolata;p__norank;c__Dinophyceae;o__Gymnodiniales;f__Gymnodiniaceae;g__Gymnodinium;s__Gymnodinium_microreticulatum |
| OTU554 | 515 | DQ991375 | 99.03 | 516 | 100 | d__Eukaryota;k__Alveolata;p__norank;c__Dinophyceae;o__Peridiniales;f__Pfiesteriaceae;g__Cryptoperidiniopsis;s__Cryptoperidiniopsis_brodyi |
| OTU555 | 509 | DQ991375 | 97.45 | 509 | 100 | d__Eukaryota;k__Alveolata;p__norank;c__Dinophyceae;o__Peridiniales;f__Pfiesteriaceae;g__Cryptoperidiniopsis;s__Cryptoperidiniopsis_brodyi |
| OTU556 | 498 | DQ991376 | 95.6 | 500 | 100 | d__Eukaryota;k__Alveolata;p__norank;c__Dinophyceae;o__Peridiniales;f__Pfiesteriaceae;g__Cryptoperidiniopsis;s__Cryptoperidiniopsis_brodyi |
| OTU56 | 499 | AF260393 | 97.18 | 497 | 99 | d__Eukaryota;k__Alveolata;p__norank;c__Dinophyceae;o__Peridiniales;f__Thoracosphaeraceae;g__Scrippsiella;s__Scrippsiella_trochoidea |
| OTU57 | 505 | AF260393 | 98.57 | 490 | 97 | d__Eukaryota;k__Alveolata;p__norank;c__Dinophyceae;o__Peridiniales;f__Thoracosphaeraceae;g__Scrippsiella;s__Scrippsiella_trochoidea |

| | | | | | | |
|---|---|---|---|---|---|---|
| OTU573 | 490 | AM408889 | 81.48 | 486 | 99 | d__Eukaryota;k__Alveolata;p__norank;c__Dinophyceae;o__Gymnodiniales;f__Gymnodiniaceae;g__Paragymnodinium;s__Paragymnodinium_shiwhaense |
| OTU58 | 505 | AF260393 | 97.23 | 505 | 100 | d__Eukaryota;k__Alveolata;p__norank;c__Dinophyceae;o__Peridiniales;f__Thoracosphaeraceae;g__Scrippsiella;s__Scrippsiella_trochoidea |
| OTU60 | 498 | AF260393 | 94.19 | 396 | 79 | d__Eukaryota;k__Alveolata;p__norank;c__Dinophyceae;o__Peridiniales;f__Thoracosphaeraceae;g__Scrippsiella;s__Scrippsiella_sp. |
| OTU601 | 477 | KM042422 | 76.3 | 481 | 100 | d__Eukaryota;k__Alveolata;p__norank;c__Dinophyceae;o__Peridiniales;f__Protoperidiniaceae;g__Protoperidinium;s__Protoperidinium_abei |
| OTU614 | 507 | EF052683 | 96.65 | 507 | 100 | d__Eukaryota;k__Alveolata;p__norank;c__Dinophyceae;o__Peridiniales;f__unclassified_Peridiniales;g__Baldinia;s__Baldinia_anauniensis |
| OTU615 | 490 | EF052683 | 91.26 | 492 | 100 | d__Eukaryota;k__Alveolata;p__norank;c__Dinophyceae;o__Peridiniales;f__unclassified_Peridiniales;g__Baldinia;s__Baldinia_anauniensis |
| OTU62 | 413 | AF260393 | 95.27 | 402 | 97 | d__Eukaryota;k__Alveolata;p__norank;c__Dinophyceae;o__Peridiniales;f__Thoracosphaeraceae;g__Scrippsiella;s__Scrippsiella_trochoidea |
| OTU634 | 265 | KT389966 | 97 | 246 | 100 | d__Eukaryota;k__Alveolata;p__norank;c__Dinophyceae;o__Peridiniales;f__Peridiniaceae;g__Pentapharsodinium;s__Pentapharsodinium_tyrrhenicum |
| OTU638 | 485 | AY571374 | 84.63 | 488 | 99 | d__Eukaryota;k__Alveolata;p__norank;c__Dinophyceae;o__Lophodiniales;f__Lophodiniaceae;g__Woloszynskia;s__Woloszynskia_tenuissima |
| OTU639 | 487 | AY571374 | 83.95 | 486 | 99 | d__Eukaryota;k__Alveolata;p__norank;c__Dinophyceae;o__Lophodiniales;f__Lophodiniaceae;g__Woloszynskia;s__Woloszynskia_tenuissima |
| OTU64 | 498 | AF260393 | 95.98 | 498 | 100 | d__Eukaryota;k__Alveolata;p__norank;c__Dinophyceae;o__Peridiniales;f__Thoracosphaeraceae;g__Scrippsiella;s__Scrippsiella_trochoidea |
| OTU640 | 485 | AY571374 | 84.62 | 481 | 99 | d__Eukaryota;k__Alveolata;p__norank;c__Dinophyceae;o__Lophodiniales;f__Lophodiniaceae;g__Woloszynskia;s__Woloszynskia_tenuissima |
| OTU641 | 485 | AY571374 | 84.12 | 485 | 99 | d__Eukaryota;k__Alveolata;p__norank;c__Dinophyceae;o__Lophodiniales;f__Lophodiniaceae;g__Woloszynskia;s__Woloszynskia_tenuissima |
| OTU642 | 489 | AY571374 | 80.52 | 462 | 92 | d__Eukaryota;k__Alveolata;p__norank;c__Dinophyceae;o__Lophodiniales;f__Lophodiniaceae;g__Woloszynskia;s__Woloszynskia_tenuissima |
| OTU643 | 504 | AY571374 | 81.27 | 411 | 80 | d__Eukaryota;k__Alveolata;p__norank;c__Dinophyceae;o__Lophodiniales;f__Lophodiniaceae;g__Woloszynskia;s__Woloszynskia-like_Sp. |
| OTU644 | 502 | AY571374 | 83.85 | 452 | 89 | d__Eukaryota;k__Alveolata;p__norank;c__Dinophyceae;o__Lophodiniales;f__Lophodiniaceae;g__Woloszynskia;s__Woloszynskia-like_sp. |
| OTU645 | 501 | AY571374 | 81.05 | 459 | 90 | d__Eukaryota;k__Alveolata;p__norank;c__Dinophyceae;o__Lophodiniales;f__Lophodiniaceae;g__Woloszynskia;s__Woloszynskia-like_sp. |
| OTU646 | 500 | AY571374 | 81.19 | 452 | 90 | d__Eukaryota;k__Alveolata;p__norank;c__Dinophyceae;o__Lophodiniales;f__Lophodiniaceae;g__Woloszynskia;s__Woloszynskia-like_sp. |
| OTU647 | 495 | AY571374 | 81.62 | 457 | 91 | d__Eukaryota;k__Alveolata;p__norank;c__Dinophyceae;o__Lophodiniales;f__Lophodiniaceae;g__Woloszynskia;s__Woloszynskia-like_sp. |
| OTU648 | 503 | AY571374 | 78.12 | 425 | 77 | d__Eukaryota;k__Alveolata;p__norank;c__Dinophyceae;o__Lophodiniales;f__Lophodiniaceae;g__Woloszynskia;s__Woloszynskia-like_sp. |

| | | | | | | |
|---|---|---|---|---|---|---|
| OTU649 | 437 | AY571374 | 75 | 452 | 99 | d__Eukaryota;k__Alveolata;p__norank;c__Dinophyceae;o__Lophodiniales;f__Lophodiniaceae;g__Woloszynskia;s__Woloszynskia_sp. |
| OTU650 | 283 | EF058276 | 94.31 | 281 | 99 | d__Eukaryota;k__Alveolata;p__norank;c__Dinophyceae;o__Lophodiniales;f__Lophodiniaceae;g__Woloszynskia;s__Woloszynskia_pascheri |
| OTU651 | 351 | EF058276 | 77.58 | 281 | 79 | d__Eukaryota;k__Alveolata;p__norank;c__Dinophyceae;o__Lophodiniales;f__Lophodiniaceae;g__Woloszynskia;s__Woloszynskia-like_sp. |
| OTU652 | 368 | EF058276 | 86.02 | 279 | 76 | d__Eukaryota;k__Alveolata;p__norank;c__Dinophyceae;o__Lophodiniales;f__Lophodiniaceae;g__Woloszynskia;s__Woloszynskia-like_sp. |
| OTU653 | 327 | EF058276 | 87.41 | 278 | 85 | d__Eukaryota;k__Alveolata;p__norank;c__Dinophyceae;o__Lophodiniales;f__Lophodiniaceae;g__Woloszynskia;s__Woloszynskia-like_sp. |
| OTU654 | 322 | AY571374 | 96.89 | 322 | 100 | d__Eukaryota;k__Alveolata;p__norank;c__Dinophyceae;o__Lophodiniales;f__Lophodiniaceae;g__Woloszynskia;s__Woloszynskia_tenuissima |
| OTU655 | 224 | KM058700 | 96.23 | 106 | 47 | d__Eukaryota;k__Alveolata;p__norank;c__Dinophyceae;o__Suessiales;f__Unclassified;g__Unclassified;s__Unclassified DINO |
| OTU656 | 235 | EF058276 | 86.36 | 220 | 94 | d__Eukaryota;k__Alveolata;p__norank;c__Dinophyceae;o__Lophodiniales;f__Lophodiniaceae;g__Woloszynskia;s__Woloszynskia_pascheri |
| OTU657 | 248 | EF058276 | 94.76 | 248 | 99 | d__Eukaryota;k__Alveolata;p__norank;c__Dinophyceae;o__Lophodiniales;f__Lophodiniaceae;g__Woloszynskia;s__Woloszynskia_pascheri |
| OTU670 | 476 | KT389956.1 | 86 | 481 | 100 | d__Eukaryota;k__Alveolata;p__norank;c__Dinophyceae;o__Peridiniales;f__Thoracosphaeraceae;g__Scrippsiella;s__Scrippsiella_sp. |
| OTU671 | 472 | KT389946.1 | 84 | 478 | 100 | d__Eukaryota;k__Alveolata;p__norank;c__Dinophyceae;o__Peridiniales;f__Thoracosphaeraceae;g__Scrippsiella;s__Scrippsiella_sp. |
| OTU674 | 493 | JF430394 | 78.47 | 497 | 100 | d__Eukaryota;k__Alveolata;p__norank;c__Dinophyceae;o__Peridiniales;f__Pfiesteriaceae;g__Chimonodinium;s__Chimonodinium_lomnickii |
| OTU676 | 502 | HQ176320 | 77.53 | 503 | 99 | d__Eukaryota;k__Alveolata;p__norank;c__Dinophyceae;o__Peridiniales;f__Pfiesteriaceae;g__Chimonodinium;s__Chimonodinium_lomnickii |
| OTU677 | 496 | KP702719 | 99.8 | 496 | 100 | d__Eukaryota;k__Alveolata;p__norank;c__Dinophyceae;o__Peridiniales;f__Diplopsaliaceae;g__Oblea;s__Oblea_rotunda |
| OTU679 | 487 | EF152794 | 85.04 | 488 | 100 | d__Eukaryota;k__Alveolata;p__norank;c__Dinophyceae;o__Peridiniales;f__Diplopsaliaceae;g__Diplopsalis;s__Diplopsalis_lenticula |
| OTU683 | 455 | KP702719 | 95.83 | 456 | 100 | d__Eukaryota;k__Alveolata;p__norank;c__Dinophyceae;o__Peridiniales;f__Diplopsaliaceae;g__Oblea;s__Oblea_rotunda |
| OTU695 | 461 | EF152953 | 91.99 | 462 | 100 | d__Eukaryota;k__Alveolata;p__norank;c__Dinophyceae;o__Peridiniales;f__Diplopsaliaceae;g__Preperidinium;s__Preperidinium_sp._M064sm1 |
| OTU697 | 524 | EF152961 | 96.37 | 524 | 100 | d__Eukaryota;k__Alveolata;p__norank;c__Dinophyceae;o__Peridiniales;f__Diplopsaliaceae;g__Preperidinium;s__Protoperidinium_cf._steidingerae |
| OTU701 | 521 | EF152961 | 96.16 | 521 | 100 | d__Eukaryota;k__Alveolata;p__norank;c__Dinophyceae;o__Peridiniales;f__Diplopsaliaceae;g__Preperidinium;s__Protoperidinium_cf._steidingerae |
| OTU702 | 527 | EF152961 | 90.74 | 529 | 100 | d__Eukaryota;k__Alveolata;p__norank;c__Dinophyceae;o__Peridiniales;f__Diplopsaliaceae;g__Preperidinium;s__Protoperidinium_cf._steidingerae |

| | | | | | | |
|---|---|---|---|---|---|---|
| OTU71 | 341 | AF260393 | 96.44 | 337 | 99 | d__Eukaryota;k__Alveolata;p__norank;c__Dinophyceae;o__Peridiniales;f__Thoracosphaeraceae;g__Scrippsiella;s__Scrippsiella_trochoidea |
| OTU718 | 506 | EF152961 | 94.73 | 493 | 97 | d__Eukaryota;k__Alveolata;p__norank;c__Dinophyceae;o__Peridiniales;f__Diplopsaliaceae;g__Preperidinium;s__Protoperidinium_cf._steidingerae |
| OTU719 | 517 | EF152961 | 88.87 | 557 | 99 | d__Eukaryota;k__Alveolata;p__norank;c__Dinophyceae;o__Peridiniales;f__Diplopsaliaceae;g__Preperidinium;s__Protoperidinium_cf._steidingerae |
| OTU72 | 488 | AF260394 | 100 | 488 | 100 | d__Eukaryota;k__Alveolata;p__norank;c__Dinophyceae;o__Peridiniales;f__Peridiniaceae;g__Peridinium;s__Peridinium_palatinum |
| OTU723 | 517 | AB288382 | 99.81 | 517 | 100 | d__Eukaryota;k__Alveolata;p__norank;c__Dinophyceae;o__Gymnodiniales;f__Gymnodiniaceae;g__Cochlodinium;s__Cochlodinium_fulvescens |
| OTU740 | 474 | EF205010 | 97.72 | 482 | 100 | d__Eukaryota;k__Alveolata;p__norank;c__Dinophyceae;o__Peridiniales;f__Glenodiniaceae;g__Peridiniopsis;s__Peridiniopsis_polonicum |
| OTU743 | 294 | EF205014 | 99.32 | 294 | 100 | d__Eukaryota;k__Alveolata;p__norank;c__Dinophyceae;o__unclassified_Dinophyceae;f__norank;g__Protodinium;s__Protodinium_simplex |
| OTU745 | 429 | EF205019 | 97.67 | 430 | 100 | d__Eukaryota;k__Alveolata;p__norank;c__Dinophyceae;o__Lophodiniales;f__Lophodiniaceae;g__Woloszynskia;s__Woloszynskia_halophila |
| OTU75 | 498 | AF260392 | 86 | 500 | 100 | d__Eukaryota;k__Alveolata;p__norank;c__Dinophyceae;o__Peridiniales;f__Thoracosphaeraceae;g__Scrippsiella;s__Scrippsiella_sp. |
| OTU754 | 405 | KJ508376.1 | 77 | 74 | 72 | d__Eukaryota;k__Alveolata;p__norank;c__Dinophyceae;o__Gymnodiniales;f__Kareniaceae-like;g__Unclassified;s__Unclassified DINO |
| OTU755 | 415 | X16108.1 | 77 | 75 | 69 | d__Eukaryota;k__Alveolata;p__norank;c__Dinophyceae;o__norank;f__norank;g__norank;s__Unclassified DINO |
| OTU76 | 499 | AF260392 | 87.23 | 501 | 100 | d__Eukaryota;k__Alveolata;p__norank;c__Dinophyceae;o__Peridiniales;f__Thoracosphaeraceae;g__Scrippsiella;s__Scrippsiella_sp. |
| OTU768 | 482 | EF613349 | 84.42 | 77 | 53 | d__Eukaryota;k__Alveolata;p__norank;c__Dinophyceae;o__Gonyaulacales;f__Gonyaulacaceae;g__Unclassified;s__Unclassified DINO |
| OTU77 | 499 | JX262498 | 86.4 | 500 | 100 | d__Eukaryota;k__Alveolata;p__norank;c__Dinophyceae;o__Peridiniales;f__Peridiniaceae;g__Pentapharsodinium;s__Pentapharsodinium_dalei |
| OTU771 | 489 | EF613353 | 98.57 | 490 | 100 | d__Eukaryota;k__Alveolata;p__norank;c__Dinophyceae;o__unclassified_Dinophyceae;f__norank;g__Levanderina;s__Levanderina_fissa |
| OTU772 | 504 | EF613353 | 94.64 | 504 | 100 | d__Eukaryota;k__Alveolata;p__norank;c__Dinophyceae;o__unclassified_Dinophyceae;f__norank;g__Levanderina;s__Levanderina_fissa |
| OTU774 | 511 | JF430394 | 83.82 | 513 | 100 | d__Eukaryota;k__Alveolata;p__norank;c__Dinophyceae;o__Peridiniales;f__Pfiesteriaceae;g__Chimonodinium;s__Chimonodinium_sp. |
| OTU776 | 515 | KT389885.1 | 93 | 88 | 80 | d__Eukaryota;k__Alveolata;p__norank;c__Dinophyceae;o__Gonyaulacales; f__Amphidomataceae;g__norank;s__Unclassified DINO |
| OTU778 | 522 | EF613356 | 77.63 | 541 | 100 | d__Eukaryota;k__Alveolata;p__norank;c__Dinophyceae;o__Gymnodiniales;f__Gymnodiniaceae;g__Katodinium;s__Katodinium_glaucum |
| OTU78 | 497 | AF260393 | 86 | 500 | 100 | d__Eukaryota;k__Alveolata;p__norank;c__Dinophyceae;o__Peridiniales;f__Thoracosphaeraceae;g__Scrippsiella;s__Scrippsiella_trochoidea |

| | | | | | | |
|---|---|---|---|---|---|---|
| OTU781 | 524 | KP790221 | 97.53 | 160 | 33 | d__Eukaryota;k__Alveolata;p__norank;c__Dinophyceae;o__Gymnodiniales;f__Gymnodiniaceae;g__Katodinium-like;s__Katodinium-like_sp. |
| OTU90 | 499 | AF260400 | 97.19 | 498 | 99 | d__Eukaryota;k__Alveolata;p__norank;c__Dinophyceae;o__Peridiniales;f__Heterocapsaceae;g__Heterocapsa;s__Heterocapsa_rotundata |
| OTU91 | 492 | AF260400 | 98.17 | 493 | 100 | d__Eukaryota;k__Alveolata;p__norank;c__Dinophyceae;o__Peridiniales;f__Heterocapsaceae;g__Heterocapsa;s__Heterocapsa_rotundata |
| OTU92 | 487 | AF260400 | 96.92 | 487 | 100 | d__Eukaryota;k__Alveolata;p__norank;c__Dinophyceae;o__Peridiniales;f__Heterocapsaceae;g__Heterocapsa;s__Heterocapsa_rotundata |
| OTU93 | 483 | AF260400 | 96.07 | 483 | 100 | d__Eukaryota;k__Alveolata;p__norank;c__Dinophyceae;o__Peridiniales;f__Heterocapsaceae;g__Heterocapsa;s__Heterocapsa_rotundata |
| OTU94 | 490 | AF260400 | 96.74 | 491 | 100 | d__Eukaryota;k__Alveolata;p__norank;c__Dinophyceae;o__Peridiniales;f__Heterocapsaceae;g__Heterocapsa;s__Heterocapsa_rotundata |
| OTU950 | 486 | EF613366 | 92.18 | 486 | 100 | d__Eukaryota;k__Alveolata;p__norank;c__Dinophyceae;o__Peridiniales;f__Thoracosphaeraceae;g__Scrippsiella;s__Scrippsiella_trochoidea |
| OTU953 | 527 | EF613367 | 98.67 | 527 | 100 | d__Eukaryota;k__Alveolata;p__norank;c__Dinophyceae;o__Gymnodiniales;f__Polykrikaceae;g__Polykrikos;s__Polykrikos_kofoidii |
| OTU954 | 524 | EF613367 | 98.73 | 474 | 90 | d__Eukaryota;k__Alveolata;p__norank;c__Dinophyceae;o__Gymnodiniales;f__Polykrikaceae;g__Polykrikos;s__Polykrikos_kofoidii |
| OTU958 | 489 | EU165308 | 82.41 | 506 | 100 | d__Eukaryota;k__Alveolata;p__norank;c__Dinophyceae;o__Gymnodiniales;f__Kareniaceae;g__Karenia;s__Karenia_brevis |
| OTU959 | 496 | EF469234 | 94.35 | 496 | 100 | d__Eukaryota;k__Alveolata;p__norank;c__Dinophyceae;o__Gymnodiniales;f__Kareniaceae;g__Karlodinium;s__Karlodinium_antarcticum |
| OTU960 | 511 | EF469234 | 89.34 | 516 | 100 | d__Eukaryota;k__Alveolata;p__norank;c__Dinophyceae;o__Gymnodiniales;f__Kareniaceae;g__Karlodinium;s__Karlodinium_antarcticum |
| OTU962 | 488 | EF469234 | 87.58 | 499 | 100 | d__Eukaryota;k__Alveolata;p__norank;c__Dinophyceae;o__Gymnodiniales;f__Kareniaceae;g__Karlodinium;s__Karlodinium_antarcticum |
| OTU97 | 485 | AF260400 | 95.67 | 485 | 100 | d__Eukaryota;k__Alveolata;p__norank;c__Dinophyceae;o__Peridiniales;f__Heterocapsaceae;g__Heterocapsa;s__Heterocapsa_rotundata |
| OTU975 | 505 | AB295051 | 93.89 | 507 | 100 | d__Eukaryota;k__Alveolata;p__norank;c__Dinophyceae;o__Gymnodiniales;f__Gymnodiniaceae;g__Cochlodinium;s__Cochlodinium_fulvescens |
| OTU976 | 512 | EF616462 | 86.16 | 513 | 99 | d__Eukaryota;k__Alveolata;p__norank;c__Dinophyceae;o__Gymnodiniales;f__Gymnodiniaceae;g__Cochlodinium;s__Cochlodinium_cf._geminatum |
| OTU977 | 517 | EF616462 | 81.73 | 520 | 100 | d__Eukaryota;k__Alveolata;p__norank;c__Dinophyceae;o__Gymnodiniales;f__Gymnodiniaceae;g__Cochlodinium;s__Cochlodinium_cf._geminatum |
| OTU979 | 516 | EF616462 | 84.04 | 520 | 100 | d__Eukaryota;k__Alveolata;p__norank;c__Dinophyceae;o__Gymnodiniales;f__Gymnodiniaceae;g__Cochlodinium;s__Cochlodinium_cf._geminatum |
| OTU98 | 498 | EF616464 | 92.65 | 68 | 61 | d__Eukaryota;k__Alveolata;p__norank;c__Dinophyceae;o__Lophodiniales;f__Lophodiniaceae;g__Woloszynskia;s__Woloszynskia-like_sp. |
| OTU980 | 497 | EF616462 | 94.74 | 133 | 90 | d__Eukaryota;k__Alveolata;p__norank;c__Dinophyceae;o__Gymnodiniales;f__Gymnodiniaceae;g__Cochlodinium;s__Cochlodinium_cf._geminatum-like |

| | | | | | | |
|---|---|---|---|---|---|---|
| OTU984 | 501 | EF616462 | 84.1 | 497 | 99 | d__Eukaryota;k__Alveolata;p__norank;c__Dinophyceae;o__Gymnodiniales;f__Gymnodiniaceae;g__Cochlodinium;s__Cochlodinium_cf._geminatum |
| OTU986 | 511 | EF616462 | 85.28 | 496 | 97 | d__Eukaryota;k__Alveolata;p__norank;c__Dinophyceae;o__Gymnodiniales;f__Gymnodiniaceae;g__Cochlodinium;s__Cochlodinium_cf._geminatum |
| OTU999 | 385 | EF616462 | 86.75 | 317 | 82 | d__Eukaryota;k__Alveolata;p__norank;c__Dinophyceae;o__Gymnodiniales;f__Gymnodiniaceae;g__Cochlodinium;s__Cochlodinium_cf._geminatum |

**Table S2.** The 51 operational taxonomic units (OTUs) taxonomically identical to an entity in GenBank but having no species name provided.

| OTU ID | OUT length | Gi number | Identity | Align length | Coverage | Taxon |
|---|---|---|---|---|---|---|
| OTU1034 | 524 | EU048553 | 98.46 | 520 | 99 | d__Eukaryota;k__Alveolata;p__norank;c__Dinophyceae;o__Peridiniales;f__Pfiesteriaceae;g__unclassified_Pfiesteriaceae;s__Pfiesteriaceae_sp._masanensis |
| OTU1043 | 432 | EU048553 | 97.92 | 433 | 100 | d__Eukaryota;k__Alveolata;p__norank;c__Dinophyceae;o__Peridiniales;f__Pfiesteriaceae;g__unclassified_Pfiesteriaceae;s__Pfiesteriaceae_sp._masanensis |
| OTU105 | 251 | AF318248 | 96.41 | 251 | 99 | d__Eukaryota;k__Alveolata;p__norank;c__Dinophyceae;o__Gymnodiniales;f__Gymnodiniaceae;g__Gymnodinium;s__unclassified_Gymnodinium |
| OTU1051 | 374 | EU048553 | 99.43 | 352 | 94 | d__Eukaryota;k__Alveolata;p__norank;c__Dinophyceae;o__Peridiniales;f__Pfiesteriaceae;g__unclassified_Pfiesteriaceae;s__Pfiesteriaceae_sp._masanensis |
| OTU1150 | 317 | EU165289 | 97.16 | 317 | 100 | d__Eukaryota;k__Alveolata;p__norank;c__Dinophyceae;o__Peridiniales;f__Thoracosphaeraceae;g__Scrippsiella;s__Scrippsiella_sp._CCMP2775 |
| OTU1151 | 261 | EU165289 | 98.43 | 255 | 98 | d__Eukaryota;k__Alveolata;p__norank;c__Dinophyceae;o__Peridiniales;f__Thoracosphaeraceae;g__Scrippsiella;s__Scrippsiella_sp._CCMP2775 |
| OTU1184 | 544 | KT390028.1 | 95.22 | 544 | 100 | d__Eukaryota;k__Alveolata;p__norank;c__Dinophyceae;o__Blastodiniales;f__norank;g__Blastodinium;s__Blastodinium_sp. |
| OTU1370 | 209 | FJ024706 | 98.06 | 206 | 98 | d__Eukaryota;k__Alveolata;p__norank;c__Dinophyceae;o__Lophodiniales;f__Lophodiniaceae;g__Woloszynskia;s__Woloszynskia_sp._MB-1 |
| OTU1372 | 229 | FJ024706 | 98.67 | 225 | 98 | d__Eukaryota;k__Alveolata;p__norank;c__Dinophyceae;o__Lophodiniales;f__Lophodiniaceae;g__Woloszynskia;s__Woloszynskia_sp._MB-1 |
| OTU1414 | 521 | KT390028 | 94.63 | 521 | 100 | d__Eukaryota;k__Alveolata;p__norank;c__Dinophyceae;o__Blastodiniales; f__Blastodiniales;g__Blastodinium-like;s__Blastodinium-like_sp. |
| OTU1570 | 500 | KP790240 | 98.33 | 419 | 84 | d__Eukaryota;k__Alveolata;p__norank;c__Dinophyceae;o__Gymnodiniales;f__Warnowiaceae;g__Warnowia;s__Warnowia_sp._4_AR-2015 |
| OTU1577 | 418 | KP790242 | 98.8 | 418 | 100 | d__Eukaryota;k__Alveolata;p__norank;c__Dinophyceae;o__Gymnodiniales;f__Warnowiaceae;g__Warnowia;s__Warnowia_sp._5_AR-2015 |
| OTU1636 | 494 | FN557541 | 96.76 | 494 | 100 | d__Eukaryota;k__Alveolata;p__norank;c__Dinophyceae;o__unclassified_Dinophyceae;f__norank;g__Stoeckeria;s__Stoeckeria_sp._SSMS0806 |
| OTU1639 | 504 | FN557541 | 99.8 | 504 | 100 | d__Eukaryota;k__Alveolata;p__norank;c__Dinophyceae;o__unclassified_Dinophyceae;f__norank;g__Stoeckeria;s__Stoeckeria_sp._SSMS0806 |
| OTU1643 | 498 | FN557541 | 96.79 | 498 | 100 | d__Eukaryota;k__Alveolata;p__norank;c__Dinophyceae;o__unclassified_Dinophyceae;f__norank;g__Stoeckeria;s__Stoeckeria_sp._SSMS0806 |
| OTU185 | 518 | AY245689 | 97.68 | 517 | 99 | d__Eukaryota;k__Alveolata;p__norank;c__Dinophyceae;o__Peridiniales;f__Pfiesteriaceae;g__unclassified_Pfiesteriaceae;s__Pfiesteria-like_DINO_Lucy |
| OTU186 | 519 | AY245689 | 94.8 | 519 | 100 | d__Eukaryota;k__Alveolata;p__norank;c__Dinophyceae;o__Peridiniales;f__Pfiesteriaceae;g__unclassified_Pfiesteriaceae;s__Pfiesteria-like_DINO_Lucy |
| OTU192 | 499 | AY245689 | 94.59 | 499 | 100 | d__Eukaryota;k__Alveolata;p__norank;c__Dinophyceae;o__Peridiniales;f__Pfiesteriaceae;g__unclassified_Pfiesteriaceae;s__Pfiesteria-like_DINO_Lucy |

| | | | | | | |
|---|---|---|---|---|---|---|
| OTU199 | 525 | AY245689 | 96.88 | 32 | 81 | d__Eukaryota;k__Alveolata;p__norank;c__Dinophyceae;o__Peridiniales;f__Pfiesteriaceae;g__unclassified_Pfiesteriaceae;s__Pfiesteria-like_DINO_Lucy |
| OTU2267 | 516 | JQ247714 | 93.86 | 521 | 100 | d__Eukaryota;k__Alveolata;p__norank;c__Dinophyceae;o__Gonyaulacales;f__Cladopyxidaceae;g__Peridiniella;s__Peridiniella_sp._NC-2011 |
| OTU2316 | 542 | JN934984 | 96.68 | 542 | 100 | d__Eukaryota;k__Alveolata;p__norank;c__Dinophyceae;o__Syndiniales;f__Eudubosquellidae;g__Euduboscquella;s__Euduboscquella_sp._ex_Tintinnopsis_sp._1 |
| OTU2321 | 359 | JN934984 | 94.74 | 361 | 100 | d__Eukaryota;k__Alveolata;p__norank;c__Dinophyceae;o__Syndiniales;f__Eudubosquellidae;g__Euduboscquella;s__Euduboscquella_sp._ex_Tintinnopsis_sp._1 |
| OTU2344 | 540 | JN934994 | 99.63 | 540 | 100 | d__Eukaryota;k__Alveolata;p__norank;c__Dinophyceae;o__Syndiniales;f__Eudubosquellidae;g__Euduboscquella;s__Euduboscquella_sp._ex_Tintinnopsis_sp._2 |
| OTU3368 | 513 | KF751926 | 97.83 | 508 | 99 | d__Eukaryota;k__Alveolata;p__norank;c__Dinophyceae;o__Peridiniales;f__Thoracosphaeraceae;g__Scrippsiella;s__Scrippsiella_sp. |
| OTU3376 | 487 | KF751926 | 94.26 | 488 | 100 | d__Eukaryota;k__Alveolata;p__norank;c__Dinophyceae;o__Peridiniales;f__Thoracosphaeraceae;g__Scrippsiella;s__Scrippsiella_sp. |
| OTU3377 | 425 | KF751926 | 96.47 | 425 | 100 | d__Eukaryota;k__Alveolata;p__norank;c__Dinophyceae;o__Peridiniales;f__Thoracosphaeraceae;g__Scrippsiella;s__Scrippsiella_sp. |
| OTU3379 | 502 | KF751926 | 95.59 | 521 | 100 | d__Eukaryota;k__Alveolata;p__norank;c__Dinophyceae;o__Peridiniales;f__Thoracosphaeraceae;g__Scrippsiella;s__Scrippsiella_sp. |
| OTU3391 | 423 | KF751926 | 95.72 | 421 | 99 | d__Eukaryota;k__Alveolata;p__norank;c__Dinophyceae;o__Peridiniales;f__Thoracosphaeraceae;g__Scrippsiella;s__Scrippsiella_sp. |
| OTU3394 | 510 | AB871535 | 96.86 | 509 | 99 | d__Eukaryota;k__Alveolata;p__norank;c__Dinophyceae;o__Gonyaulacales;f__Crypthecodiniaceae;g__Crypthecodinium;s__Crypthecodinium_sp._ShSu-2013 |
| OTU342 | 481 | AY571374 | 99.5 | 398 | 83 | d__Eukaryota;k__Alveolata;p__norank;c__Dinophyceae;o__Lophodiniales;f__Lophodiniaceae;g__Woloszynskia;s__Woloszynskia_tenuissima-like |
| OTU343 | 476 | AY571374 | 99.52 | 416 | 87 | d__Eukaryota;k__Alveolata;p__norank;c__Dinophyceae;o__Lophodiniales;f__Lophodiniaceae;g__Woloszynskia;s__Woloszynskia_tenuissima-like |
| OTU3467 | 490 | HG005135 | 94.29 | 490 | 100 | d__Eukaryota;k__Alveolata;p__norank;c__Dinophyceae;o__Gymnodiniales;f__Gymnodiniaceae;g__Gymnodinium;s__unclassified_Gymnodinium |
| OTU3509 | 483 | HG005135 | 99.17 | 483 | 100 | d__Eukaryota;k__Alveolata;p__norank;c__Dinophyceae;o__Gymnodiniales;f__Gymnodiniaceae;g__Gymnodinium;s__Gymnodinium_sp. GSSW10 |
| OTU357 | 328 | AY590477 | 98.79 | 330 | 100 | d__Eukaryota;k__Alveolata;p__norank;c__Dinophyceae;o__Peridiniales;f__Pfiesteriaceae;g__unclassified_Pfiesteriaceae;s__Pfiesteriaceae_sp._CCMP1835 |
| OTU3686 | 495 | JX262498 | 75 | 356 | 72 | d__Eukaryota;k__Alveolata;p__norank;c__Dinophyceae;o__Peridiniales; f__Peridiniaceae;g__Pentapharsodinium;s__Pentapharsodinium_sp. |
| OTU3690 | 384 | KJ433986 | 74 | 384 | 100 | d__Eukaryota;k__Alveolata;p__norank;c__Dinophyceae;o__Peridiniales; f__Peridiniaceae; g__Pentapharsodinium; s__Pentapharsodinium_sp. |
| OTU3693 | 403 | KJ433986 | 74 | 113 | 99 | d__Eukaryota;k__Alveolata;p__norank;c__Dinophyceae;o__Peridiniales; f__Peridiniaceae; g__Pentapharsodinium; s__Pentapharsodinium_sp. |
| OTU3694 | 459 | JX262498 | 75 | 66 | 85 | d__Eukaryota;k__Alveolata;p__norank;c__Dinophyceae;o__Peridiniales; f__Peridiniaceae; g__Pentapharsodinium; s__Pentapharsodinium_sp. |

| | | | | | | |
|---|---|---|---|---|---|---|
| OTU3720 | 479 | HG792066 | 100 | 479 | 100 | d__Eukaryota;k__Alveolata;p__norank;c__Dinophyceae;o__unclassified_Dinophyceae;f__norank;g__norank;s__Dinophyceae_sp._1_HJH-2013 |
| OTU3726 | 475 | HG792066 | 96.84 | 475 | 100 | d__Eukaryota;k__Alveolata;p__norank;c__Dinophyceae;o__unclassified_Dinophyceae;f__norank;g__norank;s__Dinophyceae_sp._1_HJH-2013 |
| OTU376 | 505 | AY916546 | 99.8 | 499 | 99 | d__Eukaryota;k__Alveolata;p__norank;c__Dinophyceae;o__Peridiniales;f__Thoracosphaeraceae;g__Scrippsiella;s__Scrippsiella_sp._CS297 |
| OTU3788 | 490 | KJ508397 | 95.74 | 493 | 100 | d__Eukaryota;k__Alveolata;p__norank;c__Dinophyceae;o__Gymnodiniales;f__Warnowiaceae;g__Warnowia;s__Warnowia_sp._IFR1101 |
| OTU379 | 492 | AY916546 | 95.9 | 488 | 99 | d__Eukaryota;k__Alveolata;p__norank;c__Dinophyceae;o__Peridiniales;f__Thoracosphaeraceae;g__Scrippsiella;s__Scrippsiella_sp._CS297 |
| OTU382 | 498 | AY916546 | 96.75 | 492 | 99 | d__Eukaryota;k__Alveolata;p__norank;c__Dinophyceae;o__Peridiniales;f__Thoracosphaeraceae;g__Scrippsiella;s__Scrippsiella_sp._CS297 |
| OTU385 | 499 | AY916546 | 94.93 | 493 | 99 | d__Eukaryota;k__Alveolata;p__norank;c__Dinophyceae;o__Peridiniales;f__Thoracosphaeraceae;g__Scrippsiella;s__Scrippsiella_sp._CS297 |
| OTU388 | 499 | AY916546 | 96.15 | 493 | 99 | d__Eukaryota;k__Alveolata;p__norank;c__Dinophyceae;o__Peridiniales;f__Thoracosphaeraceae;g__Scrippsiella;s__Scrippsiella_sp._CS297 |
| OTU392 | 475 | AY916553 | 96.57 | 466 | 98 | d__Eukaryota;k__Alveolata;p__norank;c__Dinophyceae;o__Lophodiniales;f__Lophodiniaceae;g__Woloszynskia;s__Woloszynskia_sp._CS-341 |
| OTU395 | 448 | AY916553 | 96.88 | 449 | 99 | d__Eukaryota;k__Alveolata;p__norank;c__Dinophyceae;o__Lophodiniales;f__Lophodiniaceae;g__Woloszynskia;s__Woloszynskia_sp._CS-341 |
| OTU396 | 441 | AY916553 | 96.55 | 435 | 99 | d__Eukaryota;k__Alveolata;p__norank;c__Dinophyceae;o__Lophodiniales;f__Lophodiniaceae;g__Woloszynskia;s__Woloszynskia_sp._CS-341 |
| OTU398 | 472 | AY916553 | 96.33 | 463 | 98 | d__Eukaryota;k__Alveolata;p__norank;c__Dinophyceae;o__Lophodiniales;f__Lophodiniaceae;g__Woloszynskia;s__Woloszynskia_sp._CS-341 |
| OTU60 | 498 | AF260393 | 94.19 | 396 | 79 | d__Eukaryota;k__Alveolata;p__norank;c__Dinophyceae;o__Peridiniales;f__Thoracosphaeraceae;g__Scrippsiella;s__Scrippsiella_sp. |

**Table S3.** The most abundant taxa (each with reads >0.9% of the total reads).

| OTU ID | Total reads in 32 samples | Percentage of total reads of 801 OTUs | OUT length | Gi number | Identity | Align length | Coverage | Taxon |
|---|---|---|---|---|---|---|---|---|
| **OTU1321** | **29186** | **7.18%** | **489** | **FJ024705** | **100** | **489** | **100** | **d__Eukaryota;k__Alveolata;p__norank;c__Dinophyceae;o__Suessiales;f__Biecheleriaceae;g__Biecheleria;s__Biecheleria_cincta** |
| OTU2708 | 20465 | 5.04% | 515 | JX262498 | 87.77 | 515 | 99 | d__Eukaryota;k__Alveolata;p__norank;c__Dinophyceae;o__Peridiniales;f__Peridiniaceae;g__Pentapharsodinium;s__Pentapharsodinium_dalei |
| **OTU1775** | **18635** | **4.59%** | **504** | **HM483396** | **96.83** | **504** | **100** | **d__Eukaryota;k__Alveolata;p__norank;c__Dinophyceae;o__Peridiniales;f__Thoracosphaeraceae;g__Scrippsiella;s__Scrippsiella_trochoidea** |
| **OTU3819** | **15455** | **3.80%** | **490** | **KJ450986** | **98.36** | **487** | **99** | **d__Eukaryota;k__Alveolata;p__norank;c__Dinophyceae;o__Peridiniales;f__Peridiniaceae;g__Peridinium;s__Peridinium_baicalense** |
| OTU493 | 14553 | 3.58% | 495 | FJ024705 | 82.02 | 495 | 99 | d__Eukaryota;k__Alveolata;p__norank;c__Dinophyceae;o__Suessiales;f__Biecheleriaceae;g__Biecheleria;s__Biecheleria_cincta |
| OTU638 | 13831 | 3.40% | 485 | AY571374 | 84.63 | 488 | 99 | d__Eukaryota;k__Alveolata;p__norank;c__Dinophyceae;o__Lophodiniales;f__Lophodiniaceae;g__Woloszynskia;s__Woloszynskia_tenuissima |
| **OTU1322** | **13764** | **3.39%** | **497** | **FJ024705** | **98.39** | **497** | **100** | **d__Eukaryota;k__Alveolata;p__norank;c__Dinophyceae;o__Suessiales;f__Biecheleriaceae;g__Biecheleria;s__Biecheleria_cincta** |
| OTU341 | 12008 | 2.96% | 488 | AY571374 | 83.88 | 490 | 99 | d__Eukaryota;k__Alveolata;p__norank;c__Dinophyceae;o__Lophodiniales;f__Lophodiniaceae;g__Woloszynskia;s__Woloszynskia_tenuissima |
| OTU1059 | 11531 | 2.84% | 488 | AY571374 | 84.05 | 489 | 99 | d__Eukaryota;k__Alveolata;p__norank;c__Dinophyceae;o__Lophodiniales;f__Lophodiniaceae;g__Woloszynskia;s__Woloszynskia_tenuissima |
| OTU1996 | 10526 | 2.59% | 482 | EF205002 | 85.9 | 78 | 38 | d__Eukaryota;k__Alveolata;p__norank;c__Dinophyceae;o__norank;f__norank;g__norank;s__Unclassified DINO |
| **OTU1351** | **10101** | **2.49%** | **485** | **FJ024705** | **95.82** | **502** | **99** | **d__Eukaryota;k__Alveolata;p__norank;c__Dinophyceae;o__Suessiales;f__Biecheleriaceae;g__Biecheleria;s__Biecheleria_cincta** |
| OTU338 | 9934 | 2.45% | 492 | AY571374 | 85.25 | 488 | 97 | d__Eukaryota;k__Alveolata;p__norank;c__Dinophyceae;o__Lophodiniales;f__Lophodiniaceae;g__Woloszynskia;s__Woloszynskia_tenuissima |
| **OTU376** | **9438** | **2.32%** | **505** | **AY916546** | **99.8** | **499** | **99** | **d__Eukaryota;k__Alveolata;p__norank;c__Dinophyceae;o__Peridiniales;f__Thoracosphaeraceae;g__Scrippsiella;s__Scrippsiella_sp._CS297** |
| OTU670 | 8007 | 1.97% | 476 | KT389956.1 | 86 | 481 | 100 | d__Eukaryota;k__Alveolata;p__norank;c__Dinophyceae;o__Peridiniales;f__Thoracosphaeraceae;g__Scrippsiella;s__Scrippsiella_sp. |
| **OTU339** | **7625** | **1.88%** | **470** | **AY571374** | **100** | **470** | **100** | **d__Eukaryota;k__Alveolata;p__norank;c__Dinophyceae;o__Lophodiniales;f__Lophodiniaceae;g__Woloszynskia;s__Woloszynskia_tenuissima** |
| **OTU1780** | **7339** | **1.81%** | **499** | **HM483396** | **94.39** | **499** | **100** | **d__Eukaryota;k__Alveolata;p__norank;c__Dinophyceae;o__Peridiniales;f__Thoracosphaeraceae;g__Scrippsiella;s__Scrippsiella_trochoidea** |
| OTU340 | 7103 | 1.75% | 490 | AY571374 | 84.71 | 484 | 98 | d__Eukaryota;k__Alveolata;p__norank;c__Dinophyceae;o__Lophodiniales;f__Lophodiniaceae;g__Woloszynskia;s__Woloszynskia_tenuissima |

| | | | | | | | | |
|---|---|---|---|---|---|---|---|---|
| OTU1926 | 7043 | 1.73% | 502 | HM483399 | 92.87 | 505 | 100 | d__Eukaryota;k__Alveolata;p__norank;c__Dinophyceae;o__unclassified_Dinophyceae;f__norank;g__Duboscquodinium;s__Duboscquodinium_collinii |
| OTU1058 | 6993 | 1.72% | 500 | EU126801 | 77.84 | 528 | 100 | d__Eukaryota;k__Alveolata;p__norank;c__Dinophyceae;o__Suessiales;f__Borghiellaceae;g__Borghiella;s__Borghiella_dodgei |
| **OTU3297** | **6547** | **1.61%** | **499** | **KF878935** | **100** | **499** | **100** | **d__Eukaryota;k__Alveolata;p__norank;c__Dinophyceae;o__Gymnodiniales;f__Gymnodiniaceae;g__Gymnodinium;s__Gymnodinium_instriatum** |
| OTU2723 | 5681 | 1.40% | 527 | JX262498 | 86.55 | 528 | 100 | d__Eukaryota;k__Alveolata;p__norank;c__Dinophyceae;o__Peridiniales;f__Peridiniaceae;g__Pentapharsodinium;s__Pentapharsodinium_dalei |
| OTU2393 | 5472 | 1.35% | 477 | JQ616825 | 92 | 106 | 32 | d__Eukaryota;k__Alveolata;p__norank;c__Dinophyceae;o__Gymnodiniales;f__norank;g__norank;s__Unclassified DINO |
| **OTU351** | **5336** | **1.31%** | **488** | **AY571374** | **96.29** | **485** | **99** | **d__Eukaryota;k__Alveolata;p__norank;c__Dinophyceae;o__Lophodiniales;f__Lophodiniaceae;g__Woloszynskia;s__Woloszynskia_tenuissima** |
| **OTU1332** | **4291** | **1.06%** | **494** | **FJ024705** | **97.57** | **494** | **100** | **d__Eukaryota;k__Alveolata;p__norank;c__Dinophyceae;o__Suessiales;f__Biecheleriaceae;g__Biecheleria;s__Biecheleria_cincta** |
| **OTU323** | **4101** | **1.01%** | **510** | **AY571369** | **98.82** | **510** | **100** | **d__Eukaryota;k__Alveolata;p__norank;c__Dinophyceae;o__Gymnodiniales;f__Gymnodiniaceae;g__Gyrodinium;s__Gyrodinium_rubrum** |
| **OTU489** | **3773** | **0.9%** | **481** | **DQ195368** | **100** | **481** | **100** | **d__Eukaryota;k__Alveolata;p__norank;c__Dinophyceae;o__Suessiales;f__Suessiaceae;g__Pelagodinium;s__Pelagodinium_béii** |
| **OTU3404** | **3685** | **0.9%** | **494** | **AB858353** | **98.38** | **494** | **100** | **d__Eukaryota;k__Alveolata;p__norank;c__Dinophyceae;o__Suessiales;f__Biecheleriaceae;g__Biecheleria;s__Biecheleria_brevisulcata** |
| OTU3721 | 3595 | 0.9% | 505 | LM992906 | 91 | 89 | 72 | d__Eukaryota;k__Alveolata;p__norank;c__Dinophyceae;o__Suessiales;f__Biecheleriaceae;g__norank;s__Biecheleriopsis-like species |
| **OTU3694** | **3553** | **0.9%** | **459** | **JX262498** | **75** | **66** | **85** | **d__Eukaryota;k__Alveolata;p__norank;c__Dinophyceae;o__Peridiniales; f__Peridiniaceae; g__Pentapharsodinium; s__Pentapharsodinium_sp.** |
| **OTU72** | **3478** | **0.9%** | **488** | **AF260394** | **100** | **488** | **100** | **d__Eukaryota;k__Alveolata;p__norank;c__Dinophyceae;o__Peridiniales;f__Peridiniaceae;g__Peridinium;s__Peridinium_palatinum** |

Bold font indicates fully identified species.

**Table S4.** The most frequently detected operational taxonomic units (OTUs) (detected in >50% of the samples) among the 801 OTUs annotated as dinoflagellates.

| OTU ID | Frequency | Percentage of frequency | OUT length | Gi number | Identity | Align length | Coverage | Taxon |
|---|---|---|---|---|---|---|---|---|
| **OTU1321** | **29** | **91%** | **489** | **FJ024705** | **100** | **489** | **100** | **d__Eukaryota;k__Alveolata;p__norank;c__Dinophyceae;o__Suessiales;f__Biecheleriaceae;g__Biecheleria;s__Biecheleria_cincta** |
| **OTU1351** | **29** | **91%** | **485** | **FJ024705** | **95.82** | **502** | **99** | **d__Eukaryota;k__Alveolata;p__norank;c__Dinophyceae;o__Suessiales;f__Biecheleriaceae;g__Biecheleria;s__Biecheleria_cincta** |
| **OTU1322** | **28** | **88%** | **497** | **FJ024705** | **98.39** | **497** | **100** | **d__Eukaryota;k__Alveolata;p__norank;c__Dinophyceae;o__Suessiales;f__Biecheleriaceae;g__Biecheleria;s__Biecheleria_cincta** |
| **OTU1332** | **28** | **88%** | **494** | **FJ024705** | **97.57** | **494** | **100** | **d__Eukaryota;k__Alveolata;p__norank;c__Dinophyceae;o__Suessiales;f__Biecheleriaceae;g__Biecheleria;s__Biecheleria_cincta** |
| **OTU3404** | **27** | **84%** | **494** | **AB858353** | **98.38** | **494** | **100** | **d__Eukaryota;k__Alveolata;p__norank;c__Dinophyceae;o__Suessiales;f__Biecheleriaceae;g__Biecheleria;s__Biecheleria_brevisulcata** |
| OTU2473 | 25 | 78% | 485 | JQ413374 | 98.68 | 303 | 62 | d__Eukaryota;k__Alveolata;p__norank;c__Dinophyceae;o__Suessiales;f__Biecheleriaceae;g__Biecheleria;s__Biecheleria-like_sp. |
| **OTU1343** | **22** | **69%** | **490** | **FJ024705** | **97.56** | **491** | **100** | **d__Eukaryota;k__Alveolata;p__norank;c__Dinophyceae;o__Suessiales;f__Biecheleriaceae;g__Biecheleria;s__Biecheleria_cincta** |
| OTU338 | 21 | 66% | 492 | AY571374 | 85.25 | 488 | 97 | d__Eukaryota;k__Alveolata;p__norank;c__Dinophyceae;o__Lophodiniales;f__Lophodiniaceae;g__Woloszynskia;s__Woloszynskia_tenuissima |
| **OTU2452** | **20** | **63%** | **487** | **FJ024705** | **95.89** | **487** | **100** | **d__Eukaryota;k__Alveolata;p__norank;c__Dinophyceae;o__Suessiales;f__Biecheleriaceae;g__Biecheleria;s__Biecheleria_cincta** |
| OTU2471 | 19 | 59% | 470 | JQ413374 | 98.67 | 300 | 63 | d__Eukaryota;k__Alveolata;p__norank;c__Dinophyceae;o__Suessiales;f__Biecheleriaceae;g__Biecheleria;s__Biecheleria-like_sp. |
| OTU638 | 18 | 56% | 485 | AY571374 | 84.63 | 488 | 99 | d__Eukaryota;k__Alveolata;p__norank;c__Dinophyceae;o__Lophodiniales;f__Lophodiniaceae;g__Woloszynskia;s__Woloszynskia_tenuissima |
| **OTU489** | **18** | **56%** | **481** | **DQ195368** | **100** | **481** | **100** | **d__Eukaryota;k__Alveolata;p__norank;c__Dinophyceae;o__Suessiales;f__Suessiaceae;g__Pelagodinium;s__Pelagodinium_béii** |
| **OTU488** | **17** | **53%** | **492** | **DQ195367** | **98.14** | **484** | **98** | **d__Eukaryota;k__Alveolata;p__norank;c__Dinophyceae;o__Suessiales;f__Suessiaceae;g__Pelagodinium;s__Pelagodinium_béii** |
| OTU647 | 16 | 50% | 495 | AY571374 | 81.62 | 457 | 91 | d__Eukaryota;k__Alveolata;p__norank;c__Dinophyceae;o__Lophodiniales;f__Lophodiniaceae;g__Woloszynskia;s__Woloszynskia-like_sp. |
| **OTU3426** | **16** | **50%** | **496** | **AB858353** | **93.75** | **496** | **100** | **d__Eukaryota;k__Alveolata;p__norank;c__Dinophyceae;o__Suessiales;f__Biecheleriaceae;g__Biecheleria;s__Biecheleria_brevisulcata** |
| **OTU2463** | **16** | **50%** | **404** | **JQ413374** | **97.28** | **404** | **100** | **d__Eukaryota;k__Alveolata;p__norank;c__Dinophyceae;o__Suessiales;f__Biecheleriaceae;g__Biecheleria;s__Biecheleria_cincta** |

Bold font indicates fully identified species.

**Table S5.** The most rare taxa among the 801 operational taxonomic units (OTUs) annotated as dinoflagellates (detected with 1 or 2 reads).

| OTU ID | Reads | OUT length | Gi number | Identity | Align length | Coverage | Taxon |
|---|---|---|---|---|---|---|---|
| OTU1066 | 1 | 507 | JN119844 | 76.14 | 549 | 100 | d__Eukaryota;k__Alveolata;p__norank;c__Dinophyceae;o__Peridiniales;f__Heterocapsaceae;g__Heterocapsa;s__Heterocapsa_sp._HCBC88 |
| **OTU1151** | **1** | **261** | **EU165289** | **98.43** | **255** | **98** | **d__Eukaryota;k__Alveolata;p__norank;c__Dinophyceae;o__Peridiniales;f__Thoracosphaeraceae;g__Scrippsiella;s__Scrippsiella_sp._CCMP2775** |
| OTU1183 | 1 | 487 | AY154965 | 74.64 | 414 | 81 | d__Eukaryota;k__Alveolata;p__norank;c__Dinophyceae;o__Gonyaulacales;f__Gonyaulacaceae;g__Gonyaulax;s__Gonyaulax-like_sp. |
| OTU12 | 1 | 523 | EU126801 | 74.54 | 538 | 100 | d__Eukaryota;k__Alveolata;p__norank;c__Dinophyceae;o__Suessiales;f__Borghiellaceae;g__Borghiella;s__Borghiella_sp. |
| OTU1328 | 1 | 510 | FJ024705 | 91.73 | 508 | 99 | d__Eukaryota;k__Alveolata;p__norank;c__Dinophyceae;o__Suessiales;f__Biecheleriaceae;g__Biecheleria;s__Biecheleria_cincta |
| **OTU1337** | **1** | **499** | **FJ024705** | **94.19** | **499** | **100** | **d__Eukaryota;k__Alveolata;p__norank;c__Dinophyceae;o__Suessiales;f__Biecheleriaceae;g__Biecheleria;s__Biecheleria_cincta** |
| OTU1347 | 1 | 499 | FJ024705 | 92.22 | 501 | 100 | d__Eukaryota;k__Alveolata;p__norank;c__Dinophyceae;o__Suessiales;f__Biecheleriaceae;g__Biecheleria;s__Biecheleria_cincta |
| OTU1360 | 1 | 491 | FJ024705 | 92.68 | 492 | 100 | d__Eukaryota;k__Alveolata;p__norank;c__Dinophyceae;o__Suessiales;f__Biecheleriaceae;g__Biecheleria;s__Biecheleria_cincta |
| OTU1520 | 1 | 484 | FJ939579 | 72.27 | 494 | 99 | d__Eukaryota;k__Alveolata;p__norank;c__Dinophyceae;o__Gonyaulacales;f__Gonyaulacaceae;g__Gonyaulax;s__Gonyaulax_sp. |
| OTU1607 | 1 | 503 | FJ600087 | 88.66 | 529 | 100 | d__Eukaryota;k__Alveolata;p__norank;c__Dinophyceae;o__Peridiniales;f__Pfiesteriaceae;g__Pfiesteria;s__Pfiesteria_piscicida |
| OTU1620 | 1 | 334 | LC027066 | 82.08 | 106 | 32 | d__Eukaryota;k__Alveolata;p__norank;c__Dinophyceae;o__norank;f__norank;g__norank;s__Unclassified DINO |
| OTU1776 | 1 | 482 | HM483396 | 92.95 | 482 | 100 | d__Eukaryota;k__Alveolata;p__norank;c__Dinophyceae;o__Peridiniales;f__Thoracosphaeraceae;g__Scrippsiella;s__Scrippsiella_trochoidea |
| OTU1799 | 1 | 503 | HM483396 | 89.66 | 503 | 100 | d__Eukaryota;k__Alveolata;p__norank;c__Dinophyceae;o__Peridiniales;f__Thoracosphaeraceae;g__Scrippsiella;s__Scrippsiella_trochoidea |
| **OTU1842** | **1** | **516** | **HM483396** | **94.79** | **518** | **100** | **d__Eukaryota;k__Alveolata;p__norank;c__Dinophyceae;o__Peridiniales;f__Thoracosphaeraceae;g__Scrippsiella;s__Scrippsiella_trochoidea** |
| OTU1879 | 1 | 436 | HM483396 | 89.33 | 431 | 99 | d__Eukaryota;k__Alveolata;p__norank;c__Dinophyceae;o__Peridiniales;f__Thoracosphaeraceae;g__Scrippsiella;s__Scrippsiella_trochoidea |
| OTU1897 | 1 | 498 | HM483396 | 90.56 | 498 | 100 | d__Eukaryota;k__Alveolata;p__norank;c__Dinophyceae;o__Peridiniales;f__Thoracosphaeraceae;g__Scrippsiella;s__Scrippsiella_trochoidea |
| **OTU1912** | **1** | **507** | **HM483396** | **95.87** | **508** | **100** | **d__Eukaryota;k__Alveolata;p__norank;c__Dinophyceae;o__Peridiniales;f__Thoracosphaeraceae;g__Scrippsiella;s__Scrippsiella_trochoidea** |
| OTU2000 | 1 | 494 | FR720082 | 80.72 | 498 | 100 | d__Eukaryota;k__Alveolata;p__norank;c__Dinophyceae;o__Gymnodiniales;f__Gymnodiniaceae;g__Gyrodiniellum;s__Gyrodiniellum_sp. |

| | | | | | | | |
|---|---|---|---|---|---|---|---|
| OTU2044 | 1 | 502 | HQ902267 | 82.9 | 503 | 100 | d__Eukaryota;k__Alveolata;p__norank;c__Dinophyceae;o__Peridiniales;f__Heterocapsaceae;g__Heterocapsa;s__Heterocapsa_triquetra |
| OTU2068 | 1 | 502 | JF430394 | 92.63 | 502 | 100 | d__Eukaryota;k__Alveolata;p__norank;c__Dinophyceae;o__Peridiniales;f__Pfiesteriaceae;g__Chimonodinium;s__Chimonodinium_lomnickii |
| OTU210 | 1 | 426 | EU165272 | 80.12 | 322 | 75 | d__Eukaryota;k__Alveolata;p__norank;c__Dinophyceae;o__Peridiniales;f__Heterocapsaceae;g__unclassified_Heterocapsaceae;s__Heterocapsaceae-like sp. |
| **OTU2110** | **1** | **436** | **HQ670228** | **95.86** | **435** | **99** | **d__Eukaryota;k__Alveolata;p__norank;c__Dinophyceae;o__Peridiniales;f__Thoracosphaeraceae;g__Scrippsiella;s__Scrippsiella_trochoidea** |
| **OTU2188** | **1** | **494** | **FN647674** | **98.1** | **421** | **85** | **d__Eukaryota;k__Alveolata;p__norank;c__Dinophyceae;o__Gymnodiniales;f__Gymnodiniaceae;g__Barrufeta;s__Barrufeta_bravensis** |
| OTU2196 | 1 | 479 | DQ195375 | 75.21 | 480 | 99 | d__Eukaryota;k__Alveolata;p__norank;c__Dinophyceae;o__Suessiales;f__Suessiaceae;g__Pelagodinium;s__Pelagodinium_sp. |
| **OTU2202** | **1** | **478** | **JN558107** | **96.44** | **478** | **100** | **d__Eukaryota;k__Alveolata;p__norank;c__Dinophyceae;o__Suessiales;f__Suessiaceae;g__Pelagodinium;s__Pelagodinium_béii** |
| **OTU2316** | **1** | **542** | **JN934984** | **96.68** | **542** | **100** | **d__Eukaryota;k__Alveolata;p__norank;c__Dinophyceae;o__Syndiniales;f__Euduboscquellidae;g__Euduboscquella;s__Euduboscquella_sp._ex_Tintinnopsis_sp._1** |
| **OTU2321** | **1** | **359** | **JN934984** | **94.74** | **361** | **100** | **d__Eukaryota;k__Alveolata;p__norank;c__Dinophyceae;o__Syndiniales;f__Euduboscquellidae;g__Euduboscquella;s__Euduboscquella_sp._ex_Tintinnopsis_sp._1** |
| **OTU2325** | **1** | **561** | **JN934988** | **96.8** | **563** | **100** | **d__Eukaryota;k__Alveolata;p__norank;c__Dinophyceae;o__Syndiniales;f__Euduboscquellidae;g__Euduboscquella;s__Euduboscquella_cachoni** |
| OTU2332 | 1 | 498 | JN934989 | 69.26 | 501 | 99 | d__Eukaryota;k__Alveolata;p__norank;c__Dinophyceae;o__Syndiniales;f__Euduboscquellidae;g__Euduboscquella;s__Euduboscquella_sp._ex_Favella_arcuata |
| **OTU2344** | **1** | **540** | **JN934994** | **99.63** | **540** | **100** | **d__Eukaryota;k__Alveolata;p__norank;c__Dinophyceae;o__Syndiniales;f__Euduboscquellidae;g__Euduboscquella;s__Euduboscquella_sp._ex_Tintinnopsis_sp._2** |
| OTU2428 | 1 | 235 | JQ639752 | 85.71 | 189 | 80 | d__Eukaryota;k__Alveolata;p__norank;c__Dinophyceae;o__Peridiniales;f__Glenodiniaceae;g__Peridiniopsis;s__Peridiniopsis_minima |
| OTU2447 | 1 | 502 | FJ024705 | 88.14 | 506 | 100 | d__Eukaryota;k__Alveolata;p__norank;c__Dinophyceae;o__Suessiales;f__Biecheleriaceae;g__Biecheleria;s__Biecheleria_cincta |
| OTU2451 | 1 | 481 | FJ024705 | 91.12 | 484 | 100 | d__Eukaryota;k__Alveolata;p__norank;c__Dinophyceae;o__Suessiales;f__Biecheleriaceae;g__Biecheleria;s__Biecheleria_cincta |
| **OTU2456** | **1** | **444** | **JQ413374** | **96.26** | **454** | **100** | **d__Eukaryota;k__Alveolata;p__norank;c__Dinophyceae;o__Suessiales;f__Biecheleriaceae;g__Biecheleria;s__Biecheleria_cincta** |
| OTU2458 | 1 | 505 | AB858353 | 91.68 | 505 | 100 | d__Eukaryota;k__Alveolata;p__norank;c__Dinophyceae;o__Suessiales;f__Biecheleriaceae;g__Biecheleria;s__Biecheleria_brevisulcata |
| OTU2537 | 1 | 477 | HQ845327 | 88 | 84 | 23 | d__Eukaryota;k__Alveolata;p__norank;c__Dinophyceae;o__norank;f__norank;g__norank;s__Unclassified DINO |

| | | | | | | | |
|---|---|---|---|---|---|---|---|
| OTU2583 | 1 | 464 | AF260400 | 71.22 | 476 | 99 | d__Eukaryota;k__Alveolata;p__norank;c__Dinophyceae;o__Peridiniales;f__Heterocapsaceae;g__Heterocapsa;s__Heterocapsa_rotundata |
| OTU2641 | 1 | 299 | JN982402 | 97.57 | 288 | 96 | d__Eukaryota;k__Alveolata;p__norank;c__Dinophyceae;o__Peridiniales;f__Thoracosphaeraceae;g__Scrippsiella;s__Scrippsiella_sp. |
| OTU2666 | 1 | 490 | AB716928 | 91.65 | 491 | 100 | d__Eukaryota;k__Alveolata;p__norank;c__Dinophyceae;o__Peridiniales;f__Protoperidiniaceae;g__Protoperidinium;s__Protoperidinium_monovelum |
| OTU2669 | 1 | 359 | AB716928 | 86.35 | 359 | 100 | d__Eukaryota;k__Alveolata;p__norank;c__Dinophyceae;o__Peridiniales;f__Protoperidiniaceae;g__Protoperidinium;s__Protoperidinium_monovelum |
| **OTU2711** | **1** | **473** | **JX262498** | **73** | **473** | **100** | **d__Eukaryota;k__Alveolata;p__norank;c__Dinophyceae;o__Peridiniales;f__Peridiniaceae;g__Pentapharsodinium;s__Pentapharsodinium_sp.** |
| **OTU2808** | **1** | **219** | **KC895475** | **96.36** | **220** | **100** | **d__Eukaryota;k__Alveolata;p__norank;c__Dinophyceae;o__Suessiales;f__Biecheleriaceae;g__Biecheleria;s__Biecheleria_cincta** |
| OTU2856 | 1 | 478 | KP790181 | 89.42 | 104 | 22 | d__Eukaryota;k__Alveolata;p__norank;c__Dinophyceae;o__norank;f__norank;g__norank;s__Unclassified DINO |
| **OTU2953** | **1** | **491** | **KF031312** | **99.32** | **443** | **90** | **d__Eukaryota;k__Alveolata;p__norank;c__Dinophyceae;o__Peridiniales;f__Heterocapsaceae;g__Heterocapsa;s__Heterocapsa_minima** |
| OTU3287 | 1 | 499 | EF616462 | 86.07 | 524 | 100 | d__Eukaryota;k__Alveolata;p__norank;c__Dinophyceae;o__Gymnodiniales;f__Gymnodiniaceae;g__Cochlodinium;s__Cochlodinium_cf._geminatum |
| OTU3288 | 1 | 500 | HQ834210 | 84.79 | 539 | 100 | d__Eukaryota;k__Alveolata;p__norank;c__Dinophyceae;o__Gymnodiniales;f__Polykrikaceae;g__Pheopolykrikos;s__Pheopolykrikos_hartmannii |
| OTU3328 | 1 | 499 | HM483396 | 92.99 | 499 | 100 | d__Eukaryota;k__Alveolata;p__norank;c__Dinophyceae;o__Peridiniales;f__Thoracosphaeraceae;g__Scrippsiella;s__Scrippsiella_trochoidea |
| OTU3335 | 1 | 519 | KF751924 | 89.21 | 519 | 99 | d__Eukaryota;k__Alveolata;p__norank;c__Dinophyceae;o__Peridiniales;f__Thoracosphaeraceae;g__Scrippsiella;s__Scrippsiella_aff._acuminata |
| OTU334 | 1 | 497 | AF260392 | 86.97 | 499 | 100 | d__Eukaryota;k__Alveolata;p__norank;c__Dinophyceae;o__Peridiniales;f__Thoracosphaeraceae;g__Scrippsiella;s__Scrippsiella_sp. |
| OTU3365 | 1 | 410 | KF751924 | 90.64 | 406 | 99 | d__Eukaryota;k__Alveolata;p__norank;c__Dinophyceae;o__Peridiniales;f__Thoracosphaeraceae;g__Scrippsiella;s__Scrippsiella_aff._acuminata |
| **OTU3377** | **1** | **425** | **KF751926** | **96.47** | **425** | **100** | **d__Eukaryota;k__Alveolata;p__norank;c__Dinophyceae;o__Peridiniales;f__Thoracosphaeraceae;g__Scrippsiella;s__Scrippsiella_sp.** |
| **OTU3385** | **1** | **495** | **HM483396** | **94.34** | **495** | **100** | **d__Eukaryota;k__Alveolata;p__norank;c__Dinophyceae;o__Peridiniales;f__Thoracosphaeraceae;g__Scrippsiella;s__Scrippsiella_trochoidea** |
| OTU3407 | 1 | 501 | AB858353 | 88.42 | 501 | 100 | d__Eukaryota;k__Alveolata;p__norank;c__Dinophyceae;o__Suessiales;f__Biecheleriaceae;g__Biecheleria;s__Biecheleria_brevisulcata |
| **OTU3408** | **1** | **225** | **AB858353** | **99.06** | **213** | **94** | **d__Eukaryota;k__Alveolata;p__norank;c__Dinophyceae;o__Suessiales;f__Biecheleriaceae;g__Biecheleria;s__Biecheleria_brevisulcata** |
| OTU3413 | 1 | 495 | AB858353 | 89.54 | 497 | 100 | d__Eukaryota;k__Alveolata;p__norank;c__Dinophyceae;o__Suessiales;f__Biecheleriaceae;g__Biecheleria;s__Biecheleria_brevisulcata |
| **OTU342** | **1** | **481** | **AY571374** | **99.5** | **398** | **83** | **d__Eukaryota;k__Alveolata;p__norank;c__Dinophyceae;o__Lophodiniales;f__Lophodiniaceae;g__Woloszynskia;s__Woloszynskia_tenuissima-like** |

| | | | | | | | |
|---|---|---|---|---|---|---|---|
| OTU3443 | 1 | 489 | LM992906 | 91.46 | 492 | 100 | d__Eukaryota;k__Alveolata;p__norank;c__Dinophyceae;o__Lophodiniales;f__Lophodiniaceae;g__Biecheleriopsis;s__Biecheleriopsis_adriatica |
| OTU3490 | 1 | 501 | HG005135 | 92.38 | 499 | 99 | d__Eukaryota;k__Alveolata;p__norank;c__Dinophyceae;o__Gymnodiniales;f__Gymnodiniaceae;g__Gymnodinium;s__unclassified_Gymnodinium |
| **OTU3509** | **1** | **483** | **HG005135** | **99.17** | **483** | **100** | **d__Eukaryota;k__Alveolata;p__norank;c__Dinophyceae;o__Gymnodiniales;f__Gymnodiniaceae;g__Gymnodinium;s__Gymnodinium_sp. GSSW10** |
| OTU3530 | 1 | 497 | KJ433986 | 87.62 | 501 | 99 | d__Eukaryota;k__Alveolata;p__norank;c__Dinophyceae;o__Peridiniales;f__Peridiniaceae;g__Pentapharsodinium;s__Pentapharsodinium_sp._ZL-2014 |
| OTU3535 | 1 | 437 | KJ433986 | 90.13 | 476 | 100 | d__Eukaryota;k__Alveolata;p__norank;c__Dinophyceae;o__Peridiniales;f__Peridiniaceae;g__Pentapharsodinium;s__Pentapharsodinium_sp._ZL-2014 |
| OTU3538 | 1 | 466 | KJ433986 | 90.79 | 467 | 100 | d__Eukaryota;k__Alveolata;p__norank;c__Dinophyceae;o__Peridiniales;f__Peridiniaceae;g__Pentapharsodinium;s__Pentapharsodinium_sp._ZL-2014 |
| **OTU355** | **1** | **505** | **DQ991376** | **95.86** | **507** | **100** | **d__Eukaryota;k__Alveolata;p__norank;c__Dinophyceae;o__Peridiniales;f__Pfiesteriaceae;g__Cryptoperidiniopsis;s__Cryptoperidiniopsis_brodyi** |
| **OTU3602** | **1** | **486** | **KP790206** | **97.24** | **434** | **89** | **d__Eukaryota;k__Alveolata;p__norank;c__Dinophyceae;o__Gymnodiniales;f__Gymnodiniaceae;g__Gyrodinium;s__Gyrodinium_undulans** |
| **OTU3686** | **1** | **495** | **JX262498** | **75** | **356** | **72** | **d__Eukaryota;k__Alveolata;p__norank;c__Dinophyceae;o__Peridiniales; f__Peridiniaceae;g__Pentapharsodinium;s__Pentapharsodinium_sp.** |
| **OTU3726** | **1** | **475** | **HG792066** | **96.84** | **475** | **100** | **d__Eukaryota;k__Alveolata;p__norank;c__Dinophyceae;o__unclassified_Dinophyceae;f__norank;g__norank;s__Dinophyceae_sp._1_HJH-2013** |
| **OTU3757** | **1** | **480** | **LC027049** | **98.34** | **481** | **100** | **d__Eukaryota;k__Alveolata;p__norank;c__Dinophyceae;o__unclassified_Dinophyceae;f__norank;g__Pellucidodinium;s__Pellucidodinium_psammophilum** |
| OTU3759 | 1 | 478 | LC027049 | 89.77 | 479 | 99 | d__Eukaryota;k__Alveolata;p__norank;c__Dinophyceae;o__unclassified_Dinophyceae;f__norank;g__Pellucidodinium;s__Pellucidodinium_psammophilum |
| OTU3831 | 1 | 498 | JX262498 | 83.03 | 501 | 100 | d__Eukaryota;k__Alveolata;p__norank;c__Dinophyceae;o__Peridiniales;f__Peridiniaceae;g__Pentapharsodinium;s__Pentapharsodinium_dalei |
| **OTU385** | **1** | **499** | **AY916546** | **94.93** | **493** | **99** | **d__Eukaryota;k__Alveolata;p__norank;c__Dinophyceae;o__Peridiniales;f__Thoracosphaeraceae;g__Scrippsiella;s__Scrippsiella_sp._CS297** |
| **OTU398** | **1** | **472** | **AY916553** | **96.33** | **463** | **98** | **d__Eukaryota;k__Alveolata;p__norank;c__Dinophyceae;o__Lophodiniales;f__Lophodiniaceae;g__Woloszynskia;s__Woloszynskia_sp._CS-341** |
| **OTU4** | **1** | **259** | **KF245459** | **79.28** | **111** | **43** | **d__Eukaryota;k__Alveolata;p__norank;c__Dinophyceae;o__unclassified;f__unclassified;g__unclassified;s__unclassified DINO** |
| OTU470 | 1 | 378 | AY154965 | 73.74 | 396 | 99 | d__Eukaryota;k__Alveolata;p__norank;c__Dinophyceae;o__Gonyaulacales;f__Gonyaulacaceae;g__Gonyaulax;s__Gonyaulax_membranacea |
| OTU475 | 1 | 411 | AY154965 | 73.26 | 430 | 99 | d__Eukaryota;k__Alveolata;p__norank;c__Dinophyceae;o__Gonyaulacales;f__Gonyaulacaceae;g__Gonyaulax;s__Gonyaulax_membranacea |
| **OTU499** | **1** | **447** | **DQ195376** | **96.65** | **448** | **100** | **d__Eukaryota;k__Alveolata;p__norank;c__Dinophyceae;o__Suessiales;f__Suessiaceae;g__Pelagodinium;s__Pelagodinium_béii** |
| OTU535 | 1 | 435 | AM408889 | 79.27 | 439 | 100 | d__Eukaryota;k__Alveolata;p__norank;c__Dinophyceae;o__Gymnodiniales;f__Gymnodiniaceae;g__Paragymnodinium;s__Paragymnodinium_shiwhaense |

| | | | | | | | |
|---|---|---|---|---|---|---|---|
| **OTU58** | **1** | **505** | **AF260393** | **97.23** | **505** | **100** | **d__Eukaryota;k__Alveolata;p__norank;c__Dinophyceae;o__Peridiniales;f__Thoracosphaeraceae;g__Scrippsiella;s__Scrippsiella_trochoidea** |
| **OTU60** | **1** | **498** | **AF260393** | **94.19** | **396** | **79** | **d__Eukaryota;k__Alveolata;p__norank;c__Dinophyceae;o__Peridiniales;f__Thoracosphaeraceae;g__Scrippsiella;s__Scrippsiella_sp.** |
| **OTU62** | **1** | **413** | **AF260393** | **95.27** | **402** | **97** | **d__Eukaryota;k__Alveolata;p__norank;c__Dinophyceae;o__Peridiniales;f__Thoracosphaeraceae;g__Scrippsiella;s__Scrippsiella_trochoidea** |
| OTU702 | 1 | 527 | EF152961 | 90.74 | 529 | 100 | d__Eukaryota;k__Alveolata;p__norank;c__Dinophyceae;o__Peridiniales;f__Diplopsaliaceae;g__Preperidinium;s__Protoperidinium_cf._steidingerae |
| **OTU71** | **1** | **341** | **AF260393** | **96.44** | **337** | **99** | **d__Eukaryota;k__Alveolata;p__norank;c__Dinophyceae;o__Peridiniales;f__Thoracosphaeraceae;g__Scrippsiella;s__Scrippsiella_trochoidea** |
| OTU719 | 1 | 517 | EF152961 | 88.87 | 557 | 99 | d__Eukaryota;k__Alveolata;p__norank;c__Dinophyceae;o__Peridiniales;f__Diplopsaliaceae;g__Preperidinium;s__Protoperidinium_cf._steidingerae |
| **OTU1043** | **2** | **432** | **EU048553** | **97.92** | **433** | **100** | **d__Eukaryota;k__Alveolata;p__norank;c__Dinophyceae;o__Peridiniales;f__Pfiesteriaceae;g__unclassified_Pfiesteriaceae;s__Pfiesteriaceae_sp._masanensis** |
| **OTU105** | **2** | **251** | **AF318248** | **96.41** | **251** | **99** | **d__Eukaryota;k__Alveolata;p__norank;c__Dinophyceae;o__Gymnodiniales;f__Gymnodiniaceae;g__Gymnodinium;s__unclassified_Gymnodinium** |
| OTU1091 | 2 | 514 | HQ902267 | 76.44 | 556 | 100 | d__Eukaryota;k__Alveolata;p__norank;c__Dinophyceae;o__Peridiniales;f__Heterocapsaceae;g__Heterocapsa;s__Heterocapsa_triquetra |
| OTU110 | 2 | 413 | KF031312 | 76.43 | 420 | 99 | d__Eukaryota;k__Alveolata;p__norank;c__Dinophyceae;o__Peridiniales;f__Heterocapsaceae;g__Heterocapsa;s__Heterocapsa_minima |
| **OTU1150** | **2** | **317** | **EU165289** | **97.16** | **317** | **100** | **d__Eukaryota;k__Alveolata;p__norank;c__Dinophyceae;o__Peridiniales;f__Thoracosphaeraceae;g__Scrippsiella;s__Scrippsiella_sp._CCMP2775** |
| **OTU1158** | **2** | **236** | **HQ845331** | **94.92** | **236** | **100** | **d__Eukaryota;k__Alveolata;p__norank;c__Dinophyceae;o__Peridiniales;f__Thoracosphaeraceae;g__Scrippsiella;s__Scrippsiella_sweeneyae** |
| **OTU1178** | **2** | **487** | **HQ670228.1** | **98** | **487** | **100** | **d__Eukaryota;k__Alveolata;p__norank;c__Dinophyceae;o__Peridiniales;f__Thoracosphaeraceae;g__Scrippsiella;s__Scrippsiella_trochoidea** |
| **OTU1180** | **2** | **295** | **EU370959** | **96.96** | **296** | **100** | **d__Eukaryota;k__Alveolata;p__norank;c__Dinophyceae;o__Peridiniales;f__Thoracosphaeraceae;g__Scrippsiella;s__Scrippsiella_trochoidea** |
| **OTU1329** | **2** | **508** | **FJ024705** | **95.44** | **504** | **99** | **d__Eukaryota;k__Alveolata;p__norank;c__Dinophyceae;o__Suessiales;f__Biecheleriaceae;g__Biecheleria;s__Biecheleria_cincta** |
| **OTU1339** | **2** | **485** | **FJ024705** | **94.02** | **485** | **100** | **d__Eukaryota;k__Alveolata;p__norank;c__Dinophyceae;o__Suessiales;f__Biecheleriaceae;g__Biecheleria;s__Biecheleria_cincta** |
| OTU1346 | 2 | 489 | FJ024705 | 91.85 | 491 | 100 | d__Eukaryota;k__Alveolata;p__norank;c__Dinophyceae;o__Suessiales;f__Biecheleriaceae;g__Biecheleria;s__Biecheleria_cincta |
| OTU1356 | 2 | 493 | FJ024705 | 93.1 | 493 | 100 | d__Eukaryota;k__Alveolata;p__norank;c__Dinophyceae;o__Suessiales;f__Biecheleriaceae;g__Biecheleria;s__Biecheleria_cincta |
| **OTU1359** | **2** | **492** | **FJ024705** | **95.53** | **492** | **100** | **d__Eukaryota;k__Alveolata;p__norank;c__Dinophyceae;o__Suessiales;f__Biecheleriaceae;g__Biecheleria;s__Biecheleria_cincta** |
| OTU1361 | 2 | 491 | FJ024705 | 90.84 | 491 | 100 | d__Eukaryota;k__Alveolata;p__norank;c__Dinophyceae;o__Suessiales;f__Biecheleriaceae;g__Biecheleria;s__Biecheleria_cincta |

| | | | | | | | |
|---|---|---|---|---|---|---|---|
| OTU1363 | 2 | 488 | FJ024705 | 93.24 | 488 | 100 | d__Eukaryota;k__Alveolata;p__norank;c__Dinophyceae;o__Suessiales;f__Biecheleriaceae;g__Biecheleria;s__Biecheleria_cincta |
| OTU1613 | 2 | 461 | EF205003 | 83.72 | 86 | 53 | d__Eukaryota;k__Alveolata;p__norank;c__Dinophyceae;o__norank;f__norank;g__norank;s__Unclassified DINO |
| OTU1695 | 2 | 507 | LC002848 | 82.09 | 402 | 79 | d__Eukaryota;k__Alveolata;p__norank;c__Dinophyceae;o__unclassified_Dinophyceae;f__norank;g__norank;s__Unclassified DINO |
| **OTU1794** | **2** | **499** | **HM483396** | **96.79** | **498** | **99** | **d__Eukaryota;k__Alveolata;p__norank;c__Dinophyceae;o__Peridiniales;f__Thoracosphaeraceae;g__Scrippsiella;s__Scrippsiella_trochoidea** |
| OTU1853 | 2 | 494 | EF152961 | 91.5 | 494 | 100 | d__Eukaryota;k__Alveolata;p__norank;c__Dinophyceae;o__Peridiniales;f__Diplopsaliaceae;g__Preperidinium;s__Protoperidinium_cf._steidingerae |
| OTU1884 | 2 | 489 | HM483396 | 92.02 | 489 | 100 | d__Eukaryota;k__Alveolata;p__norank;c__Dinophyceae;o__Peridiniales;f__Thoracosphaeraceae;g__Scrippsiella;s__Scrippsiella_trochoidea |
| OTU2042 | 2 | 512 | HQ902267 | 82.91 | 515 | 99 | d__Eukaryota;k__Alveolata;p__norank;c__Dinophyceae;o__Peridiniales;f__Heterocapsaceae;g__Heterocapsa;s__Heterocapsa_triquetra |
| **OTU2059** | **2** | **416** | **JF521637** | **98.56** | **417** | **100** | **d__Eukaryota;k__Alveolata;p__norank;c__Dinophyceae;o__Gonyaulacales;f__Gonyaulacaceae;g__Alexandrium;s__Alexandrium_ostenfeldii** |
| OTU2078 | 2 | 515 | JN119844 | 84.17 | 518 | 100 | d__Eukaryota;k__Alveolata;p__norank;c__Dinophyceae;o__Peridiniales;f__Heterocapsaceae;g__Heterocapsa;s__Heterocapsa_sp._HCBC88 |
| OTU236 | 2 | 473 | AY154960 | 81.54 | 65 | 47 | d__Eukaryota;k__Alveolata;p__norank;c__Dinophyceae;o__Gonyaulacales;f__Gonyaulacaceae;g__norank;s__Unclassified DINO |
| **OTU240** | **2** | **436** | **AY154960** | **96.45** | **422** | **97** | **d__Eukaryota;k__Alveolata;p__norank;c__Dinophyceae;o__Gonyaulacales;f__Gonyaulacaceae;g__Gonyaulax;s__Gonyaulax_cf._spinifera** |
| OTU2407 | 2 | 494 | JQ616825 | 76.41 | 496 | 99 | d__Eukaryota;k__Alveolata;p__norank;c__Dinophyceae;o__Gymnodiniales;f__Gymnodiniaceae;g__Gymnodinium;s__Gymnodinium_catenatum |
| OTU2446 | 2 | 507 | FJ024705 | 89.11 | 505 | 99 | d__Eukaryota;k__Alveolata;p__norank;c__Dinophyceae;o__Suessiales;f__Biecheleriaceae;g__Biecheleria;s__Biecheleria_cincta |
| OTU2450 | 2 | 492 | FJ024705 | 86.87 | 495 | 100 | d__Eukaryota;k__Alveolata;p__norank;c__Dinophyceae;o__Suessiales;f__Biecheleriaceae;g__Biecheleria;s__Biecheleria_cincta |
| OTU2455 | 2 | 506 | FJ024705 | 88.39 | 508 | 100 | d__Eukaryota;k__Alveolata;p__norank;c__Dinophyceae;o__Suessiales;f__Biecheleriaceae;g__Biecheleria;s__Biecheleria_cincta |
| OTU2467 | 2 | 492 | FJ024705 | 86.49 | 496 | 100 | d__Eukaryota;k__Alveolata;p__norank;c__Dinophyceae;o__Suessiales;f__Biecheleriaceae;g__Biecheleria;s__Biecheleria_cincta |
| OTU2496 | 2 | 517 | KP790208 | 89.47 | 76 | 15 | d__Eukaryota;k__Alveolata;p__norank;c__Dinophyceae;o__norank;f__norank;g__norank;s__Unclassifid DINO |
| **OTU2644** | **2** | **513** | **JX559886** | **99.1** | **442** | **86** | **d__Eukaryota;k__Alveolata;p__norank;c__Dinophyceae;o__Gonyaulacales;f__Amphidomataceae;g__Azadinium;s__Azadinium_polongum** |
| OTU2768 | 2 | 501 | JQ247713 | 73.05 | 512 | 100 | d__Eukaryota;k__Alveolata;p__norank;c__Dinophyceae;o__Peridiniales;f__Heterocapsaceae;g__Heterocapsa;s__Heterocapsa_niei |
| **OTU2965** | **2** | **257** | **KF240778** | **96.96** | **263** | **100** | **d__Eukaryota;k__Alveolata;p__norank;c__Dinophyceae;o__Peridiniales;f__Heterocapsaceae;g__Heterocapsa;s__Heterocapsa_rotundata** |

| | | | | | | | |
|---|---|---|---|---|---|---|---|
| OTU2984 | 2 | 519 | KF543359 | 96.29 | 431 | 83 | d__Eukaryota;k__Alveolata;p__norank;c__Dinophyceae;o__Gonyaulacales;f__Amphidomataceae;g__Azadinium;s__Azadinium_dalianense |
| **OTU3033** | **2** | **509** | **KF651047** | **94.5** | **509** | **100** | **d__Eukaryota;k__Alveolata;p__norank;c__Dinophyceae;o__Peridiniales;f__Protoperidiniaceae;g__Protoperidinium;s__Protoperidinium_tricingulatum** |
| **OTU3344** | **2** | **323** | **KF751924** | **96.6** | **324** | **100** | **d__Eukaryota;k__Alveolata;p__norank;c__Dinophyceae;o__Peridiniales;f__Thoracosphaeraceae;g__Scrippsiella;s__Scrippsiella_aff._acuminata** |
| **OTU3364** | **2** | **279** | **KF751924** | **97.84** | **278** | **99** | **d__Eukaryota;k__Alveolata;p__norank;c__Dinophyceae;o__Peridiniales;f__Thoracosphaeraceae;g__Scrippsiella;s__Scrippsiella_aff._acuminata** |
| OTU337 | 2 | 497 | JX262498 | 89.16 | 498 | 100 | d__Eukaryota;k__Alveolata;p__norank;c__Dinophyceae;o__Peridiniales;f__Peridiniaceae;g__Pentapharsodinium;s__Pentapharsodinium_dalei |
| **OTU3391** | **2** | **423** | **KF751926** | **95.72** | **421** | **99** | **d__Eukaryota;k__Alveolata;p__norank;c__Dinophyceae;o__Peridiniales;f__Thoracosphaeraceae;g__Scrippsiella;s__Scrippsiella_sp.** |
| OTU3402 | 2 | 496 | AB858353 | 92.96 | 497 | 100 | d__Eukaryota;k__Alveolata;p__norank;c__Dinophyceae;o__Suessiales;f__Biecheleriaceae;g__Biecheleria;s__Biecheleria_brevisulcata |
| **OTU3406** | **2** | **515** | **AB858353** | **94.69** | **508** | **99** | **d__Eukaryota;k__Alveolata;p__norank;c__Dinophyceae;o__Suessiales;f__Biecheleriaceae;g__Biecheleria;s__Biecheleria_brevisulcata** |
| **OTU3424** | **2** | **488** | **AB858353** | **95.09** | **489** | **100** | **d__Eukaryota;k__Alveolata;p__norank;c__Dinophyceae;o__Suessiales;f__Biecheleriaceae;g__Biecheleria;s__Biecheleria_brevisulcata** |
| OTU3446 | 2 | 485 | LM992905 | 100 | 339 | 70 | d__Eukaryota;k__Alveolata;p__norank;c__Dinophyceae;o__Lophodiniales;f__Lophodiniaceae;g__Biecheleriopsis;s__Biecheleriopsis_adriatica |
| OTU361 | 2 | 413 | LC027052 | 76.64 | 244 | 58 | d__Eukaryota;k__Alveolata;p__norank;c__Dinophyceae;o__Gymnodiniales;f__Gymnodiniaceae;g__Unclassified;s__Unclassified_Gymnodiniaceae |
| OTU3712 | 2 | 494 | EU490149 | 72.55 | 499 | 98 | d__Eukaryota;k__Alveolata;p__norank; c__Dinophyceae;o__norank;f__norank;g__norank;s__unclassified DINO |
| **OTU379** | **2** | **492** | **AY916546** | **95.9** | **488** | **99** | **d__Eukaryota;k__Alveolata;p__norank;c__Dinophyceae;o__Peridiniales;f__Thoracosphaeraceae;g__Scrippsiella;s__Scrippsiella_sp._CS297** |
| **OTU382** | **2** | **498** | **AY916546** | **96.75** | **492** | **99** | **d__Eukaryota;k__Alveolata;p__norank;c__Dinophyceae;o__Peridiniales;f__Thoracosphaeraceae;g__Scrippsiella;s__Scrippsiella_sp._CS297** |
| **OTU683** | **2** | **455** | **KP702719** | **95.83** | **456** | **100** | **d__Eukaryota;k__Alveolata;p__norank;c__Dinophyceae;o__Peridiniales;f__Diplopsaliaceae;g__Oblea;s__Oblea_rotunda** |
| OTU980 | 2 | 497 | EF616462 | 94.74 | 133 | 90 | d__Eukaryota;k__Alveolata;p__norank;c__Dinophyceae;o__Gymnodiniales;f__Gymnodiniaceae;g__Cochlodinium;s__Cochlodinium_cf._geminatum-like |

Bold font indicates fully identified species.

**Table S6.** The least frequently detected operational taxonomic units (OTUs) (detected in 1 or 2 samples).

| OTU ID | Frequency | OUT length | Gi number | Identity | Align length | Coverage | Taxon |
| --- | --- | --- | --- | --- | --- | --- | --- |
| OTU1059 | 1 | 488 | AY571374 | 84.05 | 489 | 99 | d__Eukaryota;k__Alveolata;p__norank;c__Dinophyceae;o__Lophodiniales;f__Lophodiniaceae;g__Woloszynskia;s__Woloszynskia_tenuissima |
| OTU1498 | 1 | 507 | FJ236464 | 100 | 502 | 99 | d__Eukaryota;k__Alveolata;p__norank;c__Dinophyceae;o__Peridiniales;f__Glenodiniaceae;g__Peridiniopsis;s__Peridiniopsis_borgei |
| OTU1082 | 1 | 509 | EU126801 | 79.62 | 520 | 100 | d__Eukaryota;k__Alveolata;p__norank;c__Dinophyceae;o__Suessiales;f__Borghiellaceae;g__Borghiella;s__Borghiella_dodgei |
| OTU2060 | 1 | 466 | JF521638 | 100 | 466 | 100 | d__Eukaryota;k__Alveolata;p__norank;c__Dinophyceae;o__Gonyaulacales;f__Gonyaulacaceae;g__Alexandrium;s__Alexandrium_pseudogoniaulax |
| OTU1073 | 1 | 501 | AY571374 | 83.83 | 507 | 100 | d__Eukaryota;k__Alveolata;p__norank;c__Dinophyceae;o__Lophodiniales;f__Lophodiniaceae;g__Woloszynskia;s__Woloszynskia_tenuissima |
| OTU3822 | 1 | 501 | AF260392 | 87.08 | 503 | 99 | d__Eukaryota;k__Alveolata;p__norank;c__Dinophyceae;o__Peridiniales;f__Thoracosphaeraceae;g__Scrippsiella;s__Scrippsiella_sp. |
| OTU3697 | 1 | 494 | EU490149 | 73.02 | 504 | 99 | d__Eukaryota;k__Fungi;p__environmental_samples;c__Dinophyceae;o__norank;f__norank;g__norank;s__unclassified DINO |
| OTU1800 | 1 | 504 | HM483396 | 96.63 | 504 | 100 | d__Eukaryota;k__Alveolata;p__norank;c__Dinophyceae;o__Peridiniales;f__Thoracosphaeraceae;g__Scrippsiella;s__Scrippsiella_trochoidea |
| OTU3724 | 1 | 492 | AF260402 | 81.51 | 465 | 100 | d__Eukaryota;k__Alveolata;p__norank;c__Dinophyceae;o__Lophodiniales;f__Lophodiniaceae;g__Woloszynskia;s__Woloszynskia-like_sp. |
| OTU2061 | 1 | 481 | JF521638 | 100 | 398 | 83 | d__Eukaryota;k__Alveolata;p__norank;c__Dinophyceae;o__Gonyaulacales;f__Gonyaulacaceae;g__Alexandrium;s__Alexandrium_pseudogoniaulax |
| OTU776 | 1 | 515 | KT389885.1 | 93 | 88 | 80 | d__Eukaryota;k__Alveolata;p__norank;c__Dinophyceae;o__Gonyaulacales; f__Amphidomataceae;g__norank;s__Unclassified DINO |
| OTU1085 | 1 | 507 | EU126801.1 | 80 | 540 | 100 | d__Eukaryota;k__Alveolata;p__norank;c__Dinophyceae;o__Suessiales;f__Borghiellaceae;g__Borghiella;s__Borghiella_sp. |
| OTU367 | 1 | 484 | AY916538 | 97.49 | 479 | 99 | d__Eukaryota;k__Alveolata;p__norank;c__Dinophyceae;o__Gymnodiniales;f__Gymnodiniaceae;g__Gymnodinium;s__Gymnodinium_microreticulatum |
| OTU695 | 1 | 461 | EF152953 | 91.99 | 462 | 100 | d__Eukaryota;k__Alveolata;p__norank;c__Dinophyceae;o__Peridiniales;f__Diplopsaliaceae;g__Preperidinium;s__Preperidinium_sp._M064sm1 |
| OTU1076 | 1 | 481 | AY571374 | 81.24 | 517 | 100 | d__Eukaryota;k__Alveolata;p__norank;c__Dinophyceae;o__Lophodiniales;f__Lophodiniaceae;g__Woloszynskia;s__Woloszynskia_tenuissima |
| OTU325 | 1 | 499 | AY571369 | 88.6 | 500 | 100 | d__Eukaryota;k__Alveolata;p__norank;c__Dinophyceae;o__Gymnodiniales;f__Gymnodiniaceae;g__Gyrodinium;s__Gyrodinium_rubrum |
| OTU1201 | 1 | 497 | FJ947042 | 86.08 | 510 | 100 | d__Eukaryota;k__Alveolata;p__norank;c__Dinophyceae;o__Gymnodiniales;f__Warnowiaceae;g__Warnowia;s__Warnowia_sp._BSL-2009a |
| OTU3815 | 1 | 446 | KP099821 | 85.71 | 56 | 33 | d__Eukaryota;k__Alveolata;p__norank;c__Dinophyceae;o__environmental_samples;f__norank;g__norank;s__unclassified_DINO |

| | | | | | | | |
|---|---|---|---|---|---|---|---|
| OTU2858 | 1 | 496 | HQ834210 | 78.67 | 497 | 100 | d__Eukaryota;k__Alveolata;p__norank;c__Dinophyceae;o__Gymnodiniales;f__Polykrikaceae;g__Pheopolykrikos;s__Pheopolykrikos_hartmannii-like |
| OTU2264 | 1 | 510 | HQ902267 | 72.46 | 541 | 99 | d__Eukaryota;k__Alveolata;p__norank;c__Dinophyceae;o__Peridiniales;f__Heterocapsaceae;g__Heterocapsa;s__Heterocapsa_triquetra |
| OTU207 | 1 | 488 | KJ670426.1 | 76 | 488 | 100 | d__Eukaryota;k__Alveolata;p__norank;c__Dinophyceae;o__Gymnodiniales;f__Kareniaceae;g__Karlodinium;s__Karlodinium_sp. |
| OTU494 | 1 | 492 | LC068838.1 | 88 | 492 | 100 | d__Eukaryota;k__Alveolata;p__norank;c__Dinophyceae;o__Suessiales;f__Suessiaceae;g__Unclassified;s__Unclassified Suessiaceae |
| OTU2173 | 1 | 499 | LC027067 | 74 | 82 | 50 | d__Eukaryota;k__Alveolata;p__norank;c__Dinophyceae;o__norank;f__norank;g__norank;s__Unclassified DINO |
| OTU2212 | 1 | 489 | JN558110 | 79 | 544 | 97 | d__Eukaryota;k__Alveolata;p__norank;c__Dinophyceae;o__Suessiales;f__Suessiaceae;g__Polarella;s__Polarella_sp. |
| OTU1318 | 1 | 533 | KP790233 | 93.58 | 436 | 82 | d__Eukaryota;k__Alveolata;p__norank;c__Dinophyceae;o__Gymnodiniales;f__Brachidiniaceae;g__Torodinium;s__Torodinium_teredo |
| OTU645 | 1 | 501 | AY571374 | 81.05 | 459 | 90 | d__Eukaryota;k__Alveolata;p__norank;c__Dinophyceae;o__Lophodiniales;f__Lophodiniaceae;g__Woloszynskia;s__Woloszynskia-like_sp. |
| OTU3616 | 1 | 477 | JX262498 | 77.78 | 477 | 100 | d__Eukaryota;k__Alveolata;p__norank;c__Dinophyceae;o__Peridiniales;f__Peridiniaceae;g__Pentapharsodinium;s__Pentapharsodinium_dalei |
| OTU2014 | 1 | 296 | EF205002 | 92.59 | 108 | 36 | d__Eukaryota;k__Alveolata;p__norank;c__Dinophyceae;o__Peridiniales;f__Glenodiniaceae;g__Glenodiniopsis;s__Glenodiniopsis_sp. |
| OTU2027 | 1 | 484 | JX262498 | 91.74 | 484 | 100 | d__Eukaryota;k__Alveolata;p__norank;c__Dinophyceae;o__Peridiniales;f__Peridiniaceae;g__Pentapharsodinium;s__Pentapharsodinium_dalei |
| OTU3698 | 1 | 511 | KF751922 | 95.35 | 86 | 53 | d__Eukaryota;k__Alveolata;p__norank;c__Dinophyceae;o__Peridiniales;f__Thoracosphaeraceae;g__Calciodinellum;s__Calciodinellum_operosum |
| OTU1414 | 1 | 521 | KT390028 | 94.63 | 521 | 100 | d__Eukaryota;k__Alveolata;p__norank;c__Dinophyceae;o__Blastodiniales; f__Blastodiniales;g__Blastodinium-like;s__Blastodinium-like_sp. |
| OTU1319 | 1 | 509 | FJ024703 | 98.43 | 509 | 100 | d__Eukaryota;k__Alveolata;p__norank;c__Dinophyceae;o__Gymnodiniales;f__Kareniaceae;g__Takayama;s__Takayama_acrotrocha |
| OTU1410 | 1 | 531 | KT390028 | 86.96 | 537 | 100 | d__Eukaryota;k__Alveolata;p__norank;c__Dinophyceae;o__Blastodiniales; f__Blastodiniales;g__Blastodinium-like;s__Blastodinium-like_sp. |
| OTU1626 | 1 | 506 | HQ902267 | 80 | 141 | 90 | d__Eukaryota;k__Alveolata;p__norank;c__Dinophyceae;o__Peridiniales;f__Heterocapsaceae;g__Heterocapsa;s__Heterocapsa_sp. |
| OTU3717 | 1 | 543 | KF835600 | 90.18 | 112 | 21 | d__Eukaryota;k__Alveolata;p__norank;c__Dinophyceae;o__Prorocentrales;f__Prorocentraceae;g__Prorocentrum;s__unclassified DINO |
| OTU959 | 1 | 496 | EF469234 | 94.35 | 496 | 100 | d__Eukaryota;k__Alveolata;p__norank;c__Dinophyceae;o__Gymnodiniales;f__Kareniaceae;g__Karlodinium;s__Karlodinium_antarcticum |
| OTU332 | 1 | 505 | AY571371 | 99.21 | 505 | 100 | d__Eukaryota;k__Alveolata;p__norank;c__Dinophyceae;o__Gymnodiniales;f__Gymnodiniaceae;g__Gyrodinium;s__Gyrodinium_spirale |
| OTU2748 | 1 | 490 | JX262498 | 92.67 | 505 | 100 | d__Eukaryota;k__Alveolata;p__norank;c__Dinophyceae;o__Peridiniales;f__Peridiniaceae;g__Pentapharsodinium;s__Pentapharsodinium_dalei |

| | | | | | | | |
|---|---|---|---|---|---|---|---|
| OTU192 | 1 | 499 | AY245689 | 94.59 | 499 | 100 | d__Eukaryota;k__Alveolata;p__norank;c__Dinophyceae;o__Peridiniales;f__Pfiesteriaceae;g__unclassified_Pfiesteriaceae;s__Pfiesteria-like_DINO_Lucy |
| OTU262 | 1 | 455 | AY154960 | 70.06 | 471 | 100 | d__Eukaryota;k__Alveolata;p__norank;c__Dinophyceae;o__Gonyaulacales;f__Gonyaulacaceae;g__Gonyaulax;s__Gonyaulax_sp. |
| OTU3699 | 1 | 506 | EU490149 | 71.49 | 505 | 97 | d__Eukaryota;k__Fungi;p__environmental_samples;c__Dinophyceae;o__norank;f__norank;g__norank;s__unclassified DINO |
| OTU347 | 1 | 487 | AY571374 | 82.92 | 486 | 98 | d__Eukaryota;k__Alveolata;p__norank;c__Dinophyceae;o__Lophodiniales;f__Lophodiniaceae;g__Woloszynskia;s__Woloszynskia_like |
| OTU2343 | 1 | 537 | JN934989 | 76.33 | 545 | 100 | d__Eukaryota;k__Alveolata;p__norank;c__Dinophyceae;o__Syndiniales;f__Euduboscquellidae;g__Euduboscquella;s__Euduboscquella_sp._ex_Favella_arcuata |
| OTU2680 | 1 | 459 | AB716929 | 96.8 | 125 | 59 | d__Eukaryota;k__Alveolata;p__norank;c__Dinophyceae;o__Peridiniales;f__Protoperidiniaceae;g__Protoperidinium;s__Protoperidinium-like_sp. |
| OTU3 | 1 | 385 | FN649409 | 88.79 | 116 | 30 | d__Eukaryota;k__Alveolata;p__norank;c__Dinophyceae;o__unclassified;f__unclassified;g__unclassified;s__unclassified DINO |
| OTU3429 | 1 | 478 | LM992906 | 99.79 | 478 | 100 | d__Eukaryota;k__Alveolata;p__norank;c__Dinophyceae;o__Lophodiniales;f__Lophodiniaceae;g__Biecheleriopsis;s__Biecheleriopsis_adriatica |
| OTU2265 | 1 | 493 | JQ247714 | 88.38 | 499 | 100 | d__Eukaryota;k__Alveolata;p__norank;c__Dinophyceae;o__Gonyaulacales;f__Cladopyxidaceae;g__Peridiniella;s__Peridiniella_sp._NC-2011 |
| OTU348 | 1 | 471 | AY571374 | 85.2 | 473 | 100 | d__Eukaryota;k__Alveolata;p__norank;c__Dinophyceae;o__Lophodiniales;f__Lophodiniaceae;g__Woloszynskia;s__Woloszynskia_tenuissima |
| OTU2952 | 1 | 489 | KF245462 | 91.24 | 491 | 100 | d__Eukaryota;k__Alveolata;p__norank;c__Dinophyceae;o__Gymnodiniales;f__Gymnodiniaceae;g__Gymnodinium;s__Gymnodinium_sp. |
| OTU3320 | 1 | 488 | KP702714 | 84.62 | 65 | 53 | d__Eukaryota;k__Alveolata;p__norank;c__Dinophyceae;o__Peridiniales;f__Diplopsaliaceae;g__Lebouraia;s__Unclassified DINO |
| OTU464 | 1 | 451 | DQ162802 | 95.37 | 454 | 100 | d__Eukaryota;k__Alveolata;p__norank;c__Dinophyceae;o__Gonyaulacales;f__Gonyaulacaceae;g__Gonyaulax;s__Gonyaulax_polygramma |
| OTU1019 | 1 | 499 | FJ947042 | 83.53 | 510 | 100 | d__Eukaryota;k__Alveolata;p__norank;c__Dinophyceae;o__Gymnodiniales;f__Warnowiaceae;g__Warnowia;s__Warnowia_sp._BSL-2009a |
| OTU648 | 1 | 503 | AY571374 | 78.12 | 425 | 77 | d__Eukaryota;k__Alveolata;p__norank;c__Dinophyceae;o__Lophodiniales;f__Lophodiniaceae;g__Woloszynskia;s__Woloszynskia-like_sp. |
| OTU1092 | 1 | 510 | JN119844 | 76.97 | 547 | 100 | d__Eukaryota;k__Alveolata;p__norank;c__Dinophyceae;o__Peridiniales;f__Heterocapsaceae;g__Heterocapsa;s__Heterocapsa_sp._HCBC88 |
| OTU3702 | 1 | 495 | HQ176320 | 84 | 507 | 55 | d__Eukaryota;k__Fungi;p__environmental_samples;c__Dinophyceae;o__norank;f__norank;g__norank;s__unclassified DINO |
| OTU373 | 1 | 485 | AY916539 | 89 | 482 | 99 | d__Eukaryota;k__Alveolata;p__norank;c__Dinophyceae;o__Gymnodiniales;f__Gymnodiniaceae;g__Gymnodinium;s__Gymnodinium_microreticulatum |
| OTU3701 | 1 | 511 | HQ176320 | 87.74 | 106 | 57 | d__Eukaryota;k__Alveolata;p__norank;c__Dinophyceae;o__Peridiniales;f__Pfiesteriaceae;g__Chimonodinium;s__Chimonodinium_like |
| OTU2426 | 1 | 460 | JX262498 | 78.46 | 455 | 96 | d__Eukaryota;k__Alveolata;p__norank;c__Dinophyceae;o__Peridiniales;f__Peridiniaceae;g__Pentapharsodinium;s__Pentapharsodinium_sp. |

| | | | | | | | |
|---|---|---|---|---|---|---|---|
| OTU530 | 1 | 450 | DQ480430 | 94.29 | 35 | 22 | d__Eukaryota;k__Alveolata;p__norank;c__Dinophyceae;o__Suessiales;f__Symbiodiniaceae;g__Symbiodinium;s__unclassified_Symbiodinium |
| OTU1570 | 1 | 500 | KP790240 | 98.33 | 419 | 84 | d__Eukaryota;k__Alveolata;p__norank;c__Dinophyceae;o__Gymnodiniales;f__Warnowiaceae;g__Warnowia;s__Warnowia_sp._4_AR-2015 |
| OTU1593 | 1 | 490 | JQ247713 | 73.1 | 513 | 100 | d__Eukaryota;k__Alveolata;p__norank;c__Dinophyceae;o__Peridiniales;f__Heterocapsaceae;g__Heterocapsa;s__Heterocapsa_sp. |
| OTU1864 | 1 | 476 | HM483396 | 94.76 | 477 | 100 | d__Eukaryota;k__Alveolata;p__norank;c__Dinophyceae;o__Peridiniales;f__Thoracosphaeraceae;g__Scrippsiella;s__Scrippsiella_trochoidea |
| OTU2050 | 1 | 487 | KJ433986 | 75.51 | 490 | 100 | d__Eukaryota;k__Alveolata;p__norank;c__Dinophyceae;o__Peridiniales;f__Peridiniaceae;g__Pentapharsodinium;s__Pentapharsodinium_sp._ZL-2014 |
| OTU3804 | 1 | 497 | AF260400 | 71.6 | 507 | 100 | d__Eukaryota;k__Alveolata;p__norank;c__Dinophyceae;o__Peridiniales;f__Heterocapsaceae;g__Heterocapsa;s__Heterocapsa_rotundata |
| OTU1320 | 1 | 495 | FJ024703 | 96.38 | 497 | 100 | d__Eukaryota;k__Alveolata;p__norank;c__Dinophyceae;o__Gymnodiniales;f__Kareniaceae;g__Takayama;s__Takayama_acrotrocha |
| OTU21 | 1 | 481 | AF260389 | 92.53 | 482 | 100 | d__Eukaryota;k__Alveolata;p__norank;c__Dinophyceae;o__Gonyaulacales;f__Ceratiaceae;g__Neoceratium;s__Neoceratium_tripos |
| OTU960 | 1 | 511 | EF469234 | 89.34 | 516 | 100 | d__Eukaryota;k__Alveolata;p__norank;c__Dinophyceae;o__Gymnodiniales;f__Kareniaceae;g__Karlodinium;s__Karlodinium_antarcticum |
| OTU1090 | 1 | 500 | AY571374 | 82.02 | 534 | 100 | d__Eukaryota;k__Alveolata;p__norank;c__Dinophyceae;o__Lophodiniales;f__Lophodiniaceae;g__Woloszynskia;s__Woloszynskia_tenuissima |
| OTU2348 | 1 | 503 | FR877580 | 93.47 | 505 | 100 | d__Eukaryota;k__Alveolata;p__norank;c__Dinophyceae;o__Gonyaulacales;f__Amphidomataceae;g__Azadinium;s__Azadinium_cf._poporum_HJ-2011 |
| OTU2218 | 1 | 440 | KF031312 | 77.95 | 449 | 100 | d__Eukaryota;k__Alveolata;p__norank;c__Dinophyceae;o__Peridiniales;f__Heterocapsaceae;g__Heterocapsa;s__Heterocapsa_minima |
| OTU3823 | 1 | 454 | KJ508367 | 98.68 | 455 | 100 | d__Eukaryota;k__Alveolata;p__norank;c__Dinophyceae;o__Gymnodiniales;f__Kareniaceae;g__Karenia;s__Karenia_papilionacea |
| OTU326 | 1 | 514 | AY571369 | 93.35 | 511 | 99 | d__Eukaryota;k__Alveolata;p__norank;c__Dinophyceae;o__Gymnodiniales;f__Gymnodiniaceae;g__Gyrodinium;s__Gyrodinium_rubrum |
| OTU533 | 1 | 503 | FJ032677 | 80.71 | 508 | 99 | d__Eukaryota;k__Alveolata;p__norank;c__Dinophyceae;o__environmental_samples;f__norank;g__norank;s__uncultured_marine_DINO |
| OTU1495 | 1 | 503 | KT389895.1 | 99 | 503 | 100 | d__Eukaryota;k__Alveolata;p__norank;c__Dinophyceae;o__Pyrocystales;f__Pyrocystaceae;g__Dissodinium;s__Dissodinium_pseudolunula |
| OTU327 | 1 | 512 | AY571369 | 91.23 | 513 | 100 | d__Eukaryota;k__Alveolata;p__norank;c__Dinophyceae;o__Gymnodiniales;f__Gymnodiniaceae;g__Gyrodinium;s__Gyrodinium_rubrum |
| OTU331 | 1 | 510 | AY571370 | 99.8 | 510 | 100 | d__Eukaryota;k__Alveolata;p__norank;c__Dinophyceae;o__Gymnodiniales;f__Gymnodiniaceae;g__Gyrodinium;s__Gyrodinium_dominans |
| OTU77 | 1 | 499 | JX262498 | 86.4 | 500 | 100 | d__Eukaryota;k__Alveolata;p__norank;c__Dinophyceae;o__Peridiniales;f__Peridiniaceae;g__Pentapharsodinium;s__Pentapharsodinium_dalei |
| OTU778 | 1 | 522 | EF613356 | 77.63 | 541 | 100 | d__Eukaryota;k__Alveolata;p__norank;c__Dinophyceae;o__Gymnodiniales;f__Gymnodiniaceae;g__Katodinium;s__Katodinium_glaucum |

| | | | | | | | |
|---|---|---|---|---|---|---|---|
| OTU1417 | 1 | 526 | KT390028 | 86.09 | 532 | 100 | d__Eukaryota;k__Alveolata;p__norank;c__Dinophyceae;o__Blastodiniales; f__Blastodiniales;g__Blastodinium-like;s__Blastodinium-like_sp. |
| OTU1440 | 1 | 488 | FR720082.1 | 92.21 | 488 | 100 | d_Eukaryota;k_Alveolata;p_norank;c_Dinophyceae;o_Gymnodiniales;f_Gymnodiniaceae;g_Gyrodiniellum;s_Gyrodiniellum_shiwhaense |
| OTU2013 | 1 | 221 | EF205002 | 92.59 | 108 | 49 | d__Eukaryota;k__Alveolata;p__norank;c__Dinophyceae;o__Peridiniales;f__Glenodiniaceae;g__Glenodiniopsis;s__Glenodiniopsis_sp. |
| OTU215 | 1 | 465 | AY284951 | 98.71 | 465 | 100 | d__Eukaryota;k__Alveolata;p__norank;c__Dinophyceae;o__Gymnodiniales;f__Kareniaceae;g__Takayama;s__Takayama_helix |
| OTU3624 | 1 | 506 | AY284949 | 73 | 506 | 100 | d__Eukaryota;k__Alveolata;p__norank;c__Dinophyceae;o__Gymnodiniales;f__Kareniaceae;g__Takayama; s__Takayama_sp. |
| OTU1639 | 1 | 504 | FN557541 | 99.8 | 504 | 100 | d__Eukaryota;k__Alveolata;p__norank;c__Dinophyceae;o__unclassified_Dinophyceae;f__norank;g__Stoeckeria;s__Stoeckeria_sp._SSMS0806 |
| OTU979 | 1 | 516 | EF616462 | 84.04 | 520 | 100 | d__Eukaryota;k__Alveolata;p__norank;c__Dinophyceae;o__Gymnodiniales;f__Gymnodiniaceae;g__Cochlodinium;s__Cochlodinium_cf._geminatum |
| OTU2419 | 1 | 460 | KP702713 | 74.19 | 461 | 98 | d__Eukaryota;k__Alveolata;p__norank;c__Dinophyceae;o__Peridiniales;f__Diplopsaliaceae;g__Lebouraia;s__Lebouraia_pusilla |
| OTU962 | 1 | 488 | EF469234 | 87.58 | 499 | 100 | d__Eukaryota;k__Alveolata;p__norank;c__Dinophyceae;o__Gymnodiniales;f__Kareniaceae;g__Karlodinium;s__Karlodinium_antarcticum |
| OTU3829 | 1 | 491 | KJ508395 | 90.02 | 491 | 100 | d__Eukaryota;k__Alveolata;p__norank;c__Dinophyceae;o__unclassified_Dinophyceae;f__norank;g__Levanderina;s__Levanderina_sp. |
| OTU522 | 1 | 475 | DQ444233 | 84 | 475 | 100 | d__Eukaryota;k__Alveolata;p__norank;c__Dinophyceae;o__Peridiniales;f__Protoperidiniaceae;g__Protoperidinium;s__Protoperidinium_pellucidum |
| OTU2177 | 1 | 501 | HQ176320 | 99.6 | 502 | 100 | d__Eukaryota;k__Alveolata;p__norank;c__Dinophyceae;o__Peridiniales;f__Pfiesteriaceae;g__Chimonodinium;s__Chimonodinium_lomnickii |
| OTU986 | 1 | 511 | EF616462 | 85.28 | 496 | 97 | d__Eukaryota;k__Alveolata;p__norank;c__Dinophyceae;o__Gymnodiniales;f__Gymnodiniaceae;g__Cochlodinium;s__Cochlodinium_cf._geminatum |
| OTU2267 | 1 | 516 | JQ247714 | 93.86 | 521 | 100 | d__Eukaryota;k__Alveolata;p__norank;c__Dinophyceae;o__Gonyaulacales;f__Cladopyxidaceae;g__Peridiniella;s__Peridiniella_sp._NC-2011 |
| OTU369 | 1 | 482 | AY916538 | 98.74 | 476 | 99 | d__Eukaryota;k__Alveolata;p__norank;c__Dinophyceae;o__Gymnodiniales;f__Gymnodiniaceae;g__Gymnodinium;s__Gymnodinium_microreticulatum |
| OTU1099 | 1 | 496 | AY571374 | 77.92 | 530 | 100 | d__Eukaryota;k__Alveolata;p__norank;c__Dinophyceae;o__Lophodiniales;f__Lophodiniaceae;g__Woloszynskia;s__Woloszynskia_tenuissima |
| OTU3730 | 1 | 475 | HG792066 | 91.01 | 89 | 71 | d__Eukaryota;k__Alveolata;p__norank;c__Dinophyceae;o__unclassified_Dinophyceae;f__norank;g__norank;s__Dinophyceae_sp._1_HJH-2013 |
| OTU496 | 1 | 485 | FJ024705 | 81.39 | 489 | 100 | d__Eukaryota;k__Alveolata;p__norank;c__Dinophyceae;o__Suessiales;f__Biecheleriaceae;g__Biecheleria;s__Biecheleria_cincta |
| OTU186 | 1 | 519 | AY245689 | 94.8 | 519 | 100 | d__Eukaryota;k__Alveolata;p__norank;c__Dinophyceae;o__Peridiniales;f__Pfiesteriaceae;g__unclassified_Pfiesteriaceae;s__Pfiesteria-like_DINO_Lucy |
| OTU2750 | 1 | 490 | JX262498 | 97.13 | 488 | 99 | d__Eukaryota;k__Alveolata;p__norank;c__Dinophyceae;o__Peridiniales;f__Peridiniaceae;g__Pentapharsodinium;s__Pentapharsodinium_dalei |

| | | | | | | | |
|---|---|---|---|---|---|---|---|
| OTU2961 | 1 | 503 | JQ247713 | 83.4 | 512 | 99 | d__Eukaryota;k__Alveolata;p__norank;c__Dinophyceae;o__Peridiniales;f__Heterocapsaceae;g__Heterocapsa;s__Heterocapsa_niei |
| OTU3812 | 1 | 497 | EF469234 | 78.52 | 512 | 100 | d__Eukaryota;k__Alveolata;p__norank;c__Dinophyceae;o__Gymnodiniales;f__Kareniaceae;g__Karenia;s__Unclassified Kareniaceae__sp. |
| OTU1676 | 1 | 525 | HE611580 | 94.32 | 88 | 55 | d__Eukaryota;k__Alveolata;p__norank;c__Dinophyceae;o__Gymnodiniales;f__Gymnodiniaceae;g__Gyrodinium;s__Gyrodinium_sp._HJ-2011 |
| OTU1014 | 1 | 435 | EF616464 | 98.68 | 76 | 17 | d__Eukaryota;k__Alveolata;p__norank;c__Dinophyceae;o__norank;f__norank;g__norank;s__Unclassified DINO |
| OTU3589 | 1 | 416 | KJ450988 | 91.15 | 418 | 100 | d__Eukaryota;k__Alveolata;p__norank;c__Dinophyceae;o__Peridiniales;f__Peridiniaceae;g__Peridinium;s__Peridinium_euryceps |
| OTU3744 | 1 | 531 | LM049720 | 85.71 | 63 | 27 | d__Eukaryota;k__Alveolata;p__norank;c__Dinophyceae;o__norank;f__norank;g__norank;s__unclassified DINO |
| OTU3816 | 1 | 485 | HQ176320.1 | 85 | 240 | 50 | d__Eukaryota;k__Alveolata;p__norank;c__Dinophyceae;o__no rank;f__no rank;g__no rank;s__Unclassified DINO |
| OTU1454 | 1 | 562 | FJ160592 | 90.12 | 81 | 31 | d__Eukaryota;k__Alveolata;p__norank;c__Dinophyceae;o__norank;f__norank;g__norank;s__Unclassified DINO |
| OTU2345 | 1 | 477 | JN934999 | 90.79 | 76 | 27 | d__Eukaryota;k__Alveolata;p__norank;c__Dinophyceae;o__Syndiniales;f__Eudubosquellidae;g__norank;s__Unclassified Eudubosquellidae species |
| OTU2675 | 1 | 480 | AB716929 | 98.8 | 125 | 61 | d__Eukaryota;k__Alveolata;p__norank;c__Dinophyceae;o__Peridiniales;f__Protoperidiniaceae;g__Protoperidinium;s__Protoperidinium-like_sp. |
| OTU3300 | 1 | 496 | KF878935 | 96.57 | 496 | 100 | d__Eukaryota;k__Alveolata;p__norank;c__Dinophyceae;o__Gymnodiniales;f__Gymnodiniaceae;g__Gymnodinium;s__Gymnodinium_instriatum |
| OTU3449 | 1 | 499 | KJ879232 | 100 | 499 | 100 | d__Eukaryota;k__Alveolata;p__norank;c__Dinophyceae;o__Gonyaulacales;f__Gonyaulacaceae;g__Alexandrium;s__Alexandrium_catenella |
| OTU3639 | 1 | 510 | JX262498 | 85 | 510 | 100 | d__Eukaryota;k__Alveolata;p__norank;c__Dinophyceae;o__Peridiniales; f__Peridiniaceae; g__Pentapharsodinium; s__Pentapharsodinium_sp. |
| OTU1850 | 1 | 486 | HM483396 | 91.56 | 486 | 100 | d__Eukaryota;k__Alveolata;p__norank;c__Dinophyceae;o__Peridiniales;f__Thoracosphaeraceae;g__Scrippsiella;s__Scrippsiella_trochoidea |
| OTU2724 | 1 | 457 | JX262498 | 86.68 | 458 | 100 | d__Eukaryota;k__Alveolata;p__norank;c__Dinophyceae;o__Peridiniales;f__Peridiniaceae;g__Pentapharsodinium;s__Pentapharsodinium_dalei |
| OTU1345 | 1 | 477 | FJ024705 | 92.75 | 483 | 100 | d__Eukaryota;k__Alveolata;p__norank;c__Dinophyceae;o__Suessiales;f__Biecheleriaceae;g__Biecheleria;s__Biecheleria_cincta |
| OTU772 | 1 | 504 | EF613353 | 94.64 | 504 | 100 | d__Eukaryota;k__Alveolata;p__norank;c__Dinophyceae;o__unclassified_Dinophyceae;f__norank;g__Levanderina;s__Levanderina_fissa |
| OTU102 | 1 | 496 | KJ508360 | 95.77 | 497 | 100 | d__Eukaryota;k__Alveolata;p__norank;c__Dinophyceae;o__Gymnodiniales;f__Kareniaceae;g__Karenia;s__Karenia_cristata |
| OTU1110 | 1 | 501 | AY571374 | 80.9 | 513 | 100 | d__Eukaryota;k__Alveolata;p__norank;c__Dinophyceae;o__Lophodiniales;f__Lophodiniaceae;g__Woloszynskia;s__Woloszynskia_tenuissima |
| OTU250 | 1 | 462 | AY154962 | 90.52 | 464 | 100 | d__Eukaryota;k__Alveolata;p__norank;c__Dinophyceae;o__Gonyaulacales;f__Gonyaulacaceae;g__Gonyaulax;s__Gonyaulax_baltica |

| | | | | | | | |
|---|---|---|---|---|---|---|---|
| OTU365 | 1 | 504 | AY863008 | 99.4 | 504 | 100 | d__Eukaryota;k__Alveolata;p__norank;c__Dinophyceae;o__Prorocentrales;f__Prorocentraceae;g__Prorocentrum;s__Prorocentrum_micans |
| OTU3797 | 1 | 528 | JX262498 | 76 | 109 | 100 | d__Eukaryota;k__Alveolata;p__norank;c__Dinophyceae;o__Peridiniales;f__Peridiniaceae;g__Pentapharsodinium;s__Pentapharsodinium_sp. |
| OTU950 | 1 | 486 | EF613366 | 92.18 | 486 | 100 | d__Eukaryota;k__Alveolata;p__norank;c__Dinophyceae;o__Peridiniales;f__Thoracosphaeraceae;g__Scrippsiella;s__Scrippsiella_trochoidea |
| OTU1071 | 1 | 513 | JN119844 | 76.35 | 554 | 100 | d__Eukaryota;k__Alveolata;p__norank;c__Dinophyceae;o__Peridiniales;f__Heterocapsaceae;g__Heterocapsa;s__Heterocapsa_sp._HCBC88 |
| OTU1415 | 1 | 531 | KT390028 | 90.4 | 531 | 100 | d__Eukaryota;k__Alveolata;p__norank;c__Dinophyceae;o__Blastodiniales; f__Blastodiniales;g__Blastodinium-like;s__Blastodinium-like_sp. |
| OTU2207 | 1 | 489 | JN558110 | 77.92 | 548 | 100 | d__Eukaryota;k__Alveolata;p__norank;c__Dinophyceae;o__Suessiales;f__Suessiaceae;g__Polarella;s__Polarella_glacialis |
| OTU3527 | 1 | 483 | KF646481 | 99.79 | 484 | 100 | d__Eukaryota;k__Alveolata;p__norank;c__Dinophyceae;o__Gonyaulacales;f__Gonyaulacaceae;g__Alexandrium;s__Alexandrium_tamarense_species_complex_group_I |
| OTU3814 | 1 | 499 | KJ508393 | 99 | 499 | 100 | d__Eukaryota;k__Alveolata;p__norank;c__Dinophyceae;o__Gymnodiniales;f__Gymnodiniaceae;g__Gymnodinium;s__Gymnodinium_impudicum |
| OTU1402 | 1 | 517 | KT390028 | 91.68 | 517 | 100 | d__Eukaryota;k__Alveolata;p__norank;c__Dinophyceae;o__Blastodiniales; f__Blastodiniales;g__Blastodinium-like;s__Blastodinium-like_sp. |
| OTU615 | 1 | 490 | EF052683 | 91.26 | 492 | 100 | d__Eukaryota;k__Alveolata;p__norank;c__Dinophyceae;o__Peridiniales;f__unclassified_Peridiniales;g__Baldinia;s__Baldinia_anauniensis |
| OTU3336 | 1 | 537 | KF751924 | 85.92 | 547 | 100 | d__Eukaryota;k__Alveolata;p__norank;c__Dinophyceae;o__Peridiniales;f__Thoracosphaeraceae;g__Scrippsiella;s__Scrippsiella_aff._acuminata |
| OTU2038 | 1 | 505 | AF260399 | 91.18 | 510 | 100 | d__Eukaryota;k__Alveolata;p__norank;c__Dinophyceae;o__Peridiniales;f__Heterocapsaceae;g__Heterocapsa;s__Heterocapsa_sp. |
| OTU2614 | 1 | 281 | JN982400 | 98.12 | 266 | 95 | d__Eukaryota;k__Alveolata;p__norank;c__Dinophyceae;o__Peridiniales;f__Thoracosphaeraceae;g__Scrippsiella;s__Scrippsiella_aff._acuminata |
| OTU2655 | 1 | 506 | AB716928 | 96.05 | 506 | 100 | d__Eukaryota;k__Alveolata;p__norank;c__Dinophyceae;o__Peridiniales;f__Protoperidiniaceae;g__Protoperidinium;s__Protoperidinium_monovelum |
| OTU2679 | 1 | 440 | AB716929 | 97.6 | 125 | 64 | d__Eukaryota;k__Alveolata;p__norank;c__Dinophyceae;o__Peridiniales;f__Protoperidiniaceae;g__Protoperidinium;s__Protoperidinium-like_sp. |
| OTU723 | 1 | 517 | AB288382 | 99.81 | 517 | 100 | d__Eukaryota;k__Alveolata;p__norank;c__Dinophyceae;o__Gymnodiniales;f__Gymnodiniaceae;g__Cochlodinium;s__Cochlodinium_fulvescens |
| OTU1138 | 1 | 506 | JN119844 | 82.84 | 507 | 100 | d__Eukaryota;k__Alveolata;p__norank;c__Dinophyceae;o__Peridiniales;f__Heterocapsaceae;g__Heterocapsa;s__Heterocapsa_sp._HCBC88 |
| OTU2191 | 1 | 478 | AF200673 | 91.96 | 112 | 42 | d__Eukaryota;k__Alveolata;p__norank;c__Dinophyceae;o__Gymnodiniales;f__Gymnodiniaceae;g__norank;s__Unclassified Gymnodinium-like_species |
| OTU356 | 1 | 507 | AY590476 | 99.6 | 501 | 99 | d__Eukaryota;k__Alveolata;p__norank;c__Dinophyceae;o__Peridiniales;f__Pfiesteriaceae;g__Cryptoperidiniopsis;s__Cryptoperidiniopsis_brodyi |
| OTU3731 | 1 | 469 | HG792066 | 94.38 | 89 | 74 | d__Eukaryota;k__Alveolata;p__norank;c__Dinophyceae;o__unclassified_Dinophyceae;f__norank;g__norank;s__Dinophyceae_sp._1_HJH-2013 |

| | | | | | | | |
|---|---|---|---|---|---|---|---|
| OTU497 | 1 | 467 | JN558110 | 76.54 | 486 | 100 | d__Eukaryota;k__Alveolata;p__norank;c__Dinophyceae;o__Suessiales;f__Suessiaceae;g__Polarella;s__Polarella_glacialis |
| OTU507 | 1 | 474 | DQ289020 | 90.62 | 64 | 36 | d__Eukaryota;k__Alveolata;p__norank;c__Dinophyceae;o__Gymnodiniales;f__Unclassified;g__Unclassified;s__Unclassified_Gymnodiniales |
| OTU754 | 1 | 405 | KJ508376.1 | 77 | 74 | 72 | d__Eukaryota;k__Alveolata;p__norank;c__Dinophyceae;o__Gymnodiniales;f__Kareniaceae-like;g__Unclassified;s__Unclassified DINO |
| OTU1915 | 1 | 280 | HM483396 | 95.68 | 278 | 99 | d__Eukaryota;k__Alveolata;p__norank;c__Dinophyceae;o__Peridiniales;f__Thoracosphaeraceae;g__Scrippsiella;s__Scrippsiella_trochoidea |
| OTU3706 | 1 | 519 | HE611580 | 96.51 | 86 | 54 | d__Eukaryota;k__Alveolata;p__norank;c__Dinophyceae;o__Gymnodiniales;f__Gymnodiniaceae;g__Gyrodinium;s__Gyrodinium_sp._HJ-2011 |
| OTU76 | 1 | 499 | AF260392 | 87.23 | 501 | 100 | d__Eukaryota;k__Alveolata;p__norank;c__Dinophyceae;o__Peridiniales;f__Thoracosphaeraceae;g__Scrippsiella;s__Scrippsiella_sp. |
| OTU1017 | 1 | 346 | EF616464 | 98.68 | 76 | 22 | d__Eukaryota;k__Alveolata;p__norank;c__Dinophyceae;o__norank;f__norank;g__norank;s__Unclassified DINO |
| OTU1097 | 1 | 487 | EU126801 | 76.76 | 525 | 100 | d__Eukaryota;k__Alveolata;p__norank;c__Dinophyceae;o__Suessiales;f__Borghiellaceae;g__Borghiella;s__Borghiella_dodgei |
| OTU1103 | 1 | 485 | AY571374 | 80.67 | 507 | 100 | d__Eukaryota;k__Alveolata;p__norank;c__Dinophyceae;o__Lophodiniales;f__Lophodiniaceae;g__Woloszynskia;s__Woloszynskia_tenuissima |
| OTU2224 | 1 | 495 | JN119844 | 80.24 | 506 | 100 | d__Eukaryota;k__Alveolata;p__norank;c__Dinophyceae;o__Peridiniales;f__Heterocapsaceae;g__Heterocapsa;s__Heterocapsa_sp._HCBC88 |
| OTU2495 | 1 | 501 | KP790208 | 89.47 | 76 | 15 | d__Eukaryota;k__Alveolata;p__norank;c__Dinophyceae;o__norank;f__norank;g__norank;s__Unclassifid DINO |
| OTU975 | 1 | 505 | AB295051 | 93.89 | 507 | 100 | d__Eukaryota;k__Alveolata;p__norank;c__Dinophyceae;o__Gymnodiniales;f__Gymnodiniaceae;g__Cochlodinium;s__Cochlodinium_fulvescens |
| OTU1413 | 1 | 529 | KT390028 | 88.72 | 532 | 100 | d__Eukaryota;k__Alveolata;p__norank;c__Dinophyceae;o__Blastodiniales; f__Blastodiniales;g__Blastodinium-like;s__Blastodinium-like_sp. |
| OTU3705 | 1 | 516 | FN557541 | 85.38 | 89 | 54 | d__Eukaryota;k__Alveolata;p__norank;c__Dinophyceae;o__unclassified_Dinophyceae;f__norank;g__Stoeckeria;s__Stoeckeria_sp._SSMS0806 |
| OTU3795 | 1 | 510 | KJ508377 | 79 | 520 | 99 | d__Eukaryota;k__Alveolata;p__norank;c__Dinophyceae;o__Gymnodiniales;f__Kareniaceae;g__Karlodinium;s__Karlodinium_sp. |
| OTU601 | 1 | 477 | KM042422 | 76.3 | 481 | 100 | d__Eukaryota;k__Alveolata;p__norank;c__Dinophyceae;o__Peridiniales;f__Protoperidiniaceae;g__Protoperidinium;s__Protoperidinium_abei |
| OTU1007 | 1 | 331 | EF616462 | 86.41 | 309 | 92 | d__Eukaryota;k__Alveolata;p__norank;c__Dinophyceae;o__Gymnodiniales;f__Gymnodiniaceae;g__Cochlodinium;s__Cochlodinium_cf._geminatum |
| OTU1622 | 1 | 447 | KJ189480 | 83.5 | 103 | 23 | d__Eukaryota;k__Alveolata;p__norank;c__Dinophyceae;o__Peridiniales;f__Thoracosphaeraceae;g__Scrippsiella-like;s__Scrippsiella-like_sp. |
| OTU2261 | 1 | 492 | AF260400 | 81.25 | 496 | 100 | d__Eukaryota;k__Alveolata;p__norank;c__Dinophyceae;o__Peridiniales;f__Heterocapsaceae;g__Heterocapsa;s__Heterocapsa_rotundata |
| OTU495 | 1 | 492 | LC068838.1 | 87 | 492 | 100 | d__Eukaryota;k__Alveolata;p__norank;c__Dinophyceae;o__Suessiales;f__Suessiaceae;g__Unclassified;s__Unclassified Suessiaceae |

| | | | | | | | |
|---|---|---|---|---|---|---|---|
| OTU768 | 1 | 482 | EF613349 | 84.42 | 77 | 53 | d__Eukaryota;k__Alveolata;p__norank;c__Dinophyceae;o__Gonyaulacales;f__Gonyaulacaceae;g__Unclassified;s__Unclassified DINO |
| OTU1182 | 1 | 549 | KT804914.1 | 90 | 95 | 18 | d__Eukaryota;k__Alveolata;p__norank;c__Dinophyceae;o__norank;f__norank;g__norank;s__Unclassified DINO |
| OTU1577 | 1 | 418 | KP790242 | 98.8 | 418 | 100 | d__Eukaryota;k__Alveolata;p__norank;c__Dinophyceae;o__Gymnodiniales;f__Warnowiaceae;g__Warnowia;s__Warnowia_sp._5_AR-2015 |
| OTU1594 | 1 | 463 | FN357291 | 72.73 | 484 | 99 | d__Eukaryota;k__Alveolata;p__norank;c__Dinophyceae;o__Gymnodiniales;f__Kareniaceae;g__Karlodinium;s__Karlodinium_sp._KAMS0708 |
| OTU2497 | 1 | 515 | AB702991 | 99.42 | 515 | 100 | d__Eukaryota;k__Alveolata;p__norank;c__Dinophyceae;o__Peridiniales;f__Amphidiniopsidaceae;g__Archaeperidinium;s__Archaeperidinium_saanichi |
| OTU3447 | 1 | 275 | LM992906 | 81.58 | 152 | 99 | d__Eukaryota;k__Alveolata;p__norank;c__Dinophyceae;o__Lophodiniales;f__Lophodiniaceae;g__Biecheleriopsis;s__Biecheleriopsis_sp. |
| OTU3593 | 1 | 516 | AB716928 | 89.36 | 517 | 100 | d__Eukaryota;k__Alveolata;p__norank;c__Dinophyceae;o__Peridiniales;f__Protoperidiniaceae;g__Protoperidinium;s__Protoperidinium_monovelum |
| OTU532 | 1 | 480 | AM408889 | 82.27 | 485 | 100 | d__Eukaryota;k__Alveolata;p__norank;c__Dinophyceae;o__Gymnodiniales;f__Gymnodiniaceae;g__Paragymnodinium;s__Paragymnodinium_shiwhaense |
| OTU1511 | 1 | 489 | EU707473 | 92.06 | 491 | 100 | d__Eukaryota;k__Alveolata;p__norank;c__Dinophyceae;o__Gonyaulacales;f__Gonyaulacaceae;g__Alexandrium;s__Alexandrium_minutum |
| OTU1640 | 1 | 485 | LK934662 | 92.26 | 478 | 98 | d__Eukaryota;k__Alveolata;p__norank;c__Dinophyceae;o__Peridiniales;f__Pfiesteriaceae;g__Aduncodinium;s__Aduncodinium_glandula |
| OTU2003 | 1 | 499 | AM408889 | 81.37 | 510 | 100 | d__Eukaryota;k__Alveolata;p__norank;c__Dinophyceae;o__Gymnodiniales;f__Gymnodiniaceae;g__Paragymnodinium;s__Paragymnodinium_sp. |
| OTU2271 | 1 | 513 | JQ247714 | 92.65 | 517 | 100 | d__Eukaryota;k__Alveolata;p__norank;c__Dinophyceae;o__Gonyaulacales;f__Cladopyxidaceae;g__Peridiniella;s__Peridiniella_sp._NC-2011 |
| OTU3434 | 1 | 484 | LM992906 | 92.52 | 481 | 99 | d__Eukaryota;k__Alveolata;p__norank;c__Dinophyceae;o__Lophodiniales;f__Lophodiniaceae;g__Biecheleriopsis;s__Biecheleriopsis_adriatica |
| OTU506 | 1 | 463 | JQ439944 | 79.28 | 111 | 46 | d__Eukaryota;k__Alveolata;p__norank;c__Dinophyceae;o__Gymnodiniales;f__Unclassified;g__Unclassified;s__Unclassified_Gymnodiniales |
| OTU1013 | 1 | 489 | AB858353 | 92.86 | 490 | 100 | d__Eukaryota;k__Alveolata;p__norank;c__Dinophyceae;o__Suessiales;f__Biecheleriaceae;g__Biecheleria;s__Biecheleria_brevisulcata |
| OTU1906 | 1 | 395 | HM483396 | 96.23 | 398 | 100 | d__Eukaryota;k__Alveolata;p__norank;c__Dinophyceae;o__Peridiniales;f__Thoracosphaeraceae;g__Scrippsiella;s__Scrippsiella_trochoidea |
| OTU2039 | 1 | 496 | HQ902267 | 97.98 | 496 | 100 | d__Eukaryota;k__Alveolata;p__norank;c__Dinophyceae;o__Peridiniales;f__Heterocapsaceae;g__Heterocapsa;s__Heterocapsa_triquetra |
| OTU2408 | 1 | 507 | AY331681 | 81.57 | 510 | 100 | d__Eukaryota;k__Alveolata;p__norank;c__Dinophyceae;o__Gymnodiniales;f__Gymnodiniaceae;g__Lepidodinium;s__Lepidodinium_chlorophorum |
| OTU2425 | 1 | 409 | HM596557 | 90 | 154 | 50 | d__Eukaryota;k__Alveolata;p__norank;c__Dinophyceae;o__Gymnodiniales;f__Gymnodiniaceae;g__norank;s__Unclassified gymnodiniaceae species |
| OTU2588 | 1 | 290 | JN982392 | 94.96 | 278 | 96 | d__Eukaryota;k__Alveolata;p__norank;c__Dinophyceae;o__Peridiniales;f__Thoracosphaeraceae;g__Scrippsiella;s__Scrippsiella_sweeneyae |

| | | | | | | | |
|---|---|---|---|---|---|---|---|
| OTU2652 | 1 | 497 | DQ195367 | 86.21 | 87 | 63 | d__Eukaryota;k__Alveolata;p__norank;c__Dinophyceae;o__Suessiales;f__Suessiaceae;g__Pelagodinium;s__Pelagodinium-like species |
| OTU2730 | 1 | 418 | JX262498 | 95.92 | 147 | 98 | d__Eukaryota;k__Alveolata;p__norank;c__Dinophyceae;o__Peridiniales;f__Peridiniaceae;g__Pentapharsodinium;s__Pentapharsodinium_dalei |
| OTU3430 | 1 | 494 | LM992906 | 94.13 | 494 | 100 | d__Eukaryota;k__Alveolata;p__norank;c__Dinophyceae;o__Lophodiniales;f__Lophodiniaceae;g__Biecheleriopsis;s__Biecheleriopsis_adriatica |
| OTU3612 | 1 | 499 | KJ481814 | 90.2 | 500 | 100 | d__Eukaryota;k__Alveolata;p__norank;c__Dinophyceae;o__Gonyaulacales;f__Amphidomataceae;g__Azadinium;s__Azadinium_trinitatum |
| OTU1161 | 1 | 313 | HM483396 | 93.29 | 313 | 100 | d__Eukaryota;k__Alveolata;p__norank;c__Dinophyceae;o__Peridiniales;f__Thoracosphaeraceae;g__Scrippsiella;s__Scrippsiella_trochoidea |
| OTU277 | 1 | 467 | KF998563 | 88.89 | 90 | 26 | d__Eukaryota;k__Alveolata;p__norank;c__Dinophyceae;o__unclassified;f__unclassified;g__unclassified;s__Unclassified DINO |
| OTU329 | 1 | 394 | AY571369 | 97.22 | 395 | 100 | d__Eukaryota;k__Alveolata;p__norank;c__Dinophyceae;o__Gymnodiniales;f__Gymnodiniaceae;g__Gyrodinium;s__Gyrodinium_rubrum |
| OTU64 | 1 | 498 | AF260393 | 95.98 | 498 | 100 | d__Eukaryota;k__Alveolata;p__norank;c__Dinophyceae;o__Peridiniales;f__Thoracosphaeraceae;g__Scrippsiella;s__Scrippsiella_trochoidea |
| OTU671 | 1 | 472 | KT389946.1 | 84 | 478 | 100 | d__Eukaryota;k__Alveolata;p__norank;c__Dinophyceae;o__Peridiniales;f__Thoracosphaeraceae;g__Scrippsiella;s__Scrippsiella_sp. |
| OTU2037 | 1 | 379 | HM483396 | 93.8 | 371 | 98 | d__Eukaryota;k__Alveolata;p__norank;c__Dinophyceae;o__Peridiniales;f__Thoracosphaeraceae;g__Scrippsiella;s__Scrippsiella_trochoidea |
| OTU2093 | 1 | 497 | HQ670228 | 94.37 | 497 | 100 | d__Eukaryota;k__Alveolata;p__norank;c__Dinophyceae;o__Peridiniales;f__Thoracosphaeraceae;g__Scrippsiella;s__Scrippsiella_trochoidea |
| OTU2266 | 1 | 515 | HQ902267 | 81.98 | 516 | 100 | d__Eukaryota;k__Alveolata;p__norank;c__Dinophyceae;o__Peridiniales;f__Heterocapsaceae;g__Heterocapsa;s__Heterocapsa_triquetra |
| OTU2492 | 1 | 512 | JQ179865 | 80 | 78 | 61 | d__Eukaryota;k__Alveolata;p__norank;c__Dinophyceae;o__Gymnodiniales;f__Unclassified Gymnodiniaceae;g__norank;s__Unclassified Gymnodiniaceae |
| OTU3463 | 1 | 489 | AB860180 | 87.7 | 496 | 100 | d__Eukaryota;k__Alveolata;p__norank;c__Dinophyceae;o__Gymnodiniales;f__Gymnodiniaceae;g__Gymnodinium;s__unclassified_Gymnodinium |
| OTU3710 | 1 | 507 | EU490149 | 72.34 | 499 | 96 | d__Eukaryota;k__Alveolata;p__norank;;c__Dinophyceae;o__norank;f__norank;g__norank;s__unclassified DINO |
| OTU2025 | 1 | 504 | AF260399 | 80.35 | 514 | 100 | d__Eukaryota;k__Alveolata;p__norank;c__Dinophyceae;o__Peridiniales;f__Heterocapsaceae;g__Heterocapsa;s__Heterocapsa_sp. |
| OTU2277 | 1 | 446 | HQ890883 | 98 | 47 | 9 | d__Eukaryota;k__Alveolata;p__norank;c__Dinophyceae;o__norank;f__norank;g__norank;s__Unclassified DINO |
| OTU2653 | 1 | 523 | AB716928 | 90.06 | 523 | 100 | d__Eukaryota;k__Alveolata;p__norank;c__Dinophyceae;o__Peridiniales;f__Protoperidiniaceae;g__Protoperidinium;s__Protoperidinium_monovelum |
| OTU3370 | 1 | 510 | KF751926 | 97.98 | 496 | 97 | d__Eukaryota;k__Alveolata;p__norank;c__Dinophyceae;o__Peridiniales;f__Thoracosphaeraceae;g__Scrippsiella;s__Scrippsiella_sp. |
| OTU3508 | 1 | 470 | AB860180 | 84.07 | 477 | 100 | d__Eukaryota;k__Alveolata;p__norank;c__Dinophyceae;o__Gymnodiniales;f__Gymnodiniaceae;g__Gymnodinium;s__unclassified_Gymnodinium |

| | | | | | | | |
|---|---|---|---|---|---|---|---|
| OTU56 | 1 | 499 | AF260393 | 97.18 | 497 | 99 | d__Eukaryota;k__Alveolata;p__norank;c__Dinophyceae;o__Peridiniales;f__Thoracosphaeraceae;g__Scrippsiella;s__Scrippsiella_trochoidea |
| OTU573 | 1 | 490 | AM408889 | 81.48 | 486 | 99 | d__Eukaryota;k__Alveolata;p__norank;c__Dinophyceae;o__Gymnodiniales;f__Gymnodiniaceae;g__Paragymnodinium;s__Paragymnodinium_shiwhaense |
| OTU999 | 1 | 385 | EF616462 | 86.75 | 317 | 82 | d__Eukaryota;k__Alveolata;p__norank;c__Dinophyceae;o__Gymnodiniales;f__Gymnodiniaceae;g__Cochlodinium;s__Cochlodinium_cf._geminatum |
| OTU1113 | 1 | 528 | EU126801.1 | 94 | 91 | 49 | d__Eukaryota;k__Alveolata;p__norank;c__Dinophyceae;o__norank;f__norank;g__norank;s__Unclassified DINO |
| OTU1162 | 1 | 501 | KF751924 | 92.48 | 532 | 100 | d__Eukaryota;k__Alveolata;p__norank;c__Dinophyceae;o__Peridiniales;f__Thoracosphaeraceae;g__Scrippsiella;s__Scrippsiella_aff._acuminata |
| OTU1314 | 1 | 497 | FR720082 | 81.71 | 514 | 100 | d__Eukaryota;k__Alveolata;p__norank;c__Dinophyceae;o__Gymnodiniales;f__Gymnodiniaceae;g__Gyrodiniellum;s__Gyrodiniellum_shiwhaense |
| OTU1404 | 1 | 528 | KT390028 | 90.53 | 528 | 100 | d__Eukaryota;k__Alveolata;p__norank;c__Dinophyceae;o__Blastodiniales; f__Blastodiniales;g__Blastodinium-like;s__Blastodinium-like_sp. |
| OTU1572 | 1 | 492 | FJ947041 | 93.71 | 493 | 100 | d__Eukaryota;k__Alveolata;p__norank;c__Dinophyceae;o__Gymnodiniales;f__Warnowiaceae;g__Nematodinium;s__Nematodinium_sp._BSL-2009a |
| OTU1643 | 1 | 498 | FN557541 | 96.79 | 498 | 100 | d__Eukaryota;k__Alveolata;p__norank;c__Dinophyceae;o__unclassified_Dinophyceae;f__norank;g__Stoeckeria;s__Stoeckeria_sp._SSMS0806 |
| OTU1790 | 1 | 511 | HM483396 | 86.19 | 507 | 98 | d__Eukaryota;k__Alveolata;p__norank;c__Dinophyceae;o__Peridiniales;f__Thoracosphaeraceae;g__Scrippsiella;s__Scrippsiella_trochoidea |
| OTU1824 | 1 | 492 | HM483396 | 88.96 | 498 | 100 | d__Eukaryota;k__Alveolata;p__norank;c__Dinophyceae;o__Peridiniales;f__Thoracosphaeraceae;g__Scrippsiella;s__Scrippsiella_trochoidea |
| OTU1832 | 1 | 369 | HM483396 | 92.14 | 369 | 100 | d__Eukaryota;k__Alveolata;p__norank;c__Dinophyceae;o__Peridiniales;f__Thoracosphaeraceae;g__Scrippsiella;s__Scrippsiella_trochoidea |
| OTU2002 | 1 | 493 | FR720082 | 82.29 | 497 | 100 | d__Eukaryota;k__Alveolata;p__norank;c__Dinophyceae;o__Gymnodiniales;f__Gymnodiniaceae;g__Gyrodiniellum;s__Gyrodiniellum_sp. |
| OTU2033 | 1 | 288 | HQ845329 | 96.89 | 289 | 100 | d__Eukaryota;k__Alveolata;p__norank;c__Dinophyceae;o__Peridiniales;f__Peridiniaceae;g__Pentapharsodinium;s__Pentapharsodinium_tyrrhenicum |
| OTU2198 | 1 | 503 | JN558107 | 73.87 | 509 | 100 | d__Eukaryota;k__Alveolata;p__norank;c__Dinophyceae;o__Suessiales;f__Suessiaceae;g__Pelagodinium;s__Pelagodinium_sp. |
| OTU2206 | 1 | 443 | JN558107 | 94 | 147 | 46 | d__Eukaryota;k__Alveolata;p__norank;c__Dinophyceae;o__norank;f__norank;g__norank;s__Unclassified DINO |
| OTU2263 | 1 | 496 | JQ247713 | 81.6 | 500 | 100 | d__Eukaryota;k__Alveolata;p__norank;c__Dinophyceae;o__Peridiniales;f__Heterocapsaceae;g__Heterocapsa;s__Heterocapsa_niei |
| OTU2324 | 1 | 555 | JN934988 | 98.2 | 556 | 100 | d__Eukaryota;k__Alveolata;p__norank;c__Dinophyceae;o__Syndiniales;f__Euduboscquellidae;g__Euduboscquella;s__Euduboscquella_cachoni |
| OTU2561 | 1 | 502 | EU165273 | 84.34 | 198 | 39 | d__Eukaryota;k__Alveolata;p__norank;c__Dinophyceae;o__Peridiniales;f__Heterocapsaceae;g__unclassified_Heterocapsaceae;s__Unclassified Heterocapsaceae_sp. |
| OTU2586 | 1 | 437 | HM483396 | 87.53 | 465 | 100 | d__Eukaryota;k__Alveolata;p__norank;c__Dinophyceae;o__Peridiniales;f__Thoracosphaeraceae;g__Scrippsiella;s__Scrippsiella_trochoidea |

| | | | | | | | |
|---|---|---|---|---|---|---|---|
| OTU3298 | 1 | 512 | KF878935 | 90.66 | 514 | 100 | d__Eukaryota;k__Alveolata;p__norank;c__Dinophyceae;o__Gymnodiniales;f__Gymnodiniaceae;g__Gymnodinium;s__Gymnodinium_instriatum |
| OTU3309 | 1 | 460 | KF878935 | 96.52 | 460 | 100 | d__Eukaryota;k__Alveolata;p__norank;c__Dinophyceae;o__Gymnodiniales;f__Gymnodiniaceae;g__Gymnodinium;s__Gymnodinium_instriatum |
| OTU3316 | 1 | 509 | KF568378 | 86.6 | 97 | 19 | d__Eukaryota;k__Alveolata;p__norank;c__Dinophyceae;s__Unclaassified DINO |
| OTU3333 | 1 | 515 | KF751924 | 90.72 | 517 | 100 | d__Eukaryota;k__Alveolata;p__norank;c__Dinophyceae;o__Peridiniales;f__Thoracosphaeraceae;g__Scrippsiella;s__Scrippsiella_aff._acuminata |
| OTU3390 | 1 | 459 | KF751926 | 93.08 | 491 | 100 | d__Eukaryota;k__Alveolata;p__norank;c__Dinophyceae;o__Peridiniales;f__Thoracosphaeraceae;g__Scrippsiella;s__Scrippsiella_sp. |
| OTU3448 | 1 | 441 | KF646451 | 98.19 | 441 | 100 | d__Eukaryota;k__Alveolata;p__norank;c__Dinophyceae;o__Gonyaulacales;f__Gonyaulacaceae;g__Alexandrium;s__Alexandrium_tamarense_species_complex_group_IV |
| OTU3736 | 1 | 287 | LC068838.1 | 83.19 | 282 | 98 | d__Eukaryota;k__Alveolata;p__norank;c__Dinophyceae;o__Suessiales;f__Biecheleriaceae;g__norank;s__Unclassified Suessiales |
| OTU3820 | 1 | 468 | KP790228 | 88.76 | 89 | 19 | d__Eukaryota;k__Alveolata;p__norank;c__Dinophyceae;o__Gymnodiniales;f__Polykrikaceae;g__Polykrikos;s__Polykrikos-like_sp. |
| OTU459 | 1 | 485 | AY154965 | 76.63 | 475 | 95 | d__Eukaryota;k__Alveolata;p__norank;c__Dinophyceae;o__Gonyaulacales;f__Gonyaulacaceae;g__Gonyaulax;s__Gonyaulax_sp. |
| OTU781 | 1 | 524 | KP790221 | 97.53 | 160 | 33 | d__Eukaryota;k__Alveolata;p__norank;c__Dinophyceae;o__Gymnodiniales;f__Gymnodiniaceae;g__Katodinium-like;s__Katodinium-like_sp. |
| OTU1821 | 1 | 493 | HM483396 | 94.64 | 504 | 100 | d__Eukaryota;k__Alveolata;p__norank;c__Dinophyceae;o__Peridiniales;f__Thoracosphaeraceae;g__Scrippsiella;s__Scrippsiella_trochoidea |
| OTU1907 | 1 | 500 | HM483396 | 92.83 | 516 | 100 | d__Eukaryota;k__Alveolata;p__norank;c__Dinophyceae;o__Peridiniales;f__Thoracosphaeraceae;g__Scrippsiella;s__Scrippsiella_trochoidea |
| OTU1913 | 1 | 495 | AF260393 | 94.55 | 495 | 100 | d__Eukaryota;k__Alveolata;p__norank;c__Dinophyceae;o__Peridiniales;f__Thoracosphaeraceae;g__Scrippsiella;s__Scrippsiella_trochoidea |
| OTU199 | 1 | 525 | AY245689 | 96.88 | 32 | 81 | d__Eukaryota;k__Alveolata;p__norank;c__Dinophyceae;o__Peridiniales;f__Pfiesteriaceae;g__unclassified_Pfiesteriaceae;s__Pfiesteria-like_DINO_Lucy |
| OTU2046 | 1 | 423 | EU165312 | 88.86 | 431 | 100 | d__Eukaryota;k__Alveolata;p__norank;c__Dinophyceae;o__Peridiniales;f__Heterocapsaceae;g__Heterocapsa;s__Heterocapsa_rotundata |
| OTU2062 | 1 | 474 | JF521638 | 90.32 | 475 | 100 | d__Eukaryota;k__Alveolata;p__norank;c__Dinophyceae;o__Gonyaulacales;f__Gonyaulacaceae;g__Alexandrium;s__Alexandrium_pseudogoniaulax |
| OTU226 | 1 | 486 | AY154960 | 97.54 | 407 | 83 | d__Eukaryota;k__Alveolata;p__norank;c__Dinophyceae;o__Gonyaulacales;f__Gonyaulacaceae;g__Gonyaulax;s__Gonyaulax_cf._spinifera |
| OTU2269 | 1 | 497 | JQ247713 | 83 | 500 | 100 | d__Eukaryota;k__Alveolata;p__norank;c__Dinophyceae;o__Peridiniales;f__Heterocapsaceae;g__Heterocapsa;s__Heterocapsa_niei |
| OTU2310 | 1 | 533 | JN606065 | 98.12 | 533 | 100 | d__Eukaryota;k__Alveolata;p__norank;c__Dinophyceae;o__Syndiniales;f__Euduboscquellidae;g__Euduboscquella;s__Euduboscquella_crenulata |
| OTU2350 | 1 | 499 | FR877580 | 96.99 | 499 | 100 | d__Eukaryota;k__Alveolata;p__norank;c__Dinophyceae;o__Gonyaulacales;f__Amphidomataceae;g__Azadinium;s__Azadinium_cf._poporum_HJ-2011 |

| | | | | | | | |
|---|---|---|---|---|---|---|---|
| OTU2560 | 1 | 508 | HQ902267 | 73.01 | 515 | 98 | d__Eukaryota;k__Alveolata;p__norank;c__Dinophyceae;o__Peridiniales;f__Heterocapsaceae;g__Heterocapsa;s__Heterocapsa_triquetra |
| OTU2572 | 1 | 484 | HM483396 | 91.21 | 478 | 99 | d__Eukaryota;k__Alveolata;p__norank;c__Dinophyceae;o__Peridiniales;f__Thoracosphaeraceae;g__Scrippsiella;s__Scrippsiella_trochoidea |
| OTU2582 | 1 | 505 | HQ902267 | 73.15 | 514 | 99 | d__Eukaryota;k__Alveolata;p__norank;c__Dinophyceae;o__Peridiniales;f__Heterocapsaceae;g__Heterocapsa;s__Heterocapsa_triquetra |
| OTU2971 | 1 | 504 | JN119844 | 83.2 | 506 | 100 | d__Eukaryota;k__Alveolata;p__norank;c__Dinophyceae;o__Peridiniales;f__Heterocapsaceae;g__Heterocapsa;s__Heterocapsa_sp._HCBC88 |
| OTU3307 | 1 | 491 | KF878935 | 96.96 | 493 | 100 | d__Eukaryota;k__Alveolata;p__norank;c__Dinophyceae;o__Gymnodiniales;f__Gymnodiniaceae;g__Gymnodinium;s__Gymnodinium_instriatum |
| OTU3452 | 1 | 541 | LC002848 | 87.36 | 87 | 43 | d__Eukaryota;k__Alveolata;p__norank;c__Dinophyceae;o__unclassified_Dinophyceae;f__norank;g__Pseudadenoides;s__Unclassified DINO |
| OTU3512 | 1 | 484 | AB860180 | 84.88 | 496 | 100 | d__Eukaryota;k__Alveolata;p__norank;c__Dinophyceae;o__Gymnodiniales;f__Gymnodiniaceae;g__Gymnodinium;s__unclassified_Gymnodinium |
| OTU357 | 1 | 328 | AY590477 | 98.79 | 330 | 100 | d__Eukaryota;k__Alveolata;p__norank;c__Dinophyceae;o__Peridiniales;f__Pfiesteriaceae;g__unclassified_Pfiesteriaceae;s__Pfiesteriaceae_sp._CCMP1835 |
| OTU3689 | 1 | 262 | FJ808690 | 80 | 200 | 75 | d__Eukaryota;k__Alveolata;p__norank;c__Dinophyceae;o__Dinophysiales;f__Dinophysiaceae;g__Dinophysis;s__Dinophysis_sp. |
| OTU3711 | 1 | 514 | EU490149 | 72.58 | 507 | 96 | d__Eukaryota;k__Alveolata;p__norank;c__Dinophyceae;o__norank;f__norank;g__norank;s__unclassified DINO |
| OTU461 | 1 | 478 | AY154965 | 74.14 | 406 | 81 | d__Eukaryota;k__Alveolata;p__norank;c__Dinophyceae;o__Gonyaulacales;f__Gonyaulacaceae;g__Gonyaulax;s__Gonyaulax_sp. |
| OTU508 | 1 | 444 | DQ286734 | 100 | 444 | 100 | d__Eukaryota;k__Alveolata;p__norank;c__Dinophyceae;o__Gymnodiniales;f__Gymnodiniaceae;g__Gymnodinium;s__Gymnodinium_catenatum |
| OTU755 | 1 | 415 | X16108.1 | 77 | 75 | 69 | d__Eukaryota;k__Alveolata;p__norank;c__Dinophyceae;o__norank;f__norank;g__norank;s__Unclassified DINO |
| OTU98 | 1 | 498 | EF616464 | 92.65 | 68 | 61 | d__Eukaryota;k__Alveolata;p__norank;c__Dinophyceae;o__Lophodiniales;f__Lophodiniaceae;g__Woloszynskia;s__Woloszynskia-like_sp. |
| OTU984 | 1 | 501 | EF616462 | 84.1 | 497 | 99 | d__Eukaryota;k__Alveolata;p__norank;c__Dinophyceae;o__Gymnodiniales;f__Gymnodiniaceae;g__Cochlodinium;s__Cochlodinium_cf._geminatum |
| OTU105 | 1 | 251 | AF318248 | 96.41 | 251 | 99 | d__Eukaryota;k__Alveolata;p__norank;c__Dinophyceae;o__Gymnodiniales;f__Gymnodiniaceae;g__Gymnodinium;s__unclassified_Gymnodinium |
| OTU1091 | 1 | 514 | HQ902267 | 76.44 | 556 | 100 | d__Eukaryota;k__Alveolata;p__norank;c__Dinophyceae;o__Peridiniales;f__Heterocapsaceae;g__Heterocapsa;s__Heterocapsa_triquetra |
| OTU110 | 1 | 413 | KF031312 | 76.43 | 420 | 99 | d__Eukaryota;k__Alveolata;p__norank;c__Dinophyceae;o__Peridiniales;f__Heterocapsaceae;g__Heterocapsa;s__Heterocapsa_minima |
| OTU1150 | 1 | 317 | EU165289 | 97.16 | 317 | 100 | d__Eukaryota;k__Alveolata;p__norank;c__Dinophyceae;o__Peridiniales;f__Thoracosphaeraceae;g__Scrippsiella;s__Scrippsiella_sp._CCMP2775 |
| OTU1158 | 1 | 236 | HQ845331 | 94.92 | 236 | 100 | d__Eukaryota;k__Alveolata;p__norank;c__Dinophyceae;o__Peridiniales;f__Thoracosphaeraceae;g__Scrippsiella;s__Scrippsiella_sweeneyae |

| | | | | | | | |
|---|---|---|---|---|---|---|---|
| OTU1180 | 1 | 295 | EU370959 | 96.96 | 296 | 100 | d__Eukaryota;k__Alveolata;p__norank;c__Dinophyceae;o__Peridiniales;f__Thoracosphaeraceae;g__Scrippsiella;s__Scrippsiella_trochoidea |
| OTU1339 | 1 | 485 | FJ024705 | 94.02 | 485 | 100 | d__Eukaryota;k__Alveolata;p__norank;c__Dinophyceae;o__Suessiales;f__Biecheleriaceae;g__Biecheleria;s__Biecheleria_cincta |
| OTU1356 | 1 | 493 | FJ024705 | 93.1 | 493 | 100 | d__Eukaryota;k__Alveolata;p__norank;c__Dinophyceae;o__Suessiales;f__Biecheleriaceae;g__Biecheleria;s__Biecheleria_cincta |
| OTU1613 | 1 | 461 | EF205003 | 83.72 | 86 | 53 | d__Eukaryota;k__Alveolata;p__norank;c__Dinophyceae;o__norank;f__norank;g__norank;s__Unclassified DINO |
| OTU1794 | 1 | 499 | HM483396 | 96.79 | 498 | 99 | d__Eukaryota;k__Alveolata;p__norank;c__Dinophyceae;o__Peridiniales;f__Thoracosphaeraceae;g__Scrippsiella;s__Scrippsiella_trochoidea |
| OTU1853 | 1 | 494 | EF152961 | 91.5 | 494 | 100 | d__Eukaryota;k__Alveolata;p__norank;c__Dinophyceae;o__Peridiniales;f__Diplopsaliaceae;g__Preperidinium;s__Protoperidinium_cf._steidingerae |
| OTU2042 | 1 | 512 | HQ902267 | 82.91 | 515 | 99 | d__Eukaryota;k__Alveolata;p__norank;c__Dinophyceae;o__Peridiniales;f__Heterocapsaceae;g__Heterocapsa;s__Heterocapsa_triquetra |
| OTU2059 | 1 | 416 | JF521637 | 98.56 | 417 | 100 | d__Eukaryota;k__Alveolata;p__norank;c__Dinophyceae;o__Gonyaulacales;f__Gonyaulacaceae;g__Alexandrium;s__Alexandrium_ostenfeldii |
| OTU2078 | 1 | 515 | JN119844 | 84.17 | 518 | 100 | d__Eukaryota;k__Alveolata;p__norank;c__Dinophyceae;o__Peridiniales;f__Heterocapsaceae;g__Heterocapsa;s__Heterocapsa_sp._HCBC88 |
| OTU236 | 1 | 473 | AY154960 | 81.54 | 65 | 47 | d__Eukaryota;k__Alveolata;p__norank;c__Dinophyceae;o__Gonyaulacales;f__Gonyaulacaceae;g__norank;s__Unclassified DINO |
| OTU2455 | 1 | 506 | FJ024705 | 88.39 | 508 | 100 | d__Eukaryota;k__Alveolata;p__norank;c__Dinophyceae;o__Suessiales;f__Biecheleriaceae;g__Biecheleria;s__Biecheleria_cincta |
| OTU2496 | 1 | 517 | KP790208 | 89.47 | 76 | 15 | d__Eukaryota;k__Alveolata;p__norank;c__Dinophyceae;o__norank;f__norank;g__norank;s__Unclassifid DINO |
| OTU2644 | 1 | 513 | JX559886 | 99.1 | 442 | 86 | d__Eukaryota;k__Alveolata;p__norank;c__Dinophyceae;o__Gonyaulacales;f__Amphidomataceae;g__Azadinium;s__Azadinium_polongum |
| OTU2768 | 1 | 501 | JQ247713 | 73.05 | 512 | 100 | d__Eukaryota;k__Alveolata;p__norank;c__Dinophyceae;o__Peridiniales;f__Heterocapsaceae;g__Heterocapsa;s__Heterocapsa_niei |
| OTU2984 | 1 | 519 | KF543359 | 96.29 | 431 | 83 | d__Eukaryota;k__Alveolata;p__norank;c__Dinophyceae;o__Gonyaulacales;f__Amphidomataceae;g__Azadinium;s__Azadinium_dalianense |
| OTU3033 | 1 | 509 | KF651047 | 94.5 | 509 | 100 | d__Eukaryota;k__Alveolata;p__norank;c__Dinophyceae;o__Peridiniales;f__Protoperidiniaceae;g__Protoperidinium;s__Protoperidinium_tricingulatum |
| OTU3344 | 1 | 323 | KF751924 | 96.6 | 324 | 100 | d__Eukaryota;k__Alveolata;p__norank;c__Dinophyceae;o__Peridiniales;f__Thoracosphaeraceae;g__Scrippsiella;s__Scrippsiella_aff._acuminata |
| OTU3364 | 1 | 279 | KF751924 | 97.84 | 278 | 99 | d__Eukaryota;k__Alveolata;p__norank;c__Dinophyceae;o__Peridiniales;f__Thoracosphaeraceae;g__Scrippsiella;s__Scrippsiella_aff._acuminata |
| OTU337 | 1 | 497 | JX262498 | 89.16 | 498 | 100 | d__Eukaryota;k__Alveolata;p__norank;c__Dinophyceae;o__Peridiniales;f__Peridiniaceae;g__Pentapharsodinium;s__Pentapharsodinium_dalei |
| OTU3391 | 1 | 423 | KF751926 | 95.72 | 421 | 99 | d__Eukaryota;k__Alveolata;p__norank;c__Dinophyceae;o__Peridiniales;f__Thoracosphaeraceae;g__Scrippsiella;s__Scrippsiella_sp. |

| | | | | | | | |
|---|---|---|---|---|---|---|---|
| OTU3402 | 1 | 496 | AB858353 | 92.96 | 497 | 100 | d__Eukaryota;k__Alveolata;p__norank;c__Dinophyceae;o__Suessiales;f__Biecheleriaceae;g__Biecheleria;s__Biecheleria_brevisulcata |
| OTU3446 | 1 | 485 | LM992905 | 100 | 339 | 70 | d__Eukaryota;k__Alveolata;p__norank;c__Dinophyceae;o__Lophodiniales;f__Lophodiniaceae;g__Biecheleriopsis;s__Biecheleriopsis_adriatica |
| OTU361 | 1 | 413 | LC027052 | 76.64 | 244 | 58 | d__Eukaryota;k__Alveolata;p__norank;c__Dinophyceae;o__Gymnodiniales;f__Gymnodiniaceae;g__Unclassified;s__Unclassified_Gymnodiniaceae |
| OTU3712 | 1 | 494 | EU490149 | 72.55 | 499 | 98 | d__Eukaryota;k__Alveolata;p__norank; c__Dinophyceae;o__norank;f__norank;g__norank;s__unclassified DINO |
| OTU683 | 1 | 455 | KP702719 | 95.83 | 456 | 100 | d__Eukaryota;k__Alveolata;p__norank;c__Dinophyceae;o__Peridiniales;f__Diplopsaliaceae;g__Oblea;s__Oblea_rotunda |
| OTU980 | 1 | 497 | EF616462 | 94.74 | 133 | 90 | d__Eukaryota;k__Alveolata;p__norank;c__Dinophyceae;o__Gymnodiniales;f__Gymnodiniaceae;g__Cochlodinium;s__Cochlodinium_cf._geminatum-like |
| OTU1066 | 1 | 507 | JN119844 | 76.14 | 549 | 100 | d__Eukaryota;k__Alveolata;p__norank;c__Dinophyceae;o__Peridiniales;f__Heterocapsaceae;g__Heterocapsa;s__Heterocapsa_sp._HCBC88 |
| OTU1151 | 1 | 261 | EU165289 | 98.43 | 255 | 98 | d__Eukaryota;k__Alveolata;p__norank;c__Dinophyceae;o__Peridiniales;f__Thoracosphaeraceae;g__Scrippsiella;s__Scrippsiella_sp._CCMP2775 |
| OTU1183 | 1 | 487 | AY154965 | 74.64 | 414 | 81 | d__Eukaryota;k__Alveolata;p__norank;c__Dinophyceae;o__Gonyaulacales;f__Gonyaulacaceae;g__Gonyaulax;s__Gonyaulax-like_sp. |
| OTU12 | 1 | 523 | EU126801 | 74.54 | 538 | 100 | d__Eukaryota;k__Alveolata;p__norank;c__Dinophyceae;o__Suessiales;f__Borghiellaceae;g__Borghiella;s__Borghiella_sp. |
| OTU1328 | 1 | 510 | FJ024705 | 91.73 | 508 | 99 | d__Eukaryota;k__Alveolata;p__norank;c__Dinophyceae;o__Suessiales;f__Biecheleriaceae;g__Biecheleria;s__Biecheleria_cincta |
| OTU1337 | 1 | 499 | FJ024705 | 94.19 | 499 | 100 | d__Eukaryota;k__Alveolata;p__norank;c__Dinophyceae;o__Suessiales;f__Biecheleriaceae;g__Biecheleria;s__Biecheleria_cincta |
| OTU1347 | 1 | 499 | FJ024705 | 92.22 | 501 | 100 | d__Eukaryota;k__Alveolata;p__norank;c__Dinophyceae;o__Suessiales;f__Biecheleriaceae;g__Biecheleria;s__Biecheleria_cincta |
| OTU1360 | 1 | 491 | FJ024705 | 92.68 | 492 | 100 | d__Eukaryota;k__Alveolata;p__norank;c__Dinophyceae;o__Suessiales;f__Biecheleriaceae;g__Biecheleria;s__Biecheleria_cincta |
| OTU1520 | 1 | 484 | FJ939579 | 72.27 | 494 | 99 | d__Eukaryota;k__Alveolata;p__norank;c__Dinophyceae;o__Gonyaulacales;f__Gonyaulacaceae;g__Gonyaulax;s__Gonyaulax_sp. |
| OTU1607 | 1 | 503 | FJ600087 | 88.66 | 529 | 100 | d__Eukaryota;k__Alveolata;p__norank;c__Dinophyceae;o__Peridiniales;f__Pfiesteriaceae;g__Pfiesteria;s__Pfiesteria_piscicida |
| OTU1620 | 1 | 334 | LC027066 | 82.08 | 106 | 32 | d__Eukaryota;k__Alveolata;p__norank;c__Dinophyceae;o__norank;f__norank;g__norank;s__Unclassified DINO |
| OTU1776 | 1 | 482 | HM483396 | 92.95 | 482 | 100 | d__Eukaryota;k__Alveolata;p__norank;c__Dinophyceae;o__Peridiniales;f__Thoracosphaeraceae;g__Scrippsiella;s__Scrippsiella_trochoidea |
| OTU1799 | 1 | 503 | HM483396 | 89.66 | 503 | 100 | d__Eukaryota;k__Alveolata;p__norank;c__Dinophyceae;o__Peridiniales;f__Thoracosphaeraceae;g__Scrippsiella;s__Scrippsiella_trochoidea |
| OTU1842 | 1 | 516 | HM483396 | 94.79 | 518 | 100 | d__Eukaryota;k__Alveolata;p__norank;c__Dinophyceae;o__Peridiniales;f__Thoracosphaeraceae;g__Scrippsiella;s__Scrippsiella_trochoidea |

| | | | | | | | |
|---|---|---|---|---|---|---|---|
| OTU1879 | 1 | 436 | HM483396 | 89.33 | 431 | 99 | d__Eukaryota;k__Alveolata;p__norank;c__Dinophyceae;o__Peridiniales;f__Thoracosphaeraceae;g__Scrippsiella;s__Scrippsiella_trochoidea |
| OTU1897 | 1 | 498 | HM483396 | 90.56 | 498 | 100 | d__Eukaryota;k__Alveolata;p__norank;c__Dinophyceae;o__Peridiniales;f__Thoracosphaeraceae;g__Scrippsiella;s__Scrippsiella_trochoidea |
| OTU1912 | 1 | 507 | HM483396 | 95.87 | 508 | 100 | d__Eukaryota;k__Alveolata;p__norank;c__Dinophyceae;o__Peridiniales;f__Thoracosphaeraceae;g__Scrippsiella;s__Scrippsiella_trochoidea |
| OTU2000 | 1 | 494 | FR720082 | 80.72 | 498 | 100 | d__Eukaryota;k__Alveolata;p__norank;c__Dinophyceae;o__Gymnodiniales;f__Gymnodiniaceae;g__Gyrodiniellum;s__Gyrodiniellum_sp. |
| OTU2044 | 1 | 502 | HQ902267 | 82.9 | 503 | 100 | d__Eukaryota;k__Alveolata;p__norank;c__Dinophyceae;o__Peridiniales;f__Heterocapsaceae;g__Heterocapsa;s__Heterocapsa_triquetra |
| OTU2068 | 1 | 502 | JF430394 | 92.63 | 502 | 100 | d__Eukaryota;k__Alveolata;p__norank;c__Dinophyceae;o__Peridiniales;f__Pfiesteriaceae;g__Chimonodinium;s__Chimonodinium_lomnickii |
| OTU210 | 1 | 426 | EU165272 | 80.12 | 322 | 75 | d__Eukaryota;k__Alveolata;p__norank;c__Dinophyceae;o__Peridiniales;f__Heterocapsaceae;g__unclassified_Heterocapsaceae;s__Heterocapsaceae-like sp. |
| OTU2110 | 1 | 436 | HQ670228 | 95.86 | 435 | 99 | d__Eukaryota;k__Alveolata;p__norank;c__Dinophyceae;o__Peridiniales;f__Thoracosphaeraceae;g__Scrippsiella;s__Scrippsiella_trochoidea |
| OTU2188 | 1 | 494 | FN647674 | 98.1 | 421 | 85 | d__Eukaryota;k__Alveolata;p__norank;c__Dinophyceae;o__Gymnodiniales;f__Gymnodiniaceae;g__Barrufeta;s__Barrufeta_bravensis |
| OTU2196 | 1 | 479 | DQ195375 | 75.21 | 480 | 99 | d__Eukaryota;k__Alveolata;p__norank;c__Dinophyceae;o__Suessiales;f__Suessiaceae;g__Pelagodinium;s__Pelagodinium_sp. |
| OTU2202 | 1 | 478 | JN558107 | 96.44 | 478 | 100 | d__Eukaryota;k__Alveolata;p__norank;c__Dinophyceae;o__Suessiales;f__Suessiaceae;g__Pelagodinium;s__Pelagodinium_béii |
| OTU2316 | 1 | 542 | JN934984 | 96.68 | 542 | 100 | d__Eukaryota;k__Alveolata;p__norank;c__Dinophyceae;o__Syndiniales;f__Euduboscquellidae;g__Euduboscquella;s__Euduboscquella_sp._ex_Tintinnopsis_sp._1 |
| OTU2321 | 1 | 359 | JN934984 | 94.74 | 361 | 100 | d__Eukaryota;k__Alveolata;p__norank;c__Dinophyceae;o__Syndiniales;f__Euduboscquellidae;g__Euduboscquella;s__Euduboscquella_sp._ex_Tintinnopsis_sp._1 |
| OTU2325 | 1 | 561 | JN934988 | 96.8 | 563 | 100 | d__Eukaryota;k__Alveolata;p__norank;c__Dinophyceae;o__Syndiniales;f__Euduboscquellidae;g__Euduboscquella;s__Euduboscquella_cachoni |
| OTU2332 | 1 | 498 | JN934989 | 69.26 | 501 | 99 | d__Eukaryota;k__Alveolata;p__norank;c__Dinophyceae;o__Syndiniales;f__Euduboscquellidae;g__Euduboscquella;s__Euduboscquella_sp._ex_Favella_arcuata |
| OTU2344 | 1 | 540 | JN934994 | 99.63 | 540 | 100 | d__Eukaryota;k__Alveolata;p__norank;c__Dinophyceae;o__Syndiniales;f__Euduboscquellidae;g__Euduboscquella;s__Euduboscquella_sp._ex_Tintinnopsis_sp._2 |
| OTU2428 | 1 | 235 | JQ639752 | 85.71 | 189 | 80 | d__Eukaryota;k__Alveolata;p__norank;c__Dinophyceae;o__Peridiniales;f__Glenodiniaceae;g__Peridiniopsis;s__Peridiniopsis_minima |
| OTU2447 | 1 | 502 | FJ024705 | 88.14 | 506 | 100 | d__Eukaryota;k__Alveolata;p__norank;c__Dinophyceae;o__Suessiales;f__Biecheleriaceae;g__Biecheleria;s__Biecheleria_cincta |
| OTU2451 | 1 | 481 | FJ024705 | 91.12 | 484 | 100 | d__Eukaryota;k__Alveolata;p__norank;c__Dinophyceae;o__Suessiales;f__Biecheleriaceae;g__Biecheleria;s__Biecheleria_cincta |
| OTU2456 | 1 | 444 | JQ413374 | 96.26 | 454 | 100 | d__Eukaryota;k__Alveolata;p__norank;c__Dinophyceae;o__Suessiales;f__Biecheleriaceae;g__Biecheleria;s__Biecheleria_cincta |

| | | | | | | | |
|---|---|---|---|---|---|---|---|
| OTU2458 | 1 | 505 | AB858353 | 91.68 | 505 | 100 | d__Eukaryota;k__Alveolata;p__norank;c__Dinophyceae;o__Suessiales;f__Biecheleriaceae;g__Biecheleria;s__Biecheleria_brevisulcata |
| OTU2537 | 1 | 477 | HQ845327 | 88 | 84 | 23 | d__Eukaryota;k__Alveolata;p__norank;c__Dinophyceae;o__norank;f__norank;g__norank;s__Unclassified DINO |
| OTU2583 | 1 | 464 | AF260400 | 71.22 | 476 | 99 | d__Eukaryota;k__Alveolata;p__norank;c__Dinophyceae;o__Peridiniales;f__Heterocapsaceae;g__Heterocapsa;s__Heterocapsa_rotundata |
| OTU2641 | 1 | 299 | JN982402 | 97.57 | 288 | 96 | d__Eukaryota;k__Alveolata;p__norank;c__Dinophyceae;o__Peridiniales;f__Thoracosphaeraceae;g__Scrippsiella;s__Scrippsiella_sp. |
| OTU2666 | 1 | 490 | AB716928 | 91.65 | 491 | 100 | d__Eukaryota;k__Alveolata;p__norank;c__Dinophyceae;o__Peridiniales;f__Protoperidiniaceae;g__Protoperidinium;s__Protoperidinium_monovelum |
| OTU2669 | 1 | 359 | AB716928 | 86.35 | 359 | 100 | d__Eukaryota;k__Alveolata;p__norank;c__Dinophyceae;o__Peridiniales;f__Protoperidiniaceae;g__Protoperidinium;s__Protoperidinium_monovelum |
| OTU2711 | 1 | 473 | JX262498 | 73 | 473 | 100 | d__Eukaryota;k__Alveolata;p__norank;c__Dinophyceae;o__Peridiniales;f__Peridiniaceae;g__Pentapharsodinium;s__Pentapharsodinium_sp. |
| OTU2808 | 1 | 219 | KC895475 | 96.36 | 220 | 100 | d__Eukaryota;k__Alveolata;p__norank;c__Dinophyceae;o__Suessiales;f__Biecheleriaceae;g__Biecheleria;s__Biecheleria_cincta |
| OTU2856 | 1 | 478 | KP790181 | 89.42 | 104 | 22 | d__Eukaryota;k__Alveolata;p__norank;c__Dinophyceae;o__norank;f__norank;g__norank;s__Unclassified DINO |
| OTU2953 | 1 | 491 | KF031312 | 99.32 | 443 | 90 | d__Eukaryota;k__Alveolata;p__norank;c__Dinophyceae;o__Peridiniales;f__Heterocapsaceae;g__Heterocapsa;s__Heterocapsa_minima |
| OTU3287 | 1 | 499 | EF616462 | 86.07 | 524 | 100 | d__Eukaryota;k__Alveolata;p__norank;c__Dinophyceae;o__Gymnodiniales;f__Gymnodiniaceae;g__Cochlodinium;s__Cochlodinium_cf._geminatum |
| OTU3288 | 1 | 500 | HQ834210 | 84.79 | 539 | 100 | d__Eukaryota;k__Alveolata;p__norank;c__Dinophyceae;o__Gymnodiniales;f__Polykrikaceae;g__Pheopolykrikos;s__Pheopolykrikos_hartmannii |
| OTU3328 | 1 | 499 | HM483396 | 92.99 | 499 | 100 | d__Eukaryota;k__Alveolata;p__norank;c__Dinophyceae;o__Peridiniales;f__Thoracosphaeraceae;g__Scrippsiella;s__Scrippsiella_trochoidea |
| OTU3335 | 1 | 519 | KF751924 | 89.21 | 519 | 99 | d__Eukaryota;k__Alveolata;p__norank;c__Dinophyceae;o__Peridiniales;f__Thoracosphaeraceae;g__Scrippsiella;s__Scrippsiella_aff._acuminata |
| OTU334 | 1 | 497 | AF260392 | 86.97 | 499 | 100 | d__Eukaryota;k__Alveolata;p__norank;c__Dinophyceae;o__Peridiniales;f__Thoracosphaeraceae;g__Scrippsiella;s__Scrippsiella_sp. |
| OTU3365 | 1 | 410 | KF751924 | 90.64 | 406 | 99 | d__Eukaryota;k__Alveolata;p__norank;c__Dinophyceae;o__Peridiniales;f__Thoracosphaeraceae;g__Scrippsiella;s__Scrippsiella_aff._acuminata |
| OTU3377 | 1 | 425 | KF751926 | 96.47 | 425 | 100 | d__Eukaryota;k__Alveolata;p__norank;c__Dinophyceae;o__Peridiniales;f__Thoracosphaeraceae;g__Scrippsiella;s__Scrippsiella_sp. |
| OTU3385 | 1 | 495 | HM483396 | 94.34 | 495 | 100 | d__Eukaryota;k__Alveolata;p__norank;c__Dinophyceae;o__Peridiniales;f__Thoracosphaeraceae;g__Scrippsiella;s__Scrippsiella_trochoidea |
| OTU3407 | 1 | 501 | AB858353 | 88.42 | 501 | 100 | d__Eukaryota;k__Alveolata;p__norank;c__Dinophyceae;o__Suessiales;f__Biecheleriaceae;g__Biecheleria;s__Biecheleria_brevisulcata |
| OTU3408 | 1 | 225 | AB858353 | 99.06 | 213 | 94 | d__Eukaryota;k__Alveolata;p__norank;c__Dinophyceae;o__Suessiales;f__Biecheleriaceae;g__Biecheleria;s__Biecheleria_brevisulcata |

| | | | | | | | |
|---|---|---|---|---|---|---|---|
| OTU3413 | 1 | 495 | AB858353 | 89.54 | 497 | 100 | d__Eukaryota;k__Alveolata;p__norank;c__Dinophyceae;o__Suessiales;f__Biecheleriaceae;g__Biecheleria;s__Biecheleria_brevisulcata |
| OTU342 | 1 | 481 | AY571374 | 99.5 | 398 | 83 | d__Eukaryota;k__Alveolata;p__norank;c__Dinophyceae;o__Lophodiniales;f__Lophodiniaceae;g__Woloszynskia;s__Woloszynskia_tenuissima-like |
| OTU3443 | 1 | 489 | LM992906 | 91.46 | 492 | 100 | d__Eukaryota;k__Alveolata;p__norank;c__Dinophyceae;o__Lophodiniales;f__Lophodiniaceae;g__Biecheleriopsis;s__Biecheleriopsis_adriatica |
| OTU3490 | 1 | 501 | HG005135 | 92.38 | 499 | 99 | d__Eukaryota;k__Alveolata;p__norank;c__Dinophyceae;o__Gymnodiniales;f__Gymnodiniaceae;g__Gymnodinium;s__unclassified_Gymnodinium |
| OTU3509 | 1 | 483 | HG005135 | 99.17 | 483 | 100 | d__Eukaryota;k__Alveolata;p__norank;c__Dinophyceae;o__Gymnodiniales;f__Gymnodiniaceae;g__Gymnodinium;s__Gymnodinium_sp. GSSW10 |
| OTU3530 | 1 | 497 | KJ433986 | 87.62 | 501 | 99 | d__Eukaryota;k__Alveolata;p__norank;c__Dinophyceae;o__Peridiniales;f__Peridiniaceae;g__Pentapharsodinium;s__Pentapharsodinium_sp._ZL-2014 |
| OTU3535 | 1 | 437 | KJ433986 | 90.13 | 476 | 100 | d__Eukaryota;k__Alveolata;p__norank;c__Dinophyceae;o__Peridiniales;f__Peridiniaceae;g__Pentapharsodinium;s__Pentapharsodinium_sp._ZL-2014 |
| OTU3538 | 1 | 466 | KJ433986 | 90.79 | 467 | 100 | d__Eukaryota;k__Alveolata;p__norank;c__Dinophyceae;o__Peridiniales;f__Peridiniaceae;g__Pentapharsodinium;s__Pentapharsodinium_sp._ZL-2014 |
| OTU355 | 1 | 505 | DQ991376 | 95.86 | 507 | 100 | d__Eukaryota;k__Alveolata;p__norank;c__Dinophyceae;o__Peridiniales;f__Pfiesteriaceae;g__Cryptoperidiniopsis;s__Cryptoperidiniopsis_brodyi |
| OTU3602 | 1 | 486 | KP790206 | 97.24 | 434 | 89 | d__Eukaryota;k__Alveolata;p__norank;c__Dinophyceae;o__Gymnodiniales;f__Gymnodiniaceae;g__Gyrodinium;s__Gyrodinium_undulans |
| OTU3686 | 1 | 495 | JX262498 | 75 | 356 | 72 | d__Eukaryota;k__Alveolata;p__norank;c__Dinophyceae;o__Peridiniales; f__Peridiniaceae;g__Pentapharsodinium;s__Pentapharsodinium_sp. |
| OTU3726 | 1 | 475 | HG792066 | 96.84 | 475 | 100 | d__Eukaryota;k__Alveolata;p__norank;c__Dinophyceae;o__unclassified_Dinophyceae;f__norank;g__norank;s__Dinophyceae_sp._1_HJH-2013 |
| OTU3757 | 1 | 480 | LC027049 | 98.34 | 481 | 100 | d__Eukaryota;k__Alveolata;p__norank;c__Dinophyceae;o__unclassified_Dinophyceae;f__norank;g__Pellucidodinium;s__Pellucidodinium_psammophilum |
| OTU3759 | 1 | 478 | LC027049 | 89.77 | 479 | 99 | d__Eukaryota;k__Alveolata;p__norank;c__Dinophyceae;o__unclassified_Dinophyceae;f__norank;g__Pellucidodinium;s__Pellucidodinium_psammophilum |
| OTU3831 | 1 | 498 | JX262498 | 83.03 | 501 | 100 | d__Eukaryota;k__Alveolata;p__norank;c__Dinophyceae;o__Peridiniales;f__Peridiniaceae;g__Pentapharsodinium;s__Pentapharsodinium_dalei |
| OTU385 | 1 | 499 | AY916546 | 94.93 | 493 | 99 | d__Eukaryota;k__Alveolata;p__norank;c__Dinophyceae;o__Peridiniales;f__Thoracosphaeraceae;g__Scrippsiella;s__Scrippsiella_sp._CS297 |
| OTU398 | 1 | 472 | AY916553 | 96.33 | 463 | 98 | d__Eukaryota;k__Alveolata;p__norank;c__Dinophyceae;o__Lophodiniales;f__Lophodiniaceae;g__Woloszynskia;s__Woloszynskia_sp._CS-341 |
| OTU4 | 1 | 259 | KF245459 | 79.28 | 111 | 43 | d__Eukaryota;k__Alveolata;p__norank;c__Dinophyceae;o__unclassified;f__unclassified;g__unclassified;s__unclassified DINO |
| OTU470 | 1 | 378 | AY154965 | 73.74 | 396 | 99 | d__Eukaryota;k__Alveolata;p__norank;c__Dinophyceae;o__Gonyaulacales;f__Gonyaulacaceae;g__Gonyaulax;s__Gonyaulax_membranacea |
| OTU475 | 1 | 411 | AY154965 | 73.26 | 430 | 99 | d__Eukaryota;k__Alveolata;p__norank;c__Dinophyceae;o__Gonyaulacales;f__Gonyaulacaceae;g__Gonyaulax;s__Gonyaulax_membranacea |

| | | | | | | | |
|---|---|---|---|---|---|---|---|
| OTU499 | 1 | 447 | DQ195376 | 96.65 | 448 | 100 | d__Eukaryota;k__Alveolata;p__norank;c__Dinophyceae;o__Suessiales;f__Suessiaceae;g__Pelagodinium;s__Pelagodinium_béii |
| OTU535 | 1 | 435 | AM408889 | 79.27 | 439 | 100 | d__Eukaryota;k__Alveolata;p__norank;c__Dinophyceae;o__Gymnodiniales;f__Gymnodiniaceae;g__Paragymnodinium;s__Paragymnodinium_shiwhaense |
| OTU58 | 1 | 505 | AF260393 | 97.23 | 505 | 100 | d__Eukaryota;k__Alveolata;p__norank;c__Dinophyceae;o__Peridiniales;f__Thoracosphaeraceae;g__Scrippsiella;s__Scrippsiella_trochoidea |
| OTU60 | 1 | 498 | AF260393 | 94.19 | 396 | 79 | d__Eukaryota;k__Alveolata;p__norank;c__Dinophyceae;o__Peridiniales;f__Thoracosphaeraceae;g__Scrippsiella;s__Scrippsiella_sp. |
| OTU62 | 1 | 413 | AF260393 | 95.27 | 402 | 97 | d__Eukaryota;k__Alveolata;p__norank;c__Dinophyceae;o__Peridiniales;f__Thoracosphaeraceae;g__Scrippsiella;s__Scrippsiella_trochoidea |
| OTU702 | 1 | 527 | EF152961 | 90.74 | 529 | 100 | d__Eukaryota;k__Alveolata;p__norank;c__Dinophyceae;o__Peridiniales;f__Diplopsaliaceae;g__Preperidinium;s__Protoperidinium_cf._steidingerae |
| OTU71 | 1 | 341 | AF260393 | 96.44 | 337 | 99 | d__Eukaryota;k__Alveolata;p__norank;c__Dinophyceae;o__Peridiniales;f__Thoracosphaeraceae;g__Scrippsiella;s__Scrippsiella_trochoidea |
| OTU719 | 1 | 517 | EF152961 | 88.87 | 557 | 99 | d__Eukaryota;k__Alveolata;p__norank;c__Dinophyceae;o__Peridiniales;f__Diplopsaliaceae;g__Preperidinium;s__Protoperidinium_cf._steidingerae |
| OTU1996 | 2 | 482 | EF205002 | 85.9 | 78 | 38 | d__Eukaryota;k__Alveolata;p__norank;c__Dinophyceae;o__norank;f__norank;g__norank;s__Unclassified DINO |
| OTU3788 | 2 | 490 | KJ508397 | 95.74 | 493 | 100 | d__Eukaryota;k__Alveolata;p__norank;c__Dinophyceae;o__Gymnodiniales;f__Warnowiaceae;g__Warnowia;s__Warnowia_sp._IFR1101 |
| OTU1796 | 2 | 505 | HM483396 | 95.84 | 505 | 100 | d__Eukaryota;k__Alveolata;p__norank;c__Dinophyceae;o__Peridiniales;f__Thoracosphaeraceae;g__Scrippsiella;s__Scrippsiella_trochoidea |
| OTU2008 | 2 | 333 | EF205002 | 92.59 | 108 | 32 | d__Eukaryota;k__Alveolata;p__norank;c__Dinophyceae;o__norank;f__norank;g__norank;s__Unclassified DINO |
| OTU2190 | 2 | 477 | FN649409 | 91.96 | 112 | 23 | d__Eukaryota;k__Alveolata;p__norank;c__Dinophyceae;o__Gymnodiniales;f__Gymnodiniaceae;g__norank;s__Unclassified Gymnodinium-like_species |
| OTU1795 | 2 | 489 | HM483396 | 96.52 | 489 | 100 | d__Eukaryota;k__Alveolata;p__norank;c__Dinophyceae;o__Peridiniales;f__Thoracosphaeraceae;g__Scrippsiella;s__Scrippsiella_trochoidea |
| OTU1629 | 2 | 458 | KJ433986 | 72.79 | 463 | 99 | d__Eukaryota;k__Alveolata;p__norank;c__Dinophyceae;o__Peridiniales;f__Peridiniaceae;g__Pentapharsodinium;s__Pentapharsodinium_sp._ZL-2014 |
| OTU1779 | 2 | 503 | HM483396 | 97.01 | 501 | 99 | d__Eukaryota;k__Alveolata;p__norank;c__Dinophyceae;o__Peridiniales;f__Thoracosphaeraceae;g__Scrippsiella;s__Scrippsiella_trochoidea |
| OTU481 | 2 | 472 | JN558110 | 82.77 | 499 | 100 | d__Eukaryota;k__Alveolata;p__norank;c__Dinophyceae;o__Suessiales;f__Suessiaceae;g__Polarella;s__Polarella_glacialis |
| OTU2340 | 2 | 542 | JN934989 | 76.78 | 547 | 100 | d__Eukaryota;k__Alveolata;p__norank;c__Dinophyceae;o__Syndiniales;f__Euduboscquellidae;g__Euduboscquella;s__Euduboscquella_sp._ex_Favella_arcuata |
| OTU3696 | 2 | 507 | EU490149 | 73.41 | 504 | 96 | d__Eukaryota;k__Fungi;p__environmental_samples;c__Dinophyceae;o__norank;f__norank;g__norank;s__unclassified DINO |
| OTU1072 | 2 | 487 | AY571374 | 84.6 | 487 | 99 | d__Eukaryota;k__Alveolata;p__norank;c__Dinophyceae;o__Lophodiniales;f__Lophodiniaceae;g__Woloszynskia;s__Woloszynskia_tenuissima |

| | | | | | | | |
|---|---|---|---|---|---|---|---|
| OTU482 | 2 | 475 | DQ195346 | 93.47 | 475 | 100 | d__Eukaryota;k__Alveolata;p__norank;c__Dinophyceae;o__Suessiales;f__Suessiaceae;g__Pelagodinium;s__Pelagodinium_béii |
| OTU3338 | 2 | 496 | KF751924 | 96.57 | 496 | 100 | d__Eukaryota;k__Alveolata;p__norank;c__Dinophyceae;o__Peridiniales;f__Thoracosphaeraceae;g__Scrippsiella;s__Scrippsiella_aff._acuminata |
| OTU1866 | 2 | 358 | HM483396 | 92.46 | 358 | 100 | d__Eukaryota;k__Alveolata;p__norank;c__Dinophyceae;o__Peridiniales;f__Thoracosphaeraceae;g__Scrippsiella;s__Scrippsiella_trochoidea |
| OTU641 | 2 | 485 | AY571374 | 84.12 | 485 | 99 | d__Eukaryota;k__Alveolata;p__norank;c__Dinophyceae;o__Lophodiniales;f__Lophodiniaceae;g__Woloszynskia;s__Woloszynskia_tenuissima |
| OTU1105 | 2 | 505 | AY571374 | 81.84 | 523 | 100 | d__Eukaryota;k__Alveolata;p__norank;c__Dinophyceae;o__Lophodiniales;f__Lophodiniaceae;g__Woloszynskia;s__Woloszynskia_tenuissima |
| OTU1494 | 2 | 490 | FJ211386 | 98.98 | 490 | 100 | d__Eukaryota;k__Alveolata;p__norank;c__Dinophyceae;o__Gymnodiniales;f__Polykrikaceae;g__Polykrikos;s__Polykrikos_geminatum |
| OTU556 | 2 | 498 | DQ991376 | 95.6 | 500 | 100 | d__Eukaryota;k__Alveolata;p__norank;c__Dinophyceae;o__Peridiniales;f__Pfiesteriaceae;g__Cryptoperidiniopsis;s__Cryptoperidiniopsis_brodyi |
| OTU1792 | 2 | 503 | HM483396 | 96.21 | 501 | 99 | d__Eukaryota;k__Alveolata;p__norank;c__Dinophyceae;o__Peridiniales;f__Thoracosphaeraceae;g__Scrippsiella;s__Scrippsiella_trochoidea |
| OTU1789 | 2 | 513 | HM483396 | 95.13 | 513 | 100 | d__Eukaryota;k__Alveolata;p__norank;c__Dinophyceae;o__Peridiniales;f__Thoracosphaeraceae;g__Scrippsiella;s__Scrippsiella_trochoidea |
| OTU519 | 2 | 448 | DQ320627 | 98.21 | 448 | 100 | d__Eukaryota;k__Alveolata;p__norank;c__Dinophyceae;o__Gymnodiniales;f__Tovelliaceae;g__Tovellia;s__Tovellia_sanguinea |
| OTU505 | 2 | 437 | JQ439944 | 84.48 | 58 | 37 | d__Eukaryota;k__Alveolata;p__norank;c__Dinophyceae;o__Gymnodiniales;f__Unclassified;g__Unclassified;s__Unclassified_Gymnodiniales |
| OTU1083 | 2 | 488 | AY571374 | 83.5 | 491 | 99 | d__Eukaryota;k__Alveolata;p__norank;c__Dinophyceae;o__Lophodiniales;f__Lophodiniaceae;g__Woloszynskia;s__Woloszynskia_tenuissima |
| OTU679 | 2 | 487 | EF152794 | 85.04 | 488 | 100 | d__Eukaryota;k__Alveolata;p__norank;c__Dinophyceae;o__Peridiniales;f__Diplopsaliaceae;g__Diplopsalis;s__Diplopsalis_lenticula |
| OTU1925 | 2 | 492 | HM483399 | 88.66 | 494 | 100 | d__Eukaryota;k__Alveolata;p__norank;c__Dinophyceae;o__unclassified_Dinophyceae;f__norank;g__Duboscquodinium;s__Duboscquodinium_collinii |
| OTU1438 | 2 | 483 | FR720082.1 | 94 | 483 | 100 | d_Eukaryota;k_Alveolata;p_norank;c_Dinophyceae;o_Gymnodiniales;f_Gymnodiniaceae;g_Gyrodiniellum;s_Gyrodiniellum_shiwhaense |
| OTU1625 | 2 | 466 | EU126801.1 | 70 | 466 | 100 | d__Eukaryota;k__Alveolata;p__norank;c__Dinophyceae;o__Suessiales; ;f__Borghiellaceae;g__Borghiella;s__Borghiella__sp. |
| OTU1436 | 2 | 478 | EF616465.1 | 87 | 478 | 100 | d__Eukaryota;k__Alveolata;p__norank;c__Dinophyceae;o__Gymnodiniales;f_Gymnodiniaceae;g__Gymnodinium;s__Gymnodinium_impudicum-like |
| OTU976 | 2 | 512 | EF616462 | 86.16 | 513 | 99 | d__Eukaryota;k__Alveolata;p__norank;c__Dinophyceae;o__Gymnodiniales;f__Gymnodiniaceae;g__Cochlodinium;s__Cochlodinium_cf._geminatum |
| OTU2185 | 2 | 497 | FN647674 | 96.45 | 422 | 85 | d__Eukaryota;k__Alveolata;p__norank;c__Dinophyceae;o__Gymnodiniales;f__Gymnodiniaceae;g__Barrufeta;s__Barrufeta_bravensis |
| OTU1599 | 2 | 506 | FJ600087 | 93.48 | 506 | 100 | d__Eukaryota;k__Alveolata;p__norank;c__Dinophyceae;o__Peridiniales;f__Pfiesteriaceae;g__Pfiesteria;s__Pfiesteria_piscicida |

| | | | | | | | |
|---|---|---|---|---|---|---|---|
| OTU3467 | 2 | 490 | HG005135 | 94.29 | 490 | 100 | d__Eukaryota;k__Alveolata;p__norank;c__Dinophyceae;o__Gymnodiniales;f__Gymnodiniaceae;g__Gymnodinium;s__unclassified_Gymnodinium |
| OTU2962 | 2 | 491 | AY571372 | 75.36 | 491 | 98 | d__Eukaryota;k__Alveolata;p__norank;c__Dinophyceae;o__Peridiniales;f__Heterocapsaceae;g__Heterocapsa;s__Heterocapsa_arctica |
| OTU958 | 2 | 489 | EU165308 | 82.41 | 506 | 100 | d__Eukaryota;k__Alveolata;p__norank;c__Dinophyceae;o__Gymnodiniales;f__Kareniaceae;g__Karenia;s__Karenia_brevis |
| OTU953 | 2 | 527 | EF613367 | 98.67 | 527 | 100 | d__Eukaryota;k__Alveolata;p__norank;c__Dinophyceae;o__Gymnodiniales;f__Polykrikaceae;g__Polykrikos;s__Polykrikos_kofoidii |
| OTU1316 | 2 | 500 | KP790188 | 91.71 | 422 | 84 | d__Eukaryota;k__Alveolata;p__norank;c__Dinophyceae;o__Gymnodiniales;f__Gymnodiniaceae;g__Gymnodinium;s__Gymnodinium_sp. |
| OTU1101 | 2 | 489 | AY571374 | 79.11 | 517 | 100 | d__Eukaryota;k__Alveolata;p__norank;c__Dinophyceae;o__Lophodiniales;f__Lophodiniaceae;g__Woloszynskia;s__Woloszynskia_tenuissima |
| OTU2208 | 2 | 485 | JN558110 | 83 | 485 | 99 | d__Eukaryota;k__Alveolata;p__norank;c__Dinophyceae;o__Suessiales;f__Suessiaceae;g__Polarella;s__Polarella_sp. |
| OTU3835 | 2 | 480 | JX262498 | 80.93 | 493 | 100 | d__Eukaryota;k__Alveolata;p__norank;c__Dinophyceae;o__Peridiniales;f__Peridiniaceae;g__Pentapharsodinium;s__Pentapharsodinium_dalei |
| OTU2399 | 2 | 490 | JF921198 | 98.78 | 490 | 100 | d__Eukaryota;k__Alveolata;p__norank;c__Dinophyceae;o__Gonyaulacales;f__Gonyaulacaceae;g__Alexandrium;s__Alexandrium_peruvianum |
| OTU1202 | 2 | 487 | AF200669 | 79.84 | 501 | 99 | d__Eukaryota;k__Alveolata;p__norank;c__Dinophyceae;o__Gymnodiniales;f__Gymnodiniaceae;g__Lepidodinium;s__Lepidodinium_chlorophorum |
| OTU42 | 2 | 513 | AF260393 | 97.86 | 513 | 100 | d__Eukaryota;k__Alveolata;p__norank;c__Dinophyceae;o__Peridiniales;f__Thoracosphaeraceae;g__Scrippsiella;s__Scrippsiella_trochoidea |
| OTU2085 | 2 | 498 | HQ670228 | 95.38 | 498 | 100 | d__Eukaryota;k__Alveolata;p__norank;c__Dinophyceae;o__Peridiniales;f__Thoracosphaeraceae;g__Scrippsiella;s__Scrippsiella_trochoidea |
| OTU3720 | 2 | 479 | HG792066 | 100 | 479 | 100 | d__Eukaryota;k__Alveolata;p__norank;c__Dinophyceae;o__unclassified_Dinophyceae;f__norank;g__norank;s__Dinophyceae_sp._1_HJH-2013 |
| OTU185 | 2 | 518 | AY245689 | 97.68 | 517 | 99 | d__Eukaryota;k__Alveolata;p__norank;c__Dinophyceae;o__Peridiniales;f__Pfiesteriaceae;g__unclassified_Pfiesteriaceae;s__Pfiesteria-like_DINO_Lucy |
| OTU1451 | 2 | 552 | FJ160592 | 90.12 | 81 | 32 | d__Eukaryota;k__Alveolata;p__norank;c__Dinophyceae;o__norank;f__norank;g__norank;s__Unclassified DINO |
| OTU2028 | 2 | 458 | JN119844 | 74 | 500 | 100 | d__Eukaryota;k__Alveolata;p__norank;c__Dinophyceae;o__Peridiniales;f__Heterocapsaceae;g__Heterocapsa;s__Heterocapsa_sp._HCBC88 |
| OTU3704 | 2 | 522 | HM483397 | 85.32 | 88 | 54 | d__Eukaryota;k__Alveolata;p__norank;c__Dinophyceae;o__unclassified_Dinophyceae;f__norank;g__Tintinnophagus;s__unclassified DINO |
| OTU740 | 2 | 474 | EF205010 | 97.72 | 482 | 100 | d__Eukaryota;k__Alveolata;p__norank;c__Dinophyceae;o__Peridiniales;f__Glenodiniaceae;g__Peridiniopsis;s__Peridiniopsis_polonicum |
| OTU2674 | 2 | 492 | AB716928 | 89.11 | 496 | 100 | d__Eukaryota;k__Alveolata;p__norank;c__Dinophyceae;o__Peridiniales;f__Protoperidiniaceae;g__Protoperidinium;s__Protoperidinium_monovelum |
| OTU2717 | 2 | 498 | JX262498 | 98.6 | 357 | 72 | d__Eukaryota;k__Alveolata;p__norank;c__Dinophyceae;o__Peridiniales;f__Peridiniaceae;g__Pentapharsodinium;s__Pentapharsodinium_sp. |

| | | | | | | | |
|---|---|---|---|---|---|---|---|
| OTU548 | 2 | 373 | AB265968 | 83.91 | 379 | 100 | d__Eukaryota;k__Alveolata;p__norank;c__Dinophyceae;o__Gymnodiniales;f__Gymnodiniaceae;g__Gymnodinium;s__Gymnodinium_microreticulatum |
| OTU2213 | 2 | 469 | JN558110 | 90.04 | 472 | 100 | d__Eukaryota;k__Alveolata;p__norank;c__Dinophyceae;o__Suessiales;f__Suessiaceae;g__Polarella;s__Polarella_glacialis |
| OTU2710 | 2 | 507 | JX262498 | 89.15 | 507 | 100 | d__Eukaryota;k__Alveolata;p__norank;c__Dinophyceae;o__Peridiniales;f__Peridiniaceae;g__Pentapharsodinium;s__Pentapharsodinium_dalei |
| OTU2258 | 2 | 498 | HQ902267 | 71 | 531 | 100 | d__Eukaryota;k__Alveolata;p__norank;c__Dinophyceae;o__Peridiniales;f__Heterocapsaceae;g__Heterocapsa;s__Heterocapsa_triquetra |
| OTU3256 | 2 | 524 | AB778763 | 87 | 84 | 26 | d__Eukaryota;k__Alveolata; c__Dinophyceae;o__norank;f__norank;g__norank;s__Unclassified DINO |
| OTU3330 | 2 | 499 | HM483396 | 92.35 | 497 | 99 | d__Eukaryota;k__Alveolata;p__norank;c__Dinophyceae;o__Peridiniales;f__Thoracosphaeraceae;g__Scrippsiella;s__Scrippsiella_trochoidea |
| OTU2181 | 2 | 471 | HQ176320 | 86.86 | 472 | 100 | d__Eukaryota;k__Alveolata;p__norank;c__Dinophyceae;o__Peridiniales;f__Pfiesteriaceae;g__Chimonodinium;s__Chimonodinium_lomnickii |
| OTU1098 | 2 | 476 | AY571374 | 79.18 | 490 | 99 | d__Eukaryota;k__Alveolata;p__norank;c__Dinophyceae;o__Lophodiniales;f__Lophodiniaceae;g__Woloszynskia;s__Woloszynskia_tenuissima |
| OTU2311 | 2 | 425 | JN606065 | 95.67 | 416 | 98 | d__Eukaryota;k__Alveolata;p__norank;c__Dinophyceae;o__Syndiniales;f__Euduboscquellidae;g__Euduboscquella;s__Euduboscquella_crenulata |
| OTU977 | 2 | 517 | EF616462 | 81.73 | 520 | 100 | d__Eukaryota;k__Alveolata;p__norank;c__Dinophyceae;o__Gymnodiniales;f__Gymnodiniaceae;g__Cochlodinium;s__Cochlodinium_cf._geminatum |
| OTU2665 | 2 | 510 | AB716928 | 90.14 | 507 | 99 | d__Eukaryota;k__Alveolata;p__norank;c__Dinophyceae;o__Peridiniales;f__Protoperidiniaceae;g__Protoperidinium;s__Protoperidinium_monovelum |
| OTU1836 | 2 | 508 | HM483396 | 88.04 | 510 | 100 | d__Eukaryota;k__Alveolata;p__norank;c__Dinophyceae;o__Peridiniales;f__Thoracosphaeraceae;g__Scrippsiella;s__Scrippsiella_trochoidea |
| OTU3733 | 2 | 265 | AB858353 | 86.36 | 132 | 50 | d__Eukaryota;k__Alveolata;p__norank;c__Dinophyceae;o__Suessiales;f__Biecheleriaceae;g__Biecheleria;s__unclassified DINO |
| OTU1822 | 2 | 497 | HM483396 | 93.37 | 498 | 100 | d__Eukaryota;k__Alveolata;p__norank;c__Dinophyceae;o__Peridiniales;f__Thoracosphaeraceae;g__Scrippsiella;s__Scrippsiella_trochoidea |
| OTU1848 | 2 | 500 | HM483396 | 94.19 | 516 | 100 | d__Eukaryota;k__Alveolata;p__norank;c__Dinophyceae;o__Peridiniales;f__Thoracosphaeraceae;g__Scrippsiella;s__Scrippsiella_trochoidea |
| OTU201 | 2 | 227 | LK934662 | 91.15 | 226 | 99 | d__Eukaryota;k__Alveolata;p__norank;c__Dinophyceae;o__Peridiniales;f__Pfiesteriaceae;g__Aduncodinium;s__Aduncodinium_glandula |
| OTU2024 | 2 | 518 | JN119844 | 76.28 | 548 | 99 | d__Eukaryota;k__Alveolata;p__norank;c__Dinophyceae;o__Peridiniales;f__Heterocapsaceae;g__Heterocapsa;s__Heterocapsa_sp._HCBC88 |
| OTU2738 | 2 | 386 | KP702719 | 98.19 | 387 | 100 | d__Eukaryota;k__Alveolata;p__norank;c__Dinophyceae;o__Peridiniales;f__Diplopsaliaceae;g__Oblea;s__Oblea_rotunda |
| OTU3394 | 2 | 510 | AB871535 | 96.86 | 509 | 99 | d__Eukaryota;k__Alveolata;p__norank;c__Dinophyceae;o__Gonyaulacales;f__Crypthecodiniaceae;g__Crypthecodinium;s__Crypthecodinium_sp._ShSu-2013 |
| OTU2488 | 2 | 226 | HQ845329 | 84.58 | 227 | 99 | d__Eukaryota;k__Alveolata;p__norank;c__Dinophyceae;o__Peridiniales;f__Peridiniaceae;g__Pentapharsodinium;s__Pentapharsodinium_tyrrhenicum |

| | | | | | | | |
|---|---|---|---|---|---|---|---|
| OTU676 | 2 | 502 | HQ176320 | 77.53 | 503 | 99 | d__Eukaryota;k__Alveolata;p__norank;c__Dinophyceae;o__Peridiniales;f__Pfiesteriaceae;g__Chimonodinium;s__Chimonodinium_lomnickii |
| OTU954 | 2 | 524 | EF613367 | 98.73 | 474 | 90 | d__Eukaryota;k__Alveolata;p__norank;c__Dinophyceae;o__Gymnodiniales;f__Polykrikaceae;g__Polykrikos;s__Polykrikos_kofoidii |
| OTU40 | 2 | 515 | AF260393 | 96.7 | 515 | 100 | d__Eukaryota;k__Alveolata;p__norank;c__Dinophyceae;o__Peridiniales;f__Thoracosphaeraceae;g__Scrippsiella;s__Scrippsiella_trochoidea |
| OTU3692 | 2 | 317 | KP790210 | 92.86 | 112 | 35 | d__Eukaryota;k__Alveolata;p__norank;c__Dinophyceae;o__Gymnodiniales;f__Gymnodiniaceae;g__Gyrodinium;s__Gyrodinium_sp. |
| OTU1826 | 2 | 493 | HM483396 | 92.12 | 520 | 100 | d__Eukaryota;k__Alveolata;p__norank;c__Dinophyceae;o__Peridiniales;f__Thoracosphaeraceae;g__Scrippsiella;s__Scrippsiella_trochoidea |
| OTU2176 | 2 | 513 | KJ508390 | 92.42 | 66 | 35 | d__Eukaryota;k__Alveolata;p__norank;c__Dinophyceae;o__norank;f__norank;g__norank;s__Unclassified DINO |
| OTU2034 | 2 | 364 | HQ845328 | 79.94 | 359 | 99 | d__Eukaryota;k__Alveolata;p__norank;c__Dinophyceae;o__Peridiniales;f__Thoracosphaeraceae;g__Ensiculifera;s__Ensiculifera_aff._loeblichii |
| OTU45 | 2 | 516 | AF260393 | 97.09 | 516 | 100 | d__Eukaryota;k__Alveolata;p__norank;c__Dinophyceae;o__Peridiniales;f__Thoracosphaeraceae;g__Scrippsiella;s__Scrippsiella_trochoidea |
| OTU1372 | 2 | 229 | FJ024706 | 98.67 | 225 | 98 | d__Eukaryota;k__Alveolata;p__norank;c__Dinophyceae;o__Lophodiniales;f__Lophodiniaceae;g__Woloszynskia;s__Woloszynskia_sp._MB-1 |
| OTU237 | 2 | 470 | AY154960 | 94.89 | 489 | 100 | d__Eukaryota;k__Alveolata;p__norank;c__Dinophyceae;o__Gonyaulacales;f__Gonyaulacaceae;g__Gonyaulax;s__Gonyaulax_cf._spinifera |
| OTU393 | 2 | 490 | FJ024705 | 93.27 | 490 | 100 | d__Eukaryota;k__Alveolata;p__norank;c__Dinophyceae;o__Suessiales;f__Biecheleriaceae;g__Biecheleria;s__Biecheleria_cincta |
| OTU78 | 2 | 497 | AF260393 | 86 | 500 | 100 | d__Eukaryota;k__Alveolata;p__norank;c__Dinophyceae;o__Peridiniales;f__Thoracosphaeraceae;g__Scrippsiella;s__Scrippsiella_trochoidea |
| OTU1645 | 2 | 489 | FN557541 | 93.05 | 489 | 100 | d__Eukaryota;k__Alveolata;p__norank;c__Dinophyceae;o__unclassified_Dinophyceae;f__norank;g__Stoeckeria;s__Stoeckeria_sp._SSMS0806 |
| OTU3423 | 2 | 331 | AB858353 | 96.07 | 331 | 100 | d__Eukaryota;k__Alveolata;p__norank;c__Dinophyceae;o__Suessiales;f__Biecheleriaceae;g__Biecheleria;s__Biecheleria_brevisulcata |
| OTU1117 | 2 | 235 | EF058276 | 83.33 | 234 | 99 | d__Eukaryota;k__Alveolata;p__norank;c__Dinophyceae;o__Lophodiniales;f__Lophodiniaceae;g__Woloszynskia;s__Woloszynskia_pascheri |
| OTU1898 | 2 | 480 | HM483396 | 92.97 | 498 | 100 | d__Eukaryota;k__Alveolata;p__norank;c__Dinophyceae;o__Peridiniales;f__Thoracosphaeraceae;g__Scrippsiella;s__Scrippsiella_trochoidea |
| OTU1910 | 2 | 505 | HM483396 | 89.5 | 505 | 100 | d__Eukaryota;k__Alveolata;p__norank;c__Dinophyceae;o__Peridiniales;f__Thoracosphaeraceae;g__Scrippsiella;s__Scrippsiella_trochoidea |
| OTU1012 | 2 | 478 | AB858353 | 92.48 | 479 | 100 | d__Eukaryota;k__Alveolata;p__norank;c__Dinophyceae;o__Suessiales;f__Biecheleriaceae;g__Biecheleria;s__Biecheleria_brevisulcata |
| OTU2809 | 2 | 221 | KC895475 | 96.38 | 221 | 100 | d__Eukaryota;k__Alveolata;p__norank;c__Dinophyceae;o__Suessiales;f__Biecheleriaceae;g__Biecheleria;s__Biecheleria_cincta |
| OTU3345 | 2 | 474 | KF751924 | 93.26 | 475 | 100 | d__Eukaryota;k__Alveolata;p__norank;c__Dinophyceae;o__Peridiniales;f__Thoracosphaeraceae;g__Scrippsiella;s__Scrippsiella_aff._acuminata |

| | | | | | | | |
|---|---|---|---|---|---|---|---|
| OTU228 | 2 | 483 | AY154960 | 97.49 | 478 | 99 | d__Eukaryota;k__Alveolata;p__norank;c__Dinophyceae;o__Gonyaulacales;f__Gonyaulacaceae;g__Gonyaulax;s__Gonyaulax_cf._spinifera |
| OTU280 | 2 | 477 | AY154965 | 77.62 | 496 | 99 | d__Eukaryota;k__Alveolata;p__norank;c__Dinophyceae;o__Gonyaulacales;f__Gonyaulacaceae;g__Gonyaulax;s__Gonyaulax_sp. |
| OTU3156 | 2 | 400 | KF383297 | 88.89 | 90 | 23 | d__Eukaryota;k__Alveolata;p__norank;c__Dinophyceae;o__Suessiales;f__Symbiodiniaceae;g__Symbiodinium;s__Symbiodinium-like_sp. |
| OTU3708 | 2 | 523 | HM483395 | 73.16 | 529 | 99 | d__Eukaryota;k__Alveolata;p__norank;c__Dinophyceae;o__Syndiniales;f__Amoebophryaceae;g__Amoebophrya;s__Amoebophrya_sp._ex_Akashiwo_sanguineaum |
| OTU774 | 2 | 511 | JF430394 | 83.82 | 513 | 100 | d__Eukaryota;k__Alveolata;p__norank;c__Dinophyceae;o__Peridiniales;f__Pfiesteriaceae;g__Chimonodinium;s__Chimonodinium_sp. |
| OTU2969 | 2 | 313 | KF240778 | 98.06 | 310 | 99 | d__Eukaryota;k__Alveolata;p__norank;c__Dinophyceae;o__Peridiniales;f__Heterocapsaceae;g__Heterocapsa;s__Heterocapsa_rotundata |
| OTU3354 | 2 | 504 | KF751924 | 96.43 | 504 | 100 | d__Eukaryota;k__Alveolata;p__norank;c__Dinophyceae;o__Peridiniales;f__Thoracosphaeraceae;g__Scrippsiella;s__Scrippsiella_aff._acuminata |
| OTU1146 | 2 | 522 | HM483396 | 88.7 | 522 | 100 | d__Eukaryota;k__Alveolata;p__norank;c__Dinophyceae;o__Peridiniales;f__Thoracosphaeraceae;g__Scrippsiella;s__Scrippsiella_trochoidea |
| OTU1456 | 2 | 540 | FJ160592 | 90.12 | 81 | 33 | d__Eukaryota;k__Alveolata;p__norank;c__Dinophyceae;o__norank;f__norank;g__norank;s__Unclassified DINO |
| OTU2031 | 2 | 282 | JN119844 | 82.17 | 258 | 91 | d__Eukaryota;k__Alveolata;p__norank;c__Dinophyceae;o__Peridiniales;f__Heterocapsaceae;g__Heterocapsa;s__Heterocapsa_sp._HCBC88 |
| OTU2656 | 2 | 499 | AB716928 | 90.38 | 499 | 100 | d__Eukaryota;k__Alveolata;p__norank;c__Dinophyceae;o__Peridiniales;f__Protoperidiniaceae;g__Protoperidinium;s__Protoperidinium_monovelum |
| OTU3349 | 2 | 451 | KF751924 | 94.81 | 462 | 100 | d__Eukaryota;k__Alveolata;p__norank;c__Dinophyceae;o__Peridiniales;f__Thoracosphaeraceae;g__Scrippsiella;s__Scrippsiella_aff._acuminata |
| OTU3414 | 2 | 505 | AB858353 | 98.35 | 303 | 66 | d__Eukaryota;k__Alveolata;p__norank;c__Dinophyceae;o__Suessiales;f__Biecheleriaceae;g__Biecheleria;s__Biecheleria-like_sp. |
| OTU1459 | 2 | 505 | FJ167681 | 96.63 | 505 | 100 | d__Eukaryota;k__Alveolata;p__norank;c__Dinophyceae;o__Peridiniales;f__Pfiesteriaceae;g__Tyrannodinium;s__Tyrannodinium_berolinense |
| OTU1886 | 2 | 490 | HM483396 | 94.67 | 488 | 99 | d__Eukaryota;k__Alveolata;p__norank;c__Dinophyceae;o__Peridiniales;f__Thoracosphaeraceae;g__Scrippsiella;s__Scrippsiella_trochoidea |
| OTU2740 | 2 | 484 | JX262498 | 95.66 | 484 | 100 | d__Eukaryota;k__Alveolata;p__norank;c__Dinophyceae;o__Peridiniales;f__Peridiniaceae;g__Pentapharsodinium;s__Pentapharsodinium_dalei |
| OTU1010 | 2 | 472 | EF616463 | 95.35 | 86 | 18 | d__Eukaryota;k__Alveolata;p__norank;c__Dinophyceae;o__norank;f__norank;g__norank;s__Unclassified DINO |
| OTU1088 | 2 | 512 | JN119844 | 75.27 | 558 | 100 | d__Eukaryota;k__Alveolata;p__norank;c__Dinophyceae;o__Peridiniales;f__Heterocapsaceae;g__Heterocapsa;s__Heterocapsa_sp._HCBC88 |
| OTU1876 | 2 | 459 | HM483396 | 95.21 | 459 | 100 | d__Eukaryota;k__Alveolata;p__norank;c__Dinophyceae;o__Peridiniales;f__Thoracosphaeraceae;g__Scrippsiella;s__Scrippsiella_trochoidea |
| OTU2326 | 2 | 544 | JN934988 | 99.26 | 537 | 99 | d__Eukaryota;k__Alveolata;p__norank;c__Dinophyceae;o__Syndiniales;f__Euduboscquellidae;g__Euduboscquella;s__Euduboscquella_cachoni |

| | | | | | | | |
|---|---|---|---|---|---|---|---|
| OTU3357 | 2 | 493 | KF751924 | 93.74 | 495 | 100 | d__Eukaryota;k__Alveolata;p__norank;c__Dinophyceae;o__Peridiniales;f__Thoracosphaeraceae;g__Scrippsiella;s__Scrippsiella_aff._acuminata |
| OTU3358 | 2 | 452 | KF751924 | 94.91 | 452 | 100 | d__Eukaryota;k__Alveolata;p__norank;c__Dinophyceae;o__Peridiniales;f__Thoracosphaeraceae;g__Scrippsiella;s__Scrippsiella_aff._acuminata |
| OTU3376 | 2 | 487 | KF751926 | 94.26 | 488 | 100 | d__Eukaryota;k__Alveolata;p__norank;c__Dinophyceae;o__Peridiniales;f__Thoracosphaeraceae;g__Scrippsiella;s__Scrippsiella_sp. |
| OTU1333 | 2 | 500 | FJ024705 | 92.22 | 501 | 100 | d__Eukaryota;k__Alveolata;p__norank;c__Dinophyceae;o__Suessiales;f__Biecheleriaceae;g__Biecheleria;s__Biecheleria_cincta |
| OTU1364 | 2 | 486 | FJ024705 | 92.59 | 486 | 100 | d__Eukaryota;k__Alveolata;p__norank;c__Dinophyceae;o__Suessiales;f__Biecheleriaceae;g__Biecheleria;s__Biecheleria_cincta |
| OTU1785 | 2 | 527 | HM483396 | 95.98 | 522 | 99 | d__Eukaryota;k__Alveolata;p__norank;c__Dinophyceae;o__Peridiniales;f__Thoracosphaeraceae;g__Scrippsiella;s__Scrippsiella_trochoidea |
| OTU1828 | 2 | 490 | HM483396 | 91.8 | 488 | 99 | d__Eukaryota;k__Alveolata;p__norank;c__Dinophyceae;o__Peridiniales;f__Thoracosphaeraceae;g__Scrippsiella;s__Scrippsiella_trochoidea |
| OTU1867 | 2 | 489 | HM483396 | 95.71 | 489 | 100 | d__Eukaryota;k__Alveolata;p__norank;c__Dinophyceae;o__Peridiniales;f__Thoracosphaeraceae;g__Scrippsiella;s__Scrippsiella_trochoidea |
| OTU2442 | 2 | 496 | JQ413373 | 89.56 | 498 | 100 | d__Eukaryota;k__Alveolata;p__norank;c__Dinophyceae;o__Suessiales;f__Biecheleriaceae;g__Biecheleria;s__Biecheleria_cincta |
| OTU2466 | 2 | 403 | JQ413374 | 96.78 | 404 | 100 | d__Eukaryota;k__Alveolata;p__norank;c__Dinophyceae;o__Suessiales;f__Biecheleriaceae;g__Biecheleria;s__Biecheleria_cincta |
| OTU25 | 2 | 503 | AF260392 | 85.74 | 505 | 100 | d__Eukaryota;k__Alveolata;p__norank;c__Dinophyceae;o__Peridiniales;f__Thoracosphaeraceae;g__Scrippsiella;s__Scrippsiella_sp. |
| OTU2602 | 2 | 298 | KJ189491 | 97.99 | 299 | 100 | d__Eukaryota;k__Alveolata;p__norank;c__Dinophyceae;o__Peridiniales;f__Thoracosphaeraceae;g__Scrippsiella;s__Scrippsiella_cf._acuminata |
| OTU2619 | 2 | 499 | JN982400 | 100 | 499 | 65 | d__Eukaryota;k__Alveolata;p__norank;c__Dinophyceae;o__Peridiniales;f__Thoracosphaeraceae;g__Scrippsiella;s__Scrippsiella_aff._acuminata |
| OTU3346 | 2 | 405 | KF751924 | 96.77 | 402 | 99 | d__Eukaryota;k__Alveolata;p__norank;c__Dinophyceae;o__Peridiniales;f__Thoracosphaeraceae;g__Scrippsiella;s__Scrippsiella_aff._acuminata |
| OTU634 | 2 | 265 | KT389966 | 97 | 246 | 100 | d__Eukaryota;k__Alveolata;p__norank;c__Dinophyceae;o__Peridiniales;f__Peridiniaceae;g__Pentapharsodinium;s__Pentapharsodinium_tyrrhenicum |
| OTU1043 | 2 | 432 | EU048553 | 97.92 | 433 | 100 | d__Eukaryota;k__Alveolata;p__norank;c__Dinophyceae;o__Peridiniales;f__Pfiesteriaceae;g__unclassified_Pfiesteriaceae;s__Pfiesteriaceae_sp._masanensis |
| OTU1178 | 2 | 487 | HQ670228.1 | 98 | 487 | 100 | d__Eukaryota;k__Alveolata;p__norank;c__Dinophyceae;o__Peridiniales;f__Thoracosphaeraceae;g__Scrippsiella;s__Scrippsiella_trochoidea |
| OTU1329 | 2 | 508 | FJ024705 | 95.44 | 504 | 99 | d__Eukaryota;k__Alveolata;p__norank;c__Dinophyceae;o__Suessiales;f__Biecheleriaceae;g__Biecheleria;s__Biecheleria_cincta |
| OTU1346 | 2 | 489 | FJ024705 | 91.85 | 491 | 100 | d__Eukaryota;k__Alveolata;p__norank;c__Dinophyceae;o__Suessiales;f__Biecheleriaceae;g__Biecheleria;s__Biecheleria_cincta |
| OTU1359 | 2 | 492 | FJ024705 | 95.53 | 492 | 100 | d__Eukaryota;k__Alveolata;p__norank;c__Dinophyceae;o__Suessiales;f__Biecheleriaceae;g__Biecheleria;s__Biecheleria_cincta |

| | | | | | | | |
|---|---|---|---|---|---|---|---|
| OTU1361 | 2 | 491 | FJ024705 | 90.84 | 491 | 100 | d__Eukaryota;k__Alveolata;p__norank;c__Dinophyceae;o__Suessiales;f__Biecheleriaceae;g__Biecheleria;s__Biecheleria_cincta |
| OTU1363 | 2 | 488 | FJ024705 | 93.24 | 488 | 100 | d__Eukaryota;k__Alveolata;p__norank;c__Dinophyceae;o__Suessiales;f__Biecheleriaceae;g__Biecheleria;s__Biecheleria_cincta |
| OTU1695 | 2 | 507 | LC002848 | 82.09 | 402 | 79 | d__Eukaryota;k__Alveolata;p__norank;c__Dinophyceae;o__unclassified_Dinophyceae;f__norank;g__norank;s__Unclassified DINO |
| OTU1884 | 2 | 489 | HM483396 | 92.02 | 489 | 100 | d__Eukaryota;k__Alveolata;p__norank;c__Dinophyceae;o__Peridiniales;f__Thoracosphaeraceae;g__Scrippsiella;s__Scrippsiella_trochoidea |
| OTU240 | 2 | 436 | AY154960 | 96.45 | 422 | 97 | d__Eukaryota;k__Alveolata;p__norank;c__Dinophyceae;o__Gonyaulacales;f__Gonyaulacaceae;g__Gonyaulax;s__Gonyaulax_cf._spinifera |
| OTU2407 | 2 | 494 | JQ616825 | 76.41 | 496 | 99 | d__Eukaryota;k__Alveolata;p__norank;c__Dinophyceae;o__Gymnodiniales;f__Gymnodiniaceae;g__Gymnodinium;s__Gymnodinium_catenatum |
| OTU2446 | 2 | 507 | FJ024705 | 89.11 | 505 | 99 | d__Eukaryota;k__Alveolata;p__norank;c__Dinophyceae;o__Suessiales;f__Biecheleriaceae;g__Biecheleria;s__Biecheleria_cincta |
| OTU2450 | 2 | 492 | FJ024705 | 86.87 | 495 | 100 | d__Eukaryota;k__Alveolata;p__norank;c__Dinophyceae;o__Suessiales;f__Biecheleriaceae;g__Biecheleria;s__Biecheleria_cincta |
| OTU2467 | 2 | 492 | FJ024705 | 86.49 | 496 | 100 | d__Eukaryota;k__Alveolata;p__norank;c__Dinophyceae;o__Suessiales;f__Biecheleriaceae;g__Biecheleria;s__Biecheleria_cincta |
| OTU2965 | 2 | 257 | KF240778 | 96.96 | 263 | 100 | d__Eukaryota;k__Alveolata;p__norank;c__Dinophyceae;o__Peridiniales;f__Heterocapsaceae;g__Heterocapsa;s__Heterocapsa_rotundata |
| OTU3406 | 2 | 515 | AB858353 | 94.69 | 508 | 99 | d__Eukaryota;k__Alveolata;p__norank;c__Dinophyceae;o__Suessiales;f__Biecheleriaceae;g__Biecheleria;s__Biecheleria_brevisulcata |
| OTU3424 | 2 | 488 | AB858353 | 95.09 | 489 | 100 | d__Eukaryota;k__Alveolata;p__norank;c__Dinophyceae;o__Suessiales;f__Biecheleriaceae;g__Biecheleria;s__Biecheleria_brevisulcata |
| OTU379 | 2 | 492 | AY916546 | 95.9 | 488 | 99 | d__Eukaryota;k__Alveolata;p__norank;c__Dinophyceae;o__Peridiniales;f__Thoracosphaeraceae;g__Scrippsiella;s__Scrippsiella_sp._CS297 |
| OTU382 | 2 | 498 | AY916546 | 96.75 | 492 | 99 | d__Eukaryota;k__Alveolata;p__norank;c__Dinophyceae;o__Peridiniales;f__Thoracosphaeraceae;g__Scrippsiella;s__Scrippsiella_sp._CS297 |

**Table S7.** Numbers of dinoflagellate taxa reported in ships' ballast tanks, including the present study, and the studies' method(s) of identification.

| References | Number of dinoflagellates identified to species level (n) | Number of dinoflagellates not identified to species level (n) | Total number of the reported dinoflagellate taxa (n) | Identification method | Sample type |
|---|---|---|---|---|---|
| This study | 73 | 545 OTUs | | Microscopy/Single-cell PCR/NGS | BS |
| Casas-Monroy et al., 2011 [1] | 32 | 26 | 58 | Microscopy/Culture | BS |
| Casas-Monroy et al., 2013 [2] | 39 | 21 | 60 | Microscopy/Culture | BS |
| Fahnenstiel et al., 2009 [3] | 17 | 18 | 35 | Microscopy | BS |
| Garrett et al., 2011 [4] | 10 | 1 | 11 | Microscopy/Culture | BS |
| Garrett et al., 2014 [5] | 1 | 0 | 1 | Microscopy/Culture/PCR | BS |
| Gollasch et al., 2000a [6] | Unknown | Unknown | Unknown | Unknown | BS |
| Hallegraeff and Bolch, 1992 [7] | 33 | 20 | 53 | Microscopy/Culture | BS |
| Hamer et al., 2000 [8] | 17 | 14 | 31 | Microscopy | BS |
| Hamer et al., 2001 [9] | 42 | 12 | 54 | Microscopy/Culture | BS |
| Harvey et al., 1999 [10] | 14 | 9 | 23 | Microscopy/Culture | BS |
| Johengen et al., 2005 [11] | 32 | 1 | 33 | Microscopy/Culture | BS |
| Macdonald, 1995 [12] | 23 | 10 | 33 | Microscopy | BS |
| Pertola et al., 2006 [13] | 4 | 9 | 13 | Microscopy/Culture | BS |
| Shaw et al. 2019 [14] | 0 | 35 OTUs | | Microscopy/NGS | BS |

| | | | | | |
|---|---|---|---|---|---|
| Cabrini et al. 2018 [15] | 25 | 8 | 33 | Microscopy | BW |
| Cheniti et al. 2018 [16] | 32 | 9 | 41 | Microscopy | BW |
| Baek et al., 2012 [17] | 3 | 0 | 3 | Microscopy/Culture | BW |
| Boltovskoy et al., 2011 [18] | 50 | 17 | 67 | Microscopy | BW |
| Briski et al., 2015 [19] | 14 | 3 | 17 | Microscopy | BW |
| Burkholder et al., 2007 [20] | 32 | 0 | 32 | Microscopy/Culture/PCR | BW |
| Butron et al., 2011 [21] | 20 | 9 | 29 | Microscopy/Culture | BW |
| Carlton and Geller, 1993 [22] | | 4 | 4 | Unknown | BW |
| Casas-Monroy et al., 2016 [23] | 142 | 13 | 155 | Microscopy/Culture | BW |
| David et al., 2007 [24] | 9 | 14 | 23 | Microscopy/Culture | BW |
| DiBacco et al., 2012 [25] | 1 | | 1 | Microscopy | BW |
| Drake et al., 2005 [26] | 1 | 0 | 1 | Culture/PCR | BW |
| Galil and Hulsmann, 1997 [27] | 1 | 4 | 5 | Microscopy | BW |
| Garrett et al., 2011 [4] | 23 | 5 | 28 | Microscopy/Culture | BW |
| Gollasch et al., 2000a [6] | Unknown | Unknown | Unknown | Unknown | BW |
| Gollasch et al., 2000b [28] | | 9 | 9 | Microscopy | BW |
| Harvey et al., 1999 [10] | 45 | 4 | 49 | Microscopy | BW |
| Hyun et al., 2016 [29] | 21 | 3 | 24 | Microscopy/Culture | BW |
| Lavoie et al., 1999 [30] | | 1 | 1 | Microscopy | BW |
| Levings et al., 2004 [31] | | 2 | 2 | Microscopy | BW |
| Lewis et al., 2003 [32] | 1 | 1 | 2 | Microscopy | BW |
| Masson et al., 2013 [33] | 11 | 3 | 14 | Microscopy | BW |

| | | | | | |
|---|---|---|---|---|---|
| Olenin et al., 2000 [34] | 6 | 0 | 6 | Microscopy | BW |
| Park et al., 2007 [35] | 1 | | 1 | Culture/Molecular identification | BW |
| Rao et al., 1994 [36] | 25 | 5 | 30 | Microscopy | BW |
| Roy et al., 2012 [37] | 114 | 45 | 159 | Microscopy | BW |
| Selifonova, 2009 [38] | 1 | | 1 | Unknown | BW |
| Stat and Gates, 2008 [39] | | 1 | 1 | PCR | BW |
| Steichen et al., 2014 [40] | 4 | 4 | 8 | DGGE | BW |
| Steichen et al., 2015 [41] | 2 | 6 | 8 | Culture/PCR | BW |
| Steichen and Quigg, 2015 [42] | | 10 | 10 | Microscopy | BW |
| Wonham et al., 2001 [43] | | 3 | 3 | Microscopy | BW |
| Zaiko et al., 2015 [44] | | 5 | 5 | NGS | BW |
| Zhang and Dickman, 1999 [45] | 8 | 1 | 9 | Microscopy | BW |
| Zmerli and Yahia-Kefi, 2012 [46] | 3 | 1 | 4 | Microscopy | BW |
| Zvyagintsev et al., 2009 [47] | 11 | 3 | 14 | Microscopy | BW |
| Zvyagintsev and Selifonova, 2010 [48] | 12 | 3 | 15 | Unknown | BW |

OTUs = Operational Taxonomic Units; NGS = Next Generation Sequencing; BS = Ballast Tank Sediment Samples; BW = Ballast Water Samples; PCR = Polymerase Chain Reaction; DGGE = Denaturing Gradient Gel Electrophoresis.

References:

1. Casas-Monroy, O.; Roy, S.; Rochon, A. Ballast sediment-mediated transport of non-indigenous species of dinoflagellates on the East Coast of Canada. *Aquatic Invasions* **2011**, *6*, 231-248, doi:10.3391/ai.2011.6.3.01.
2. Casas-Monroy, O.; Roy, S.; Rochon, A. Dinoflagellate cysts in ballast sediments: differences between Canada's east coast, west coast and the Great Lakes. *Aquat. Conserv. Mar. Freshwater Ecosyst.* **2013**, *23*, 254-276, doi:10.1002/aqc.2310.
3. Fahnenstiel, G.; Hong, Y.; Millie, D.; Doblin, M.; Johengen, T.; Reid, D. Marine dinoflagellate cysts in the ballast tank sediments of ships entering the Laurentian Great Lakes. *International Association of Theoretical and Applied Limnology* **2009**, *30*, 1035-1038, doi:10.2307/4563729.
4. Garrett, M.J.; Wolny, J.L.; Williams, B.J.; Dirks, M.D.; Brame, J.A.; Richardson, R.W. Methods for sampling and analysis of marine microalgae in ship ballast tanks: a case study from Tampa Bay, Florida, USA. *Algae* **2011**, *26*, 181-192.
5. Garrett, M.J.; Puchulutegui, C.; Selwood, A.I.; Wolny, J.L. Identification of the harmful dinoflagellate *Vulcanodinium rugosum* recovered from a ballast tank of a globally traveled

ship in Port Tampa Bay, Florida, USA. *Harmful Algae* **2014**, *39*, 202-209, doi:10.1016/j.hal.2014.07.014.

6. Gollasch, S.; Dammer, M.; Lenz, J.; Andres, H.G. Non-indigenous organisms introduced via ships into German waters. *Canadian journal of anaesthesia = Journal canadien d'anesthésie* **2000**, *47*, 716-717.
7. Hallegraeff, G.M.; Bolch, C.J. Transport of diatom and dinoflagellate resting spores in ships' ballast water: implications for plankton biogeography and aquaculture. *J. Plankton Res.* **1992**, *14*, 1067-1084, doi:10.1093/plankt/14.8.1067.
8. Hamer, J.P.; McCollin, T.A.; Lucas, I.A.N. Dinoflagellate cysts in ballast tank sediments: between tank variability. *Mar. Pollut. Bull.* **2000**, *40*, 731-733, doi:http://dx.doi.org/10.1016/S0025-326X(99)00198-8.
9. Hamer, J.P.; Lucas, I.A.N.; McCollin, T.A. Harmful dinoflagellate resting cysts in ships' ballast tank sediments: potential for introduction into English and Welsh waters. *Phycologia* **2001**, *40*, 246-255, doi:10.2216/i0031-8884-40-3-246.1.
10. Harvey, M.; Gilbert, M.; Gauthierl, D.; Reid, D.M. A preliminary assessment of risks for the ballast water-mediated introduction of nonindigenous marine organisms in the Estuary and Gulf of St. Lawrence. *Journal of Geo-Information Science* **1999**, *12*, 89-94.
11. Johengen, T.H.; Reid, D.; Fahnenstiel, G.; MacIsaac, H.; Dobbs, F.; Doblin, M.; Ruiz, G.; Jenkins, P. *Assessment of transoceanic NOBOB vessels and low-salinity ballast water as vectors for nonindigenous species introductions to the Great Lakes*; University of Michigan and NOAA-Great Lakes Environmental Research Laboratory: Ann Arbor, MI, 2005; pp 3-1–3-53.
12. Macdonald, E.M. *Dinoflagellate resting cysts and ballast water discharges in Scottish ports*; Aalborg, Denmark, 1995.
13. Pertola, S.; Faust, M.A.; Kuosa, H. Survey on germination and species composition of dinoflagellates from ballast tanks and recent sediments in ports on the South Coast of Finland, North-Eastern Baltic Sea. *Mar. Pollut. Bull.* **2006**, *52*, 900-911, doi:http://dx.doi.org/10.1016/j.marpolbul.2005.11.028.
14. Shaw, J.L.A.; Weyrich, L.S.; Hallegraeff, G.; Cooper, A. Retrospective eDNA assessment of potentially harmful algae in historical ship ballast tank and marine port sediments. *Mol. Ecol.* **2019**, doi: 10.1111/mec.15055, doi:doi: 10.1111/mec.15055.
15. Cabrini, M.; Cerino, F.; de Olazabal, A.; Di Poi, E.; Fabbro, C.; Fornasaro, D.; Goruppi, A.; Flander-Putrle, V.; France, J.; Gollasch, S., et al. Potential transfer of aquatic organisms via ballast water with a particular focus on harmful and non-indigenous species: A survey from Adriatic ports. *Mar. Pollut. Bull.* **2018**, *14*, 331-338, doi:10.1016/j.marpolbul.2018.02.004.
16. Cheniti, R.; Rochon, A.; Frihi, H. Ship traffic and the introduction of diatoms and dinoflagellates via ballast water in the port of Annaba, Algeria. *J. Sea Res.* **2018**, *133*, 154-165, doi:10.1016/j.seares.2017.07.008.
17. Baek, S.H.; Jung, S.W.; Jang, M.C.; Hyun, B.; Shin, K. Survival potential of autotrophic phytoplankton species collected from ballast water in international commercial ships. *N. Z. J. Mar. Freshwater Res.* **2012**, *46*, 125-136, doi:10.1080/00288330.2011.610326.
18. Boltovskoy, D.; Almada, P.; Correa, N. Biological invasions: assessment of threat from ballast-water discharge in Patagonian (Argentina) ports. *Environ. Sci. Policy* **2011**, *14*, 578-583, doi:10.1016/j.envsci.2011.03.007.

19. Briski, E.; Gollasch, S.; David, M.; Linley, R.D.; Casas-Monroy, O.; Rajakaruna, H.; Bailey, S.A. Combining ballast water exchange and treatment to maximize prevention of species introductions to freshwater ecosystems. *Environ. Sci. Technol.* **2015**, *49*, 9566-9573, doi:10.1021/acs.est.5b01795.
20. Burkholder, J.M.; Hallegraeff, G.M.; Melia, G.; Cohen, A.; Bowers, H.A.; Oldach, D.W.; Parrow, M.W.; Sullivan, M.J.; Zimba, P.V.; Allen, E.H., et al. Phytoplankton and bacterial assemblages in ballast water of US military ships as a function of port of origin, voyage time, and ocean exchange practices. *Harmful Algae* **2007**, *6*, 486-518, doi:10.1016/j.hal.2006.11.006.
21. Butron, A.; Orive, E.; Madariaga, I. Potential risk of harmful algae transport by ballast waters: The case of Bilbao Harbour. *Mar. Pollut. Bull.* **2011**, *62*, 747-757, doi:10.1016/j.marpolbul.2011.01.008.
22. Carlton, J.T.; Geller, J.B. Ecological roulette: the global transport of nonindigenous marine organisms. *Science* **1993**, *261*, 78-82.
23. Casas-Monroy, O.; Parenteau, M.; Drake, D.A.R.; Roy, S.; Rochon, A. Absolute estimates of the propagule pressure of viable dinoflagellates across Canadian coasts: the variable influence of ballast water exchange XXX. *Mar. Biol.* **2016**, *163*, doi:10.1007/s00227-016-2946-3.
24. David, M.; Gollasch, S.; Cabrini, M.; Perkovic, M.; Bosnjak, D.; Virgilio, D. Results from the first ballast water sampling study in the Mediterranean Sea - the Port of Koper study. *Mar. Pollut. Bull.* **2007**, *54*, 53-65, doi:10.1016/j.marpolbul.2006.08.041.
25. DiBacco, C.; Humphrey, D.B.; Nasmith, L.E.; Levings, C.D. Ballast water transport of non-indigenous zooplankton to Canadian ports. *ICES J. Mar. Sci.* **2012**, *69*, 483-491, doi:10.1093/icesjms/fsr133.
26. Drake, L.A.; Meyer, A.E.; Forsberg, R.L.; Baier, R.E.; Doblin, M.A.; Heinemann, S.; Johnson, W.P.; Koch, M.; Rublee, P.A.; Dobbs, F.C. Potential invasion of microorganisms and pathogens via 'interior hull fouling': biofilms inside ballast water tanks. *Biological Invasions* **2005**, *7*, 969-982, doi:10.1007/s10530-004-3001-8.
27. Galil, B.S.; Hulsmann, N. Protist transport via ballast water - Biological classification of ballast tanks by food web interactions. *European Journal of Protistology* **1997**, *33*, 244-253.
28. Gollasch, S.; Lenz, J.; Dammer, M.; Andres, H.-G. Survival of tropical ballast water organisms during a cruise from the Indian Ocean to the North Sea. *J. Plankton Res.* **2000**, *22*, 923-937, doi:10.1093/plankt/22.5.923.
29. Hyun, B.; Shin, K.; Jang, M.-C.; Jang, P.-G.; Lee, W.-J.; Park, C.; Choi, K.-H. Potential invasions of phytoplankton in ship ballast water at South Korean ports. *Mar. Freshwater Res.* **2016**, *67*, 1906–1917, doi:http://dx.doi.org/10.1071/MF15170_AC.
30. Lavoie, D.M.; Smith, L.D.; Ruiz, G.M. The potential for intracoastal transfer of non-indigenous species in the ballast water of ships. *Estuarine Coastal and Shelf Science* **1999**, *48*, 551-564, doi:10.1006/ecss.1999.0467.
31. Levings, C.D.; Cordell, J.R.; Ong, S.; Piercey, G. The origin and identity of invertebrate organisms being transported to Canada's Pacific coast by ballast water. *Can. J. Fish. Aquat.Sci.* **2004**, *61*, 1-11, doi:10.1139/f03-135.
32. Lewis, P.N.; Hewitt, C.L.; Riddle, M.; McMinn, A. Marine introductions in the Southern Ocean: an unrecognised hazard to biodiversity. *Mar. Pollut. Bull.* **2003**, *46*, 213-223, doi:10.1016/s0025-326x(02)00364-8.

33. Masson, D.; Thomas, G.; Genauzeau, S.; Le Moine, O.; Derrien, A. Merchant ships discharging unwanted marine species in close proximity of a French aquaculture area: Risks involved. *Mar. Pollut. Bull.* **2013**, *77*, 315-319, doi:10.1016/j.marpolbul.2013.09.028.
34. Olenin, S.; Gollasch, S.; Jonusas, S.; Rimkute, I. En-route investigations of plankton in ballast water on a ship's voyage from the Baltic Sea to the open Atlantic coast of Europe. *Int. Rev. Hydrobiol.* **2000**, *85*, 577-596, doi:10.1002/1522-2632(200011)85:5/6<577::aid-iroh577>3.0.co;2-c.
35. Park, T.-G.; Bolch, C.J.S.; Hallegraeff, G.M. Morphological and molecular genetic characterization of Cryptoperidiniopsis brodyi (Dinophyceae) from Australia-wide isolates. *Harmful Algae* **2007**, *6*, 718-733, doi:http://dx.doi.org/10.1016/j.hal.2007.02.004.
36. Rao, D.V.S.; Sprules, W.G.; Locke, A.; Carlton, J.T. Exotic phytoplankton from ships' ballast waters: Risk of potential spread to mariculture sites on Canada's East Coast. **1994**.
37. Roy, S.; Parenteau, M.; Casas-Monroy, O.; Rochon, A. Coastal ship traffic: a significant introduction vector for potentially harmful dinoflagellates in eastern Canada. *Can. J. Fish. Aquat.Sci.* **2012**, *69*, 627-644, doi:10.1139/f2012-008.
38. Selifonova, Z.P. Marine biological invasions in waters of the port of Novorossiysk in the Black Sea. *Russ. J. Mar. Biol.* **2009**, *35*, 242-249, doi:10.1134/s1063074009030080.
39. Stat, M.; Gates, R.D. Vectored introductions of marine endosymbiotic dinoflagellates into Hawaii. *Biological Invasions* **2008**, *10*, 579-583, doi:10.1007/s10530-007-9167-0.
40. Steichen, J.L.; Schulze, A.; Brinkmeyer, R.; Quigg, A. All aboard! A biological survey of ballast water onboard vessels spanning the North Atlantic Ocean. *Mar. Pollut. Bull.* **2014**, *87*, 201-210, doi:10.1016/j.marpolbul.2014.07.058.
41. Steichen, J.L.; Denby, A.; Windham, R.; Brinkmeyer, R.; Quigg, A. A tale of two ports: dinoflagellate and diatom communities found in the high ship traffic region of Galveston Bay, Texas (USA). *Journal of Coastal Research* **2015**, *31*, 407-416, doi:10.2112/jcoastres-d-13-00225.1.
42. Steichen, J.L.; Quigg, A. Assessing the viability of microorganisms in the ballast water of vessels transiting the North Atlantic Ocean. *Mar. Pollut. Bull.* **2015**, *101*, 258-266, doi:10.1016/j.marpolbul.2015.09.055.
43. Wonham, M.J.; Walton, W.C.; Ruiz, G.M.; Frese, A.M.; Galil, B.S. Going to the source: role of the invasion pathway in determining potential invaders. *Mar. Ecol. Prog. Ser.* **2001**, *215*, 1-12, doi:10.3354/meps215001.
44. Zaiko, A.; Martinez, J.L.; Schmidt-Petersen, J.; Ribicic, D.; Samuiloviene, A.; Garcia-Vazquez, E. Metabarcoding approach for the ballast water surveillance - An advantageous solution or an awkward challenge? *Mar. Pollut. Bull.* **2015**, *92*, 25-34, doi:10.1016/j.marpolbul.2015.01.008.
45. Zhang, F.Z.; Dickman, M. Mid-ocean exchange of container vessel ballast water. 1: Seasonal factors affecting the transport of harmful diatoms and dinoflagellates. *Marine Ecology Progress* **1999**, *176*, 243-251.
46. Zmerli, H.T.; Yahia-Kefi, O.D. Have non-indigenous planktonic species been introduced via ballast waters in two North African ports (la Goulette and Bizerte, Tunisia)? *Vie Et Milieu-Life and Environment* **2012**, *62*, 1-9.
47. Zvyagintsev, A.Y.; Ivin, V.V.; Kashin, I.A.; Orlova, T.Y.; Selina, M.S.; Kasyan, V.V.; Korn, O.M.; Kornienko, E.S.; Kulikova, V.A.; Bezverbnaya, I.P., et al. Acclimation and introduction of hydrobionts ships' ballast water organisms in the Port of Vladivostok. *Russ. J. Mar. Biol.* **2009**, *35*, 41-52, doi:10.1134/s1063074009010076.

48. Zvyagintsev, A.Y.; Selifonova, J.P. Hydrobiological studies of the ballast waters of cargo ships in Russian Sea ports. *Oceanology* **2010**, *50*, 924-932, doi:10.1134/s0001437010060123.

**Table S8.** Dinoflagellate species reported in ballast tank sediments. Species denoted with paleontological names were not included.

| No. | Species | Sampling Time | Sampling Port | References |
|---|---|---|---|---|
| **1** | ***Alexandrium affine*** | **2000–2003** | **Great Lakes** | **This study** |
| | | 2002–2003 | Great Lakes | Reported as *Alexandrium affinis* [1] |
| | | 2001–2002 | Great Lakes | Reported as *Alexandrium affinis* [2] |
| 2 | *Alexandrium balechii* | 2003–2006 | Port of Tampa and Port Manatee | [3] |
| **3** | ***Alexandrium fundyense**** | **2000–2003** | **Great Lakes** | **This study** |
| 4 | *Alexandrium hiranoi* | 2002–2003 | Great Lakes | [1] |
| | | 2001–2002 | Great Lakes | [2] |
| 5 | *Alexandrium leei* | 2001 (published) | English and Welsh ports | [4] |
| 6 | *Alexandrium margalefi* | 1994 | Scottish ports | [5] |
| 7 | *Alexandrium minutum* | 2001–2002 | Great Lakes | Reported as *Alexandrium lusitanicum* [2] |
| | | 2002–2003 | Great Lakes | Reported as *Alexandrium lusitanicum* [1] |
| | | 2002–2003 | Great Lakes | [1] |
| | | 2001–2002 | Great Lakes | [2] |
| | | 1994 | Scottish ports | [5] |
| | | 2001 (published) | English and Welsh ports | [4] |
| **8** | ***Alexandrium ostenfeldii**** | **2000–2003** | **Great Lakes** | **This study** |
| **9** | ***Alexandrium pacificum*** | **2000–2003** | **Great Lakes** | **This study** |
| | | 1987–1990 | Australian ports | Reported as *Alexandrium catenella* [6] |
| | | 2001–2002 | Great Lakes | Reported as *Alexandrium catenella* [2] |
| **10** | ***Alexandrium peruvianum**** | **2000–2003** | **Great Lakes** | **This study** |
| **11** | ***Alexandrium pseudogonyaulax*** | **2000–2003** | **Great Lakes** | **This study** |
| | | 2001 (published) | English and Welsh ports | [4] |
| 12 | *Alexandrium tamarense* | 2007–2009 | Canada's east coast, west coast and the Great Lakes | [7] |
| | | 1987–1990 | Australian ports | [6] |
| | | 2000 (published) | English and Welsh ports | [8] |
| | | 2002–2003 | Great Lakes | [1] |
| | | 2007–2009 | East coast of Canada | [9] |

| | | | | |
|---|---|---|---|---|
| | | 2001 (published) | English and Welsh ports | [4] |
| | | 2001–2002 | Great Lakes | [2] |
| 13 | *Alexandrium tropicale* | 2001 (published) | English and Welsh ports | [4] |
| **14** | ***Apocalathium aciculiferum**** | **2000–2003** | **Great Lakes** | **This study** |
| **15** | ***Apocalathium baicalense**** | **2000–2003** | **Great Lakes** | **This study** |
| **16** | ***Apocalathium euryceps**** | **2000–2003** | **Great Lakes** | **This study** |
| **17** | ***Apocalathium malmogiense*** | **2000–2003** | **Great Lakes** | **This study** |
| | | 2000 (published) | England and Wales | Reported as *Scrippsiella hangoei* [8] |
| | | 2001 (published) | English and Welsh ports | Reported as *Scrippsiella hangoei* [4] |
| 18 | *Archaeperidinium minutum* | 2007–2009 | East coast of Canada | Reported as *Protoperidinium minutum* [9] |
| **19** | ***Archaeperidinium saanichi**** | **2000–2003** | **Great Lakes** | **This study** |
| **20** | ***Azadinium polongum**** | **2000–2003** | **Great Lakes** | **This study** |
| **21** | ***Azadinium poporum**** | **2000–2003** | **Great Lakes** | **This study** |
| **22** | ***Baldinia anauniensis**** | **2000–2003** | **Great Lakes** | **This study** |
| **23** | ***Barrufeta bravensis**** | **2000–2003** | **Great Lakes** | **This study** |
| **24** | ***Biecheleria baltica**** | **2000–2003** | **Great Lakes and the Chesapeake Bay** | **This study** |
| **25** | ***Biecheleria brevisulcata**** | **2000–2003** | **Great Lakes and the Chesapeake Bay** | **This study** |
| **26** | ***Biecheleria cincta**** | **2000–2003** | **Great Lakes and the Chesapeake Bay** | **This study** |
| **27** | ***Biecheleriopsis adriatica**** | **2000–2003** | **Great Lakes** | **This study** |
| **28** | ***Blastodinium contortum**** | **2000–2003** | **Great Lakes and the Chesapeake Bay** | **This study** |
| **29** | ***Borghiella dodgei**** | **2000–2003** | **Great Lakes** | **This study** |
| **30** | ***Borghiella tenuissima**** | **2000–2003** | **Great Lakes and the Chesapeake Bay** | **This study** |
| 31 | *Calciodinellum levantinum* | 2003–2006 | Port of Tampa and Port Manatee | [3] |
| **32** | ***Chimonodinium lomnickii**** | **2000–2003** | **Great Lakes** | **This study** |
| **33** | ***Cryptoperidiniopsis brodyi**** | **2000–2003** | **Great Lakes and the Chesapeake Bay** | **This study** |
| 34 | *Dinophysis caudata* | 1995 | Estuary and Gulf of St. Lawrence | Reported as *Dinophysis diegensis* [10] |
| **35** | ***Dinophysis lativelata**** | **2000–2003** | **Great Lakes and the Chesapeake Bay** | **This study** |
| 36 | *Dinophysis norvegica* | 1995 | Estuary and Gulf of St. Lawrence | [10] |
| 37 | *Diplopelta parva* | 1987–1990 | Australian ports | [6] |
| | | 1994 | Scottish ports | [5] |

| | | | | |
|---|---|---|---|---|
| | | 2001 (published) | English and Welsh ports | [4] |
| | | 2001–2002 | Great Lakes | [2] |
| 38 | *Diplopsalis lenticula* | 1987–1990 | Australian ports | [6] |
| | | 2001 (published) | English and Welsh ports | [4] |
| 39 | *Diplopsalopsis bomba* | 2001 (published) | English and Welsh ports | Reported as *Diplopelta symmetrica* [4] |
| 40 | *Diplopsalopsis orbicularis* | 2007–2009 | Canada's east coast, west coast and the Great Lakes | Reported as *Diplopsalis orbicularis* [7] |
| | | 1987–1990 | Australian ports | Reported as *Diplopsalopsis orbicularis* [6] |
| **41** | ***Dissodinium pseudolunula*** | **2000–2003** | **Great Lakes** | **This study** |
| | | 2000 (published) | English and Welsh ports | [8] |
| **42** | ***Duboscquodinium collinii**** | **2000–2003** | **Great Lakes** | **This study** |
| **43** | ***Euduboscquella cachoni**** | **2000–2003** | **Great Lakes** | **This study** |
| **44** | ***Euduboscquella crenulata**** | **2000–2003** | **Great Lakes and the Chesapeake Bay** | **This study** |
| 45 | *Fragilidium mexicanum* | 2007–2009 | Canada's east coast, west coast and the Great Lakes | [7] |
| | | 2007–2009 | East coast of Canada | [9] |
| 46 | *Gonyaulax digitalis* | 2007–2009 | East coast of Canada | [9] |
| | | 2007–2009 | Canada's east coast, west coast and the Great Lakes | [7] |
| | | 1987–1990 | Australian ports | Reported as *Gonyaulax digitale* [6] |
| 47 | *Gonyaulax elongata* | 2007–2009 | Canada's east coast, west coast and the Great Lakes | [7] |
| | | 2007–2009 | East coast of Canada | [9] |
| 48 | *Gonyaulax membranacea* | 2007–2009 | Canada's east coast, west coast and the Great Lakes | [7] |
| | | 2007–2009 | East coast of Canada | [9] |
| **49** | ***Gonyaulax polygramma**** | **2000–2003** | **Great Lakes** | **This study** |
| 50 | *Gonyaulax scrippsae* | 2007–2009 | Canada's east coast, west coast and the Great Lakes | [7] |

| | | | | |
|---|---|---|---|---|
| | | 1987–1990 | Australian ports | [6] |
| | | 2007–2009 | East coast of Canada | [9] |
| | | 2002–2003 | Great Lakes | [1] |
| | | 2001–2002 | Great Lakes | [2] |
| | | 2001 (published) | English and Welsh ports | [4] |
| | | 1995 | Estuary and Gulf of St. Lawrence | [10] |
| **51** | ***Gonyaulax spinifera*** | **2000–2003** | **Great Lakes and the Chesapeake Bay** | **This study** |
| | | 2007–2009 | Canada's east coast, west coast and the Great Lakes | Reported as *Gonyaulax spinifera* complex [7] |
| | | 2007–2009 | East coast of Canada | Reported as *Gonyaulax spinifera* complex [9] |
| | | 1987–1990 | Australian ports | [6] |
| | | 2001–2002 | Great Lakes | [2] |
| | | 2001 (published) | English and Welsh ports | [4] |
| | | 2003–2006 | Port of Tampa and Port Manatee | [3] |
| | | 2002–2003 | Great Lakes | [1] |
| | | 1994 | Scottish ports | Reported as *Spiniferities bentori*, *Spiniferities bulloides*, *Spiniferities mirabilis* [5] |
| 52 | *Gonyaulax verior* | 2002–2003 | Great Lakes | [1] |
| | | 2001 (published) | English and Welsh ports | [4] |
| | | 2000 (published) | English and Welsh ports | [8] |
| | | 2001–2002 | Great Lakes | [2] |
| **53** | ***Gymnodinium catenatum*** | **2000–2003** | **Great Lakes** | **This study** |
| | | 2007–2009 | Canada's east coast, west coast and the Great Lakes | [7] |
| | | 2002–2003 | Great Lakes | [1] |
| | | 2001–2002 | Great Lakes | [2] |
| | | 2007–2009 | East coast of Canada | [9] |
| | | 1994 | Scottish ports | [5] |
| **54** | ***Gymnodinium impudicum*** | **2000–2003** | **Great Lakes** | **This study** |
| | | 2007–2009 | Canada's east coast, west coast and the Great Lakes | [7] |

| | | | | |
|---|---|---|---|---|
| **55** | ***Gymnodinium microreticulatum**** | **2000–2003** | **Great Lakes** | **This study** |
| 56 | *Gymnodinium nolleri* | 2007–2009 | Canada's east coast, west coast and the Great Lakes | [7] |
| | | 2001 (published) | English and Welsh ports | [4] |
| **57** | ***Gymnodinium simplex**** | **2000–2003** | **Great Lakes** | **This study** |
| **58** | ***Gyrodiniellum shiwhaense**** | **2000–2003** | **Great Lakes and the Chesapeake Bay** | **This study** |
| **59** | ***Gyrodinium dominans**** | **2000–2003** | **Chesapeake Bay** | **This study** |
| **60** | ***Gyrodinium heterogrammum**** | **2000–2003** | **Great Lakes and the Chesapeake Bay** | **This study** |
| **61** | ***Gyrodinium rubrum**** | **2000–2003** | **Great Lakes and the Chesapeake Bay** | **This study** |
| **62** | ***Gyrodinium spirale*** | **2000–2003** | **Great Lakes** | **This study** |
| | | 2003–2006 | Port of Tampa and Port Manatee | [3] |
| **63** | ***Gyrodinium undulans**** | **2000–2003** | **Great Lakes** | **This study** |
| **64** | ***Heterocapsa minima**** | **2000–2003** | **Great Lakes** | **This study** |
| **65** | ***Heterocapsa rotundata*** | **2000–2003** | **Great Lakes** | **This study** |
| | | Aug–2003 | Finland | [11] |
| | | 2001 (published) | English and Welsh ports | Reported as *Katodinium rotundatum* [4] |
| **66** | ***Heterocapsa triquetra*** | **2000–2003** | **Great Lakes** | **This study** |
| | | 1995 | Estuary and Gulf of St. Lawrence | [10] |
| | | 2001 (published) | English and Welsh ports | [4] |
| **67** | ***Islandinium tricingulatum**** | **2000–2003** | **Great Lakes and the Chesapeake Bay** | **This study** |
| **68** | ***Karenia cristata**** | **2000–2003** | **Great Lakes** | **This study** |
| **69** | ***Karenia papilionacea**** | **2000–2003** | **Great Lakes** | **This study** |
| **70** | ***Karlodinium antarcticum**** | **2000–2003** | **Great Lakes** | **This study** |
| 71 | *Kryptoperidinium foliaceum* | Aug–2003 | Finland | [11] |
| **72** | ***Levanderina fissa**** | **2000–2003** | **Great Lakes and the Chesapeake Bay** | **This study** |
| 73 | *Lingulodinium polyedra* | 2001–2002 | Great Lakes | Reported as *Gonyaulax polyedra* [2] |
| | | 2002–2003 | Great Lakes | Reported as *Gonyaula polydra* [1] |
| | | 1994 | Scottish ports | Reported as *Lingulodinium machaerophorum* [5] |
| | | 1987–1990 | Australian ports | [6] |
| | | 2001 (published) | English and Welsh ports | [4] |
| 74 | *Lingulodinium polyedrum* | 2007–2009 | East coast of Canada | [9] |

| | | | | |
|---|---|---|---|---|
| | | 2007–2009 | Canada's east coast, west coast and the Great Lakes | [7] |
| | | 2003–2006 | Port of Tampa and Port Manatee | [3] |
| **75** | ***Margalefidinium fulvescens**** | **2000–2003** | **Great Lakes** | **This study** |
| **76** | ***Margalefidinium polykrikoides*** | **2000–2003** | **Great Lakes** | **This study** |
| | | 2007–2009 | East coast of Canada | Reported as *Cochlodinium polykrikoides* [9] |
| | | 2007–2009 | Canada's east coast, west coast and the Great Lakes | Reported as *Cochlodinium polykrikoides* [7] |
| **77** | ***Naiadinium polonicum**** | **2000–2003** | **Great Lakes** | **This study** |
| **78** | ***Oblea rotunda*** | **2000–2003** | **Great Lakes** | **This study** |
| | | Aug–2003 | Finland | [11] |
| | | 2001 (published) | English and Welsh ports | [4] |
| **79** | ***Palatinus apiculatus**** | **2000–2003** | **Great Lakes** | **This study** |
| **80** | ***Pelagodinium béii**** | **2000–2003** | **Great Lakes and the Chesapeake Bay** | **This study** |
| **81** | ***Pellucidodinium psammophilum**** | **2000–2003** | **Great Lakes** | **This study** |
| **82** | ***Pentapharsodinium dalei*** | **2000–2003** | **Great Lakes and the Chesapeake Bay** | **This study** |
| | | 2007–2009 | East coast of Canada | [9] |
| | | 2007–2009 | Canada's east coast, west coast and the Great Lakes | [7] |
| | | 2000 (published) | English and Welsh ports | [8] |
| | | 2001 (published) | English and Welsh ports | [4] |
| | | 1994 | Scottish ports | [5] |
| **83** | ***Pentapharsodinium tyrrhenicum*** | **2000–2003** | **Great Lakes** | **This study** |
| | | 2001 (published) | English and Welsh ports | [4] |
| **84** | ***Peridiniopsis borgei**** | **2000–2003** | **Great Lakes** | **This study** |
| 85 | *Peridinium cinctum* | 1995 | Estuary and Gulf of St. Lawrence | [10] |
| **86** | ***Polarella glacialis**** | **2000–2003** | **Great Lakes** | **This study** |
| **87** | ***Polykrikos geminatum**** | **2000–2003** | **Great Lakes** | **This study** |

| | | | | |
|---|---|---|---|---|
| 88 | *Polykrikos hartmannii* | 1994 | Scottish ports | Reported as *Pheopolykrikos hartmannii* [5] |
| | | 2001 (published) | English and Welsh ports | Reported as *Pheopolykrikos hartmannii* [4] |
| | | 1987–1990 | Australian ports | Reported as *Pheopolykrikos hartmanni* [6] |
| **89** | ***Polykrikos kofoidii*** | **2000–2003** | **Great Lakes and the Chesapeake Bay** | **This study** |
| | | 2007–2009 | Canada's east coast, west coast and the Great Lakes | [7] |
| | | 1987–1990 | Australian ports | [6] |
| 90 | *Polykrikos schwartzii* | 2007–2009 | East coast of Canada | [9] |
| | | 2007–2009 | Canada's east coast, west coast and the Great Lakes | [7] |
| | | 2002–2003 | Great Lakes | [1] |
| | | 1987–1990 | Australian ports | [6] |
| | | 2000 (published) | English and Welsh ports | [8] |
| | | 2001–2002 | Great Lakes | [2] |
| | | 1994 | Scottish ports | [5] |
| | | 2001 (published) | English and Welsh ports | [4] |
| 91 | *Preperidinium meunieri* | 1995 | Estuary and Gulf of St. Lawrence | Reported as *Diplopeltopsis minor* [10] |
| | | 2007–2009 | East coast of Canada | [9] |
| | | 2007–2009 | Canada's east coast, west coast and the Great Lakes | [7] |
| | | 2001 (published) | English and Welsh ports | Reported as *Preperidinium meuneri* [4] |
| | | 1987–1990 | Australian ports | Reported as *Zygabikodinium lenticulatum* [6] |
| | | 2000 (published) | England and Wales | Reported as *Zygabikodinium lenticulatum* [8] |
| **92** | ***Prorocentrum micans*** | **2000–2003** | **Great Lakes** | **This study** |
| | | 1995 | Estuary and Gulf of St. Lawrence | [10] |
| 93 | *Protoceratium reticulatum* | 2001 (published) | English and Welsh ports | Reported as *Gonyaulax grindleyi* [4] |
| | | 2001–2002 | Great Lakes | Reported as *Gonyaulax grindleyi* [2] |
| | | 1987–1990 | Australian ports | [6] |
| | | 2000 (published) | English and Welsh ports | [8] |
| | | 2003–2006 | Port of Tampa and Port Manatee | [3] |

| | | | | |
|---|---|---|---|---|
| | | 2007–2009 | Canada's east coast, west coast and the Great Lakes | [7] |
| | | 2007–2009 | East coast of Canada | [9] |
| | | 1994 | Scottish ports | Reported as *Operculodinium centrocarpum* [5] |
| 94 | *Protoperidinium americanum* | 2007–2009 | East coast of Canada | [9] |
| | | 2007–2009 | Canada's east coast, west coast and the Great Lakes | [7] |
| | | 1987–1990 | Australian ports | [6] |
| | | 2000 (published) | England and Wales | [8] |
| | | 2001 (published) | English and Welsh ports | [4] |
| | | 2001–2002 | Great Lakes | [2] |
| 95 | *Protoperidinium avellana* | 1987–1990 | Australian ports | [6] |
| | | 1994 | Scottish ports | [5] |
| | | 2007–2009 | East coast of Canada | [9] |
| | | 2007–2009 | Canada's east coast, west coast and the Great Lakes | [7] |
| | | 2001–2002 | Great Lakes | [2] |
| 96 | *Protoperidinium claudicans* | 2001–2002 | Great Lakes | [2] |
| | | 2007–2009 | Canada's east coast, west coast and the Great Lakes | [7] |
| | | 2007–2009 | East coast of Canada | [9] |
| 97 | *Protoperidinium compressum* | 2000 (published) | England and Wales | [8] |
| | | 1994 | Scottish ports | [5] |
| | | 2001 (published) | English and Welsh ports | [4] |
| | | 2007–2009 | Canada's east coast, west coast and the Great Lakes | [7] |
| | | 2007–2009 | East coast of Canada | [9] |
| 98 | *Protoperidinium conicoides* | 1987–1990 | Australian ports | [6] |
| | | 2001–2002 | Great Lakes | [2] |
| | | 2007–2009 | East coast of Canada | [9] |
| | | 2001 (published) | English and Welsh ports | [4] |

| | | | | |
|---|---|---|---|---|
| | | 2007–2009 | Canada's east coast, west coast and the Great Lakes | [7] |
| 99 | *Protoperidinium conicum* | 2002–2003 | Great Lakes | [1] |
| | | 1987–1990 | Australian ports | [6] |
| | | 2000 (published) | English and Welsh ports | [8] |
| | | 2001–2002 | Great Lakes | [2] |
| | | 1994 | Scottish ports | [5] |
| | | 1995 | Estuary and Gulf of St. Lawrence | [10] |
| | | 2001 (published) | English and Welsh ports | [4] |
| | | 2007–2009 | Canada's east coast, west coast and the Great Lakes | [7] |
| | | 2007–2009 | East coast of Canada | [9] |
| 100 | *Protoperidinium crassipes* | 1995 | Estuary and Gulf of St. Lawrence | Reported as *Peridinium crassipes* [10] |
| 101 | *Protoperidinium denticulatum* | 1987–1990 | Australian ports | [6] |
| | | 2007–2009 | East coast of Canada | [9] |
| | | 2007–2009 | Canada's east coast, west coast and the Great Lakes | [7] |
| | | 2001–2002 | Great Lakes | [2] |
| 102 | *Protoperidinium divaricatum* | 2001 (published) | English and Welsh ports | [4] |
| | | 2001–2002 | Great Lakes | [2] |
| 103 | *Protoperidinium divergens* | 2003–2006 | Port of Tampa and Port Manatee | [3] |
| | | 1995 | Estuary and Gulf of St. Lawrence | [10] |
| | | 1987–1990 | Australian ports | [6] |
| 104 | *Protoperidinium excentricum* | 2002–2003 | Great Lakes | [1] |
| | | 1987–1990 | Australian ports | [6] |
| | | 2001–2002 | Great Lakes | [2] |
| | | 2001 (published) | English and Welsh ports | [4] |
| 105 | *Protoperidinium expansum* | 1987–1990 | Australian ports | [6] |
| 106 | *Protoperidinium groenlandicum* | Aug–2003 | Finland | Reported as *Peridinium grenlandicum* [11] |
| 107 | *Protoperidinium latissimum* | 2007–2009 | East coast of Canada | [9] |

| | | | | |
|---|---|---|---|---|
| | | 2007–2009 | Canada's east coast, west coast and the Great Lakes | [7] |
| 108 | *Protoperidinium leonis* | 2007–2009 | Canada's east coast, west coast and the Great Lakes | [7] |
| | | 2002–2003 | Great Lakes | [1] |
| | | 1987–1990 | Australian ports | [6] |
| | | 2000 (published) | English and Welsh ports | [8] |
| | | 2001–2002 | Great Lakes | [2] |
| | | 1994 | Scottish ports | [5] |
| | | 2007–2009 | East coast of Canada | [9] |
| | | 2001 (published) | English and Welsh ports | [4] |
| 109 | *Protoperidinium minutum* | 2007–2009 | Canada's east coast, west coast and the Great Lakes | [7] |
| | | 2000 (published) | English and Welsh ports | [8] |
| | | 2001–2002 | Great Lakes | [2] |
| | | 2001 (published) | English and Welsh ports | [4] |
| | | 1994 | Scottish ports | [5] |
| **110** | ***Protoperidinium monovelum**** | **2000–2003** | **Great Lakes** | **This study** |
| 111 | *Protoperidinium nudum* | 2007–2009 | East coast of Canada | [9] |
| | | 2007–2009 | Canada's east coast, west coast and the Great Lakes | [7] |
| | | 2001–2002 | Great Lakes | [2] |
| | | 1987–1990 | Australian ports | [6] |
| 112 | *Protoperidinium oblongum* | 2002–2003 | Great Lakes | [1] |
| | | 1987–1990 | Australian ports | [6] |
| | | 2000 (published) | England and Wales | [8] |
| | | 2001–2002 | Great Lakes | [2] |
| | | 1994 | Scottish ports | [5] |
| | | 2003–2006 | Port of Tampa and Port Manatee | [3] |
| | | 2001 (published) | English and Welsh ports | [4] |

| | | | | |
|---|---|---|---|---|
| | | 2007–2009 | Canada's east coast, west coast and the Great Lakes | [7] |
| | | 2007–2009 | East coast of Canada | [9] |
| 113 | *Protoperidinium obtusum* | 2007–2009 | Canada's east coast, west coast and the Great Lakes | [7] |
| 114 | *Protoperidinium pellucidum* | 1995 | Estuary and Gulf of St. Lawrence | [10] |
| 115 | *Protoperidinium pentagonum* | 1987–1990 | Australian ports | [6] |
| | | 2000 (published) | England and Wales | [8] |
| | | 1994 | Scottish ports | [5] |
| | | 2001 (published) | English and Welsh ports | [4] |
| | | 2007–2009 | Canada's east coast, west coast and the Great Lakes | [7] |
| | | 2007–2009 | East coast of Canada | [9] |
| 116 | *Protoperidinium punctulatum* | 1987–1990 | Australian ports | [6] |
| | | 2001–2002 | Great Lakes | [2] |
| **117** | ***Protoperidinium steidingerae**** | **2000–2003** | **Great Lakes** | **This study** |
| 118 | *Protoperidinium stellatum* | 2007–2009 | East coast of Canada | [9] |
| | | 2007–2009 | Canada's east coast, west coast and the Great Lakes | [7] |
| 119 | *Protoperidinium subinerme* | 2003–2006 | Port of Tampa and Port Manatee | [3] |
| | | 1987–1990 | Australian ports | [6] |
| | | 2001–2002 | Great Lakes | [2] |
| | | 2001 (published) | English and Welsh ports | [4] |
| | | 2007–2009 | East coast of Canada | [9] |
| | | 2002–2003 | Great Lakes | Reported as *Protoperidinium sublinerme* [1] |
| 120 | *Protoperidinium thorianum* | 2001–2002 | Great Lakes | [2] |
| | | 2007–2009 | Canada's east coast, west coast and the Great Lakes | [7] |
| 121 | *Pyrocystis lunula* | 1987–1990 | Australian ports | [6] |
| 122 | *Pyrocystis pseudonoctiluca* | 2001 (published) | English and Welsh ports | Reported as *Pyrocystis noctiluca* [4] |

| | | | | |
|---|---|---|---|---|
| 123 | *Pyrodinium bahamense* | 2007–2009 | Canada's east coast, west coast and the Great Lakes | [7] |
| 124 | *Pyrophacus horologicum* | 2001 (published) | English and Welsh ports | [4] |
| 125 | *Pyrophacus steinii* | 2003–2006 | Port of Tampa and Port Manatee | [3] |
| | | 2007–2009 | Canada's east coast, west coast and the Great Lakes | [7] |
| | | 2007–2009 | East coast of Canada | [9] |
| | | 2001 (published) | English and Welsh ports | [4] |
| 126 | *Qia lebouriae* | 1987–1990 | Australian ports | Reported as *Diplopsalis lebourae* [6] |
| **127** | ***Scrippsiella acuminata*** | **2000–2003** | **Great Lakes and the Chesapeake Bay** | **This study** |
| | | 1987–1990 | Australian ports | Reported as *Peridinium faeroense* [6] |
| | | 2007–2009 | Canada's east coast, west coast and the Great Lakes | Reported as *Scrippsiella trochoidea* [7] |
| | | 2002–2003 | Great Lakes | Reported as *Scrippsiella trochoidea* [1] |
| | | 1987–1990 | Australian ports | Reported as *Scrippsiella trochoidea* [6] |
| | | 2000 (published) | England and Wales | Reported as *Scrippsiella trochoidea* [8] |
| | | 2001–2002 | Great Lakes | Reported as *Scrippsiella trochoidea* [2] |
| | | 1994 | Scottish ports | Reported as *Scrippsiella trochoidea* [5] |
| | | 2007–2009 | East coast of Canada | Reported as *Scrippsiella trochoidea* [9] |
| | | 1995 | Estuary and Gulf of St. Lawrence | Reported as *Scrippsiella trochoidea* [10] |
| | | 2001 (published) | English and Welsh ports | Reported as *Scrippsiella trochoidea* [4] |
| 128 | *Scrippsiella crystallina* | 2001–2002 | Great Lakes | [2] |
| | | 2001 (published) | English and Welsh ports | [4] |
| | | 1994 | Scottish ports | [5] |
| **129** | ***Scrippsiella donghaienis**** | **2000–2003** | **Great Lakes** | **This study** |
| 130 | *Scrippsiella lachrymosa* | 2000 (published) | England and Wales | [8] |
| | | 1994 | Scottish ports | [5] |
| | | 2001 (published) | English and Welsh ports | [4] |
| | | 2007–2009 | Canada's east coast, west coast and the Great Lakes | [7] |

| | | | | |
|---|---|---|---|---|
| | | 2001–2002 | Great Lakes | [2] |
| 131 | *Scrippsiella precaria* | 1987–1990 | Australian ports | [6] |
| | | 1994 | Scottish ports | [5] |
| 132 | *Scrippsiella rotunda* | 1994 | Scottish ports | [5] |
| | | 2001 (published) | English and Welsh ports | [4] |
| **133** | ***Scrippsiella sweeneyae**** | **2000–2003** | **Great Lakes** | **This study** |
| 134 | *Scrippsiella trifida* | 1994 | Scottish ports | [5] |
| | | 2001 (published) | English and Welsh ports | [4] |
| **135** | ***Takayama acrotrocha**** | **2000–2003** | **Great Lakes** | **This study** |
| **136** | ***Takayama helix**** | **2000–2003** | **Great Lakes** | **This study** |
| **137** | ***Tovellia sanguinea**** | **2000–2003** | **Great Lakes** | **This study** |
| 138 | *Tripos fusus* | 1995 | Estuary and Gulf of St. Lawrence | Reported as *Ceratium fusus* [10] |
| 139 | *Tripos longipes* | 1995 | Estuary and Gulf of St. Lawrence | Reported as *Ceratium longipes* [10] |
| **140** | ***Tyrannodinium edax**** | **2000–2003** | **Great Lakes and the Chesapeake Bay** | **This study** |
| 141 | *Vulcanodinium rugosum* | Jul–2004 | Port Tampa Bay, Florida, USA | [12] |
| **142** | ***Woloszynskia pascheri**** | **2000–2003** | **Great Lakes and the Chesapeake Bay** | **This study** |

The synonyms of the species were checked according to the information provided by AlgaeBase (http://www.algaebase.org). Species reported in this study were in bold. Species first reported in ballast tank sediments in this study were marked with an asterisk.

**References:**


1. Fahnenstiel, G.; Hong, Y.; Millie, D.; Doblin, M.; Johengen, T.; Reid, D. Marine dinoflagellate cysts in the ballast tank sediments of ships entering the Laurentian Great Lakes. *International Association of Theoretical and Applied Limnology* **2009**, *30*, 1035–1038, doi:10.2307/4563729.
2. Johengen, T.H.; Reid, D.; Fahnenstiel, G.; MacIsaac, H.; Dobbs, F.; Doblin, M.; Ruiz, G.; Jenkins, P. *Assessment of transoceanic NOBOB vessels and low–salinity ballast water as vectors for nonindigenous species introductions to the Great Lakes*; University of Michigan and NOAA–Great Lakes Environmental Research Laboratory: Ann Arbor, MI, 2005; pp 3–1–3–53.
3. Garrett, M.J.; Wolny, J.L.; Williams, B.J.; Dirks, M.D.; Brame, J.A.; Richardson, R.W. Methods for sampling and analysis of marine microalgae in ship ballast tanks: a case study from Tampa Bay, Florida, USA. *Algae* **2011**, *26*, 181–192.
4. Hamer, J.P.; Lucas, I.A.N.; McCollin, T.A. Harmful dinoflagellate resting cysts in ships' ballast tank sediments: potential for introduction into English and Welsh waters. *Phycologia* **2001**, *40*, 246–255, doi:10.2216/i0031–8884–40–3–246.1.
5. Macdonald, E.M. *Dinoflagellate resting cysts and ballast water discharges in Scottish ports*; Aalborg, Denmark, 1995.
6. Hallegraeff, G.M.; Bolch, C.J. Transport of diatom and dinoflagellate resting spores in ships' ballast water: implications for plankton biogeography and aquaculture. *J. Plankton Res.* **1992**, *14*, 1067–1084, doi:10.1093/plankt/14.8.1067.
7. Casas–Monroy, O.; Roy, S.; Rochon, A. Dinoflagellate cysts in ballast sediments: differences between Canada's east coast, west coast and the Great Lakes. *Aquat. Conserv. Mar. Freshwater Ecosyst.* **2013**, *23*, 254–276, doi:10.1002/aqc.2310.

8. Hamer, J.P.; McCollin, T.A.; Lucas, I.A.N. Dinoflagellate cysts in ballast tank sediments: between tank variability. *Mar. Pollut. Bull.* **2000**, *40*, 731–733, doi:http://dx.doi.org/10.1016/S0025–326X(99)00198–8.
9. Casas–Monroy, O.; Roy, S.; Rochon, A. Ballast sediment–mediated transport of non–indigenous species of dinoflagellates on the East Coast of Canada. *Aquatic Invasions* **2011**, *6*, 231–248, doi:10.3391/ai.2011.6.3.01.
10. Harvey, M.; Gilbert, M.; Gauthierl, D.; Reid, D.M. A preliminary assessment of risks for the ballast water–mediated introduction of nonindigenous marine organisms in the Estuary and Gulf of St. Lawrence. *Journal of Geo–Information Science* **1999**, *12*, 89–94.
11. Pertola, S.; Faust, M.A.; Kuosa, H. Survey on germination and species composition of dinoflagellates from ballast tanks and recent sediments in ports on the South Coast of Finland, North–Eastern Baltic Sea. *Mar. Pollut. Bull.* **2006**, *52*, 900–911, doi:http://dx.doi.org/10.1016/j.marpolbul.2005.11.028.
12. Garrett, M.J.; Puchulutegui, C.; Selwood, A.I.; Wolny, J.L. Identification of the harmful dinoflagellate *Vulcanodinium rugosum* recovered from a ballast tank of a globally traveled ship in Port Tampa Bay, Florida, USA. *Harmful Algae* **2014**, *39*, 202–209, doi:10.1016/j.hal.2014.07.014.

**Table S9.** Dinoflagellate species reported in ships' ballast water.

| No. | Species | Sampling Time | Sampling Port | References |
|---|---|---|---|---|
| 1 | *Akashiwo sanguinea* | 2007–2009 | South Korean ports | [1] |
| | | 1997–2006 | Bilbao Harbour, Spain | [2] |
| | | 2002–2004 | Ports on the U.S. East and West Coast | Reported as *Akashiwo sanguineum* [3] |
| | | 2007–2009 | West and east coasts of Canada | Reported as *Akashiwo sanguineum* [4] |
| 2 | *Alexandrium balechii* | 2003–2006 | Port of Tampa and Port Manatee | [5] |
| 3 | *Alexandrium minutum* | 2007–2009 | West and east coasts of Canada | [4] |
| | | 1997–2006 | Bilbao Harbour, Spain | [2] |
| | | 2015 | Adriatic ports | [6] |
| 4 | *Alexandrium monilatum* | 2007/05–2010/03 | Port of Houston, Texas (USA) | [7] |
| 5 | *Alexandrium ostenfeldii* | 2007–2009 | West and east coasts of Canada | [4] |
| 6 | *Alexandrium pacificum* | 1996–1997 | Hongkong, China | Reported as *Alexandrium catenella* [8] |
| | | 2007–2008 | Patagonian ports, Argentina | Reported as *Alexandrium catenella* [9] |
| 7 | *Alexandrium pseudogonyaulax* | 2007–2009 | Ports of eastern Canada | [10] |
| | | 2007–2009 | West and east coasts of Canada | [4] |
| 8 | *Alexandrium tamarense* | 2007–2009 | Ports of eastern Canada | [10] |
| | | 2007–2008 | Patagonian ports, Argentina | [9] |
| | | 2007–2009 | West and east coasts of Canada | [4] |
| | | 2013–2014 | Port of Annaba, Algeria | [11] |
| 9 | *Amphidinium acutum* | 2003 | Slovenian Sea | [12] |
| 10 | *Amphidinium sphenoides* | 2007–2009 | Ports of eastern Canada | [10] |
| | | 2007–2009 | West and east coasts of Canada | [4] |
| 11 | *Amylax triacantha* | 2007–2009 | West and east coasts of Canada | [4] |
| | | 2007–2009 | Ports of eastern Canada | [10] |
| 12 | *Apocalathium aciculiferum* | 2002–2004 | Ports on the U.S. East and West Coast | Reported as *Peridinium aciculiferum* [3] |

| | | | | |
|---|---|---|---|---|
| 13 | *Apocalathium malmogiense* | 1998/07/22–1998/08/27 | Open Atlantic Coast of Europe | Reported as *Scrippsiella hangoi* [13] |
| 14 | *Archaeperidinium minutum* | 2007–2009 | Ports of eastern Canada | Reported as *Protoperidinium minutum* [10] |
| | | 2007–2009 | West and east coasts of Canada | Reported as *Protoperidinium minutum* [4] |
| 15 | *Barrufeta resplendens* | 2007–2009 | West and east coasts of Canada | Reported as *Gyrodinium resplendens* [4] |
| 16 | *Ceratim fusus* | 1996–1997 | Hongkong, China | Reported as *Ceratium fusus* [8] |
| 17 | *Ceratium arcticum* | 2007–2009 | Ports of eastern Canada | Reported as *Neoceratium arcticum* [10] |
| 18 | *Ceratium contrarium* | 1995 | Estuary and Gulf of St. Lawrence | [14] |
| 19 | *Ceratium declinatum* | 1995 | Estuary and Gulf of St. Lawrence | [14] |
| 20 | *Ceratium falcatum* | 1995 | Estuary and Gulf of St. Lawrence | [14] |
| 21 | *Ceratium furca* | 2010 | Ulsan and Onsan ports, Korea | [15] |
| | | 2002–2004 | Ports on the U.S. East and West Coast | [3] |
| | | 2003 | Slovenian Sea | [12] |
| | | 2003–2006 | Port of Tampa and Port Manatee | [5] |
| | | 1995 | Estuary and Gulf of St. Lawrence | [14] |
| | | 2007–2009 | South Korean ports | [1] |
| | | 2010–2012 | France | [16] |
| | | 1990–1991 | Laurentian Great Lakes and upper St. Lawrence River | [17] |
| | | 2007–2008 | Patagonian ports, Argentina | [9] |
| 22 | *Ceratium gibberum* | 2007–2008 | Patagonian ports, Argentina | [9] |
| | | 1995 | Estuary and Gulf of St. Lawrence | [14] |
| 23 | *Ceratium hirundinella* | 2007–2008 | Patagonian ports, Argentina | [9] |
| | | 2007–2009 | West and east coasts of Canada | [4] |
| | | 2013–2014 | Strait of Gibraltar and Deception Bay, Canada | Reported as *Neoceratium hyrundinella* [18] |
| 24 | *Ceratium horridum* | 2003 | Slovenian Sea | [12] |
| | | 1995 | Estuary and Gulf of St. Lawrence | [14] |
| | | 2007–2009 | South Korean ports | [1] |
| | | 2007–2008 | Patagonian ports, Argentina | [9] |
| 25 | *Ceratium inflatum* | 2007–2009 | Ports of eastern Canada | Reported as *Neoceratium inflatum* [10] |
| 26 | *Ceratium lineatum* | 2007–2009 | Ports of eastern Canada | Reported as *Neoceratium lineatum* [10] |
| 27 | *Ceratium longirostrum* | 1995 | Estuary and Gulf of St. Lawrence | [14] |

| | | | | |
|---|---|---|---|---|
| | | 2007–2009 | Ports of eastern Canada | Reported as *Neoceratium longirostrum* [10] |
| 28 | *Ceratium massiliense* | 1995 | Estuary and Gulf of St. Lawrence | [14] |
| | | 2007–2008 | Patagonian ports, Argentina | [9] |
| 29 | *Ceratium symmetricum* | 1995 | Estuary and Gulf of St. Lawrence | [14] |
| 30 | *Ceratium trichoceros* | 2003–2006 | Port of Tampa and Port Manatee | [5] |
| | | 1995 | Estuary and Gulf of St. Lawrence | [14] |
| 31 | *Ceratium tripos* | 2002–2004 | Ports on the U.S. East and West Coast | [3] |
| | | 1995 | Estuary and Gulf of St. Lawrence | [14] |
| | | 2007–2009 | South Korean ports | [1] |
| | | 2010–2012 | France | [16] |
| | | 2007–2008 | Patagonian ports, Argentina | [9] |
| 32 | *Corynthodinium diploconus* | 2007–2009 | West and east coasts of Canada | [4] |
| | | 2007–2009 | Ports of eastern Canada | [10] |
| 33 | *Corythodinium curvicaudatum* | 2007–2009 | West and east coasts of Canada | [4] |
| | | 2007–2009 | Ports of eastern Canada | [10] |
| 34 | *Corythodinium diploconus* | 1990–1991 | Laurentian Great Lakes and upper St. Lawrence River | Reported as *Corynthodinium diplococcus* [17] |
| 35 | *Corythodinium frenguellii* | 2007–2009 | West and east coasts of Canada | Reported as *Oxytoxum frenguellii* [4] |
| 36 | *Corythodinium michaelsarsii* | 2007–2009 | West and east coasts of Canada | Reported as *Oxytoxum michaelsarsii* [4] |
| 37 | *Cucumeridinium coeruleum* | 2002–2004 | Ports on the U.S. East and West Coast | Reported as *Balechina coerulea* [3] |
| 38 | *Dinophysis acuminata* | 2007–2008 | Patagonian ports, Argentina | [9] |
| | | 2002–2004 | Ports on the U.S. East and West Coast | [3] |
| | | 2010–2012 | France | [16] |
| | | 1998/07/22–1998/08/27 | Open Atlantic Coast of Europe | [13] |
| | | 1990–1991 | Laurentian Great Lakes and upper St. Lawrence River | [17] |
| | | 2007 | Vladivostok Port | [19] |
| | | 2004/2005/2007 | Novorossiysk, Russia | [20] |
| | | 2007–2008 | Patagonian ports, Argentina | [9] |
| | | 2007–2008 | Patagonian ports, Argentina | [9] |
| | | 1997–2006 | Bilbao Harbour, Spain | [2] |

| | | | | |
|---|---|---|---|---|
| | | 2007–2009 | West and east coasts of Canada | [4] |
| | | 2007–2009 | Ports of eastern Canada | [10] |
| | | 2008 | Tunisian harbours of La Goulette and Biz– erte | [21] |
| | | 2013–2014 | Port of Annaba, Algeria | [11] |
| | | 2007–2008 | Patagonian ports, Argentina | Reported as *Dinophysis lachmanni* [9] |
| 39 | *Dinophysis acuta* | 2013–2014 | Strait of Gibraltar and Deception Bay, Canada | [18] |
| | | 2010–2012 | France | [16] |
| | | 1997–2006 | Bilbao Harbour, Spain | [2] |
| | | 2007–2009 | West and east coasts of Canada | [4] |
| | | 2007–2009 | Ports of eastern Canada | [10] |
| | | 2007–2009 | West and east coasts of Canada | Reported as *Dinophysis dens* [4] |
| | | 2007–2009 | Ports of eastern Canada | Reported as *Dinophysis dens* [10] |
| 40 | *Dinophysis caudata* | 2002–2004 | Ports on the U.S. East and West Coast | [3] |
| | | 2010–2012 | France | [16] |
| | | 2007–2008 | Patagonian ports, Argentina | [9] |
| | | 1997–2006 | Bilbao Harbour, Spain | [2] |
| | | 1996–1997 | Hongkong, China | [8] |
| | | 2007–2009 | West and east coasts of Canada | [4] |
| | | 2007–2009 | Ports of eastern Canada | [10] |
| | | 2015 | Adriatic ports | [6] |
| 41 | *Dinophysis contract* | 2007–2009 | Ports of eastern Canada | Reported as *Phalacroma contractum* [10] |
| | | 2007–2009 | West and east coasts of Canada | [4] |
| 42 | *Dinophysis fortii* | 1997–2006 | Bilbao Harbour, Spain | [2] |
| | | 2010–2012 | France | [16] |
| | | 2007–2009 | West and east coasts of Canada | [4] |
| | | 2007–2009 | Ports of eastern Canada | [10] |
| 43 | *Dinophysis norvegica* | 2013–2014 | Strait of Gibraltar and Deception Bay, Canada | [18] |
| | | 1995 | Estuary and Gulf of St. Lawrence | [14] |
| | | 1990–1991 | Laurentian Great Lakes and upper St. Lawrence River | [17] |
| | | 2007–2009 | West and east coasts of Canada | [4] |

| | | | | |
|---|---|---|---|---|
| | | 2007–2009 | Ports of eastern Canada | [10] |
| 44 | *Dinophysis odiosa* | 2007–2009 | West and east coasts of Canada | [4] |
| | | 2007–2009 | Ports of eastern Canada | [10] |
| 45 | *Dinophysis ovum* | 2007–2009 | Ports of eastern Canada | [10] |
| | | 1990–1991 | Laurentian Great Lakes and upper St. Lawrence River | [17] |
| | | 2007–2009 | West and east coasts of Canada | [4] |
| 46 | *Dinophysis parva* | 2007–2009 | West and east coasts of Canada | [4] |
| 47 | *Dinophysis pulchella* | 2007–2009 | West and east coasts of Canada | Reported as *Phalacroma pulchellum* [4] |
| | | 2007–2009 | Ports of eastern Canada | Reported as *Phalacroma pulchellum* [10] |
| 48 | *Dinophysis sacculus* | 2010–2012 | France | [16] |
| | | 2015 | Adriatic ports | [6] |
| 49 | *Dinophysis simplex* | 2007–2009 | Ports of eastern Canada | [10] |
| | | 2007–2009 | West and east coasts of Canada | [4] |
| 50 | *Dinophysis tripos* | 1990–1991 | Laurentian Great Lakes and upper St. Lawrence River | [17] |
| | | 2007–2008 | Patagonian ports, Argentina | [9] |
| | | 2007–2009 | West and east coasts of Canada | [4] |
| | | 2007–2009 | Ports of eastern Canada | [10] |
| 51 | *Dinophysis acuminata* | 2003 | Slovenian Sea | [12] |
| | | 2013–2014 | Port of Annaba, Algeria | [11] |
| 52 | *Diplopsalis lenticula* | 2007–2009 | South Korean ports | [1] |
| | | 2007–2009 | West and east coasts of Canada | [4] |
| | | 2007–2009 | Ports of eastern Canada | [10] |
| 53 | *Diplopsalopsis bomba* | 2007–2009 | West and east coasts of Canada | [4] |
| | | 2007–2009 | Ports of eastern Canada | [10] |
| 54 | *Dissodinium pseudocalani* | 1995 | Estuary and Gulf of St. Lawrence | [14] |
| 55 | *Dissodinium pseudolunula* | 1995 | Estuary and Gulf of St. Lawrence | [14] |
| | | 1990–1991 | Laurentian Great Lakes and upper St. Lawrence River | [17] |
| | | 2007–2009 | West and east coasts of Canada | [4] |

| | | | | |
|---|---|---|---|---|
| 56 | *Exuviella pusilla* | 2007/05–2010/03 | Port of Houston, Texas (USA) | [7] |
| 57 | *Gambierdiscus toxicus* | 2002–2004 | Ports on the U.S. East and West Coast | [3] |
| 58 | *Goniodoma sphaericum* | 2007–2009 | West and east coasts of Canada | [4] |
| | | 2007–2009 | Ports of eastern Canada | [10] |
| 59 | *Gonyaulax alaskensis* | 2007–2009 | West and east coasts of Canada | [4] |
| | | 2007–2009 | Ports of eastern Canada | [10] |
| 60 | *Gonyaulax diegensis* | 2007–2009 | South Korean ports | [1] |
| | | 2007–2009 | West and east coasts of Canada | [4] |
| | | 2007–2009 | Ports of eastern Canada | [10] |
| 61 | *Gonyaulax digitalis* | 2007–2009 | Ports of eastern Canada | [10] |
| | | 2003–2006 | Port of Tampa and Port Manatee | [5] |
| | | 2007–2008 | Patagonian ports, Argentina | [9] |
| | | 2007–2009 | West and east coasts of Canada | [4] |
| 62 | *Gonyaulax fragilis* | 2015 | Adriatic ports | [6] |
| 63 | *Gonyaulax polygramma* | 2003–2006 | Port of Tampa and Port Manatee | [5] |
| | | 2007–2009 | West and east coasts of Canada | [4] |
| | | 2007–2009 | Ports of eastern Canada | [10] |
| 64 | *Gonyaulax rostratum* | 1995 | Estuary and Gulf of St. Lawrence | Reported as *Gonyaulnr rostratum* [14] |
| 65 | *Gonyaulax scrippsae* | 2007–2009 | West and east coasts of Canada | [4] |
| | | 2007–2009 | Ports of eastern Canada | [10] |
| 66 | *Gonyaulax spinifera* | 2007–2009 | Ports of eastern Canada | [10] |
| | | 2013–2014 | Strait of Gibraltar and Deception Bay, Canada | [18] |
| | | 2002–2004 | Ports on the U.S. East and West Coast | [3] |
| | | 2003–2006 | Port of Tampa and Port Manatee | [5] |
| | | 2007–2009 | West and east coasts of Canada | [4] |
| | | 2013–2014 | Port of Annaba, Algeria | [11] |
| 67 | *Gonyaulax verior* | 2007–2009 | Ports of eastern Canada | [10] |
| | | 2007–2009 | West and east coasts of Canada | [4] |
| 68 | *Gymnodinium albulum* | 1996/04–1996/09 | Israeli Mediterranean ports | [22] |
| 69 | *Gymnodinium aureolum* | 2007–2009 | West and east coasts of Canada | [4] |
| | | 2007–2009 | Ports of eastern Canada | [10] |

| | | | | |
|---|---|---|---|---|
| 70 | *Gymnodinium catenatum* | 2013–2014 | Port of Annaba, Algeria | [11] |
| 71 | *Gymnodinium galeatum* | 2007–2009 | Ports of eastern Canada | [10] |
| | | 2007–2009 | West and east coasts of Canada | [4] |
| 72 | *Gymnodinium simplex* | 2007 | Vladivostok Port | [19] |
| | | 2004/2005/2007 | Novorossiysk, Russia | [20] |
| 73 | *Gymnodinium variabile* | 2007–2009 | West and east coasts of Canada | [4] |
| | | 2007–2009 | Ports of eastern Canada | [10] |
| 74 | *Gyrodinium crassum* | 2007–2009 | Ports of eastern Canada | [10] |
| | | 2007–2009 | West and east coasts of Canada | [4] |
| 75 | *Gyrodinium dominans* | 2008/06–2009/05 | Texas coast and opens into the Gulf of Mexico, United States | [23] |
| 76 | *Gyrodinium estuariale* | 2007–2009 | West and east coasts of Canada | [4] |
| 77 | *Gyrodinium fusiforme* | 2013–2014 | Strait of Gibraltar and Deception Bay, Canada | [18] |
| | | 2007 | Vladivostok Port | [19] |
| | | 2004/2005/2007 | Novorossiysk, Russia | [20] |
| 78 | *Gyrodinium fusus* | 2007–2009 | Ports of eastern Canada | [10] |
| | | 2007–2009 | West and east coasts of Canada | [4] |
| 79 | *Gyrodinium lachryma* | 1990–1991 | Laurentian Great Lakes and upper St. Lawrence River | [17] |
| | | 2007 | Vladivostok Port | [19] |
| | | 2004/2005/2007 | Novorossiysk, Russia | [20] |
| 80 | *Gyrodinium pepo* | 2007–2009 | West and east coasts of Canada | [4] |
| | | 2007–2009 | Ports of eastern Canada | [10] |
| 81 | *Gyrodinium pingue* | 2007–2009 | West and east coasts of Canada | [4] |
| | | 2003–2006 | Port of Tampa and Port Manatee | [5] |
| 82 | *Gyrodinium spirale* | 2007–2009 | Ports of eastern Canada | [10] |
| | | 2007–2009 | West and east coasts of Canada | [4] |
| | | 2007–2008 | Patagonian ports, Argentina | [9] |
| 83 | *Heterocapsa minima* | 2015 | Adriatic ports | [6] |
| 84 | *Heterocapsa niei* | 2015 | Adriatic ports | [6] |
| 85 | *Heterocapsa rotundata* | 2002–2004 | Ports on the U.S. East and West Coast | [3] |

| | | | | |
|---|---|---|---|---|
| | | 2007/05–2010/03 | Port of Houston, Texas (USA) | [7] |
| | | 2008/06–2009/05 | Texas coast and opens into the Gulf of Mexico, United States | [23] |
| 86 | *Heterocapsa triquetra* | 2013–2014 | Strait of Gibraltar and Deception Bay, Canada | [18] |
| | | 2007–2009 | Ports of eastern Canada | [10] |
| | | 2002–2004 | Ports on the U.S. East and West Coast | [3] |
| | | 1998/07/22–1998/08/27 | Open Atlantic Coast of Europe | [13] |
| | | 2007–2009 | West and east coasts of Canada | [4] |
| | | 2007–2008 | Patagonian ports, Argentina | [9] |
| | | 1997–2006 | Bilbao Harbour, Spain | [2] |
| 87 | *Islandinium minutum* | 2013–2014 | Port of Annaba, Algeria | [11] |
| 88 | *Karenia mikimotoi* | 1997–2006 | Bilbao Harbour, Spain | [2] |
| 89 | *Karenia papilionacea* | 1997–2006 | Bilbao Harbour, Spain | [2] |
| 90 | *Karlodinium australe* | 2002–2004 | Ports on the U.S. East and West Coast | [3] |
| 91 | *Karlodinium decipiens* | 1997–2006 | Bilbao Harbour, Spain | [2] |
| 92 | *Karlodinium veneficum* | 2002–2004 | Ports on the U.S. East and West Coast | [3] |
| | | 1997–2006 | Bilbao Harbour, Spain | [2] |
| | | 2003–2006 | Port of Tampa and Port Manatee | [5] |
| 93 | *Kofoidinium velelloides* | 2007–2009 | West and east coasts of Canada | [4] |
| | | 2007–2009 | Ports of eastern Canada | [10] |
| 94 | *Kryptoperidinium foliaceum* | 2003–2006 | Port of Tampa and Port Manatee | [5] |
| 95 | *Lebouridinium glaucum* | 2003–2006 | Port of Tampa and Port Manatee | Reported as *Katodinium glaucum* [5] |
| | | 2007 | Vladivostok Port | Reported as *Katodinium glaucum* [19] |
| | | 2004/2005/2007 | Novorossiysk, Russia | Reported as *Katodinium glaucum* [20] |
| | | 2007–2009 | West and east coasts of Canada | Reported as *Katodinium glaucum* [4] |
| 96 | *Lingulodinium polyedrum* | 2002–2004 | Ports on the U.S. East and West Coast | [3] |
| | | 1997–2006 | Bilbao Harbour, Spain | [2] |
| | | 2003–2006 | Port of Tampa and Port Manatee | [5] |
| | | 2007–2009 | West and east coasts of Canada | [4] |
| | | 2007–2009 | West and east coasts of Canada | Reported as *Lingulodinium polyedra* [4] |
| | | 2013–2014 | Port of Annaba, Algeria | [11] |

| | | | | | |
|---|---|---|---|---|---|
| 97 | *Noctiluca scintillans* | 2004/2005/2007 | Novorossiysk, Russia | [20] | |
| | | 2007–2008 | Patagonian ports, Argentina | [9] | |
| | | 1997–2006 | Bilbao Harbour, Spain | [2] | |
| | | 2004 – 2005 | Port of Novorossiysk in Russia | [24] | |
| | | 2006–2008 | Canadian ports | [25] | |
| | | 2013–2014 | Port of Annaba, Algeria | [11] | |
| | | 2015 | Adriatic ports | [6] | |
| 98 | *Oblea baculifera* | 2007–2008 | Patagonian ports, Argentina | [9] | |
| 99 | *Oblea rotunda* | 2007–2009 | Ports of eastern Canada | [10] | |
| | | 1998/07/22–1998/08/27 | Open Atlantic Coast of Europe | [13] | |
| | | 2007–2009 | West and east coasts of Canada | [4] | |
| | | 2007 | Vladivostok Port | Reported as *Oblea rotundata* | [19] |
| | | 2004/2005/2007 | Novorossiysk, Russia | Reported as *Oblea rotundata* | [20] |
| 100 | *Ornithocercus magnificus* | 2002–2004 | Ports on the U.S. East and West Coast | [3] | |
| 101 | *Ostreopsis siamensis* | 1997–2006 | Bilbao Harbour, Spain | [2] | |
| 102 | *Oxyrrhis marina* | 2002–2004 | Ports on the U.S. East and West Coast | [3] | |
| 103 | *Oxytoxum caudatum* | 2015 | Adriatic ports | [6] | |
| 104 | *Oxytoxum laticeps* | 2007–2009 | Ports of eastern Canada | [10] | |
| | | 1990–1991 | Laurentian Great Lakes and upper St. Lawrence River | [17] | |
| | | 2007–2009 | West and east coasts of Canada | [4] | |
| 105 | *Oxytoxum longiceps* | 2015 | Adriatic ports | [6] | |
| 106 | *Oxytoxum michaelsarsii* | 2007–2009 | Ports of eastern Canada | [10] | |
| 107 | *Oxytoxum milneri* | 1990–1991 | Laurentian Great Lakes and upper St. Lawrence River | [17] | |
| 108 | *Oxytoxum scolopax* | 2007–2009 | West and east coasts of Canada | [4] | |
| | | 2007–2009 | Ports of eastern Canada | [10] | |
| 109 | *Oxytoxum viride* | 2015 | Adriatic ports | [6] | |
| 110 | *Peridiniella danica* | 2002–2004 | Ports on the U.S. East and West Coast | [3] | |
| 111 | *Peridinium ovatum* | 1990–1991 | Laurentian Great Lakes and upper St. Lawrence River | [17] | |

| | | | | |
|---|---|---|---|---|
| 112 | *Peridinium pellucidum* | 1990–1991 | Laurentian Great Lakes and upper St. Lawrence River | [17] |
| 113 | *Peridinium quadridentatum* | 2007–2009 | West and east coasts of Canada | Reported as *Peridinium quinquecorne* [4] |
| 114 | *Peridinium volzii* | 2007–2009 | West and east coasts of Canada | [4] |
| 115 | *Peridinium willei* | 2007–2009 | West and east coasts of Canada | [4] |
| 116 | *Pfiesteria piscicida* | May–03 | Burnie, Tasmania | [26] |
| | | 2007/05–2010/03 | Port of Houston, Texas (USA) | Reported as *Pfiesteria piscida* [7] |
| | | 2002/06–2002/07 | Chesapeake Bay | [27] |
| 117 | *Phalacroma mitra* | 2007–2009 | West and east coasts of Canada | [4] |
| | | 2007–2009 | Ports of eastern Canada | [10] |
| 118 | *Phalacroma oxytoxoides* | 2007–2009 | West and east coasts of Canada | Reported as *Oxyphysis oxytoxoides* [4] |
| | | 2007–2008 | Patagonian ports, Argentina | Reported as *Oxyphysis oxytoxoides* [9] |
| | | 2015 | Adriatic ports | [6] |
| 119 | *Phalacroma rotundatum* | 1997–2006 | Bilbao Harbour, Spain | Reported as *Dinophysis rotundata* [2] |
| | | 2007–2009 | West and east coasts of Canada | Reported as *Dinophysis rotundata* [4] |
| | | 2002–2004 | Ports on the U.S. East and West Coast | [3] |
| | | 1995 | Estuary and Gulf of St. Lawrence | [14] |
| | | 2007–2009 | West and east coasts of Canada | [4] |
| | | 2007–2009 | Ports of eastern Canada | [10] |
| 120 | *Podolampas palmipes* | 2002–2004 | Ports on the U.S. East and West Coast | [3] |
| | | 2007–2009 | West and east coasts of Canada | [4] |
| | | 2007–2009 | Ports of eastern Canada | [10] |
| | | 2003–2006 | Port of Tampa and Port Manatee | [5] |
| 121 | *Podolampas spinifera* | 2007–2009 | West and east coasts of Canada | [4] |
| | | 2007–2009 | Ports of eastern Canada | [10] |
| 122 | *Polykrikos hartmannii* | 2003–2006 | Port of Tampa and Port Manatee | Reported as *Pheopolykrikos hartmannii* [5] |
| 123 | *Polykrikos kofoidii* | 2003–2006 | Port of Tampa and Port Manatee | [5] |
| | | 1990–1991 | Laurentian Great Lakes and upper St. Lawrence River | [17] |
| | | 2007–2009 | Ports of eastern Canada | [10] |
| | | 2013–2014 | Port of Annaba, Algeria | [11] |

| | | | | |
|---|---|---|---|---|
| | (vegetative cell and cyst) | 2007–2009 | West and east coasts of Canada | [4] |
| 124 | *Polykrikos schwartzi* | 2007–2009 | Ports of eastern Canada | [10] |
| | (cyst) | 2007–2009 | West and east coasts of Canada | [4] |
| 125 | *Preperidinium meunieri* | 2007–2009 | West and east coasts of Canada | [4] |
| | | 2007–2009 | Ports of eastern Canada | [10] |
| 126 | *Prorocentrum balticum* | 2007–2009 | West and east coasts of Canada | [4] |
| | | 1996–1997 | Hongkong, China | [8] |
| 127 | *Prorocentrum cordatum* | 2007 | Vladivostok Port | [19] |
| | | 2004/2005/2007 | Novorossiysk, Russia | [20] |
| | | 2015 | Adriatic ports | [6] |
| | | 2002–2004 | Ports on the U.S. East and West Coast | Reported as *Prorocentrum minimum* [3] |
| | | 2007–2009 | South Korean ports | Reported as *Prorocentrum minimum* [1] |
| | | 1998/07/22–1998/08/27 | Open Atlantic Coast of Europe | Reported as *Prorocentrum minimum* [13] |
| | | 1996–1997 | Hongkong, China | Reported as *Prorocentrum minimum* [8] |
| | | 1997–2006 | Bilbao Harbour, Spain | Reported as *Prorocentrum minimum* [2] |
| 128 | *Prorocentrum dactylus* | 2015 | Adriatic ports | [6] |
| 129 | *Prorocentrum gracile* | 2007–2009 | Ports of eastern Canada | [10] |
| | | 2007–2009 | South Korean ports | [1] |
| | | 1990–1991 | Laurentian Great Lakes and upper St. Lawrence River | [17] |
| | | 1997–2006 | Bilbao Harbour, Spain | [2] |
| | | 2008 | Tunisian harbours of La Goulette and Biz– erte | [21] |
| | | 2007–2009 | West and east coasts of Canada | [4] |
| | | 2015 | Adriatic ports | [6] |
| | | 2007–2009 | West and east coasts of Canada | Reported as *Prorocentrum sigmoides* [4] |
| | | 1996–1997 | Hongkong, China | Reported as *Prorocentrum sigmoides* [8] |
| 130 | *Prorocentrum lima* | 2007–2009 | Ports of eastern Canada | [10] |
| | | 1995 | Estuary and Gulf of St. Lawrence | [14] |
| | | 2007–2009 | West and east coasts of Canada | [4] |
| 131 | *Prorocentrum mexicanum* | 2007–2009 | Ports of eastern Canada | [10] |
| | | 2007–2009 | West and east coasts of Canada | [4] |

| | | | | |
|---|---|---|---|---|
| 132 | *Prorocentrum micans* | 2003 | Slovenian Sea | [12] |
| | | 2013–2014 | Strait of Gibraltar and Deception Bay, Canada | [18] |
| | | 2002–2004 | Ports on the U.S. East and West Coast | [3] |
| | | 2003–2006 | Port of Tampa and Port Manatee | [5] |
| | | 2007–2009 | South Korean ports | [1] |
| | | 2010–2012 | France | [16] |
| | | 1990–1991 | Laurentian Great Lakes and upper St. Lawrence River | [17] |
| | | 2007–2008 | Patagonian ports, Argentina | [9] |
| | | 1997–2006 | Bilbao Harbour, Spain | [2] |
| | | 1996–1997 | Hongkong, China | [8] |
| | | 2007–2009 | West and east coasts of Canada | [4] |
| | | 2007–2009 | Ports of eastern Canada | [10] |
| | | 2013–2014 | Port of Annaba, Algeria | [11] |
| | | 2015 | Adriatic ports | [6] |
| 133 | *Prorocentrum scutellum* | 1990–1991 | Laurentian Great Lakes and upper St. Lawrence River | [17] |
| | | 2007–2008 | Patagonian ports, Argentina | [9] |
| | | 2013–2014 | Port of Annaba, Algeria | [11] |
| 134 | *Prorocentrum triestinum* | 1997–2006 | Bilbao Harbour, Spain | [2] |
| | | 2015 | Adriatic ports | [6] |
| 135 | *Prorocentrum gracile* | 2003 | Slovenian Sea | [12] |
| 136 | *Protoceratium reticulatum* | 2013–2014 | Strait of Gibraltar and Deception Bay, Canada | [18] |
| | | 1997–2006 | Bilbao Harbour, Spain | [2] |
| | | 2013–2014 | Port of Annaba, Algeria | [11] |
| | (vegetative cell and cyst) | 2007–2009 | Ports of eastern Canada | [10] |
| | (vegetative cell and cyst) | 2007–2009 | West and east coasts of Canada | [4] |
| 137 | *Protoperidinium americanum* | 2007–2009 | West and east coasts of Canada | [4] |
| | | 2007–2009 | Ports of eastern Canada | [10] |

| | | | | |
|---|---|---|---|---|
| 138 | *Protoperidinium bipes* | 2007 | Vladivostok Port | [19] |
| | | 2004/2005/2007 | Novorossiysk, Russia | [20] |
| | | 2007–2009 | West and east coasts of Canada | [4] |
| | | 2007–2009 | Ports of eastern Canada | [10] |
| 139 | *Protoperidinium brevipes* | 2002–2004 | Ports on the U.S. East and West Coast | [3] |
| | | 2007–2009 | West and east coasts of Canada | [4] |
| | | 2007–2009 | Ports of eastern Canada | [10] |
| 140 | *Protoperidinium brochii* | 2013–2014 | Port of Annaba, Algeria | [11] |
| 141 | *Protoperidinium capurroi* | 2007–2008 | Patagonian ports, Argentina | [9] |
| 142 | *Protoperidinium cerasus* | 2007–2009 | West and east coasts of Canada | [4] |
| | | 2007–2009 | Ports of eastern Canada | [10] |
| | | 2013–2014 | Port of Annaba, Algeria | [11] |
| 143 | *Protoperidinium claudicans* | 1995 | Estuary and Gulf of St. Lawrence | [14] |
| | | 2007–2009 | South Korean ports | [1] |
| | | 2007–2009 | West and east coasts of Canada | [4] |
| | | 2007–2009 | Ports of eastern Canada | [10] |
| 144 | *Protoperidinium conicoides* | 2007–2009 | West and east coasts of Canada | [4] |
| | | 2007–2009 | Ports of eastern Canada | [10] |
| 145 | *Protoperidinium conicum* | 2007–2009 | Ports of eastern Canada | [10] |
| | | 2010 | Ulsan and Onsan ports, Korea | [15] |
| | | 2013–2014 | Strait of Gibraltar and Deception Bay, Canada | [18] |
| | | 2002–2004 | Ports on the U.S. East and West Coast | [3] |
| | | 2003–2006 | Port of Tampa and Port Manatee | [5] |
| | | 2007–2009 | South Korean ports | [1] |
| | | 2007–2008 | Patagonian ports, Argentina | [9] |
| | | 2007–2009 | West and east coasts of Canada | [4] |
| | | 2013–2014 | Port of Annaba, Algeria | [11] |
| 146 | *Protoperidinium crassipes* | 2003–2006 | Port of Tampa and Port Manatee | [5] |
| | | 2007–2008 | Patagonian ports, Argentina | [9] |
| | | 2007–2009 | West and east coasts of Canada | [4] |
| | | 2007–2009 | Ports of eastern Canada | [10] |

| | | | | |
|---|---|---|---|---|
| | | 2015 | Adriatic ports | [6] |
| 147 | *Protoperidinium curtipes* | 1995 | Estuary and Gulf of St. Lawrence | [14] |
| | | 2007–2009 | West and east coasts of Canada | [4] |
| | | 2007–2009 | Ports of eastern Canada | [10] |
| 148 | *Protoperidinium curvipes* | 2007–2009 | West and east coasts of Canada | [4] |
| | | 2013–2014 | Port of Annaba, Algeria | [11] |
| 149 | *Protoperidinium decipiens* | 2007–2009 | Ports of eastern Canada | [10] |
| | | 2007–2009 | West and east coasts of Canada | [4] |
| 150 | *Protoperidinium denticulatum* | 1995 | Estuary and Gulf of St. Lawrence | [14] |
| | | 2007–2008 | Patagonian ports, Argentina | [9] |
| | | 2007–2009 | West and east coasts of Canada | [4] |
| | | 2007–2009 | Ports of eastern Canada | [10] |
| 151 | *Protoperidinium depressum* | 2002–2004 | Ports on the U.S. East and West Coast | [3] |
| | | 2003–2006 | Port of Tampa and Port Manatee | [5] |
| | | 1995 | Estuary and Gulf of St. Lawrence | [14] |
| | | 2007–2009 | West and east coasts of Canada | [4] |
| | | 2007–2009 | Ports of eastern Canada | [10] |
| | | 2007–2008 | Patagonian ports, Argentina | [9] |
| 152 | *Protoperidinium diabolus* | 2007–2009 | Ports of eastern Canada | [10] |
| | | 2007–2009 | West and east coasts of Canada | [4] |
| 153 | *Protoperidinium divaricatum* | 2007–2008 | Patagonian ports, Argentina | [9] |
| 154 | *Protoperidinium divergens* | 2007–2009 | Ports of eastern Canada | [10] |
| | | 1995 | Estuary and Gulf of St. Lawrence | [14] |
| | | 2007–2009 | South Korean ports | [1] |
| | | 2007–2009 | West and east coasts of Canada | [4] |
| | | 2013–2014 | Port of Annaba, Algeria | [11] |
| 155 | *Protoperidinium excentricum* | 2007–2008 | Patagonian ports, Argentina | [9] |
| | | 2007–2009 | West and east coasts of Canada | [4] |
| 156 | *Protoperidinium expansum* | 2007–2008 | Patagonian ports, Argentina | [9] |
| 157 | *Protoperidinium globulus* | 2007–2009 | Ports of eastern Canada | [10] |
| | | 2007–2009 | West and east coasts of Canada | [4] |

| | | | | |
|---|---|---|---|---|
| 158 | *Protoperidinium granii* | 2007–2009 | Ports of eastern Canada | [10] |
| | | 2007–2009 | West and east coasts of Canada | [4] |
| | | 2013–2014 | Port of Annaba, Algeria | [11] |
| 159 | *Protoperidinium laticeps* | 2007–2009 | Ports of eastern Canada | [10] |
| | | 2007–2009 | West and east coasts of Canada | [4] |
| 160 | *Protoperidinium leonis* | 1990–1991 | Laurentian Great Lakes and upper St. Lawrence River | Reported as *Peridinium leonis* [17] |
| | | 1995 | Estuary and Gulf of St. Lawrence | [14] |
| | | 2007–2008 | Patagonian ports, Argentina | [9] |
| | | 2007–2009 | West and east coasts of Canada | [4] |
| | | 2007–2009 | Ports of eastern Canada | [10] |
| | | 2013–2014 | Port of Annaba, Algeria | [11] |
| 161 | *Protoperidinium marielebourae* | 2007–2009 | Ports of eastern Canada | [10] |
| | | 2007–2009 | West and east coasts of Canada | [4] |
| 162 | *Protoperidinium mite* | 2007–2009 | Ports of eastern Canada | [10] |
| | | 2007–2009 | West and east coasts of Canada | [4] |
| 163 | *Protoperidinium nudum* | 2007–2009 | West and east coasts of Canada | [4] |
| 164 | *Protoperidinium oblongum* | 2007–2009 | Ports of eastern Canada | [10] |
| | | 2007–2009 | West and east coasts of Canada | [4] |
| 165 | *Protoperidinium obtusum* | 2007–2008 | Patagonian ports, Argentina | [9] |
| | | 2007–2008 | Patagonian ports, Argentina | [9] |
| | | 2007–2009 | West and east coasts of Canada | [4] |
| | | 2007–2009 | Ports of eastern Canada | [10] |
| 166 | *Protoperidinium oceanicum* | 1995 | Estuary and Gulf of St. Lawrence | [14] |
| | | 2007–2009 | West and east coasts of Canada | [4] |
| | | 2007–2009 | Ports of eastern Canada | [10] |
| | | 2013–2014 | Port of Annaba, Algeria | [11] |
| 167 | *Protoperidinium ovatum* | 2007–2009 | Ports of eastern Canada | [10] |
| | | 1995 | Estuary and Gulf of St. Lawrence | [14] |
| | | 2007–2009 | | [1] |
| | | 2007–2009 | West and east coasts of Canada | [4] |

| | | | | |
|---|---|---|---|---|
| 168 | *Protoperidinium pallidum* | 2007–2009 | Ports of eastern Canada | [10] |
| | | 2002–2004 | Ports on the U.S. East and West Coast | [3] |
| | | 1995 | Estuary and Gulf of St. Lawrence | [14] |
| | | 2007 | Vladivostok Port | [19] |
| | | 2004/2005/2007 | Novorossiysk, Russia | [20] |
| | | 2007–2009 | West and east coasts of Canada | [4] |
| | | 2007–2009 | South Korean ports | [1] |
| 169 | *Protoperidinium parapyriforme* | 2007–2008 | Patagonian ports, Argentina | [9] |
| 170 | *Protoperidinium pellucidum* | 2007–2009 | Ports of eastern Canada | [10] |
| | | 2002–2004 | Ports on the U.S. East and West Coast | [3] |
| | | 2003 | Slovenian Sea | [12] |
| | | 2003–2006 | Port of Tampa and Port Manatee | [5] |
| | | 2007–2009 | South Korean ports | [1] |
| | | 2007 | Vladivostok Port | [19] |
| | | 2004/2005/2007 | Novorossiysk, Russia | [20] |
| | | 2008 | Tunisian harbours of La Goulette and Biz– erte | [21] |
| | | 2007–2009 | West and east coasts of Canada | [4] |
| | | 2013–2014 | Port of Annaba, Algeria | [11] |
| 171 | *Protoperidinium pentagonum* | 2002–2004 | Ports on the U.S. East and West Coast | [3] |
| | | 1995 | Estuary and Gulf of St. Lawrence | [14] |
| | | 2007–2008 | Patagonian ports, Argentina | [9] |
| | | 2007–2009 | West and east coasts of Canada | [4] |
| | | 2013–2014 | Port of Annaba, Algeria | [11] |
| 172 | *Protoperidinium punctulatum* | 2007–2009 | West and east coasts of Canada | [4] |
| | | 2007–2008 | Patagonian ports, Argentina | [9] |
| | | 2007–2008 | Patagonian ports, Argentina | [9] |
| 173 | *Protoperidinium pyriforme* | 2007–2009 | Ports of eastern Canada | [10] |
| | | 2007–2009 | West and east coasts of Canada | [4] |
| 174 | *Protoperidinium quarnerense* | 2007–2009 | West and east coasts of Canada | [4] |
| 175 | *Protoperidinium rosaceum* | 2007–2008 | Patagonian ports, Argentina | [9] |
| 176 | *Protoperidinium saltans* | 2007–2009 | West and east coasts of Canada | [4] |

| | | | | |
|---|---|---|---|---|
| | | 2007–2009 | Ports of eastern Canada | [10] |
| 177 | *Protoperidinium sphaeroideum* | 2007–2009 | Ports of eastern Canada | [10] |
| | | 2007–2009 | West and east coasts of Canada | [4] |
| 178 | *Protoperidinium steinii* | 2007–2009 | Ports of eastern Canada | [10] |
| | | 2013–2014 | Strait of Gibraltar and Deception Bay, Canada | [18] |
| | | 2003 | Slovenian Sea | [12] |
| | | 2007–2009 | West and east coasts of Canada | [4] |
| | | 2013–2014 | Port of Annaba, Algeria | [11] |
| | | 2015 | Adriatic ports | [6] |
| 179 | *Protoperidinium subcrassipes* | 2007–2008 | Patagonian ports, Argentina | [9] |
| 180 | *Protoperidinium subinerme* | 2007–2009 | Ports of eastern Canada | [10] |
| | | 2013–2014 | Strait of Gibraltar and Deception Bay, Canada | [18] |
| | | 2007–2009 | West and east coasts of Canada | [4] |
| 181 | *Protoperidinium thorianum* | 2007–2009 | West and east coasts of Canada | [4] |
| | | 2007–2009 | Ports of eastern Canada | [10] |
| 182 | *Protoperidinium thulesense* | 2007–2009 | West and east coasts of Canada | [4] |
| | | 2007–2009 | Ports of eastern Canada | [10] |
| 183 | *Protoperidinium tristylum* | 2007–2009 | Ports of eastern Canada | [10] |
| | | 2007–2009 | West and east coasts of Canada | [4] |
| 184 | *Protoperidinium verrucosum* | 2007 | Vladivostok Port | [19] |
| | | 2004/2005/2007 | Novorossiysk, Russia | [20] |
| 185 | *Pyrocystis lunula* | 2015 | Adriatic ports | [6] |
| 186 | *Pyrophacus horologium* | 1995 | Estuary and Gulf of St. Lawrence | [14] |
| | | 2007–2009 | West and east coasts of Canada | [4] |
| | | 2007–2009 | Ports of eastern Canada | [10] |
| 187 | *Pyrophacus steinii* | 2003–2006 | Port of Tampa and Port Manatee | [5] |
| | | 2007–2009 | South Korean ports | [1] |
| 188 | *Scrippsiella acuminata* | 2007–2009 | Ports of eastern Canada | Reported as *Scrippsiella trochoidea* [10] |
| | | 2013–2014 | Strait of Gibraltar and Deception Bay, Canada | Reported as *Scrippsiella trochoidea* [18] |
| | | 2002–2004 | Ports on the U.S. East and West Coast | Reported as *Scrippsiella trochoidea* [3] |
| | | 2007–2009 | South Korean ports | Reported as *Scrippsiella trochoidea* [1] |

| | | | | |
|---|---|---|---|---|
| | | 1990–1991 | Laurentian Great Lakes and upper St. Lawrence River | Reported as *Scrippsiella trochoidea* [17] |
| | | 2007–2008 | Patagonian ports, Argentina | Reported as *Scrippsiella trochoidea* [9] |
| | | 2007–2009 | West and east coasts of Canada | Reported as *Scrippsiella trochoidea* [4] |
| | | 1996–1997 | Hongkong, China | Reported as *Scrippsiella trochoidea* [8] |
| | | 2013–2014 | Port of Annaba, Algeria | Reported as *Scrippsiella trochoidea* [11] |
| | | 2015 | Adriatic ports | Reported as *Scrippsiella trochoidea* [6] |
| 189 | *Spiraulax kofoidii* | 2007–2009 | West and east coasts of Canada | [4] |
| | | 2007–2009 | Ports of eastern Canada | [10] |
| 190 | *Torodinium robustum* | 2007–2009 | West and east coasts of Canada | [4] |
| 191 | *Triadinium polyedricum* | 2007–2009 | West and east coasts of Canada | Reported as *Goniodoma polyedricum* [4] |
| | | 2007–2009 | Ports of eastern Canada | Reported as *Goniodoma polyedricum* [10] |
| 192 | *Tripos arcticus* | 2007–2008 | Patagonian ports, Argentina | Reported as *Ceratium arcticum* [9] |
| | | 1995 | Estuary and Gulf of St. Lawrence | Reported as *Ceratium arcticum* [14] |
| | | 2007–2009 | West and east coasts of Canada | [4] |
| 193 | *Tripos arietinu* | 2007–2009 | Ports of eastern Canada | Reported as *Neoceratium arietinum* [10] |
| 194 | *Tripos arietinum* | 1995 | Estuary and Gulf of St. Lawrence | Reported as *Ceratium arietinum* [14] |
| 195 | *Tripos arietinus* | 2007–2009 | West and east coasts of Canada | [4] |
| 196 | *Tripos azoricu* | 2007–2009 | Ports of eastern Canada | Reprted as *Neoceratium azoricum* [10] |
| 197 | *Tripos azoricum* | 2007–2008 | Patagonian ports, Argentina | Reported as *Ceratium azoricum* [9] |
| | | 1995 | Estuary and Gulf of St. Lawrence | Reported as *Ceratium azoricum* [14] |
| 198 | *Tripos azoricus* | 2007–2009 | West and east coasts of Canada | [4] |
| 199 | *Tripos balechii* | 2007–2009 | West and east coasts of Canada | [4] |
| 200 | *Tripos candelabrum* | 2002–2004 | Ports on the U.S. East and West Coast | Reported as *Ceratium candelabrum* [3] |
| | | 2007–2008 | Patagonian ports, Argentina | Reported as *Ceratium candelabrum* [9] |
| | | 2007–2009 | Ports of eastern Canada | Reported as *Neoceratium candelabrum* [10] |
| 201 | *Tripos candelabrus* | 2007–2009 | West and east coasts of Canada | [4] |
| | | 2015 | Adriatic ports | [6] |
| 202 | *Tripos carriensis* | 2015 | Adriatic ports | [6] |
| 203 | *Tripos compressum* | 2007–2009 | Ports of eastern Canada | Reported as *Neoceratium compressum* [10] |
| 204 | *Tripos compressus* | 2007–2009 | West and east coasts of Canada | [4] |

| | | | | |
|---|---|---|---|---|
| 205 | *Tripos concilians* | 1995 | Estuary and Gulf of St. Lawrence | Reported as *Ceratium concilians* [14] |
| 206 | *Tripos contortum* | 1995 | Estuary and Gulf of St. Lawrence | Reported as *eratium contortum* [14] |
| 207 | *Tripos declinatum* | 2013–2014 | Port of Annaba, Algeria | [11] |
| 208 | *Tripos dens* | 2007–2008 | Patagonian ports, Argentina | Reported as *Ceratium dens* [9] |
| 209 | *Tripos divaricatum* | 2007–2009 | Ports of eastern Canada | Reported as *Neoceratium divaricatum* [10] |
| 210 | *Tripos euarcuatum* | 1995 | Estuary and Gulf of St. Lawrence | Reported as *Ceratium euarcuatum* [14] |
| 211 | *Tripos extensum* | 1995 | Estuary and Gulf of St. Lawrence | Reported as *Ceratium extensum* [14] |
| | | 2013–2014 | Port of Annaba, Algeria | [11] |
| 212 | *Tripos furca* | 2013–2014 | Strait of Gibraltar and Deception Bay, Canada | Reported as *Neoceratium furca* [18] |
| | | 2007–2009 | Ports of eastern Canada | Reported as *Neoceratium furca* [10] |
| | | 2007–2009 | West and east coasts of Canada | [4] |
| | | 2013–2014 | Port of Annaba, Algeria | [11] |
| | | 2015 | Adriatic ports | [6] |
| 213 | *Tripos fusus* | 2010 | Ulsan and Onsan ports, Korea | Reported as *Ceratium fusus* [15] |
| | | 2002–2004 | Ports on the U.S. East and West Coast | Reported as *Ceratium fusus* [3] |
| | | 2003 | Slovenian Sea | Reported as *Ceratium fusus* [12] |
| | | 1995 | Estuary and Gulf of St. Lawrence | Reported as *Ceratium fusus* [14] |
| | | 2007–2009 | South Korean ports | Reported as *Ceratium fusus* [1] |
| | | 2010–2012 | France | Reported as *Ceratium fusus* [16] |
| | | 1990–1991 | Laurentian Great Lakes and upper St. Lawrence River | Reported as *Ceratium fusus* [17] |
| | | 2007–2008 | Patagonian ports, Argentina | Reported as *Ceratium fusus* [9] |
| | | 2013–2014 | Strait of Gibraltar and Deception Bay, Canada | Reported as *Neoceratium fusus* [18] |
| | | 2007–2009 | Ports of eastern Canada | Reported as *Neoceratium fusus* [10] |
| | | 2007–2009 | West and east coasts of Canada | [4] |
| | | 2013–2014 | Port of Annaba, Algeria | [11] |
| | | 2015 | Adriatic ports | [6] |
| 214 | *Tripos gibberus* | 2007–2009 | West and east coasts of Canada | [4] |
| 215 | *Tripos gravidum* | 1995 | Estuary and Gulf of St. Lawrence | Reported as *Ceratium gravidum* [14] |
| 216 | *Tripos hexacanthum* | 1995 | Estuary and Gulf of St. Lawrence | Reported as *Ceratium hexacanthum* [14] |

| | | | | |
|---|---|---|---|---|
| | | 1990–1991 | Laurentian Great Lakes and upper St. Lawrence River | Reported as *Ceratium hexacanthum* [17] |
| 217 | *Tripos hircus* | 2003–2006 | Port of Tampa and Port Manatee | Reported as *Ceratium hircus* [5] |
| 218 | *Tripos horridum* | 2007–2009 | Ports of eastern Canada | Reported as *Neoceratium horridum* [10] |
| 219 | *Tripos horridus* | 2007–2009 | West and east coasts of Canada | [4] |
| | | 2013–2014 | Port of Annaba, Algeria | Reported as *Tripos horridum* [11] |
| 220 | *Tripos inflatus* | 1995 | Estuary and Gulf of St. Lawrence | Reported as *Ceratium inflatum* [14] |
| | | 1990–1991 | Laurentian Great Lakes and upper St. Lawrence River | Reported as *Ceratium inflatum* [17] |
| | | 2007–2009 | West and east coasts of Canada | [4] |
| 222 | *Tripos kofoidii* | 2007–2009 | South Korean ports | Reported as *Ceratium kofoidii* [1] |
| 221 | *Tripos kofoidii* | 2007–2009 | Ports of eastern Canada | Reported as *Neoceratium kofoidii* [10] |
| | | 2007–2009 | West and east coasts of Canada | [4] |
| 222 | *Tripos lineatum* | 2007–2008 | Patagonian ports, Argentina | Reported as *Ceratium lineatum* [9] |
| | | 2007–2009 | South Korean ports | Reported as *Ceratium lineatum* [1] |
| | | 2010–2012 | France | Reported as *Ceratium lineatum* [16] |
| | | 1990–1991 | Laurentian Great Lakes and upper St. Lawrence River | Reported as *Ceratium lineatum* [17] |
| 223 | *Tripos lineatus* | 2007–2009 | West and east coasts of Canada | [4] |
| | | 2013–2014 | Port of Annaba, Algeria | Reported as *Tripos lineatum* [11] |
| 224 | *Tripos longipes* | 1995 | Estuary and Gulf of St. Lawrence | Reported as *Ceratium longipes* [14] |
| | | 2007–2009 | Ports of eastern Canada | Reported as *Neoceratium longipes* [10] |
| | | 2007–2009 | West and east coasts of Canada | [4] |
| 225 | *Tripos longirostrus* | 2007–2009 | West and east coasts of Canada | [4] |
| 226 | *Tripos lunula* | 2002–2004 | Ports on the U.S. East and West Coast | Reported as *Ceratium lunula* [3] |
| | | 2013–2014 | Port of Annaba, Algeria | [11] |
| 227 | *Tripos macroceros* | 2002–2004 | Ports on the U.S. East and West Coast | Reported as *Ceratium macroceros* [3] |
| | | 1995 | Estuary and Gulf of St. Lawrence | Reported as *Ceratium macroceros* [14] |
| | | 2007–2009 | South Korean ports | Reported as *Ceratium macroceros* [1] |
| | | 2010–2012 | France | Reported as *Ceratium macroceros* [16] |
| | | 2007–2008 | Patagonian ports, Argentina | Reported as *Ceratium macroceros* [9] |

| | | | | |
|---|---|---|---|---|
| | | 1995 | Estuary and Gulf of St. Lawrence | Reported as *Ceratium macroceros* [14] |
| | | 2007–2009 | Ports of eastern Canada | Reported as *Neoceratium macroceros* [10] |
| | | 2007–2009 | West and east coasts of Canada | [4] |
| 228 | *Tripos minutum* | 1990–1991 | Laurentian Great Lakes and upper St. Lawrence River | Reported as *Ceratium minutum* [17] |
| | | 2007–2009 | Ports of eastern Canada | Reported as *Neoceratium minutum* [10] |
| 229 | *Tripos minutus* | 2007–2009 | West and east coasts of Canada | [4] |
| 230 | *Tripos muelleri* | 2007–2009 | Ports of eastern Canada | Reported as *Neoceratium tripos* [10] |
| | | 2007–2009 | West and east coasts of Canada | [4] |
| | | 2013–2014 | Port of Annaba, Algeria | Reported as *Tripos tripos* [11] |
| 231 | *Tripos pentagonum* | 2007–2008 | Patagonian ports, Argentina | Reported as *Ceratium pentagonum* [9] |
| | | 1995 | Estuary and Gulf of St. Lawrence | Reported as *Ceratium pentagonum* [14] |
| | | 1990–1991 | Laurentian Great Lakes and upper St. Lawrence River | Reported as *Ceratium pentagonum* [17] |
| | | 2007–2009 | Ports of eastern Canada | Reported as *Neoceratium pentagonum* [10] |
| 232 | *Tripos pentagonus* | 2007–2009 | West and east coasts of Canada | [4] |
| 233 | *Tripos platycorne* | 2007–2009 | Ports of eastern Canada | Reported as *Neoceratium platycorne* [10] |
| 234 | *Tripos platycornis* | 2007–2009 | West and east coasts of Canada | Regarded as *Tripos platycorne* [4] |
| 235 | *Tripos pulchellum* | 2007–2009 | Ports of eastern Canada | Reported as *Neoceratium pulchellum* [10] |
| 236 | *Tripos pulchellus* | 2007–2009 | West and east coasts of Canada | [4] |
| 237 | *Tripos teres* | 2007–2009 | Ports of eastern Canada | Reported as *Neoceratium teres* [10] |
| | | 2007–2009 | West and east coasts of Canada | [4] |
| 238 | *Tryblionella compressa* | 2007–2009 | Ports of eastern Canada | Reported as *Prorocentrum compressum* [10] |
| | | 2003–2006 | Port of Tampa and Port Manatee | Reported as *Prorocentrum compressum* [5] |
| | | 1995 | Estuary and Gulf of St. Lawrence | Reported as *Prorocentrum compressum* [14] |
| | | 2007–2008 | Patagonian ports, Argentina | Reported as *Prorocentrum compressum* [9] |
| | | 2007–2009 | West and east coasts of Canada | Reported as *Prorocentrum compressum* [4] |
| | | 2015 | Adriatic ports | Reported as *Prorocentrum compressum* [6] |

The synonyms of the species were checked according to the information provided by AlgaeBase (http://www.algaebase.org).

1. Hyun, B.; Shin, K.; Jang, M.–C.; Jang, P.–G.; Lee, W.–J.; Park, C.; Choi, K.–H. Potential invasions of phytoplankton in ship ballast water at South Korean ports. *Mar. Freshwater Res.* **2016**, *67*, 1906–1917, doi:http://dx.doi.org/10.1071/MF15170_AC.
2. Butron, A.; Orive, E.; Madariaga, I. Potential risk of harmful algae transport by ballast waters: The case of Bilbao Harbour. *Mar. Pollut. Bull.* **2011**, *62*, 747–757, doi:10.1016/j.marpolbul.2011.01.008.
3. Burkholder, J.M.; Hallegraeff, G.M.; Melia, G.; Cohen, A.; Bowers, H.A.; Oldach, D.W.; Parrow, M.W.; Sullivan, M.J.; Zimba, P.V.; Allen, E.H., et al. Phytoplankton and bacterial assemblages in ballast water of US military ships as a function of port of origin, voyage time, and ocean exchange practices. *Harmful Algae* **2007**, *6*, 486–518, doi:10.1016/j.hal.2006.11.006.
4. Casas–Monroy, O.; Parenteau, M.; Drake, D.A.R.; Roy, S.; Rochon, A. Absolute estimates of the propagule pressure of viable dinoflagellates across Canadian coasts: the variable influence of ballast water exchange XXX. *Mar. Biol.* **2016**, *163*, doi:10.1007/s00227–016–2946–3.
5. Garrett, M.J.; Wolny, J.L.; Williams, B.J.; Dirks, M.D.; Brame, J.A.; Richardson, R.W. Methods for sampling and analysis of marine microalgae in ship ballast tanks: a case study from Tampa Bay, Florida, USA. *Algae* **2011**, *26*, 181–192.
6. Cabrini, M.; Cerino, F.; de Olazabal, A.; Di Poi, E.; Fabbro, C.; Fornasaro, D.; Goruppi, A.; Flander–Putrle, V.; France, J.; Gollasch, S., et al. Potential transfer of aquatic organisms via ballast water with a particular focus on harmful and non–indigenous species: A survey from Adriatic ports. *Mar. Pollut. Bull.* **2018**, *14*, 331–338, doi:10.1016/j.marpolbul.2018.02.004.
7. Steichen, J.L.; Schulze, A.; Brinkmeyer, R.; Quigg, A. All aboard! A biological survey of ballast water onboard vessels spanning the North Atlantic Ocean. *Mar. Pollut. Bull.* **2014**, *87*, 201–210, doi:10.1016/j.marpolbul.2014.07.058.
8. Zhang, F.Z.; Dickman, M. Mid–ocean exchange of container vessel ballast water. 1: Seasonal factors affecting the transport of harmful diatoms and dinoflagellates. *Marine Ecology Progress* **1999**, *176*, 243–251.
9. Boltovskoy, D.; Almada, P.; Correa, N. Biological invasions: assessment of threat from ballast–water discharge in Patagonian (Argentina) ports. *Environ. Sci. Policy* **2011**, *14*, 578–583, doi:10.1016/j.envsci.2011.03.007.
10. Roy, S.; Parenteau, M.; Casas–Monroy, O.; Rochon, A. Coastal ship traffic: a significant introduction vector for potentially harmful dinoflagellates in eastern Canada. *Can. J. Fish. Aquat.Sci.* **2012**, *69*, 627–644, doi:10.1139/f2012–008.
11. Cheniti, R.; Rochon, A.; Frihi, H. Ship traffic and the introduction of diatoms and dinoflagellates via ballast water in the port of Annaba, Algeria. *J. Sea Res.* **2018**, *133*, 154–165, doi:10.1016/j.seares.2017.07.008.
12. David, M.; Gollasch, S.; Cabrini, M.; Perkovic, M.; Bosnjak, D.; Virgilio, D. Results from the first ballast water sampling study in the Mediterranean Sea – the Port of Koper study. *Mar. Pollut. Bull.* **2007**, *54*, 53–65, doi:10.1016/j.marpolbul.2006.08.041.
13. Olenin, S.; Gollasch, S.; Jonusas, S.; Rimkute, I. En–route investigations of plankton in ballast water on a ship's voyage from the Baltic Sea to the open Atlantic coast of Europe.

*Int. Rev. Hydrobiol.* **2000**, *85*, 577–596, doi:10.1002/1522–2632(200011)85:5/6<577::aid–iroh577>3.0.co;2–c.

14. Harvey, M.; Gilbert, M.; Gauthierl, D.; Reid, D.M. A preliminary assessment of risks for the ballast water–mediated introduction of nonindigenous marine organisms in the Estuary and Gulf of St. Lawrence. *Journal of Geo–Information Science* **1999**, *12*, 89–94.
15. Baek, S.H.; Jung, S.W.; Jang, M.C.; Hyun, B.; Shin, K. Survival potential of autotrophic phytoplankton species collected from ballast water in international commercial ships. *N. Z. J. Mar. Freshwater Res.* **2012**, *46*, 125–136, doi:10.1080/00288330.2011.610326.
16. Masson, D.; Thomas, G.; Genauzeau, S.; Le Moine, O.; Derrien, A. Merchant ships discharging unwanted marine species in close proximity of a French aquaculture area: Risks involved. *Mar. Pollut. Bull.* **2013**, *77*, 315–319, doi:10.1016/j.marpolbul.2013.09.028.
17. Rao, D.V.S.; Sprules, W.G.; Locke, A.; Carlton, J.T. Exotic phytoplankton from ships' ballast waters: Risk of potential spread to mariculture sites on Canada's East Coast. **1994**.
18. Briski, E.; Gollasch, S.; David, M.; Linley, R.D.; Casas–Monroy, O.; Rajakaruna, H.; Bailey, S.A. Combining ballast water exchange and treatment to maximize prevention of species introductions to freshwater ecosystems. *Environ. Sci. Technol.* **2015**, *49*, 9566–9573, doi:10.1021/acs.est.5b01795.
19. Zvyagintsev, A.Y.; Ivin, V.V.; Kashin, I.A.; Orlova, T.Y.; Selina, M.S.; Kasyan, V.V.; Korn, O.M.; Kornienko, E.S.; Kulikova, V.A.; Bezverbnaya, I.P., et al. Acclimation and introduction of hydrobionts ships' ballast water organisms in the Port of Vladivostok. *Russ. J. Mar. Biol.* **2009**, *35*, 41–52, doi:10.1134/s1063074009010076.
20. Zvyagintsev, A.Y.; Selifonova, J.P. Hydrobiological studies of the ballast waters of cargo ships in Russian Sea ports. *Oceanology* **2010**, *50*, 924–932, doi:10.1134/s0001437010060123.
21. Zmerli, H.T.; Yahia–Kefi, O.D. Have non–indigenous planktonic species been introduced via ballast waters in two North African ports (la Goulette and Bizerte, Tunisia)? *Vie Et Milieu–Life and Environment* **2012**, *62*, 1–9.
22. Galil, B.S.; Hulsmann, N. Protist transport via ballast water – Biological classification of ballast tanks by food web interactions. *European Journal of Protistology* **1997**, *33*, 244–253.
23. Steichen, J.L.; Denby, A.; Windham, R.; Brinkmeyer, R.; Quigg, A. A tale of two ports: dinoflagellate and diatom communities found in the high ship traffic region of Galveston Bay, Texas (USA). *Journal of Coastal Research* **2015**, *31*, 407–416, doi:10.2112/jcoastres–d–13–00225.1.
24. Selifonova, Z.P. Marine biological invasions in waters of the port of Novorossiysk in the Black Sea. *Russ. J. Mar. Biol.* **2009**, *35*, 242–249, doi:10.1134/s1063074009030080.
25. DiBacco, C.; Humphrey, D.B.; Nasmith, L.E.; Levings, C.D. Ballast water transport of non–indigenous zooplankton to Canadian ports. *ICES J. Mar. Sci.* **2012**, *69*, 483–491, doi:10.1093/icesjms/fsr133.
26. Park, T.–G.; Bolch, C.J.S.; Hallegraeff, G.M. Morphological and molecular genetic characterization of Cryptoperidiniopsis brodyi (Dinophyceae) from Australia–wide isolates. *Harmful Algae* **2007**, *6*, 718–733, doi:http://dx.doi.org/10.1016/j.hal.2007.02.004.
27. Drake, L.A.; Meyer, A.E.; Forsberg, R.L.; Baier, R.E.; Doblin, M.A.; Heinemann, S.; Johnson, W.P.; Koch, M.; Rublee, P.A.; Dobbs, F.C. Potential invasion of microorganisms and pathogens via 'interior hull fouling': biofilms inside ballast water tanks. *Biological Invasions* **2005**, *7*, 969–982, doi:10.1007/s10530–004–3001–8.

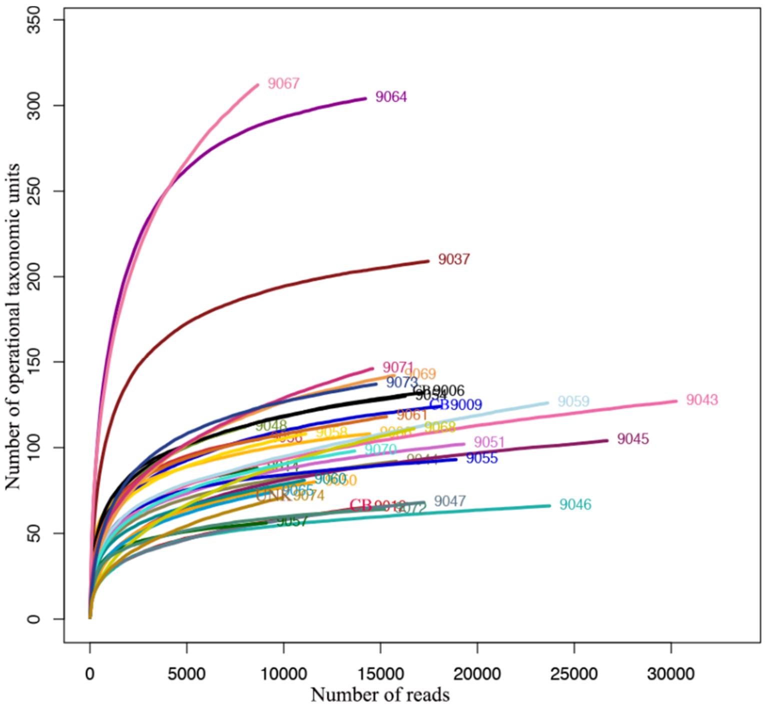


**Figure S1.** Rarefaction curves for all 32 samples showing the sequencing depths.

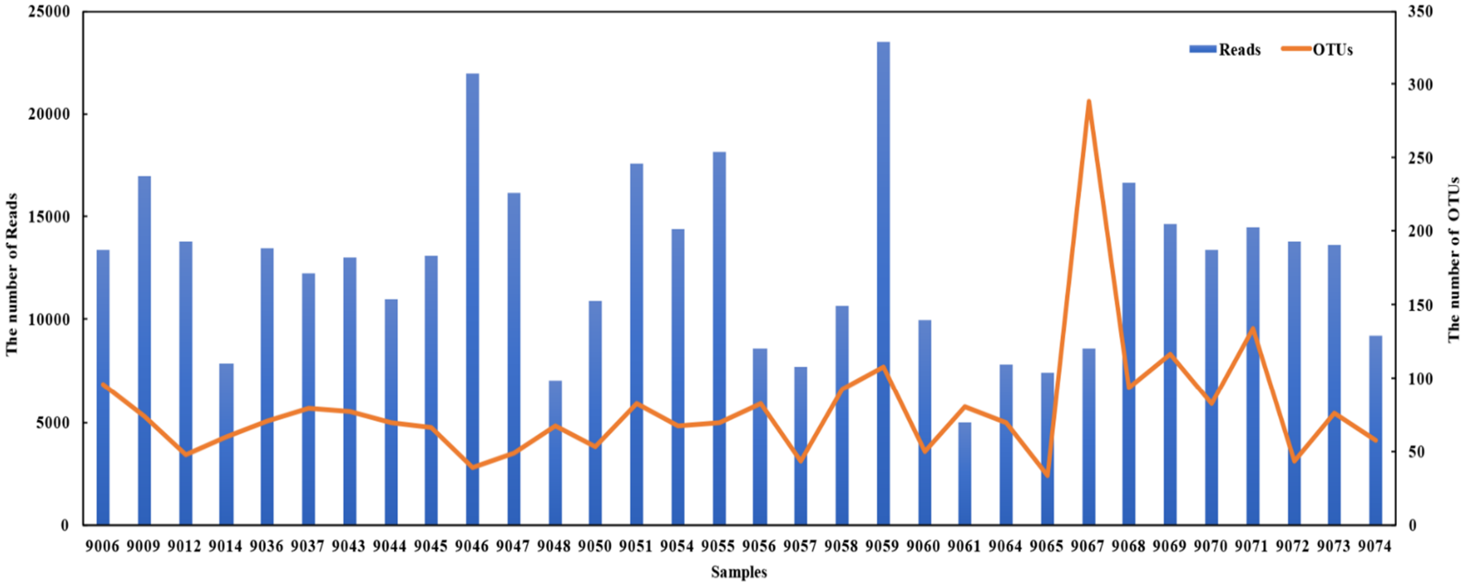


**Figure S2.** The total number of operational taxonomic units (OTUs) and reads annotated as dinoflagellates in the 32 samples.
